# Development and pilot testing of a decision aid for navigating breast cancer survivorship care

**DOI:** 10.1186/s12911-022-02056-5

**Published:** 2022-12-15

**Authors:** Yu Ke, Ivy Cheng, Gretchen Ser Hua Tan, Rose Wai Yee Fok, Jack Junjie Chan, Kiley Wei-Jen Loh, Alexandre Chan

**Affiliations:** 1 Department of Pharmacy, National University of Singapore, Singapore, Singapore; 2 Department of Pharmacy, National Cancer Centre Singapore, Singapore, Singapore; 3 Division of Medical Oncology, National Cancer Centre Singapore, Singapore, Singapore; 4 Division of Supportive and Palliative Care, National Cancer Centre Singapore, Singapore, Singapore; 5 Duke-NUS Medical School Singapore, Singapore, Singapore; 6 Department of Clinical Pharmacy Practice, University of California Irvine, 515 Bison Modular 147B, Irvine, CA 92697 USA

**Keywords:** Cancer, Alpha testing, Development, Survivorship, Decision aid, Shared decision making, Care models

## Abstract

**Background:**

The predominant oncologist-led model in many countries is unsustainable to meet the needs of a growing cohort of breast cancer survivors (BCS). Despite available alternative models, adoption rates have been poor. To help BCS navigate survivorship care, we aimed to systematically develop a decision aid (DA) to guide their choice of follow-up care model and evaluate its acceptability and usability among BCS and health care providers (HCPs).

**Methods:**

We recruited BCS aged ≥ 21 years who have completed primary treatment and understand English. BCS receiving palliative care or with cognitive impairment were excluded. HCPs who routinely discussed post-treatment care with BCS were purposively sampled based on disciplines. Each participant reviewed the DA during a semi-structured interview using the ‘think aloud’ approach and completed an acceptability questionnaire. Descriptive statistics and directed content analysis were used.

**Results:**

We conducted three rounds of alpha testing with 15 BCS and 8 HCPs. All BCS found the final DA prototype easy to navigate with sufficient interactivity. The information imbalance favouring the shared care option perceived by 60% of BCS in early rounds was rectified. The length of DA was optimized to be ‘just right’. Key revisions made included (1) presenting care options side-by-side to improve perceived information balance, (2) creating dedicated sections explaining HCPs’ care roles to address gaps in health system contextual knowledge, and (3) employing a multicriteria decision analysis method for preference clarification exercise to reflect the user’s openness towards shared care. Most BCS (73%) found the DA useful for decision-making, and 93% were willing to discuss the DA with their HCPs. Most HCPs (88%) agreed that the DA was a reliable tool and would be easily integrated into routine care.

**Conclusions:**

Our experience highlighted the need to provide contextual information on the health care system for decisions related to care delivery. Developers should address potential variability within the care model and clarify inherent biases, such as low confidence levels in primary care. Future work could expand on the developed DA’s informational structure to apply to other care models and leverage artificial intelligence to optimize information delivery.

**Supplementary Information:**

The online version contains supplementary material available at 10.1186/s12911-022-02056-5.

## Background

Globally, a rising incidence of breast cancer diagnoses with low mortality rates resulted in an increasing pool of breast cancer survivors (BCS) with long-term care needs [1]. Most countries adopted the oncologist-led model where breast cancer-related follow-up care is concentrated in specialist settings [2]. However, the extensive utilization of oncologist services in the post-treatment survivorship phase significantly strains the current capacity of cancer centres, rendering the oncologist-led model unsustainable in addressing survivors’ unmet needs [3]. In response, countries began trialling alternative care models with greater involvement of primary care health care providers (HCPs) [3–7]. Despite evidence suggesting comparable effectiveness to the oncologist-led model, adoption rates of alternative models were poor, prompting calls for strategies to guide model selection [3, 5]. Furthermore, it is increasingly important to empower BCS in this decision-making process to ensure that the chosen model aligns with each survivor’s preferences, maximizing the relevance and value of survivorship care [3, 8–10].

To help BCS navigate through possible breast cancer survivorship care models, decision aids (DAs) could be utilized [11]. A DA is a tool designed to provide neutral, balanced, and evidence-based information on the possible care options. Additionally, it elicits users’ preferences to exemplify the trade-offs between options to make an informed decision [12]. Complementing DA development, artificial intelligence (AI) tools are increasingly explored and embedded within DAs to enhance personalized information delivery and communication [13, 14]. While available evidence demonstrated favourable outcomes of DA usage in increasing knowledge, reducing decisional conflicts, and enhancing satisfaction for cancer-related decisions [15–17], there was a disproportionate focus on screening and treatment decisions. A recent systematic review focusing on health services or care modality decisions after primary cancer treatment identified a Dutch study that examined DA usage for breast cancer follow-up care intensity in hospitals [18]. The study showed promising results in improving shared decision-making and reducing cost [19]. Recognizing that cancer survivorship care follow-up options are context- and cultural-specific, direct extrapolation of this DA across countries would be suboptimal.

Singapore is a high-resource country in Southeast Asia where the oncologist-led model is the predominant follow-up modality for BCS [20]. Survivorship care development is at its infancy stage in Singapore, where primary care delivery was rated less favorably than in Western countries [21]. Thus, a complete discharge to a primary care-led model is likely unacceptable with poor adoption. A shared care model involving oncologists, family physicians, and community pharmacists is then piloted in Singapore with assurance of oncologists’ continued involvement. Complementing evaluation efforts in an ongoing trial (NCT04660188), this study aimed to develop a DA to guide BCS’ choice between oncologist-led and shared care models for cancer follow-up and evaluate its acceptability and usability among prospective users through extensive alpha testing. This development exercise would exemplify efforts to devise strategies for alternative model adoption, relieving the strain on acute care resources.

## Methods

### Study design and setting

As part of the DA development process [22], we utilized a mixed-methods design for the alpha testing phase conducted from October 2019 to April 2022. Adopting a user-centred design approach, the preliminary prototype developed was subjected to an iterative process of testing and revising [23, 24] at the National Cancer Centre Singapore, the largest public ambulatory cancer centre in Singapore. This study was approved by the SingHealth Institutional Review Board (CIRB 2019/2596).

### Theoretical framework

**Fig. 1:**
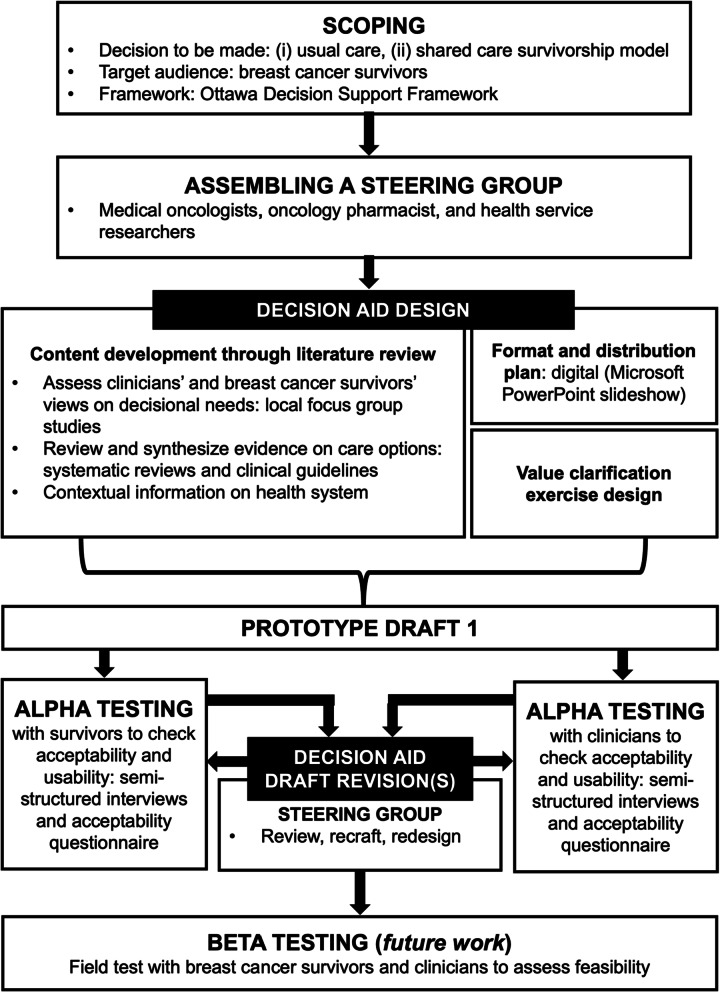
Systematic development process of decision aid for breast cancer survivorship follow-up care, adapted from Coulter et al.

We systematically developed the DA based on the recommendations by Coulter et al., adhering to the International Patient Decision Aid Standards framework (Fig. 1) [22, 25]. A steering group comprising medical oncologists, an oncology pharmacist, and health service researchers without any conflicts of interest supervised the development process. This group ensured that changes made to the DA during testing were appropriate and of clinical relevance.

The prototype design comprised three components (Fig. 1). First, we performed extensive literature review to develop the DA content. We we reviewed decisional needs from BCS’ and primary care HCPs’ perspectives based on two local qualitative studies [26, 27]. Catering to reported informational needs, we designed the DA to address the following content areas: principles of cancer survivorship, roles of participating HCPs, clinical evidence of care models, care coordination strategies, and cost considerations. Published clinical guidelines provided information on the core elements of survivorship care [28–30]. Official institutional webpages provided information on charging and patient resources [31]. The steering group reviewed the description of care models retrieved from the literature [4, 6, 7]. Second, we included a preference clarification exercise to consider BCS’ confidence in primary care HCPs to care for their cancer, an area of concern highlighted by BCS [26]. This exercise prompted users to rate the importance of a list of attributes for their care [32]. Lasty, the preliminary DA prototype (Additional file 1) was developed as an English tool in a digital format using in-built functionalities within the Microsoft PowerPoint and infographics. We then used this preliminary DA prototype in the alpha testing phase to optimize users’ experience and maximize the DA’s usability and acceptability.

### Eligibility criteria and recruitment

Adults aged ≥ 21 years old, formally diagnosed with breast cancer, completed primary treatment (excluding endocrine therapy), and able to read and speak English were eligible. BCS receiving palliative care and/or with cognitive impairment that negatively affects their ability to navigate the DA and articulate their responses were excluded. With the aim of recruiting prospective DA users, eligible participants were identified by medical and surgical oncologists from clinics attended by predominantly BCS. HCPs aged ≥ 21 years old who were involved in discussing post-treatment care with BCS were eligible and contacted by the study team. We excluded HCPs who were steering group members and purposively sampled them to achieve diversity in disciplines. Informed consent was obtained from each participant.

### Data collection

Adopting recommendations for usability testing to be held in small groups each round, we targeted five BCS and two to three HCPs per round [33]. Upon recruitment, BCS participants completed a demographic and clinical questionnaire on their age, race, education level, diagnosis date, cancer stage, and treatment history. HCP participants completed a demographic questionnaire on their profession and years of clinical experience.

Next, we conducted a qualitative interview with each participant as he/she reviewed the DA in person using tablets provided by the study team or via virtual Zoom meetings using the remote-control function. A ‘think aloud’ approach was adopted where the participants would verbalize their immediate thoughts while reviewing the DA [34–36]. The interviewer posed additional questions from the interviewer guide on the content comprehensibility, adequacy, and format (Additional file 2). Additionally, HCPs assessed the DA for accuracy. Each interview lasted approximately 20–40 min and was audio recorded. Lastly, participants completed an acceptability questionnaire adapted from the Ottawa Hospital Research Institute [37]. BCS rated the format, content comprehensibility, and DA’s utility in decision-making, while HCPs provided their perceptions of the DA and its compatibility with their current workflow.

### Data analysis

Descriptive statistics were used to summarize the participant characteristics and the quantitative measures in the acceptability questionnaires. Frequencies and percentages were used for categorical data. Median and range were used for continuous non-normally distributed data. All analyses were performed using STATA version 17.

Interviews were transcribed verbatim and analysed using directed content analysis in Microsoft Excel [38]. In each round, two study team members first reviewed the transcripts to familiarize themselves with the data before coding independently according to key concepts outlined in the interviewer guide. The coders then met to resolve any discrepancies. A summary report of findings and proposed revisions to recurring concerns raised by participants was then discussed with the steering group before finalizing the changes for the next testing round. Due to the iterative nature of alpha testing, data analysis was interspersed with rounds of interviews until thematic saturation. Saturation occurred when the steering group deemed that a new round would not yield additional significant insights.

## Results

### Study participant characteristics

We recruited 15 BCS and 8 HCPs across three rounds of alpha testing. The age range of all BCS was 46–67 years, with the majority being Chinese and diagnosed 8 to 13 years ago. In each round, BCS were sampled from different education levels. All were non-metastatic cases that received surgery. For HCPs, a diverse range of professions was sampled, including medical, radiation, and surgical oncology, nursing, and pharmacy. All HCPs had ≥ 10 years of relevant clinical practice (Table 1).

**Table 1:** Study participant characteristics

Characteristic	Round 1	Round 2	Round 3
Breast cancer survivors	n = 5	n = 5	n = 5
Age, median (range)	51 (46–54)	49 (47–64)	60 (47–67)
Race, n (%)			
Chinese	4 (80%)	4 (80%)	3 (60%)
Malay	1 (20%)	0	2 (40%)
Others	0	1^a^ (20%)	0
Education level, n (%)			
Secondary	1 (20%)	1 (20%)	1 (20%)
Pre-university	1 (20%)	1 (20%)	2 (40%)
Graduate/postgraduate	3 (60%)	3 (60%)	2 (40%)
Survivorship years since diagnosis, median (range)	9 (8–12)	8 (8–10)	7 (10–13)
Breast cancer stage^b^, n (%)			
1	1 (20%)	3 (60%)	1 (20%)
2	2 (40%)	2 (40%)	2 (40%)
3	1 (20%)	0	2 (40%)
Treatment modality, n (%)			
Surgery	5 (100%)	5 (100%)	5 (100%)
Chemotherapy	5 (100%)	5 (100%)	4 (80%)
Radiotherapy	5 (100%)	3 (60%)	4 (80%)
Endocrine therapy	3 (60%)	3 (60%)	3 (60%)

Health care professionals	n = 3	n = 3	n = 2
Discipline, n (%)			
Medical oncology	2 (67%)	0	1 (50%)
Radiation oncology	0	1 (33%)	0
Surgical oncology	0	2 (67%)	0
Nursing	0	0	1 (50%)
Pharmacy	1 (33.3%)	0	0
Years of practice, n (%)			
10–20 years	2 (67%)	3 (100%)	2 (100%)
> 20 years	1 (33%)	0	0

^a^Burmese

^b^One participant from round 1 could not recall her breast cancer stage

### Alpha testing results

We conducted three rounds of iterative testing, with quantitative acceptability measures summarized in Table 2, while the qualitative comments and modifications for each testing round summarized in Table 3.

**Table 2:** Acceptability of decision aid among breast cancer survivors

	Round 1 (n = 5)	Round 2 (n = 5)	Round 3 (n = 5)
**Format**			
Ease of navigation, n (%)			
Easy	5 (100%)	3 (60%)	5 (100%)
Neutral	0	0	0
Difficult	0	2 (40%)	0
Interactivity, n (%)			
Yes	1 (20%)	3 (60%)	4 (80%)
Neutral	3 (60%)	1 (20%)	0
No	1 (20%)	1 (20%)	1 (20%)
**Content of decision aid**			
Balance of information presentation, n (%)			
Slanted towards the usual care option	0	0	0
Balanced	2 (40%)	2 (40%)	5 (100%)
Slanted towards the shared care option	3 (60%)	3 (60%)	0
Length of decision aid, n (%)			
Too long	4 (80%)	1 (20%)	0
Just right	1 (20%)	4 (80%)	5 (100%)
Too short	0	0	0
**Perceived utility for decision-making**			
Helpfulness of preference clarification exercise, n (%)			
Helpful	4 (80%)	3 (60%)	4 (80%)
Neutral	0	1 (20%)	1 (20%)
Not helpful	1 (20%)	1 (20%)	0
Utility in making decision, n (%)			
Useful	3 (60%)	4 (80%)	4 (80%)
Not useful	2 (40%)	1 (20%)	1 (20%)
**Routine use of decision aid**			
Willingness to discuss decision aid with health care professionals, n (%)			
Yes	5 (100%)	4 (80%)	5 (100%)
No	0	1 (20%)	0
Willingness to recommend the decision aid to other cancer survivors, n (%)			
Yes	4 (80%)	4 (80%)	5 (100%)
No	1 (20%)	1 (20%)	0
Preferred mode of distribution, n (%)			
Digital	4 (80%)	4 (80%)	1 (20%)
Neutral	1 (20%)	1 (20%)	3 (60%)
Paper	0	0	1 (20%)
Suitable time to introduce decision aid, n (%)			
Upon diagnosis	1 (20%)	0	1 (20%)
During active treatment	0	0	1 (20%)
Immediately after active treatment	0	3 (60%)	2 (40%)
Years after active treatment	3 (60%)	1 (20%)	1 (20%)
Others	1 (20%)^a^	1 (20%)^b^	0

^a^Participant selects both upon diagnosis and during active treatment

^b^Participant does not want to introduce the decision aid to anyone, thus, did no suitable timing was indicated

**Table 3:** Summary of qualitative comments on prototype for each testing round with key corresponding modifications

Component	Theme	Round	Illustrative quotes	Modifications
Format	Navigation	**Round 1**: Lacked clear directives and instructions on the clickable elements (images, buttons) on each page.	“You must give instructions because just now that one the instructions have, like this one, I wouldn’t know.”—ID01	Added of an example interactive button and standardized navigation buttons across pages.
		**Round 2**: Received positive comments on the intuitiveness of the navigation. However, pop-ups and numerals added to each section was confusing. The example of an interactive button added after round 1 created some confusion.	“I think without the guidance [from interviewer], I won’t know to go to shared care [section by clicking the button].”—ID06 “I see there’s a cross (navigation of popup). Because when you read this way, I suppose I don’t go back there again.”—HCP06	1. Added an orientation section with animation and prompts to orientate users to navigation buttons. 2. For pop-ups, a clear ‘return’ button was added to guide navigation. 3. Changed navigation flow to linear instead of creating possible bidirectionality when users could choose to click into the 'shared care' option before the 'usual care' option.
		**Round 3**: Received affirmative comments on navigation after introducing an orientation guide. Some participants missed clickable buttons on the pages due to colour overuse.	“I think it’s quite clear because you’ve got the click buttons and you got the arrows and everything, it’s so… I think that’s quite clear.”—ID13 “Are the orange words actually clickable?”—HCP07	1. For all clickable portions, a cursor image was added to prompt clicks and this image was included in the orientation navigation guide. 2. Bold/ italics were used for emphasis instead of coloured text.
	Design	**Round 1**: Inconsistencies in colours between buttons and pop-up boxes caused confusion. HCPs raised concerns over the font choice and visual clutter.	“So, you see, the soft tissue cancer here, the colour suddenly becomes the lung cancer colour! So, for me, it’s confusing.”—ID03	1. Standardized the colours of all pop-up boxes with original buttons. 2. Ensured adequate empty space on each page to avoid visual clutter.
		**Round 2**: BCS preferred the use of images and were generally acceptable of the font and size. HCPs provided favourable comments on the aesthetics but suggested more suitable icons in some instances.	“Quite interesting the oncologist is dressed out like the surgeon; it is actually all of us all combined right?”—HCP05 “I think this is a very nice thing and is very pleasantly put together. I think all your graphics.”—HCP06	Amended the graphics used to represent physical effects (replace a flushed woman image) and oncologists to be not suggestive of surgeons exclusively.
		**Round 3**: Received overall positive comments, with suggestions for header font choice and animations.	“Because if you are not a text person, if you see pictures and moving things on the page, it’s easier for you to put your attention to the page rather than just pages.”—ID13	1. Added animations where appropriate for each page. 2. Standardized all fonts to be sans serif and improved colour contrast of headers.
Content	Amount and type of information	**Round 1**: Requested information. • BCS: value and roles of HCPs in shared care, usual care description, support group resources. • HCPs: quantified risk of rare and serious side effects, HCPs involved in shared care, other common treatment side effects.	“You need to paint the current [model]… because… we are trying to get people to consider this new model or embrace this new model. Then you need to paint the current.”—ID03 “Some people may ask what’s the risk [of ovarian cancer] … Small like how small? 1 in 10,000 or 1 in 100,000?”—HCP03	1. Side effects of treatment were reviewed against guidelines and literature, with further quantification of the risks of rare and serious side effects. 2. Separated the discussion of options into the respective dedicated sections to describe usual care and explain differences. 3. Added support group links under additional resources.
		**Round 2**: Requested information. • BCS: relapse management, frequency of treatment side effects, care provision by pharmacists under shared care. • HCPs: frequency of treatment side effects, care provision by pharmacists under shared care, cost.	“My suggestion is maybe you want to put some… like this you put statistics but how about this and this?”—ID06 “And why we want the aftercare because we do not want the relapse. But there is nothing mentioned about the relapse. So read already it is as good as not reading.”—ID07 “So, the navigator is free of charge is it when they call them? You mean have to pay for the navigators?”—HCP05	1. Management of relapse was described through surveillance with a need to counterbalance against excessive generalization of the risk of relapse since it depends on a variety of patient factors. The focus of the DA was not to provide a personalized risk score. 2. Provided statistics of side effects, focusing on rare and serious ones. 3. Supplemented information on pharmacists’ role in shared care.
		**Round 3**: Received affirmative comments on the content coverage, with suggested supplement on information related to psychosocial help sources. One survivor requested further for information on Traditional Chinese Medicine usage.	“Counsellor, is it good maybe to show a website, a web link, which organization can be the counsellor, or this information is at the back?”—ID14 “I think this is good enough because for a start. Unless they really need more then they have to read up more.”—ID11	1. Added a new page providing information related to psychosocial services. 2. No Traditional Chinese Medicine-related information were included due to lack of reliable official sources endorsed by the cancer centre.
	Clarity and presentation	**Round 1**: 1. Significant medical jargon used. 2. The concept ‘late and long-term side effects’ was difficult to comprehend. A timeline with specified milestones was suggested to present this concept. 3. Confusion over the naming convention of HCPs. 4. Suggested ordering side effects by frequency.	“Actually, you can have… pop-up to explain what is the long-term effects as opposed to this, and not just have this inside, separate this… We don’t really understand.”—ID04 “For side effects, you can list the order, like the more common ones or more important ones will be on top.”—HCP03	1. Language used was reviewed and simplified to avoid medical jargons. 2. Replaced words with an animated infographic to explain the concept of late and long-term side effects. 3. Clarified on the types of polyclinic doctors (general vs. regular family physician) and contextualized community pharmacists to their respective retail stores. 4. Ordered treatment side effects by frequency and separated side effects that are rare but serious.
		**Round 2**: 1. Medical jargon usage for treatment side effects. 2. The animated infographic explaining late and long-term side effects was too fast.. 3. Confusion over the naming convention of HCPs in care options persisted and pharmacists’ role under shared care was unclear. 4. Depiction of the shared care model was suboptimal as it appeared as linear instead of a team-based effort.	“It sounds like they are only restricted to only fever and runny nose… and I think the community pharmacists may protest.”—ID06 “Are you sure you see the same doctor at polyclinic?”—ID10	1. Textual changes made to minimize jargons usage. 2. Separated and slowed animations for the late and long-term side effects, respectively, with their descriptions in words. 3. Created a section to elaborate on each type of HCP before presenting the two care options to improve clarity of roles. 4. To depict shared care, HCPs involved were presented in cyclic and triangular format to illustrate the communication flow, instead of a linear flow. The survivorship care plan was featured as a communication tool to facilitate the information flow.
		**Round 3**: Received affirmative comments with suggested fine-tuning: 1. Emphasize actual cost differences between options. 2. Use a human body representation to contextualize treatment side effects for identification. 3. Contrast differences between care options further.	“The language is simple and clear.”—ID13 “I’m thinking whether it will be better if this thing is designed in a way that maybe like under physical effects, you have a[n] image of a person, of a human, like a human figure.”—HCP08 “The information is ok, just that maybe the points [differences] that you want to bring to the patient is not that clear.”—ID12	1. Emphasized cost difference through contrasting cost savings and cost incurred under each care option. 2. Added a human body figure to represent side effects within each treatment category. 3. Emphasized the differences between usual care and shared care options by isolating out comparison factors and presenting the corresponding information side-by-side.
	Accuracy	**Round 2**: Under usual care, HCPs feedbacked that the list of care providers involved are not comprehensive and there was overgeneralization of poor communication.	“If it is just usual care, we also communicate with each other. Not that distinct. We do right but I think the communication may not be as much as shared care. We don’t communicate, but if there is a problem then we communicate.”—HCP05	Amended the description of HCPs involved in usual care and qualified description of communication to avoid overgeneralization.
	Information balance	**Round 1**: Considerably more information was provided on shared care than usual care.	“I think this one [DA] will get you to accept the polyclinic one [shared care option].”—ID02	A dedicated section was created to describe what happens under usual care option.
		**Round 2**: A consensus that the information presented was slanted towards shared care.	“I found the presentation of information to be slanted towards shared [care].”—ID08	Adopting literature recommendations, sections introducing the two care options were rearranged to side-by-side presentation as much as possible.
		**Round 3**: A consensus that the information presented was balanced.	“I think it’s balanced. The information, you can decide what’s… one way or the other.”—ID13	No corresponding changes.
Preference clarification exercise	Consideration factors	**Round 1**: Affirmed the relevance of consideration factors, including HCP characteristics (regularity, expertise), oncologist involvement, cost, location/ convenience, ease of appointment arrangement.	“Because you’re seeing so many different people! At least [at cancer centre], you’re seeing the same doctor, the doctor knows your history, you don’t have to keep repeating to every single person.”—ID05 “They[survivors] say they are worried they are getting an inferior surveillance because they’ve gone from a specialist to a primary care physician.”—HCP01	The list of questions was revised according to reported attributes to increase relevance to survivors. To address concerns of perceived inferiority of primary care HCPs, the statement has been qualified to be ‘trained by oncologists from the cancer centre’.
		**Round 2**: Echoed the relevance of factors explored in questions. HCPs suggested mechanisms to allow prioritizing of factors against one another.	“I think cost is important, convenience is important, but I would be willing to pay twelve dollars more, right?”—HCP06	To weigh in the priority of factors, some preference statements were crafted with qualifiers (e.g., I am willing to explore shared care if it cost cheaper than usual care).
		**Round 3**: Echoed the relevance of factors explored in questions. One survivor disregarded all preference statements on shared care due to preconceived disposition while another HCP suggested including pharmacist in preference statements for usual care to maintain balance.	“Yeah, so that’s why I didn't look through the other side, the shared care.”—ID14 “Usual care, can we talk about like something on the pharmacist as well?”—HCP08	To remove undue influence of preconceived dispositions affecting the binary responses to each factor, the exercise was transposed to a questionnaire format, hosted on an external website.
	Comprehensibility	**Round 1**: Significant confusion over questions on patient navigation and favourability of cost-savings.	“Patient navigation… don’t understand.”—ID05 “Again, they may get confused. ‘What is patient navigation again?’ You may have to qualify.”—HCP01	Simplified language for cost-savings and explained the 'care navigation' aspect in more details under newly created section of 'Shared care' for survivors to make associations.
		**Round 2**: Significant confusion over the sample question created after round 1, specifically on its relevance to subsequent questions. Also, BCS reported difficulty in responding to some questions as they lacked concrete experience of shared care.	“So, what’s this supposed to show me? Maybe you should put, prefer usual care, and prefer shared care. Cause now its food in the cafeteria so it’s a bit confusing.”—HCP04	The example question was removed. Acknowledging the hypothetical scenario of shared care, we modified the outcomes of the exercise to ‘I prefer continuing with usual care’ and ‘I am willing to try out shared care’. We transformed this section into a tabular presentation of preference statements where users give a binary response to each attribute, reflecting their slant to either option based on responses.
		**Round 3**: no major concerns raised by survivors, but one HCP was concerned if survivors could interpret the results (i.e., preference indicated by the higher number of statements checked pro-usual vs. pro-shared care).	“I’m not sure if it’s [results interpretation] entirely clear though. Okay. [Be]cause … that may not come across immediately.”—HCP07	A multicriteria decision analysis method was eventually adopted using the external website. For each consideration factor, users could indicate their care preference and whether the factor is important to them. Responses were then scored as ‘pro-usual’ vs. ‘neutral’ vs. ‘pro-shared’. At the end, the site will return a final score (0–100%), with interpretation explained in the DA.
	Format	**Round 1**: Use of sliders on a continuous scale confused participants who suggested for markers as potentially useful guides. There was a general consensus that there should be a 'results' page to help users interpret their eventual preference based on the responses to the listed questions.	“I think its ok to have the ruler to allow people to put on exactly whatever percentage, but you still have the in-between markers to guide them as a frame of mind.”—ID03 “So, unless you want to have a summary page, you want to remind them what they have chosen…”—HCP02	1. Questions on importance of attributes were transformed into Likert scales. 2. Besides asking users to rate the importance of the attributes, responses to each question were mapped to a choice disposition (pro-usual or .pro-shared care) to improve results interpretation. An example question was included. 3. Added a final ‘leaning scale’ for users to indicate their slant to either option after considering the factors.

*BCS* Breast cancer survivors, *DA* Decision aid, *HCP* Health care professional, *ID* Identity code for breast cancer survivor participants

### Navigation and interactivity

While all BCS in round 1 found the DA to be easy to navigate, we acted on qualitative comments of a lack of clear directives on the clickable components of each page by standardizing navigation buttons and adding an example of a clickable button (Additional file 3). However, this added feature confused BCS in round 2, where 2/5 BCS indicated difficulty in navigation. Replacing with a new, animated introductory section to orientate users to the standardized navigation bar in round 3, participants responded favourably where all BCS found it easy to navigate. Adopting participants’ suggestions, we fine-tuned the colour scheme to minimize confusion over clickable text. Following increased animation use, a higher proportion of BCS (4/5) perceived the DA to be interactive in round 3.

### Amount and type of content

We consistently supplemented the DA content based on participants’ information requests across all rounds, including frequency of treatment side effects, cost listing, and psychosocial resources. Notably, requests for information on the usual care model, the value of HCPs, and their specific care roles in the shared care model recurred. We improved text conciseness and optimized the length of DA after 4/5 BCS in round 1 found it ‘too long’. Eventually, all BCS found the final prototype in round 3 ‘just right’.

### Content clarity and presentation

In the first two rounds, participants persistently feedbacked on medical jargon used in describing treatment side effects and confusion over the naming conventions of HCPs (e.g., primary care doctor, community pharmacist). In response, we reviewed the prototype to paraphrase jargon into layman language and created a new section dedicated to describing each type of HCPs a BCS may encounter before elaborating on care options. Furthermore, participants in round 1 highlighted the concept of ‘late and long-term side effects’ as challenging to grasp from text presentation. We then replaced the text with an animated infographic which participants in round 2 found useful and further addressed concerns over animations being too fast by slowing them down for better comprehension. We also incorporated participants’ suggestions for using a cyclic pictorial to depict shared care instead of linear imagery that did not capture the shared communication channels across HCPs as a distinct feature of shared care. Lastly, we utilized a human body image to contextualize the various treatment side effects for improved relatability to BCS. Screenshots reflecting the revisions are found in Additional file 3.

### Information balance

As the shared care model is a relatively new concept, the preliminary prototype provided a disproportionately higher amount of information to explain the new model. Most BCS (3/5) in round 1 found this unbalanced information to favour the shared care option. To reverse this impression, we separated the description of care options into two separate sections with similar headings at comparable lengths. Despite the revision, most BCS (3/5) in round 2 continued to perceive the information presented as unbalanced and favoured shared care. We then reviewed the literature for strategies to improve information balance and adopted a side-by-side presentation with a head-to-head comparison table [39, 40]. This change improved the perceived balance, where all BCS in round 3 perceived the DA as balanced and not favouring either option.

### Preference clarification exercise

Overall, participants affirmed the relevance of consideration factors included in the exercise but clarified phrases such as ‘patient navigation’ and ‘favourable cost-savings’. On its usability, participants in round 1 reported hesitancy in using sliders across continuous scales and wanted a results summary page to reflect a clear stance on their preference. In response, we transformed all questions in the exercise into Likert scales with interim options mapped to a choice disposition (pro-usual or pro-shared) with an introductory example question to improve results interpretation. However, this sample example question confused participants in round 2 as they reported difficulty responding to questions due to a lack of concrete experience of shared care to project their confidence. Acknowledging the hypothetical scenario of the shared care option, we revamped the exercise to a tabular presentation of consideration factors where users would provide a binary response indicative of their slant towards either option. We rephrased the shared care option to ‘I am willing to try out shared care’ to guide users to clarify their readiness for the alternative care option. While no major comprehensibility and usability concerns emerged in round 3, one participant disregarded preference statements on shared care due to preconceived disposition. To remove the undue influence of such preconceptions, we eventually hosted the exercise as a questionnaire on an external website with a scoring mechanism. Each statement addressing a consideration factor will be scored as ‘pro-usual’, ‘neutral’ vs. ‘pro-shared’ and accounted for its perceived importance. The final score will be an arithmetic mean of the responses to all questions and linearly transformed to return a final score ranging from 0 to 100%, with 50% representing neutrality towards either option, < 50% and > 50% indicating preference towards usual and shared care, respectively (Additional file 3).

### Perceived utility of DA for routine use

**Fig. 2:**
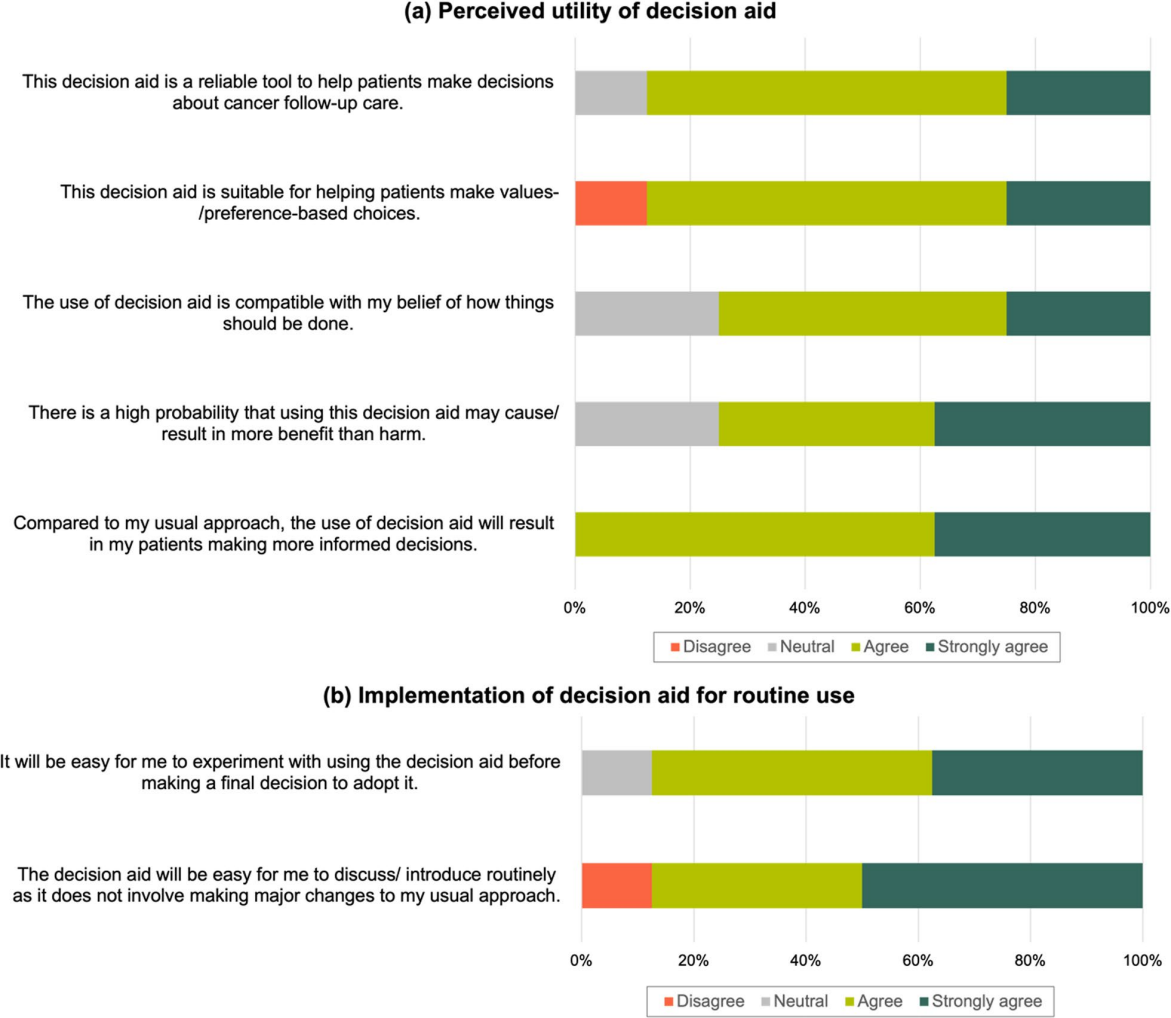
Acceptability of decision aid among health care professionals (N = 8) for **a** perceived utility of decision aid and **b** implementation of decision aid for routine use

Most BCS (60–80%) in each round found the DA useful for decision-making and the preference clarification exercise helpful (Table 3). HCPs shared similar sentiments, with the majority agreeing that the DA is a reliable and suitable tool to help BCS make informed and preference-based choices (Fig. 2). No HCP perceived the use of DA as against their beliefs and would cause more harm than benefits.

All BCS were willing to discuss the DA with their HCPs and recommend it to other survivors in the final round (Table 3). For the preferred mode of distribution, the majority’s preference for digital in the first two rounds (80%) was reversed in the last (20%). Qualitative comments revealed that the paper format would be appropriate for BCS to review in clinic waiting areas (Additional file 4). No consensus was achieved on the suitable timing of DA introduction, with participants indicating preference as early as upon diagnosis to years after active treatment. For HCPs, the majority agreed that it would be easy to experiment with the DA and integrate it into routine care provision without major changes to the existing approach (Fig. 2).

## Discussion

We described the systematic development of a digital DA for BCS considering cancer follow-up care models through three iterative rounds of alpha testing. The final prototype (Additional file 5) was of appropriate length, easy to navigate, balanced, interactive, and usable by prospective users, including BCS and HCPs. This study represents an endeavour to expand DAs usage to guide alternative care model uptake based on care preferences. The development process revealed unique challenges and opportunities for future work.

The decision on survivorship care models differed from the traditional scope of DA application in oncology for procedures such as screening and treatment. Besides the reversibility of the decision, care model selection is inherently complex as it requires BCS to consider their health care setting and context beyond conventional risk and benefits associated with well-defined procedures [3]. The implication of this complexity is two-fold. Foremost, the care model offered as an option should be developed with a well-defined structure, controlling for potential variability in health system factors such as HCPs’ experience and communication style. For instance, we emphasized that all primary HCPs involved in our piloted shared care model received formal training from tertiary HCPs and detailed the continued access to oncologists as a cornerstone of care coordination. The purpose is to ensure the accuracy and clarity of care options presentation to promote informed decision-making. Second, comprehension of concepts related to care delivery may be challenging and vary with pre-existing knowledge or familiarity. This challenge potentially explained participants’ consistent request for more information on usual care and the roles of different participating HCPs across testing rounds. Stirling et al. shared similar sentiments while developing a DA on respite services for patients with dementia in various care settings, as users reported reduced relevance of the DA based on care site accessibility [41]. Furthermore, the perceived value of shared care may be compromised by a generally poor understanding of the ‘care integration’ concept in Singapore, where a study revealed a disproportionate focus on cost and accessibility [42]. Thus, it was unsurprising that participants found phrases such as ‘care coordination’ and ‘navigation’ confusing while consistently acknowledging cost and convenience as important consideration factors. Recognizing this complexity, BCS with lower health literacy may require HCPs’ assistance to clarify concepts related to the care system.

Besides being an implementation barrier for alternative care models, low confidence in primary care HCPs among BCS also poses a recurring challenge in the design of preference clarification exercises [5, 26]. In the initial testing round, participants consistently emphasized that their low confidence in primary care to manage cancer-related issues was the major deterrent from shared care, limiting the utility of the exercise in influencing their choice disposition. In contrast, BCS perceived the highlighted strengths of primary care in health promotion and comorbidity management as relatively less important, possibly due to inadequate community health-promoting practices [43]. Correspondingly, the team focused on strategies to counter these inherent biases towards the primary care HCPs involved in shared care without disrupting information balance. Acknowledging BCS’ uncertainty, we reframed the exercise to explore BCS’ openness towards trying shared care, minimizing potential cognitive dissonance between the exercise results and choice predisposition. After experimenting with different preference clarification methods each round, the multicriteria decision analysis was employed in the final prototype, consistent with recommendations from the latest meta-analysis [44]. This method probes users to consider and weigh factors besides the sole confidence factor.

While the primary aim of this study was not to evaluate DA implementation, preliminary results revealed that both BCS and HCPs perceived the utility of the DA favourably, with no major challenges anticipated with integrating DA introduction into existing workflows. While agreeable to the usability of the DA, the preferred distribution mode and the timing of introduction were heterogeneous. While BCS generally preferred the digital format in earlier rounds, a more neutral stance in the last round could be due to the older age of the participants. The availability of print materials in a formal cancer centre setting would be compatible with the information-seeking behaviour of elderly Chinese women in Singapore [45]. However, a significant drawback of the print version would be the potential loss of interactivity and animations embedded in the current prototype. Besides providing a print version as an alternative to boost access to the older group, BCS’ interpersonal networks could be tapped to improve uptake of the digital format through recommendations by HCPs and fellow survivors [45]. Our results exemplified this strategy’s feasibility as most BCS respondents were willing to recommend the DA to others, potentially through support groups.

The main limitation of this study was the underrepresentation of low literacy groups, as all survivors minimally held a secondary school degree. Nevertheless, we also drew strength from our user-centred design approach [46], where the sample frame included prospective DA users. We purposefully sampled HCPs from disciplines that routinely encounter BCS, representing windows of opportunities for DA introduction and usage. Additionally, we managed to sample BCS with varied characteristics to capture diverse user experiences. These characteristics included an age range that coincided with the highest breast cancer incidence rates and a stage distribution that mirrored the Singapore Cancer Registry data [47]. This codesign engagement is crucial in avoiding the trend of suboptimal adoption rates reported by DA developers [48]. Furthermore, we plan to nest the developed DA formally within the shared care model to be used by prospective BCS in the upcoming scaling up phase. This formal inclusion as a packaged intervention could be an implementation strategy to promote sustained uptake. On generalizability, the DA developed provides a structural foundation for information adaptation to portray the primary care-led model as an alternative option. Notably, both scenarios necessitate a basic understanding of the health system. Lastly, future work should explore employing AI in DAs to enhance and personalize information delivery, language complexity, and quantity of information based on predefined characteristics such as age and familiarity with the primary care system.

## Conclusion

We systematically developed an acceptable DA for BCS considering the usual or shared care model piloted in an ongoing trial. Our experience highlighted an additional need to provide contextual information on the health care system when addressing decisions related to care delivery. Importantly, prospective developers for similar decisions should actively address variability in health system factors (e.g., HCPs’ training) to describe care models accurately while consciously identifying and clarifying inherent biases, such as low confidence levels in primary care observed in our study. The DA is now ready for integration into the shared care model to be scaled up and field-tested. Future work could explore adapting the informational structure to care models with different degrees of primary care involvement and leveraging AI to optimize information delivery (Additional file 5).

## Supplementary Information

**Additional file 1.** Overview of contents in the first decision aid prototype.

**Additional file 2.** Interviewer guide for semi-structured interviews.

**Additional file 3.** Screenshots of revisions made to the decision aid prototype across alpha testing rounds.

**Additional file 4.** Qualitative comments on the perceived utility of decision aid, implementation for routine use, and format of decision aid.

**Additional file 5.** Transcripts and the final decision aid prototype.

## Data Availability

All data generated or analysed during this study are included in this published article (and its supplementary information files).

## Abbreviations

BCS: Breast cancer survivors
DA: Decision aid
HCP: Health care professionals
ID: Identity code for breast cancer survivor participants
