# Supplementary material for: Development and pilot testing of a decision aid for navigating breast cancer survivorship care
Source: BMC Med Inform Decis Mak. 2022 Dec 15;22:330. doi: 10.1186/s12911-022-02056-5 (PMC9753367; doi:10.1186/s12911-022-02056-5)
Supplement: Supplementary file 1 — Additional file 1. Overview of contents in the first decision aid prototype. [file 12911_2022_2056_MOESM1_ESM.docx]

**Additional file 1** Overview of contents in the first decision aid prototype.

| **Section** | **Description** |
| --- | --- |
| Overview of decision aid | - Description of target audience - Instructions on how to use the decision aid - Highlighted the 5 key steps/ sections |
| Step 1: Introduction to cancer survivorship | Addressed the following content areas:   - What is cancer survivorship? - Why is cancer survivorship important? - Cancer prevention and surveillance - Long-term and late side effects of treatments - Emotional distress |
| Step 2: Understanding your options for cancer survivorship care follow-up | - Explanation of usual care (oncologist-centric model)   - Table summarizing key follow-up events (e.g., mammogram, bone mineral density tests, examinations) - Explanation of shared care:   - Table explaining the different roles and responsibilities of each participating health care professional   - Mechanisms of care coordination between health care professionals - Table comparing the usual care and shared care options |
| Step 3: Understanding your care preferences | Users were asked to indicate their responses to the following questions on a continuous slider anchored at the two extreme ranges (e.g., least to most important):   1. Do you have a regular doctor taking care of your non-cancer chronic conditions? 2. How important is the type of healthcare provider providing cancer survivorship care? 3. How comfortable are you with trained primary care doctors co-managing your cancer survivorship issues? 4. How important is the cost of appointment(s)? 5. In your opinion, how favourable are the cost-savings associated with an appointment in polyclinics rather than at the cancer centre for cancer survivorship care? 6. How important is the location / convenience factor? 7. How important is the ease of making clinic appointments? 8. How important is it to have patient navigation? 9. Would you like a pharmacist in a community store near you to guide you in your cancer survivorship journey and to answer your health-related queries? 10. How important is it to have care coordination? 11. Would you like your oncologist and primary care doctor to communicate with each other about your cancer survivorship care? |
| Step 4: Pilot trial of shared care option | A video explaining the study procedures of the ongoing pilot trialling shared care model (NCT04660188). |
| Step 5: Additional resources | Hyperlinks were provided to other online informational resources |
