# Supplementary material for: Development and pilot testing of a decision aid for navigating breast cancer survivorship care
Source: BMC Med Inform Decis Mak. 2022 Dec 15;22:330. doi: 10.1186/s12911-022-02056-5 (PMC9753367; doi:10.1186/s12911-022-02056-5)
Supplement: Supplementary file 2 — Additional file 2. Interviewer guide for semi-structured interviews. [file 12911_2022_2056_MOESM2_ESM.docx]

**Additional file 2** Interviewer guide for semi-structured interviews.

| **Theme** | **Sub-themes** | **Questions** |
| --- | --- | --- |
| Comprehensibility of each page/ section | Content | - What do you think the main message was? - What is new or different about the information presented here from what you already know? - What concerns do you think other patients would have that the decision aid (i) have and (ii) have not covered? - What other information or resources would you like to have for you to make an informed decision? - Which part of the content do you find to be redundant or not helpful in the decision-making process? |
|  | Language | - What have you read here that other patients/ survivors might find confusing? - What are the medical jargons that you think would require additional explanations? |
|  | Preference clarification exercise | - How did the exercise help you to better understand your preferences in follow-up care? - In what ways do you think the values/ preferences exercise will be beneficial or helpful in decision-making? |
| Format of decision aid | Appearance | - How did you find the decision aid aesthetically? You may comment on any appearance features including the colour schemes, choice of font, font size, etc. - How do you find the use of icons, images, graphics and interactive buttons? |
|  | Navigation | - How did you find the navigation buttons on the pages? In what ways do you think that they have been helpful in your viewing experience? - How did you find the progress bar at the top of each page to document your progress? - How would you suggest the developer team to further optimize the decision aid format or interface? Are there any special functions you would like? |
| Feasibility of implementation | Practical utility | - What do you think about the length and time taken to go through the decision aid? - How did you find the function of a downloadable copy of the decision aid for viewing or on your electronic device? - Will you revisit the decision aid for some of the information presented? |
|  | Routine use in clinical settings | - If given a chance, would you have used this decision aid to discuss follow-up care with your oncologists? - What feasibility aspects of the decision aid should be considered or improved before it can be rolled out to other patients? |
| Summary feedback |  | - What other thoughts came to mind while you were viewing the decision aid that you haven’t shared? |
