# Supplementary material for: Development and pilot testing of a decision aid for navigating breast cancer survivorship care
Source: BMC Med Inform Decis Mak. 2022 Dec 15;22:330. doi: 10.1186/s12911-022-02056-5 (PMC9753367; doi:10.1186/s12911-022-02056-5)
Supplement: Supplementary file 3 — Additional file 3. Screenshots of revisions made to the decision aid prototype across alpha testing rounds. [file 12911_2022_2056_MOESM3_ESM.docx]

**Additional file 3** Screenshots of revisions made to the decision aid prototype across alpha testing rounds.

**
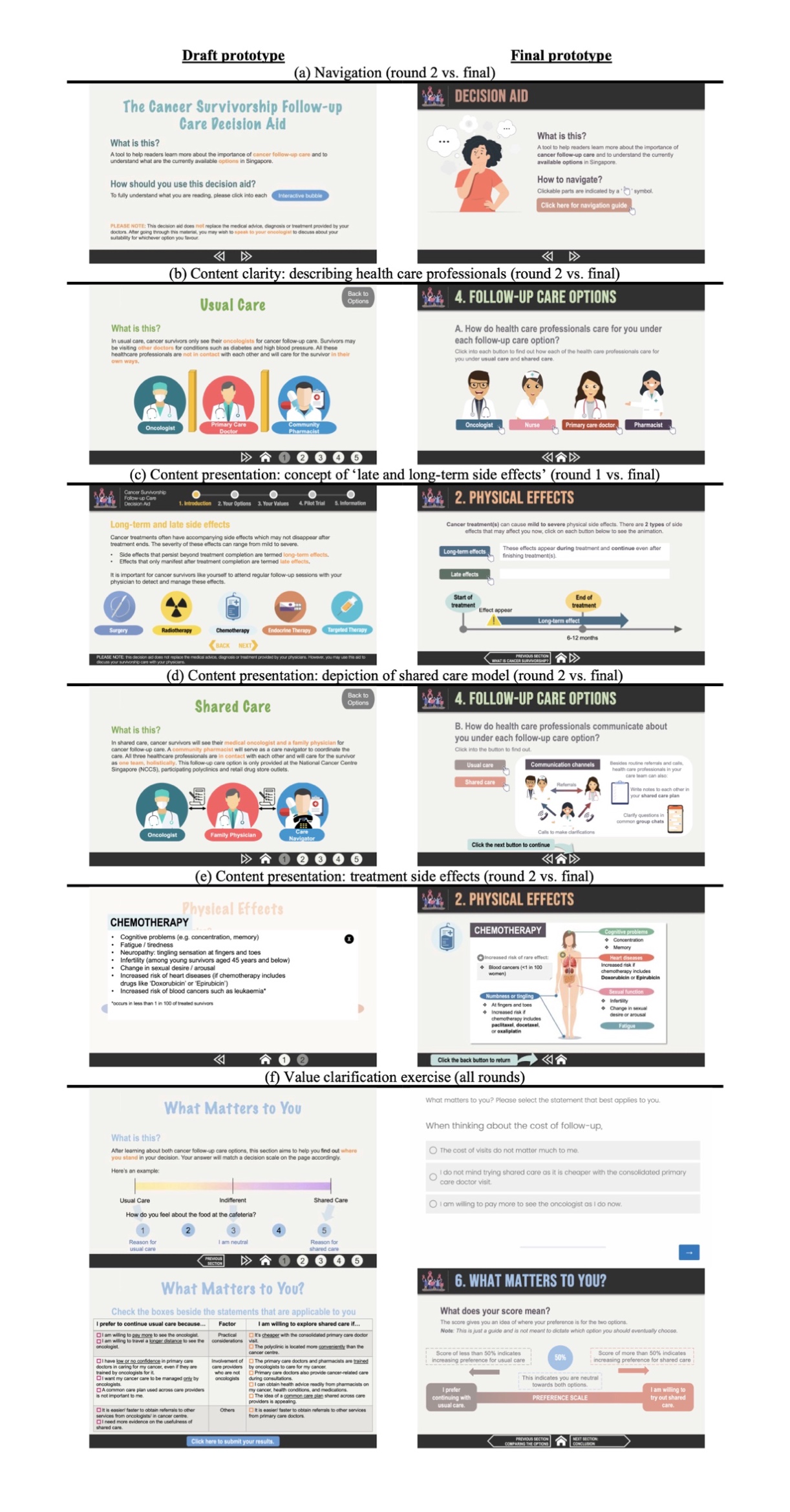
**
