# Supplementary material for: Development and pilot testing of a decision aid for navigating breast cancer survivorship care
Source: BMC Med Inform Decis Mak. 2022 Dec 15;22:330. doi: 10.1186/s12911-022-02056-5 (PMC9753367; doi:10.1186/s12911-022-02056-5)
Supplement: Supplementary file 4 — Additional file 4. Qualitative comments on the perceived utility of decision aid, implementation for routine use, and format of decision aid. [file 12911_2022_2056_MOESM4_ESM.docx]

**Additional file 4** Qualitative comments on the perceived utility of decision aid, implementation for routine use, and format of decision aid.

| **Theme** | **Round 1 (n = 8)** | **Round 2 (n = 8)** | **Round 3 (n = 7)** |
| --- | --- | --- | --- |
| Utility in decision-making | The first prototype was perceived to be useful as an information source but not for decision-making.  “*Bearing in mind that this is a decision aid, so after I read, I’m not sure how it… it doesn’t aid me to make a quick mental note of the decision. But I think it is good to .. the content is there and its good for the patient to learn about these things*.” – ID03 | The second draft was found to be useful in helping survivors understand available care options. Particularly, the additional online resources were favourably perceived.  “*To help me understand that with more health workers involved, yeah, there will be better assurance, yeah, better assurance for the patient. And I think most important is being able to see the family physician periodically will also have better assurance*.” – ID09  Health care professionals further perceived the DA as a survivor empowerment tool.  “*I think that it is actually, like the patients will feel engaged, like a bit empowered when they take this. And if I were the patient, I will be like, ‘oh, I am trying, I am being considered in shaping what I would want on the kind of care services’, so I would feel good*.” – HCP06 | Survivors perceived the third draft to be useful in preparing for what is expected under alternative care option, thereby assisting decision-making and providing a sense of ownership of final care decision.  “*So I think maybe this is a first start for example so do you want it and I think you give people a choice rather than you push it down to them. Because when you push it down to them, people will tend to complain, they will not like it*.” – ID11  One survivor further alluded to the potential long-term utility of the DA, whereby survivors may revisit the DA at a later time point in the event that they wish to switch follow-up care later in survivorship.  “*So to say at the very beginning. I think about, ok, I still wanted to go for the usual care plan. Then, after some years like me, if I'm going to retire, then I don’t have any further income, I will go to look into the share care, which is at a lower cost, more convenient. So maybe information to advise the plan that we can shift or not. If not shift, we can switch to another plan*.” – ID13 |
| Routine use of DA | Limited comments were obtained but one HCP suggested having a printed/ hardcopy format of the decision aid.  “*I think it will be good if they can print the result if they want to.*” – HCP03 | Among HCPs, there was a general consensus that it will be easy to integrate the decision aid into routine care by leveraging existing survivorship clinics which the target audience of the decision aid routinely attend.  “*I think it shouldn’t be difficult because you have a specialised subsidised, not subsidised, but a specialised survivor clinic, and all those patients are five years out, right?*” – HCP06 | Survivors stressed that the usage of DA should be discussed with the oncologists before making a final decision.  “*Get the consent from the patients and then another thing also, let the doctor choose the patients. That’s what I feel that because sometimes doctors and the patients don’t have the chemistry, no point forcing them*.” – ID11  Similar to round 2, HCPs did not raise major concerns for using the decision aid in clinical setting as it was found to be straightforward. While HCPs echoed survivors’ comments that a HCP should be involved in the DA usage, one HCP suggested other personnel besides just oncologist, including nurses and coordinators.  “*Actually, I think some of the things are quite straightforward, I mean the information is already inside, so whoever who can navigate through. Don’t really have to be nurses, I feel. Yah, I think even research coordinators are okay*.” – ID08 |
| DA format | No major comments captured. | Both survivors and HCPs requested for a paper format to complement the existing digital version. HCPs further highlighted the need to include more language versions to be inclusive of the multicultural population in Singapore.  “*Currently we only have the English version. Maybe in future, we might translate to other because we also understand that not everyone can read English*.” – HCP05 | Similar to round 2, survivors also requested for more language versions and a paper format of the DA. Survivors substantiated that the availability of the DA in hardcopy formats in clinic waiting areas would be compatible with their typical information-seeking behaviour, making better use of the waiting time. A HCP also shared this same sentiment and emphasized that multiple formats could provide adequate avenues for improved access.  “*Because like for example, if I’m sitting in waiting room, [be]cause sometimes you have to wait a long time. If you have those posters [hardcopy formats], if you have nothing to do, you can look at them and you might learn a few things from that. Or, if I’m actually looking for the information, if it’s… there are pamphlets available, then, I can just get them*.” – ID13  “*I mean if we want a higher uptake I think, because I mean I feel some patients would probably like that to have something to do during the wait, but some patients will be too worried to do anything. So, if we can have a few like both of the avenues, then maybe it’ll be better*. " – HCP08 |
| Abbreviations: DA, decision aid; HCP, health care professional; ID, identity code for breast cancer survivor participants | | | |
