# Supplementary material for: Development and pilot testing of a decision aid for navigating breast cancer survivorship care
Source: BMC Med Inform Decis Mak. 2022 Dec 15;22:330. doi: 10.1186/s12911-022-02056-5 (PMC9753367; doi:10.1186/s12911-022-02056-5)
Supplement: Supplementary file 5 — Additional file 5. Transcripts and the final decision aid prototype. [file 12911_2022_2056_MOESM5_ESM.zip › Additional file 5/HCP03 - Transcript.docx]

**Study ID: HCP03 Date: 26/11/19**

**Interviewer(s): ET & KY**

HCP: is there a Chinese version? And the patients may be old right?

HCP: I think “in this decision aid, you will learn more about”.. maybe like “this decision aid will help you learn more”

KY: “while using this decision aid?”

HCP: something like that. “in this decision aid” is a bit weird. I guess the top is ok, just a bit worried the font, some people cannot be but should be ok I guess…

HCP: is there a back button? There’s no ‘back’ button ah? (intro to DA before starting)

HCP: so it will be like a clinical trial la.

KY: ya, initially we wanted to put in the introduction of the trial into this DA but ultimately, for the trial purposes, we will be taking out certain parts of the DA. Cause we want to develop this, eventually it will not be for patients who are just going to be recruited for the trial. The target population at the end will be more broad -explains more-

HCP: Some people they see ‘trial’ then they think “am I a guinea pig?”

HCP: I think start**s** right? (typo)

HCP: is there an end date for the survivorship? Some people might ask “is it after 5years, the cancer won’t recur?” I don’t know… the ‘extends beyond your treatment’ part, is there an end date or for the rest of your life? [KY: yes]

HCP: i’m just a bit worried that some of the slides are a bit wordy so it takes a while to read and for patients who are not so literate…

HCP: im not sure. Do patients understand ‘surveillance?”

KY: what would be better? Continue to monitor? Regular checks?

HCP: yea, something like that, like “continuing monitoring”. Surveillance is, I mean we know, so sometimes when I read, I’m not the patient so I cannot really think like whether they can… but that’s one term.

HCP: is “second cancer” a term? Or is it “new” or “secondary” cancers? [ET: second cancer used in our reference] recurrence, do they know..? oh it’s explained. How about the recurrence? Don’t have in the picture, only new cancer cancers. Here is recurrence then there is relapse, will they be confused? [ok so which one is more intuitive/familiar to patients?] I think both are ok, you can put one or put both, just be consistent. So they have to click on the bubbles?

HCP: I;m not sure about the click on the bubbles to find out more… [KY: you mean make it more obvious that they can click on those things?] [ET: do you think if having the bubbles change colour when the mouse hovers over would help?] maybe? Because if I look at it I would think it’s just a picture

HCP: but after mastectomy then no need RT right? It’s only for conservation… maybe you don’t have to put mastectomy, just surgery… this risk is… the risk factor, what does it mean? “the increase risk is first seen 10 years after radiation” means that in the first 10 years you will not get lung cancer? Only after 10 years then the risk for lung cancer increases? I don’t know what this is supposed to mean. [KY: ok to refine ☺]

HCP: Actually, patients who have one side mastectomy still can recur on the other side. Maybe don’t need so much details. I think “there may be”, for the ovarian cancer, “there may be increased risk”, don’t sound so definite, don’t scare them too much.

HCP: im not sure for the tamoxifen, do you want to write something like “regular check-up is important”?

HCP: for this, the patient will read it on their own or somebody will go through with them? And then they have qns they can ask? [KY: -explains-] ok I’m not sure whether our leaflets write the risk of like secondary ovarian cancer. Some people may ask what’s the risk. I’m not sure whether or not it’s a good idea to include but you can consider. Small like how small? 1 in 10000 or 1 in 1000000.

HCP: “manifest”? like “appear” la. Maybe instead of “term” can say “called”, more intuitive. This one cannot click? [ET: can] but nobody knows can click… prompt or change colour or something.

HCP: numbness, sometimes pain also, I think sometimes there’s pain and pulling sensations. This is for breast cancer patients only right? So I don’t think sexual function issues from surgery…? [KY: ok will refine] I think it’s a bit weird… like just cut out then…??? Maybe joint stiff, pain and numbness at surgery site, sometimes you can put it together or something, or pulling feeling… weakness? You mean? I don’t know, does it cause weakness? This is just mastect and bcs(?) right? Other things for surgery… lymphedema oh late effects… I don’t think they get late lymphedema though…

HCP: radiotherapy fatigue… ok the sexual function issues also a bit weird. I’m not sure how long after the RT will the skin discoloration take to recover… [KY: can lump it with the sensitivity and pain] ok should be ok. Never talk about secondary cancer..? [KY: cause the previous slide already said so do you think we should combine?] Because RT really cause.. it’s one of the major late effects if the… secondary cancers. Fertility issues, I don’t think it will be an issue cause it’s just the breast.

HCP: typo. Fatigue, most people should understand. [KY: maybe like tiredness]. Ya, because some words we use it very commonly and we don’t really think about it. Or neuropathy also... but you did explain what it means.

HCP: many menopausal symptoms? Like the secondary cancer also, because it’s in front, the endometrial cancer. [KY: I think it’s better to put it here cause another doctor also say..] then I think.. I don’t know how you going to fit it in because if im the patient I would be thinking, how can I monitor what should i to do? So they need to actually monitor for the PB blade(?) and regular gynae follow up at least annually when they are on tamoxifen. Will they ask you what is AI? KY: yes, maybe split up then they just click into the drug they are taking cause others may not be applicable] then maybe for side effects, you can list the order, like the more common ones or more important ones will be on top. I think more common ones will be on top like according to frequency or something. It is true, the AI if you have 2 then you don’t have to repeat this twice. Then for osteoporosis, normally doctors will give Calcium and you should be compliant to it…

HCP: bankruptcy haha but it’s financially toxic. Pertuzumab? Don’t have pertuzumab. Maybe trastuzumab bracket Herceptin then pertuzumab bracket… I mean if you want la [KY: do they know of it as targeted therapy? Like when patients are introduces] Sometimes they just lump it together with chemo. Like maybe, the endocrine should be last because targeted and chemo will be together. Having said that, maybe your surgery, chemo, targeted, radio then endocrine. I don’t know whether it’s a good idea to follow [KY: so it follows their typical treatment?] Ya, at least I feel the targeted and chemo should be together, endocrine should be last.

HCP: ok, so this is…. Maybe like “situation feel tougher than usual”, I don’t know whether you can rephrase this into something that is nicer? [KY: challenging?] ya, maybe.

HCP: so this is physical then after that its emotional? Cause I felt that it was suddenly out of nowhere… ok

HCP: this is only for NCC is it? Then maybe “at NCCS” (specify according to local context). why is shared care behind. I was thinking it can be two, then what is usual care and what is shared care.

HCP: I think this one can rephrase ”survivors taking tamoxifen with uterus”… (like eating the uterus)

KY: this one do you think it’s routinely done?

HCP: yes they do, they have gynae but its not done under a medical oncologist. They will refer to a gynae to do it.

HCP: surveillance the mammogram… here we do once a year but im not sure if there are some people they do it once every two years. Cause one of the guidelines, for certain age group they do it once every two years. [KY: for older] ya correct [KY: so specify?] ya. Im not sure what’s the practice for all.

HCP: im not sure whether “like you / yourself” is a good idea? [KY: so distance it first…like perspective first] depends on what the patients prefer. Any patient feedback? [KY: not really… maybe like they don’t want to be associated with shared care yet?] ya, then cancer survivor… then, I don’t know, some people.. but here don’t have cancer survivor. Maybe “to monitor for cancer recurrence, or new/cancer recurrence”... Actually, the mammogram can only be done here? [KY: polyclinic the mammogram they can do for screening but the surveillance one is very expensive. It’s the naming, while it’s the same procedure, they will charge more for surveillance] then actually if im the patient I will just come back here since im coming back if im doing a yearly surveillance eventually…

HCP: “comorbidities”? I think “other health conditions” or “chronic conditions”. Ya sometimes the terms to use is very… maybe some I never really catch also.

HCP: pharmacist navigator? [KY: -explains-] it’s like a call centre job… ‘side effects’ instead of ‘toxicities’ and comorbidities too. Maybe not ‘patient’. They are not patients already [KY: ‘survivors’] ya…

HCP: maybe these three can be the same width, like the poor PCP is squashed (table format for roles)

HCP: same thing, not really patient anymore… ok.

HCP: thing like ‘development’ and ‘updating’, maybe the same tense. Either ‘developing’ or… so its like ‘development of’ and ‘update of’. ‘updating’ then ‘developing’

HCP: maybe the ‘screening’ instead of ‘screens’

HCP: so the high risk patients will be under usual care? Maybe for this part can be both? Like the first column for both… mortality I don’t know whether people understand…

HCP: must go to the link to see the charge? Not directly? These are all… the subsidized rates? As in after the subsidy? Are there any other schemes? I don’t think there is… pioneer, medeka…. Does it include GST? Haha…. Some people are quite particular about the price. Not sure if it’ll be better if its centralized (table layout for cost) [ET: but it’s from the original website ☹] This link is the same? Even if they go for survivorship visits, the cost is the same? [KY: -explains fam med clinic-] so its only the family medicine? Then I think it’s good to write here, family medicine doctor or something so they will know that they cannot just walk in to see anyone, if not they may have the wrong impression. Instead of having a link, not sure if it’s good to just put it here (cost)… instead of having a lot of links.

HCP: so if they’re private here, then they go polyclinic, will they be private or subsidized? But actually all polyclinics are subsidized right… ok nevermind you all can figure it out.

HCP: “will it inconvenience you?” yes. I don’t know how to phrase it in the other way? So that they won’t think ‘will it inconvenience you. So you’re giving the impression that its inconvenient... maybe I don’t know how to phrase it in a positive sense, ‘which is more convenient for you?”…

HCP: im not sure the ncc appointment, is it easy to get or what. [ET: -explain that patients feedback that it’s easy / fixed appts-] actually it’s quite true.

HCP: I was thinking if there is a tab, like this kind of bullets for every (row in the table)... ya then to make it more standardized. I don’t know whether it will waste space but you can always make the margin smaller.

HCP: this one is a bit weird right? I don’t know.. .why.. I think this is not very necessary.. I mean it’s a bit weird… maybe some of them done like to see the oncologist, some of them like to see the oncologist maybe? (last row, the onco part under usual care)

ET: so for this table, do you find any of the information, e.g. this one, redundant that we can cut down on to make the material more concise?

HCP: may this one, the cost, the finances one can be together, and this one the funding and the participation in the model. And the follow up schedule and the convenience thing is kind of together.. this one also can kind of combine with the.. maybe somewhere here; who is suitable, how well does it work.. then the follow up schedule and convenience and are they communicating, then the funding. I think it flows better. Then got one more, what else should you consider. This is like the advantages right?

HCP: actually the comorbidities the oncologist won’t manage at all.. they will just throw them back to wherever they came from, they won’t manage the comorbidities if they are on surveillance.

HCP: maybe like the convenience thing [KY: bring it back to that?] ya because the location and the polyclinics.. so it’s like together. Then the finance thing is at the back.

HCP: Maybe “you will prefer”? or this part can rephrase, “the following qns will help you understand your follow up care preferences to help you determine which of the two care models you may prefer” something like that… they have to… oh cannot click.

HCP: like “non-cancer chronic conditions”, this is better than ‘comorbidities’… “do you have a close relationship with this doctor?” ok… it’s a bit funny but… ok. ”How important is the”… but what’s the point of this [KY: slider thing?] they will.. so least important, most important? [KY: -explain purpose of the section-] so most important, if they choose here, they will feel that they want the oncologist la, then least important is the polyclinic doctor? [KY: so if I choose most important here, Ok so for this qns, if it’s most important then they only want the oncologist but if they are on the other side, it opens more room for this idea that some other doctor is also caring for your cancer stuff…] cause I think it’s not very intuitive to [KY: indicate that] ya….

HCP: in both ‘settings’

HCP: but yes no is like… [KY: dichotomy is it?] kind of [KY: so I think some of the others also feedback that they’re not sure about the important thing. So is it better if we just let the patients rank the factors of choice. So there may be a certain factor that is very important to them, for example the type of healthcare provider, if that’s the most important decision factor and if they do prefer seeing only the oncologist, then definitely, shared-care may not be as appealing to this group.] but in other countries or studies, what do they use / do [KY: ranking is also one, there’s also this kind where how important then they give you 5 grades like Likert scale] cause I find it’s actually quite long, the whole thing, you have to take some time [KY: I guess certain key factor like the type of provider will be very strongly 1 as well as the comfortable with primary care. So what some of our patient says is for e.g. convenience and cost are actually not very important to them but when they answer these kind of qns, it actually seems as though polyclinic is better because they stay near a polyclinic but even if they stay near a polyclinic, they don’t really care, they don’t want to see the polyclinic doctors. So this one quantifies the weight of this answer] I think by having a lot of questions, you can get a lot of information but I don’t know if the information will be important like what you mentioned. Maybe they don’t really care about the location or convenience. But I guess you can just get more information when you ask more questions.

KY: are there any factors that you think patients should consider or would think of when they want to make this kind of follow-up care decisions?

HCP: how about the oncologist? [KY: like whether my oncologist actually talk to me about it?] as in, (onco) is accepting about this. Let’s say you want them to go to the… because I’m not sure which patients will be administered this, but let’s say you administered this to a patient and in the end the oncologist says “no, I want you to see me” then might as well don’t look at this. So you need to check with the oncologists before you give it to their patient. Sometimes, also, some people have a lot of appointments in SGH, so they don’t mind just coming here. I’m not sure how the end product will be but maybe instead of asking them to use a pen and then odd and even question themselves, I think it’s very hard to tabulate. And then what is the answer? I just want to know the answer. Like when you do those online surveys and stuff, your just want to know the result, you don’t really care about what you filled in. Like “polyclinic is for you!” I don’t know. So instead of this, you can show them the appropriate slide for them.

HCP: Maybe ‘suboptimal’ can change to another word?...

HCP: the video cannot change one right? [ET: CAN] wait, there was one… should be ok.. like ‘follow up medical care’ is like…. What are they doing there? They’re going to assess for symptoms? [KY: mainly ask about acute symptoms or anything out of the norm. or how has the previous encounter with the doctors been] ok, should be ok… is the trial called pilot trial or BASIC? [KY: BASIC but it is a pilot trial ☺]

HCP: then the pharmacist is free la [KY: -clarify payment of pharmacists in study-]

HCP: SCS uh… I don’t know SCS website.. there are some programs which are a bit weird, like they don’t really exist kind… ‘return to work’ doesn’t exist…

KY: so what some of our participants have said is that in this case this one is good like we tell them a purposeful thing to look out for (breast examination) but for the first two, its very generic. Maybe we can give them a bit more information by pointing them to the existing things like support groups

HCP: I think instead of SCS, we have the patient support group, so we can go to the website if there is or the programmes, then they can go. Then more information maybe… I don’t know ‘even more information’… maybe for…. Like what the website says, ‘how to cope with treatment’ or whatever then you change it. Whatever the website is saying… like what is there. Then the order, you all can decide which is more important. Maybe the cancer terms.. instead of cancer terms, ‘what do the terms mean’

KY: actually you can click on top, did you read just now? (navigation bar)

HCP: cannot go forward only can go back… is there a way like once you already go before, then you click back, you can go forward?

HCP: “not sure what some of the terms mean?” It’s not a cancer term.

HCP: ya I think it will be good if they can print the result if they want to.
