# Supplementary material for: Development and pilot testing of a decision aid for navigating breast cancer survivorship care
Source: BMC Med Inform Decis Mak. 2022 Dec 15;22:330. doi: 10.1186/s12911-022-02056-5 (PMC9753367; doi:10.1186/s12911-022-02056-5)
Supplement: Supplementary file 5 — Additional file 5. Transcripts and the final decision aid prototype. [file 12911_2022_2056_MOESM5_ESM.zip › Additional file 5/ID05 - Transcript.docx]

**Study ID: ID05 Date: 15/11/19**

**Interviewer(s): ET & KY**

**PART 1**

ET: so in-between as you’re reading through, we will do this questionnaire so that its easier for you to refer back to what you just read. So for the first two pages you’ve just read, its regarding survivorship, like cancer survivorship, so we just want to get a feel of whether or not you understand it and whether or not the content was too much, and how was the design?

ID: ok…

ET: Regarding the amount of content, do you feel that its just right or it’s a bit too brief like, need more information?

ID: you’re gonna talk about it more at the back right?

ET: no, its just like that, the cancer survivorship

ID: ok… this is probably for people who’ve just been diagnosed right?

ET: for people in their survivorship phase, so after their primary treatment and they are doing their follow-up with the oncologist

ID: mmm ok.

ET: So for things like that, do you understand what is the “AI” and the other drugs? Like this part?

ID: err.. I do not know what AI means

ET: -explains- so do you feel that we should just put the drug name so that survivors can identify with it?

ID: ya, I suppose

ET: ok ☺

ID: cause I don’t take this..(AIs) I mean I only took this (tamoxifen) much much later

ID: the purpose is that you’re trying to cover everyone (BCS) right?

ET: ya

ID: ok, a lot of info la (end of section 1)

ET: ok, so having read through this section, just now it talks about late and long term effects, do you understand what is the difference between the two? Based on this description

ID: well, you already state it here right? Like this is what you probably will get for a long time, oh no no sorry, this is like you will probably get way late… eh, no. long term effects… and late effects.. that means this one (long-term) you will always have it, like you will always feel fatigue.. forever. This one is like maybe much later after treatment you will have this one. Is that what it means? (she interpreted from side effect slide rather than from definition words)

ET: yes, that’s right. Ok. So this explanation here helps you understand it better? Is this clear enough?

ID: errrmmmm ya…. Mmm ya… ok

ET: -questionnaire-

ID: I guess some will be like… (inaudible) (content amount), ok (understanding), feels very wordy (layout)

ID: usual care and shared-care… what is usual care? -continues reading-

ET: ok so usual care is supposed to be describing what you’re going through now. do you feel that it accurately depicts your situation?

ID: ya..

ET: so for shared-care, im not going to tell you what it is, you read through and see if you understand.

ID: -after first slide- so this one is 3 lots of people will be coming to you right? But who is the general care, this person?

ET: you don’t know who is playing this role (PCP) is it?

ID: primary care.. -reads- ok.. this person (PCP) is the main person? And anything serious you pass on to this person (oncologist).. and then meanwhile, if you have any medical thing you ask this person (pharm) but not this person (PCP)?

ET: so for the medical thing you can ask any of them, just that the pharmacist will be readily available for you to ask.

ID: so lets say if I need help then who do I talk to? This person (pharm)?

ET: you can talk to this person(PCP) during your consultation and if you have no consultation, you can have a contact directly to your pharmacist

ID: alright, lets say I took some medication and I feel giddy?

ET: you can talk to this person (pharm)

**PART 2**

ID: so who am I supposed to contact?

ET: so basically you contact this person (pharm) as your primary point and any issues..

ID: oh this is the primary point?

ET: he’s the pharmacist navigator that will be located near you, so basically local pharmacist like guardian, watsons all these. Then when you contact this person (pharm), any issues that he deems is severe, he will raise it back to either your primary care doctor or your oncologist.

ID: what does comorbidities mean?

ET: comorbidities? It’s like conditions like diabetes, high blood pressure, high cholesterol… do you feel that if we used the term “chronic diseases”, you will understand better?

ID: mmm (agree)…. What is PCP?

ET: the primary care physicians

ID: which is the pharmacist.

ET: er no, the primary care doctors, so actually these people are like GPs and polyclinic doctors.

ET: So after this table, do you feel that it helps you understand the roles of each healthcare professional?

ID: no really… hahahaha

ET: so its not very like… (helpful), what do you think may be more helpful in helping you understand what are they (HCPs) doing for you?

ID: like, for example, this one, accessing the need and scan… ok -starts reading each item- help promotion… don’t know what this means..

ET: so basically its like promoting healthy living, lets say you’re a regular drinker or smoker, they will tell you to stop doing that and drink less, or eat more healthy fruits and vegetables these sort of things

ID: development of survivorship care plan… what is HCPs and SCNs… supportive care nurse

ET: healthcare professionals.. so does this asterisk induce some confusion?

ID: mm(Agree), development of survivorship care plan….

ET: ya, so this is the definition of the survivorship care plan that you’ve been reading. So before this page, do you know what a survivorship care plan is? [ID: shakes head] but after reading it, can you tell me what a survivorship care plan is?

ID: I still don’t really know la..

ET: so its not very clear here?

ID: ya

ET: basically it’s the plan or rather the information that will be shared amongst the three healthcare provider regarding your care, so its like a care plan for you as a survivor that will be going through this programme, so it will contain the information that was shown in the slide, like all these information.

ID: this, for this it means you’re at very low risk of getting cancer, for this is for people with a lot of other problems.

ET: ya, like people who still have to be closely monitored

ID: so this is cheaper because you only see the pharmacists…

ET: the polyclinic doctor, so instead of your consultation fee for the oncologist each time, like it is shared between the oncologist and PCP. So lets say in a year, if you visit the oncologist twice, then you have to pay the oncologist fee 2 times. But in the shared care, you visit the oncologist one time then the second time will be at the polyclinic, so you save on that one consult.

ID: but will that really… because you’re seeing so many different people.. at least (at nccs) you’re seeing the same doctor, the doctor knows your history, you don’t have to keep repeating to every single person you see. Im very sure in the shared one, you don’t see the same pharmacist, you don’t see the same GP, its just… and then every time you see the person you have to keep repeating your story.. I mean to me, its an issue la

ET: that’s actually why they have the survivorship care plan, so they can just basically pull out your records from the care plan and then you don’t have to repeat your story to them because they can actually access the records and then read through it on their own.

ID: I doubt that will work.

**PART 3**

ID: so this is the other illness, other than cancer right?

ET: yup, like chronic conditions.

ID: so this is meant for the patient to.. what? (section 2 last slide)

ET: to compare both options and understand the difference between the two options. Then the next section, we have questions to help them decide which one they are leaning towards

ID: ok.

ET: so this section, do you feel that by drawing a comparison chart in words like that... is it easier for them (BCS) to understand it if we made it to like, maybe a pictorial form or like something easier like checkboxes

ID: probably… because this one it is like saying that this one if you have chronic illness you won’t have much care if you come to usual care but if you go to shared care, its limited also because the doctor... there are only some that are participating, right? [ET: nods]

ET: -questionnaire-

ID: its (content) a little too much and also only if you explain to me..

ET: so its difficult to understand without the explanation

ID: yeah, correct… I suppose it (presentation, table) should be less wordy

ID: huh, I don’t understand leh, im lost (qn5). What does it mean?

ET: So it’s saying like in the sense that if you can save money at the polyclinic compared to the cancer centre, to you, is that something favorable? How can I say it…

KY: So if you are able to save cost, how important is the cost factor to you

ID: versus getting the care?

KY: no, so in this case we’re just talking about the cost as a factor, assuming that, just on the assumption that both sides will be able to get the care needs that you require

ID: I think, more importantly is not just the cost but also the time, and of course like how familiar these people are in taking care of you. One of the reasons why people don’t like to go to the poly is that they consistently see different doctors… people look at you like you’re just another patient, I feel that that hampers the care a little bit la. But overall, this question is very hard to understand

ET: so one of the factors that we should consider is more like the relationship with the doctors?

ID: ya, I think that is very important… ya polyclinic you queue for hours then you see somebody who doesn’t know you. If you’re lucky, you may see the same doctor, but you queue very long for this you know? I mean here (NCC) we queue also la but at least it’s the same doctor, it probably has a shorter queue also because its something (inaudible), poly, cannot make appointment right?

ET: no, you can make appointment.

ID: oh, ok

ID: I think the ease of making appointment is very important -read…- oncologist la easier…. Patient navigation.. don’t understand

ET: it’s the navigating pharmacists

ID: ok not really… -reads and answer qns-

ET: so if you read this, does this (section3 last slide) make sense to you?

ID: -read- frankly I really don’t think people really… [KY: will read]… its like, I know what you’re trying to do, its like what colour is your aura? What kind of personality is yours… I don’t know.

ET: So rather than putting a statement like that, it’ll be easier to just generate the results to let them know what their leaning towards?

ID: ya, I think also because a lot of your questions right? Some people are like in-between so it doesn’t really accurately [ET: help you decide?] ya. Doesn’t mean that I score 10 means I am this type, but some people are like 0.5 because there are certain things they just feel otherwise? Ya. Cannot treat cancer care as just a statistics, just a number.

ET: -questionnaire- but in terms of like the slider and choosing the options, do you think these are good ways of measuring? Or would you prefer, like instead of the slider, we put numbers from 1 to 5 then they just select ‘strong agree’, ‘strongly disagree’?

ID: I suppose, but at the end of the day you still will have you ambiguous between the two? You know what I mean? It’s hard to put people in a box la.

ET: -explains section 4 purpose-

**PART 4**

ET: do you feel that you can understand the video?

ID: yup

ET: -explains info section-

ET: so with this context, do you feel that we’ve given them enough resources? Or do you feel that more resources will be better?

ID: maybe can add on a support group? If you’re gonna talk about psychological factors, add on some support group, may help to talk to people of the same… ya that’s it?

ET: yes thank you!
