# Supplementary material for: Development and pilot testing of a decision aid for navigating breast cancer survivorship care
Source: BMC Med Inform Decis Mak. 2022 Dec 15;22:330. doi: 10.1186/s12911-022-02056-5 (PMC9753367; doi:10.1186/s12911-022-02056-5)
Supplement: Supplementary file 5 — Additional file 5. Transcripts and the final decision aid prototype. [file 12911_2022_2056_MOESM5_ESM.zip › Additional file 5/ID12_transcript .docx]

**Study ID**: ID12

**Interviewer**: IC

**Date**: 4 March 2022

**Transcribed by**: KY

IC: So we will be going through the decision aid together each page at a time, and then the decision aid consists of 5 key section[s]. As you are viewing each page and section, tell me out loud any thoughts that go through your mind and I may also prompt you with some questions along the way as you navigate through the pages. So for example, maybe I can just show you. I’ll be asking for each section, what is the… how is the amount of information, is it clear, is it easy to understand and then how’s the presentation, the looks, the graphics, those kind of things [ID: Ok]. So once you are ready [ID: Let’s go], you can read and then I’ll navigate.

(Reviewing)

IC: So for the first 3 parts, the cancer survivorship - which is the first 2 pages that you saw - then physical effects, then the emotional effects, could you tell me what you thought about the amount of information, the clarity of the information, and the presentation?

ID: It’s just nice.

IC: So we start off with the cancer survivorship one? [ID: Mm] Ok, just nice. And then…

ID: The other one is?

IC: Is it easy to understand?

ID: Yes.

IC: Then the presentation…

ID: The presentation is good.

IC: 3 (questionnaire completion). Then for the physical effects with the chemotherapy, all those?

ID: The information is ok.

IC: Same? [ID: Mm] So, just write easy to understand?

ID: Ya.

IC: Presentation also?

ID: Yes, good.

IC: Then the emotional effects is the page just now.

ID: Ya, it’s just right, the same.

IC: Ok, and then, is there anything new or different about the information that was presented that you didn’t know before?

ID: No.

IC: Generally…

ID: Because I went through the whole thing (laugh).

IC: Understand, and do you think that we should include other kinds of information?

ID: I think it’s just nice because too much information will like overflow, then the patient will also get confused.

IC: Understand, ok can, alright, then can continue.

(Reviewing)

IC: So for just now those 2 parts, the follow-up care options as well as the comparing the options, so start off with the follow-up care options, the amount of information, clarity and presentation.

ID: The information is ok, just that maybe the points that you want to bring to the patient is not that clear.

IC: Ok. Then, is it easy to understand what was presented just now?

ID: It’s easy to understand but it’s just that the message across is…

IC: Is unclear?

ID: Ya.

IC: Like don’t know why this is here.

ID: Ya.

IC: And then, the presentation wise, the look…

ID: The look is ok. Good. It’s good.

IC: So what kind of information do you think should be included or would you want to know?

ID: Maybe have like more of differentiate between these 2 care [options]. I mean, maybe the information is a bit too much of… ya to throw it to a patient that is just, first time looking at this, so it should be simplified.

IC: So I would put down, I’ll change this to too much.

ID: Mm.

IC: And then the comparing the options, the one with the tables?

ID: Ok, the one with the table is ok, ya, the table is ok.

IC: So just right?

ID: Just right.

IC: Easy to understand?

ID: Yes.

IC: Good?

ID: Good (laugh).

IC: And then, are there any information that you think should be included in the table as well?

ID: No, this is ok.

IC: Then [be]cause you mentioned just now that it should be more differentiation, do you think it was easy to see the differentiation?

ID: (slide 48) It should … maybe it should be put into 2 slides instead of 1 slide.

IC: For the… for which one?

ID: This table is ok (slide 49). Maybe over here… not this one… I think, wait… this one, I think this one, I think you have to like maybe differentiate a bit because everything was jumbled together, so patient is like… It’s very difficult to understand when you first time looking at this slide. If you.. I mean, if you have this slide first to comp- ok, something like that to compare these 2, it will be easier to understand.

IC: Like showing the table upright?

ID: Correct. It’s easier to understand.

IC: Easier to understand, ok. And then, were there anything that you think will be confusing or like words, phrases, concepts, kind of thing?

ID: I think it’s ok.

IC: Still ok?

ID: It’s ok.

IC: We can go on to this next part.

ID: Ok.

(ID reviewing the decision aid)

ID: I have to submit my result?

IC: You can if you want to, but this is just a[n] exercise.

ID: Ok, I’ll…

IC: If…

ID: Actually, I’ll prefer here because all the history is here.

IC: But then if we, if for example, the history for example, your cancer history all those kind of things, is able to be shared with the community health care professionals like the sharing will be streamlined for example, would you be more inclined towards shared care? Or…?

ID: I don’t think so because first, they are… maybe they are not well trained in this kind of treatment. And secondly, they might not even read through the content or the history before they see the patient. So we don’t know what will be the end result of… ya. It’s true that you can share all the information. I mean if the information is being shared to the hospital, I think it’s still ok. For example, clinic to hospital is still ok because the doctor in the hospital are…. I believe is more well-trained in all this kind of treatment than the one in the clinic right. I think the clinics [are] all general GP.

IC: The clinic like all the general GP those kind?

ID: Ya.

IC: So it seems that you will still prefer here instead.

ID: Ya.

IC: So for this one right, are there, other than these factors that we presented here, what are the other factors do you think will be important for you or your peers when making a decision?

ID: Actually, is the experience that, is the experience and the conf… how should I say, the confidence and the…

IC: Comfort level, your comfort level with the doctor…

ID: Yes. Correct. Yes.

IC: Ok.

ID: And I don’t think a GP will know how to treat a cancer patient.

IC: Ok, understand.

ID: That’s the most important.

IC: So our idea is that they will be trained specifically from the NCC doctors, but understand your concern that even if they’re trained, it might not be the same type of care [ID: correct] that they provide right [ID: correct]. So that is…

ID: I don’t mind to go there for medications, only solely for medications, but I don’t think I will go there for any…

IC: Checkup, consultation [ID: Yes, correct] all those. Ok can. And then, how about the, these 3?

ID: It’s good. Just right.

IC: Easy to understand?

ID: Yes (laugh).

IC: Ok, alright. And then anything confusing here?

ID: No.

IC: Or generally, ok?

ID: Ya.

IC: So for the preference exercise, so we call this the preference exercise, would you think that it helps you or if we present it to your peers, would it be helpful for better understanding of preferences in follow-up care?

ID: Ya, it definitely is a good exercise, after experiencing…

IC: Showing your factors… what are important. And then, in what ways do you think that the preference exercise would be beneficial or helpful in decision-making?

ID: At least there is a choice for the patient to choose and… they can actually go for cheaper options or the expensive one (laugh), and … which one is much more convenient. Some people will choose the convenient as the top priority. But for my case, I will choose the confidence.

IC: Still prefer here?

ID: Yes.

IC: Ok. And then, can go to the next part.

ID: Doesn’t seems to go. Do I need to click here?

IC: Hold on.

(IC resolving clicking issue)

ID: Maybe is the confidence level for the hospital to give the patient on how to carry on their life after the full course… the full treatment. Ya, then that, I think is much more important.

IC: Ok.

IC: Then this part is…

ID: Ya, it’s good.

IC: Still (laugh).

ID: Yes.

IC: So for this one, do you think, are there other things you think will be important to put inside as well? Or things that maybe yourself or your peers would want to know?

ID: I think it’s good enough.

IC: Quite comprehensive?

ID: Yes.

IC: Ok, then, so I just have some additional questions. So, in general, overall, what do you think about the aesthetics like the look of it kind of thing, the graphics that were….

ID: It’s ok.

IC: It looks good?

ID: Mm.

IC: Colors wise all those things, choice of fonts [ID: Mm]. Then how about the interactiveness, do you think it was interactive?

ID: Ya.

IC: Then the navigation, do you think it’s easy to navigate.

ID: Ya.

IC: And then, do you think there should be anything that we should change about the interface or it’s generally ok?

ID: Ya.

IC: Alright, and then, so generally, quite easy to understand also?

ID: Ya.

IC: Ok, and then just some follow-up questions here. So you can tell me which one and then I will just write down here. So your answer, so for example, will you prefer….

ID: Digital.

IC: You will prefer digital?

ID: Ya. A2 is easy. A3 is yes. Part B…

IC: Part B, we went through just now, this is the 4 things that… 3 things.

ID: Ok, so this one, we went through? All the Bs we went through already, haven’t?

IC: I asked this right, yes, so it was yes right.

ID: So I think it’s only the C right.

IC: The B3?

ID: B3 is…

IC: Do you think there was a slant, or it was quite balanced?

ID: It’s balanced. The length of the decision aid was just nice. So decision-making… yes. C1 is yes.

IC: Like you mentioned just now, it will be good for the choices…

ID: Mm.

IC: Then the C2?

ID: Yes. Yes, C3 is yes. Actually is upon diagnosed.

IC: At least you know what are the options out there.

ID: Yes. Ya, I think that’s all.

IC: Then the D all those kind, quite ok, generally?

ID: Quite ok, ya.

IC: Thank you! Then, would you… do you think that you would return to this decision aid if… for information?

ID: Ya, I will.

IC: And then, generally, what are the things that you think there is new like you didn’t know before? Mostly the…

ID: I think it’s all there already.

IC: [Be]cause you went through the… [ID: Yes]. The new one is usually the shared care…

ID: Ya, this one, I think the shared care is something new.

IC: Ok can, and then, think that’s mostly it. Thank you so much. Just some demographic information I need… So age onwards.

ID: Age onwards? Where is it? Here. I key in or you write down?

IC: I write down.

ID: 47, Chinese, Diploma, date of diagnosed I think is 7 May 2012, 3, 3. I went through everything – surgery, radio[therapy], chemo[therapy], everything, I think is everything. Nothing short of… and that’s all.

IC: Ok. The diploma will be…

ID: The diploma will be under pre-u[niversity]. I think it’s under pre-u[niversity].

IC: Alright, then do you have other comments? Quite ok? Can, thank you so much.

ID: Or maybe one point is after the 10years, how long will this care, till to the end of the life or what? This is for… [what?] not mentioned over here.

IC: For the shared care is it?

ID: Yes.

IC: Ya, maybe we will look into that.

ID: Ya, it’s like 5 years, 10 years, to the end of the life.

IC: Ok, thank you so much.
