# Supplementary material for: Development and pilot testing of a decision aid for navigating breast cancer survivorship care
Source: BMC Med Inform Decis Mak. 2022 Dec 15;22:330. doi: 10.1186/s12911-022-02056-5 (PMC9753367; doi:10.1186/s12911-022-02056-5)
Supplement: Supplementary file 5 — Additional file 5. Transcripts and the final decision aid prototype. [file 12911_2022_2056_MOESM5_ESM.zip › Additional file 5/ID04 - Transcript.docx]

**Study ID: ID04 Date: 14/11/19**

**Interviewer: ET**

**PART 1**

ID: What is this? [*points to ‘endocrine therapy’ on what is cancer survivorship?*]

ET: It is the drugs like tamoxifen. So you are here [*points to woman figure*].

ID: I need to answer anything?

ET: Do you feel like in this layout, is it easy to understand?

ID: Is it easy to understand?

ET: Can you understand what it means?

ID: [inaudible]

ET: For the questionnaires, we do it simultaneously. So just now the first slide, the diagram, and this section, do you feel that the information is too much or just right because this section talks about survivorship?

ID: This one is … the whole thing or the topic itself? This is just the … Yah, so this is just the, what you need to do is, how to prevent recurrence, how to take care of this and that, but not so much … how to take note and maybe you will go into …

ET: So you will prefer more details on what you like yourself can do? [ID: Yah]

ET: These are the roles that the survivorship care plan … These are the things …

ID: [inaudible]

[*introduction – why is cancer survivorship important*?]

ET. You mention just now that… understand?

ID: Not so easy.

ET: Which part in particular do you find it a bit harder to understand…?

ID: It’s not like very straightforward, in very layman terms … like you present it, recurrence, how you would prevent recurrence then what you … every single one, you have to think a bit more in what does this mean.

ET: So if we make it more simplified like bite-sized information for you to see it, it’s better right?

ID: Better, yah. I think at that time when cancer survivors, when they are reading it, not so easy to understand.

ET: Lastly, do you like the images shown on this slide, [inaudible], or this style?

ID: I think it is okay. [inaudible]

[Mixed voices]

ID: I can understand.

ET: Do you know what is sarcoma?

ET: It’s this cancer … like… [*explanation of sarcoma*]

ID: This one I understand, but not aware when I do my chemo.

[Inaudible]

ET: Do you know what is the difference between the two?

ID: Don't really know.

ET: So what it is trying to say is that for long-term effect that you experience numbness, this numbness will persist for a long period of time; but for late effects, maybe after your chemo, after a period of time you start experience the effects. Something that did not appear during treatment, but occur a few years down the road, those are considered late effects.

ID: Okay…. because I experience, I have all these experiences.

ID: This one is when we go through or …?

ET: So basically these are what we have, at least one of the treatments [ID: Okay].

[*Going through the list of effects*]

ET: Not everybody experiences it.

[Mixed laughter in background]

ET: So do you feel like having this section will cause people to ask?

ID: AI?

ET: Aromatase inhibitors like …

ID: I don’t know these drugs, we see at the …

ET: As a user, so like because this has nothing to do with you, do you find it confusing for you? Do you think it is okay if we just not use it?

ID: I mean I will read…

ET: As you going through these slides, do you need … [inaudible] So just now this thing you don't really understand, but after I explain, you can, so how do you think we can phrase it differently? Like, do you think words are too hard to visualize, some images will be better? So like timelines just now the survivorship … help you visualize …

ID: Actually you can have… pop-up to explain what is the long-term effects as opposed to this, and not just have this inside, separate this… We don't really understand. [ET: Like normally the words got colour can click in right?] Yah! At first I thought you want to click in.

ET: That’s true. Question 1, we finish, we just repeat the same thing alike just now. How you find the information about the treatment, effects of the treatment…? Do you feel that it is too much, too little or just nice?

ID: I think it was just nice, like certain … got new things.

ET: You think you understand?

ID: Certain parts.

ET: Then, for the presentation wise?

ID: [inaudible]

**PART 2**

ET: Then we’ll move on to the next section.

ID: Have to come back and follow-up, like what I am going through? [ET: hmm]

ET: How about this section? [*usual care*]

ID: Usually the doctor will decide what is the best for …[ET: then normally you follow it] Yah.

ET: So do you feel that the usual care section, does it capture the idea of [inaudible] or should we make it more…?

[inaudible]

ET: Now we move on to the care options.

ID: So will have specialist seeing? ... [inaudible] Where can you find this? [ET: who, PCP?] When I look at this, I am like okay, but where? Are you doing like there and here?

ET: Here, if you are in this, you prefer polyclinic or GP, the difference…?

ID: GPs will actually help see the, plan for the… ? [inaudible]

ET: So like it’s like other conditions other like cancer, high blood pressure, diabetes, high blood cholesterol - these are considered comorbidities. So, when you go and visit these doctors …

ET: So Doctors are? So how do you witness?... Maybe this part we can … [inaudible]

ID: But the thing I don't understand is, why do you need to put these two for? What you can make it is outcomes, a group of oncologists, I mean these chronic diseases, of course, I won’t come to oncology, because it is … to go to GPs.

ET: So basically right, you need think about this shared-care, they are not just seeing you separately, but they are all in the know. So like, know as in [ID: share the information?] Yeah. [ID: only selected GPS?] Yeah, only for this program, for now. So for example, let’s say now you are visiting separately, you go to polyclinic, you need to tell them your whole history, but in this system, they will what to look out for because they already know your history.

ID: That is provided if it’s polyclinics, if those GPs, they won’t be able to have a linked system.

ET: So now, it’s something new, we haven’t try out yet. So, we will start out in the polyclinics, will see how it goes. So that means this explanation didn’t really tell you like who … ?

ID: Because I don't think polyclinic will come to oncologist, I don't think this one will be linked to this one.

ET: So maybe we need to highlight that your information is shared more clearly?

ID: Yah, and maybe it’s probably…

ET: And then still have the pharmacists…

ID: Where? [ET: Orchard there.]

ET: So can understand this? [ID: Yah] So because there are so many of them, we want to write down the roles for you, exactly what they do.

ID: What is rapid access?

ET: So like now in this case, care will be shared with polyclinic. So, the polyclinic find out something abnormal, and if they suspect recurrence, you can immediately … so access back to oncologists, so we need to make this clearer?

ID: Yes, maybe for polyclinics, it’s the phrasing.

ET: So do you know what is the health promotion?

ID: Related to …, basically sometimes… all these things…

ET: Is it? [ID: I don't know]

ET: So in this case, you are half correct...like exercise more, eating less unhealthy food…

ID: Then, you need to list the example.

ET: Then for this one, a bit confusing. So, what we are trying to say that basically this thing will be done in NCC here but will not be done by this.. like you feel that … survivorship care plan… [inaudible]

ID: What this means is that what is the information that is being shared out? [ET: Correct] So, that means it’s not all the information being shared? It’s only all these information is being shared, not all the information.

ET: Not like whole entire medical records of like to every single doctors you see, no. So its limited to like your … containing… screening, what advice that they should look into and vaccination … So all these are part of the survivorship care plan, it appeared previously in the previous section. Before you read this slide, do you know what’s a survivorship care plan; Do you feel like we should put this in front so that…?

ID: In front one, can I see?

ET: So just over here it says… things in survivorship care plan, it appeared… So you don't know what it was right? So the explanation slide, do you feel like we should put in front?

ID: Maybe you should, not maybe put it in front, maybe you should like make a remarks or what to say, refer to the next slide [ET: for more information] something like that.

ET: Okay, then now it’s …. [inaudible]

ET: Is this a bit hard to understand?

ID: Can understand, just don't know whether because … as a cancer survivor, whether is it high risk or low risk, we want to see an oncologist, because… cancer is still a very scary thing. So, it’s like if you are not in the hands of specialist, you’re in the hands of a GP and if there’s a recurrence …

ET: Yah, so like this part is just ... [interrupted] So when you visit your oncologist consultation, how much do you pay? [ID: 20+] So it’s like … So it’s … the consult, between seeing GP and … Compare the cost between seeing the GP and oncologist, so for polyclinic…

ID: Slightly less, the polyclinic cheaper.

ET: But like you said, some people prefer the polyclinics.

ID: …polyclinics usually, they very young… a bit scary…

ET: You don't feel like …

ID: Yes… [inaudible]

ET: This is too much information?

ID: This one? I would want to have a regular follow-up with the oncologist.

ET: So like this is something you have, and you don't feel safe with your polyclinic doctors. So probably in future like that, if only … should consider other participants, this one.. how would you have said?

ID: How do you want me to say …

ET: Because it’s for general members…

ID: Because … maybe need 10 people will …

ET: Some cancer survivors for more than 10 years out of their chemo, they finish… then they are considering like, which option, what are the other options…

[Mixed voices]

ET: So like just now we say that you know shared care for example, remember earlier on… will all be... so for this case like now you see a GP and oncologist, then it’s like saying do you consider your past experiences, how would you … I mean you want to choose this or…?

ID: I suppose. [Inaudible]

ET: Is it because there are too many like … [inaudible]

ET: So for the first section same thing, so just now you mention... some parts a bit similar, but do you like the table format?

ID: The one you put is okay, it’s just the … [inaudible]

**PART THREE**

ET: So this part is what you will do is … [inaudible] ...help you like summarize your considerations… You just circle, it was later at the back, this is a line [scratching noises

ID: I do have regular doctor … [inaudible]

ET: Basically this one have a scale, do you feel that it is most important for you or you not sure… [inaudible] or still like prefer …?

ET: What…

ID: No… won’t be able to answer, you need me to ….

ET: So let’s say, ‘cause now you say, if you say, … are you more comfortable or are you…? You can be honest about it because it is your personal opinion.

ET: And the next question is … [inaudible]

ID: I think this should be like, because when you ask me, for me this is not the best way, to experience… but if you have 100 over compared to, so how do I describe…

ET: So you’re trying to say that while the cost is important, but in this case, because it is not so significant [ID: Yah]. So, many we can change the phrasing of the question to make it more related to price…

ID: Unless they are saying that sometimes, I mean like I … at subsidized rates, then …

ID: It’s still the same, similar, same one. The difference, talk to … not just with less … [inaudible]

ET: And the second one is your case, seeing your oncologist… what is your experience with? [mixed voices]

ID: Convenience, convenience is polyclinic.

ET: Like you said, … [mixed voices]

ID: Preference-wise, it’s here specialist. But, you’re saying convenient, polyclinics is more convenient but preference is oncologist.

ET: So right, how the questionnaire work is because you rated convenience as not so important to you, so even if are closer to the polyclinic it’s not going to have a big effect on your decision.

ID: So don't need to answer.

ET: So phrasing of the question.

ET: Its asking you if it’s very difficult to make appointments, it’s also like convenience factor, is it easier to make appointment at polyclinic, or no difference…? So we’re trying to say, do you feel that convenience …

ID: Very difficult …

[Inaudible]

ID: So you can take that out?

ET: So change this question? [ID: Yah]

ET: Then I think we can skip this qn.

ET: So this one, pharmacist navigators.

[mixed voices]

ET: Not very clear is the question? [ID: Yah]

ID: It is important because every time you go and see the GP, but also crowded actually…

ET: So you must resay everything.

ID: Yah, so that you know what… what thing they can help, because certain cancer is… [inaudible]

ET: So in this case, do you want them to contact each other, communicate with each other?

ET: You view your responses. So by going through these questions, it’s supposed to help you understand better like which care you prefer. So when you see the even number questionns, you rate how important they are to you. So, like you say this is very important, these are not so important factors, these are your… So for your case, you consider the healthcare professional more important, whether or not it is expensive, it’s not that much difference, not so important.

ID: Because I think it is not significant differences.

ET: Then the other odd questions, so the higher the scores you rate, 3 5 and the rest, it shows that you prefer shared care. But in your case, 3 or 5, is more of like in the middle, not so much, then .. maybe you prefer… so basically do you feel that we didn't explain well? Like what are you supposed to do? [ID: Like what?]

[inaudible]

ET: Do you feel like, let’s say it’s survey, at the end we generate the results.

ID: That will be useful

ET: Or show you at least a summary. [inaudible]

**PART 4**

ET: Okay, so this is the last part, basically, … just now we say shared care is something new right, we are going to pilot it, so this is the information that we will give … we want you to help us see if you can understand.

ID: I understand.

ET: So this is a short video.

[Video plays]

ET: So let’s say you are to participate in this study, then I show you this video, d oyou know what is expected of you?

ID: I understand, that's because I been through all the stuff…

ET: Yah, So the in front gave you the knowledge [ID: Yah]. Then, when you see through this video… [inaudible] So this is the short version of the video … it’s like a summarized version because some people prefer to read.

ET: Then this lastly is the info section, whereby you want to find out more, you just click on all these links, it will bring you to … but do you that like if I tell you what you’re looking for … is it enough information to tell you what you are …? Or, do you prefer something like… So I show you like the example for you …

[Opens internet links]

ET: They are from reliable sources. So some of them are like from Singapore websites, some are not… So, with this right, do you feel like perhaps you, before we can put more links, as in like we maybe we describe it more clearly for symptoms specifically to … In other words, do you think like 5 links enough, should there be more of like, it's okay?

ID: That's like for me, that’s the … [inaudible]

ET: So give them more specific…

ET: Because now when you go to the website, you still need to ownself find, but do you think it will be okay to go directly to the websites?

ID: Yah, I think it is good to have all these, so for yah, more specific some will take… Instead of google, they just click, cos you click the link... you get too much information, sometimes … ask oncologist …, don't go to search too much information, it will make you worry, but anything ask them.

ET: Sometimes like you when you randomly click on random websites, you don't even know it is correct, then you scare yourself.

ID: That's the … you read… then you say like … you scare yourself.

ET: So the last step is just completing this survey. Because you have finished all these. Then actually we have … print out, and discuss with healthcare providers, these are the references used. So based on what you gone through, do you think that websites will be good or other forms?

ID: Everybody go that website…

ET: Just now because I … do you feel that the …

[questionnaire completion]

ET: But do you like the layout in the case that … all the lines… But for that right do you prefer some numbers, marking this, but you can still put anywhere …

ID: ‘Cos this is like 4 this is 2, that one is okay, so we can see that need to show the …

ET: Just now the video, that information, is it …

ID: Easy to understand

ET: For the video do you find it like clear? Or was it … ? [phone ringing]

ID: Then lastly, the … [mixed voices], presentation?

**ACCEPTABILITY QUESTIONNAIRE**

Do you find that we were bias?

ID: Because there’s a lot of information on shared-care, then you feel like we are selling to you [ET: Yes]. [inaudible] If I have a chance I will not do shared care.

ET: But that's after you read it, but let’s say if you never see this, and doctor say to you, do you want shared care?

ET: So you already know what you want? So maybe it wasn't too useful for you?
