# Supplementary material for: Development and pilot testing of a decision aid for navigating breast cancer survivorship care
Source: BMC Med Inform Decis Mak. 2022 Dec 15;22:330. doi: 10.1186/s12911-022-02056-5 (PMC9753367; doi:10.1186/s12911-022-02056-5)
Supplement: Supplementary file 5 — Additional file 5. Transcripts and the final decision aid prototype. [file 12911_2022_2056_MOESM5_ESM.zip › Additional file 5/HCP02 - Transcript.docx]

**Study ID: HCP02 Date: 25/11/19**

**Interviewer(s): ET & KY**

HCP: this angiosarcoma and osteo (sarcoma) is quite medical terms and the connective tissue as well. Whether there could be some other words that could help make it more simpler…this is all pertaining to only breast cancer survivors right? (lung cancer pop-up) ok I think this is good but for the smokers one I’m not sure if you want to elaborate a bit more as to what is the significant number of cigarettes smoked; like how many pack years, whether its 10, 20 or 30. But I think usually we take it as 30 pack years to be significant.

KY: so do you think the patient understand the concept of pack years?

HCP: maybe then you just put how many cigarettes a day, then the number of years you see whether you want to put that in or not. But at least per day, they should be able to understand. 10 cigarettes a day for 10 years then it’ll be 10 pack years.

HCP: Do I need to click this? [ET: you can click inside] ok

HCP: actually, quite a common complaint is numbness… numbness over surgery site? What is this due to? Seldom have this complaint but more of the numbness of the legs. This is what you mean by neuropathy is it? Because most of them have the paclitaxel side effects so they have numbness – mainly divide on fingers and toes. So what is this referring to? I’m not sure what is the surgery site unless you’re saying you cut the nerves, is it? Which is not so common but I guess you can put it over as a long term maybe… this is long term and late? Which one is later? What is the difference?

KY: so do you think the distinction is very confusing?

HCP: yes, its very confusing, I don’t understand what is long term and late.

KY: this one, if it is the paclitaxel all those (side effects), it should be the chemo one. But I understand your concern that it’s not that intuitive like when you think of surgery you think of neuropathy… maybe we’ll just keep the numbness at the surgery site because sometimes it may just be the wound area which may... because definitely you have some sections and everything, don’t know whether… some of them maybe they just feel that that area feels different from normal. We can just change the wording... we will take note of the neuropathy part to not confuse it with the chemotherapy things.

HCP: I mean I don’t know, unless you want to use a broader term because most of them don’t really complain of numbness but they may complain of tightness or they have seroma, so they have small, maybe the skin effects. You can see how you can use the term closely?

KY: so in this case, do you think we should not split up or how do we frame some of the…?

HCP: How many types of side effects do you have? Before that there’s a short term is it? Is there acute?

KY: ya but because the acute ones are much earlier on [HCP: it’s not under here. So this is all the side effects you have is it? I mean long term probably more intuitive, what is late? Post treatment is it? Post treatment effects? Unless you want to put it as a post treatment, that means its sort of related to the surgery and the chemotherapy. Whereas long term then it can be other things as well. So maybe unless you put it as that

HCP: sexual function… what do you mean by sexual function?

KY: or do you not related that to radiotherapy?

HCP: talking about radiotherapy of the breast is it?

KY: in this case, yes

HCP: you’re talking about fibrosis or mastectomy? But mastectomy will be under surgical right? Is the sexual one over at the surgery also? [KY: sexual function? No, it’s not at the surgery] it’s only here is it?

KY: so you don’t think it is too accurate?

HCP: I’m not sure what you mean because you’re talking about breast only, are you talking about body image? [KY: yes, but is it too far-fetched or do you usually not counsel patients on that aspect in the context of radiotherapy?] ya I thought that maybe you put it under surgery if it’s the removal of the breast. I mean in terms of the sexuality. I guess im not sure what you mean by function but they may not perceive that way. Whereas for radiotherapy, I guess if there’s any sexual function, it’s gynae cancers where they really cause fibrosis. Then that one will affect the sexual intercourse itself.

HCP: pneumonitis.. you want to… this one is a medical diagnosis is it? This one is a symptom is it? So you want to keep it to a… I mean the patient may not understand what is pneumonitis and inflammation of the lungs so you want to put it as symptom? But as a symptom, it’s also here right? This one you also have pneumonitis, so is it both the same? It can be long-term or late is it? So unless you use the same, I mean otherwise, shortness of breath will be the easiest that people can understand, but they won’t know whether it’s due to pneumonitis or lung scarring, so when they present with shortness of breath, you can say it’s not due to lung problems like asthma or heart problems but it’s a complication of the (cancer) treatment itself. I think this one they understand…

HCP: so your long term and late are very similar is it?

KY: some of them overlap cause they can be persistent effect or only occur after [HCP: this one (late effects) is what is the time frame?] there’s no time frame, it’s just after the treatment ends. So it may not be directly what the patient experiences while on treatment, that’s why… it may happen at a later stage that’s why I think it’s more of to warn, help to prepare them that certain things that you experience, even though it may happen in isolation or temporal in this sense, but it’s still related to the treatments that you have received. But we understand the confusion. Some of our participants also feedbacked. So we’ll try to see if we can either merge some of them and then change some of the wording for some.

HCP: I guess you can merge them. It can happen at a late or long term but what is different that you’re talking about is this is it – heart disease? Heart disease is long-term right?

[KY: it’s late right? Cause there may be some cardiac changes but the real clinical manifestation, if there is, is a risk factor for the later part, that’s why we parked it under late]

HCP: so late is longer.. uh, later than long term is it? Haha very confused. Maybe you put a timeframe like how many years, 3-5 years or more than 5 year then maybe people can conceptualize because we’re still thinking about the timing and then how to talk about this. This one you’re talking about what coronary artery disease? Because you’re not talking about Herceptin right? You’re not talking about cardiomyopathy right?

KY: no, because once you radiate that area, there will be some scarring of tissues. I think the purpose of putting late is more of a “all these treatments are risk factors for some of the eventual conditions and disease” like cardiomyopathy like you said, but all these don’t occur so acutely.

HCP: ya, because I did ask the radio-oncologist “does your radiotherapy cause heart disease?” so they say unless you actually radiate at that part (chest) or like NPC… NPC not so much. It’s where the cause of radiation… and the radiation causes damage to the blood vessels, so its more of ischaemic heart disease, coronary artery. So it’s like a cardiovascular risk factor.

HCP:Multitasking uh? Why you choose multitasking? (cognitive example) are these the ones that are described as cognitive related problems? [KY: ya, it’s like all the test, when you talk about cognition, these are things that they measured and that’s when they demonstrate that (there is cognitive impairment)… or do you want us to put multitasking towards the end as an example?] ya, I’m not sure if this one can be understood by patients but usually we talk about executive functioning, if that’s what you’re referring to. Usually executive will be all your shopping, telephone bills, your finances. So that one, maybe patients are not so aware but that’s how we divide basic and instrumental activities of daily living.

KY: but that’s not exactly cognitive right? How about maybe concentration and memory – ok? [HCP: ok] these 2 are more intuitive. I think the multitasking part is a bit [HCP: you want to be more specific?] so if you talk about shopping all those, it’s more related to their ADL, or even your functioning status [HCP: those are executive function. So like dementia patients, those are the ones that go, so you’re talking about cognitive, you mentioned. I mean like you can use (multitasking) but some people may think of it more as executive functioning. So you’ll cover quite a lot of things] so for multitasking, do you think patients will think of it as [HCP: multitasking seems as though you do a lot of things at one time. Like you can talk to someone while you’re shopping or while you’re doing (something else), but that one is not really cognitive per se. it’s like you’re talking about complex function like executive functioning, you’re saying that people who have cognitive impairment they will have problems managing the phone because phone is quite complex. They will have problem at the ATM or paying bills, so those are more specific examples, or you can put it as complex activities or] I think because like you said, handling the phone and everything, all these they are more of the auto functions part [HCP: motor] but multitasking, as cognition wise there’s still the motor functions part all the executive... But I think for multitasking, why we part it under cognitive is more of because that requires a more cognitive-level kind of thing [HCP: you’re talking about concentration ah?] no, multitasking, because it requires you to do two action which is actually two executive function concurrently, so that in itself is a bit more.. involves a bit more cognition. And another thing is because this is what people test. So it’s a standard test like your concentration, your memory and your multitasking level. So when we run cognitive test to even prove that the cancer patients have cognitive problems, they use tests related to the multitasking part.

HCP: ok I mean if it’s a scientific thing then it’s ok, but you have to explain

KY: ya so we try to make sure that patients can just get it (understand), so if it’s causing too much confusion then I think we better just focus on the concentration and memory

HCP: ya because all these will invite a lot of questions you know. So if that’s the case then maybe you can tell them that at the beginning, some of the things may be repeated like sexual function in radiotherapy chemotherapy, so it can take more than one area.. tingling sensation at the surgery site… this one you didn’t talk about the paclitaxel is it? [KY: oh, this one should be removed, just tingling sensation at the nerves] ya, just remove this one (surgery site), just tingling sensation, cause most of them is at the limbs, fingers

KY: do you think we should specify the chemotherapy? Because I think we’re trying to not introduce too many drug names into it, because we already list out..

HCP: oh you mean like the taxanes all those? I guess you don’t have to.

HCP: I mean usually it depends on the degree of weak bone. Usually we call it soft bones or you can put it as weak bone… sudden hot sensation… changes in menstruation... this is to test their awareness or test that they have these problems? [KY: just to tell them that, maybe associated, they may experience these problems] what is the sexual dysfunction which you’re talking about?

KY: you mean like, what specifically? [HCP: to tamoxifen] er, we’ll clarify that?

HCP: I guess usually the one we tell them is the blood clot and the cancer effect. You have the cancer effect right? The endometrial cancer? [KY: ya, that’s the previous slide one. The one we talk about the risk for recurrence and other second cancers] oh, so you park it under that? So you don’t want to put it under tamoxifen? [KY: do you want us to put it under, combine?] ya, cause these are the two things usually you will tell them. Usually 1.5% with 5 years of tamoxifen… how about weight? The weight you put somewhere else is it? [KY: you mean gain in weight?] ya, [KY: we didn’t..] weight is quite a common thing. Usually due to hormone deprivation which is either due to the tamoxifen or the AI. So this one (cholesterol) I guess you can just put lipids cause it can be triglyceride, not just cholesterol. [KY: do they understand lipids? Will the patients understand lipids? Or fats? We put in cholesterol, at least it’s a friendly term] ok but its not accurate. It’s not scientifically accurate [KY: so what would you tell the patients?] ya.. I mean you can put “cholesterol/lipids” then if they ask… yeah cause usually it’s both it’s with the hyperlipidemia. Then the sexual dysfunction im very sure. Is it due to premature menopause… AI…Actually tamoxifen doesn’t cause this meh? (vaginal dryness etc under AI)

KY: so I think it’s parked under sexual dysfunction…

HCP: ya, so maybe both of them can cause early menopause… [KY: induce menopause?] ya, maybe you use the word induce because we don’t really call it premature menopause, cause it’s an actual condition where patients just go into premature menopause. So you can use “treatment-related /-induced menopause” so some of it are overlapped.

KY: cause if it’s AI, it’s already post menopause

HCP: then why should she have vaginal dryness? Cause already post menopause.

KY: ok confirm that

HCP: decrease sexual drive.. this one is the one that they listed as a side effect is it? How did you get this list? [KY: synthesis from uptodate that kind / PIL, but we double check again on the frequency as well] ok ya, cause most of the time they have a concern with the osteoporosis part so this one with this as well as the cancer. Then the.. ok

HCP: nothing ah? No late effects is it? [KY: not enough studies, just based on case reports] so mainly it’s cardiomyopathy only right?

HCP: this is not common you know, because some people (oncologists) they don’t really recommend gynae unless until after… for extended treatment. Because some oncologists are quite worried if you refer too early, they will do a lot of test and most of the time, the risk is not very high in the first 5 years but definitely after. If they take an extended, after 10years then we would be. So the pelvic examination, I guess this one you’re talking about ultrasound right? To see the thickness of the womb. You want to put it? But I don’t know if they really do a pelvic examination per se. Examination sounds like somebody do the test [KY: this one is from IOM, the survivorship care ASCO one, but I think taking it into a local context is it uncommon?] from what I see the gynae do is that they just do ultrasound and then if they are due for pap smear then they do pap smear for prevention. That is what they usually do [KY: but it’s not routine that you see them having a pelvic examination once a year – kind of records?] I think nowadays, with the ultrasound, they don’t do.. [KY: so ultrasound they also do it once a year?] they do once a year or 6 monthly depending on the thickness, because they need to monitor the thickness of the womb lining.

HCP: (not sure which test – bmd or blood) This one again, depends, usually we would do it every year. This one may be a bit different from overseas. You can put 1-2years.

HCP: -clarifies roles of HCPs based on discussions from other study (BASIC)-

HCP: This is here is it, in NCC? (cost table) what is this? Same is it? (cost table for NCC on other column)

HCP: quite long ah this… did the patients find it difficult? A bit long ah…. (length of DA)

HCP: I thought this is quite nice, quite refreshing to just now all the wording (values section, change of pace) maybe can cut down the content cause some of them are a bit of duplication like sexual that kind… what do you mean by close relationship? Is there another word for close relationship? This one is already a regular doctor is it? [KY: so you mean the word ‘close’ is not (suitable?)?] mm or you can stroke some other thing. I guess people will probably ponder. What do you mean by close relationship… close in terms of what? That you can just share freely? Or just.. [KY: more trust] oh trust… [KY: but it’s also very weird to say “do you trust your doctor?”] I mean trust in terms of medical opinion…? [ET: what about ‘comfortable’? “are you comfortable with your doctor?”] ah ya, “are you comfortable to share with your doctors?” you can put stroke if you want to elaborate then maybe they can understand a bit more. Comfortable to share personal things. Comfortable to share cancer-related issues. So they’re supposed to do this…-read other qns- ya I guess if you use the word comfortable probably easier..

HCP: cost…………….[ET: is this hard to understand?] ya, just say is it cheaper la! Cause people will read a few times.

HCP: why you need three options, more convenient, less convenient, equally convenient? You already have a line here right? 6 and 7 are different is it? How important and how convenient..

KY: a factor can be very important but. Ok so the first one, 6, is to measure whether this factor matters to them. So if to me it’s the most important, and polyclinic is the nearest, I will just go to polyclinic. But if it’s a least important factor, even if its more convenient for me to go to the polyclinic, it is not what I want. That’s the difference between the two

HCP: So I guess you can talk about this in relation to everything or is it just cancer care only? That means comorbidities and cancer care.. or you just talking about.. is it just for the cancer part? If so, I’d definitely want to come back but if you say.. because they can be in either usual or shared care, means you’re talking about just for cancer care and not comorbidities here…

HCP: how are they going to indicate? There are no bars [ET: it’s a slider for them to drag to indicate] ok so I feel that most people will just put here. 50%. Because when you say how important, a bit difficult to.. ok they can either put it here or here, but how are you going to quantify it? You don’t want to put just fix (25, 50, 75) is it? [ET: some people are in the middle] but how are you going to describe your statistics if you have so (many) variable? [KY: maybe you see until the end] ok

HCP: ok so this part.. there’s nothing to click is it? This is the questionnaire is it? [ET: this is to summarise based on their answers, how to interpret the results.]

KY: so you said its more arbitrary where they drag but we’re not collecting data on their preferences, rather it’s an exercise for them to find out if they are more inclines towards (which care) [HCP: it’s for themselves is it?] yes, how do you think we can improve this preference elicitation exercise?

HCP: if you’re not going to quantify then just make it 4 groups then at least they sort of know what is more important to them and what is less important to them. Because with a sliding scale, I don’t know, I think it’s hard to remember also. And this is for themselves right? So unless you want to have a summary page, you want to remind them what they have chosen…

KY: so what do you think if we asked them to rank their factors. Just now we had a series of questions to ask if a factor is important to them, then they will slide on the scale. But what if we asked them to rank instead, is it easier? [HCP: yes, I find that is easier. It must be in order, probably easier]

KY: -explain removing trial section in future-

HCP: this (video) is quite good, can get them to understand. Ya, I thought this video is nice, very pictorial.

HCP: (last section) -clarify if other resources need to be read within one sitting- where is this from? (international resource) overseas? This is quite interesting, quite practical things. -go through other links-

KY: ok end, any other comments?

HCP: -clarify if we used latest guidelines- so I thought the video was very good, very pictorial. -clarify purpose of DA to be shown to who in the trial-

KY: we will followup on the side effects part

HCP: maybe you can simplify or make it into one page

KY: so for your options, you mention some information duplication and can be quite lengthy. Anything particular thing that you think we should [HCP: no, because this one (table for HCP roles) is it necessary for patients? You want to have it more pictorial? Cause you want to talk about certain points only, I guess its physical and psychosocial benefits. So the physical you can elaborate – comorbidities, the psychosocial is like stress. Just 2 points to they can understand better]
