# Supplementary material for: Development and pilot testing of a decision aid for navigating breast cancer survivorship care
Source: BMC Med Inform Decis Mak. 2022 Dec 15;22:330. doi: 10.1186/s12911-022-02056-5 (PMC9753367; doi:10.1186/s12911-022-02056-5)
Supplement: Supplementary file 5 — Additional file 5. Transcripts and the final decision aid prototype. [file 12911_2022_2056_MOESM5_ESM.zip › Additional file 5/ID10_Transcript.docx]

ID10

**Study ID:** ID10

**Interviewer:** GT

**Date:** 5 January 2022

**Transcribed by:** IC

GT: Okay, so we’re going to go through this decision aid together, each page at a time. This decision aid consists of five key sections. As you’re viewing each page and section, tell me out loud any thoughts that go through your mind. I may also prompt you with some questions along the way as you navigate across the pages. So if you are ready, you can start.

ID: Okay.

GT: Yeah you can

GT: Yeah you can click over there. The “I have read and understood the above”. Yes. Then you just read through.

GT: So also along the way, I will be asking you questions about the amount of information,

ID: Mm.

GT: Yes, the clarity as well as the presentation.

GT: Oh, wait. Okay.

ID: Do I start from one?

GT: Correct, start from one.

GT: So you pause here for a while. You finished, you have finished reading already right?

ID: Yeah.

GT: Okay. So I would just like to find out from you, for this section right, how do you feel the amount of information is? Do you feel it is too little, or sufficient or just enough or there’s too much information?

ID: Sufficient for me.

GT: Sufficient. Then, how about clarity-wise, is it easy to understand or it is, do you find some parts you don’t understand, or?

ID: No, for me, easy to understand.

GT: Easy to understand. Then how about the presentation? Do you think it is poor, fair, good or excellent?

ID: Oh it’s good. I like having pictures.

GT: Okay, that’s good. Okay can, you can continue on to the next section.

ID: For this right, are you talking about, like, during the treatment and after the treatment, or like after ten years something like that? What is the duration that you are talking about?

GT: I think, could be different ones actually. Cause different side effects might be, like some might be immediate and some might be more long-term.

ID: So you are not segregating them right?

GT: Yeah. Yes. Just grouping all the side effects.

ID: I didn’t have this one.

GT: You didn’t have?

ID: No I mean

GT: I heard though you didn’t

ID: Yeah. Yeah, I have the first one, second one, and last one.

GT: Mm. For tamoxifen.

ID: Yeah. Yeah, I have this one.

GT: So which one did you take out of these three drugs?

ID: Sorry?

GT: Which drug did you take?

ID: Letrozole and then I think the last two years Dr Rose, because I have a lot, like muscle pain, so she switched to exem, exemestane, is it?

GT: So far, so far the effect is, so far taking the medicine, is it okay? The exem

ID: Yeah.

GT: The exemestane.

ID: Yeah. Yeah. I have my cholesterol level went up. As soon as I switch to letrozole.

GT: Mm.

ID: Then I joint/muscle pain.

GT: Okay.

ID: Next section?

GT: Mm.

ID: Next one?

GT: Okay, so it’s, wait first, so for “Physical and emotional effects” section, how do you find the amount of information?

ID: It’s good.

GT: Enough?

ID: Yeah.

GT: Okay. Then how about the clarity? Easy to understand or some terms you don’t understand?

ID: No. I think it’s easy to understand and it’s clear.

GT: Okay. How about the presentation?

ID: You mean the pictures?

GT: The overall, the, with the words as well.

ID: Erm, it’s okay for me.

GT: You want to give fair good, fair poor good or excellent?

ID: Good.

GT: Good. Okay can.

ID: Maybe you can change the color for this, the heading.

GT: What, what color do you suggest we can change?

ID: Blue?

GT: Blue, but blue will it look dark, like more sad the color like blue?

ID: Erm, do you, do you factor the, the effect of the color?

GT: I think we, yeah.

ID: Oh, then you want to make it

GT: I think because that’s why we use yellow,

ID: More

GT: because it’s more bright.

ID: Okay.

GT: Yeah.

ID: Okay then it’s good. Should I go to next section?

GT: Yes.

ID: I haven’t follow up with the gynae for like, two years.

GT: How come you didn’t follow up?

ID: Because, I make the appointment, and then, then last year was too busy, and then, you know right? They only follow up for one year right. So they, so they discharged me then. If I want to go back to see the gynae I need to start, then Dr Rose said not necessary so I said okay.

ID: Are you sure you see the same doctor at polyclinic?

GT: Ideally we try to make them see the same doctor, but sometimes, in some situations, the doctor is not available, then might have to refer to another doctor.

ID: Yeah like those, I think, like five years ago right, I, I went to polyclinic for my diabetes follow up, then every time I see a different GP

GT: Yeah, this is actually a different one. This is called the family physician clinic, which is a different concept. That means these doctors are allocated to this clinic where they will, they have certain slots that they, that they allocate to the doctor, that they will see that same patient. It’s a different clinic, like in the polyclinic itself. Yeah. So if you go normal, (repeated) polyclinic, will just see then different doctor every time.

ID: You mean I can

GT: You can go through the family physician clinic, then you, you are more likely to see the same doctor.

ID: But where, where is this located? In every polyclinic or is at certain polyclinic or?

GT: Not sure if all polyclinics have, but I think most polyclinics do have this, this family physician clinic.

ID: Do they do?

GT: I think so, but I’m not sure whether all have.

ID: Oh.

GT: Mm.

ID: But how do you get referred to family physician clinic?

GT: That one I’m also not very sure.

ID: Normally, normally they just send you to a just a

GT: I think maybe the family physician also sees more different kind, like, like the chronic conditions

ID: Mm.

GT: like cholesterol, all that. Yeah. Now if you’re just going there for general sickness, then maybe it’s not the family physician clinic, it’s more of the general clinic.

ID: No, I went for my diabetes, it’s not the...[?]

GT: Oh you still see the general clinic one?

ID: Yeah.

GT: Yeah. Because this one is also more expensive, to see the same doctor you have to pay a bit more. The more, more expensive

ID: No, they didn’t even offer me at all.

GT: This option they didn’t offer to you.

ID: Yeah.

GT: I see.

ID: Should I go to four?

GT: Yeah.

GT: Okay. So for this section on usual care right, how do you find about the amount of information?

ID: I think it’s good.

GT: Okay. Just right? Just right.

ID: Yeah.

GT: How about the clarity?

ID: Maybe less words.

GT: But easy to understand, or?

ID: Yeah, it’s easy to understand.

GT: Okay. But you feel like, it should have less words.

ID: Yeah. Like this, this, like this images at the bottom is good right.

GT: So more pictures

ID: Yeah.

GT: Okay. Then how about the presentation?

ID: Good.

GT: Good.

ID: I like it. Yeah.

GT: Okay. That’s good. Yeah. So after this you can continue to the shared care section.

ID: So this family physician, you mean Dr, Dr. Dr Lee is it?

GT: Yes.

ID: The one that I see at the polyclinic.

GT: Yes.

ID: Is this you?

GT: No, this is the pharmacist.

ID: Oh okay.

GT: Yeah.

ID: They don’t remind survivor on their next medical appointment

GT: Yeah, maybe this one not so, not so accurate.

ID: Yeah. I think the pharmacist contacted me like, two times? Or two or three times.

GT: Mm. Yeah.

ID: Is this, is this care plan like a, it’s a online, online document?

GT: Yeah, so it’s like a word document that, that was sent to the oncologist as well as the family physician, and the pharmacist also. They all, they all have access to this document.

ID: Oh, so they can access it right?

GT: Mm. So for this shared care section, how do you find about the amount of information?

ID: It’s good.

GT: Just right?

ID: Yeah.

GT: Okay. How about the clarity?

ID: Very clear to me.

GT: Okay, that’s good. How about the presentation?

ID: Yeah, nice.

GT: Okay. Good?

ID: Yeah.

GT: Okay can. Then can go on to the next

GT: Okay. At the consultation cost there is a question mark. Do you see the question mark?

ID: Yeah.

GT: Okay. Hover over it. And click it. It should show, it should pull out a table. Yeah.

ID: Ah. Cool.

GT: Okay. So for “Comparing the Options” section right, how do you find about the amount of information?

ID: Yeah, it’s good. Because of the pictures, I, I can understand straight away.

GT: Okay. How about the presentation? It’s also good?

ID: Yeah, it’s good.

GT: Okay, good.

ID: Oh, I don’t, I don’t understand this.

GT: Ah, this is just an example actually. Like, we use the, the cafeteria example to explain, to, to see which side you lean on, whether it’s usual care, or you are indifferent or you are shared care. It’s just an example.

ID: Oh okay.

GT: So if you are choosing one, that, one,

ID: Do I need to choose one? No right?

GT: Not really have to choose. You can just help us to see and yeah, see the questions, whether they are relevant. After this there will be some questions that will show you whether the, to see which side you are leaning

ID: Can you put a different color, can you put different color for one two three four five?

GT: Different color for everything, is it?

ID: Yeah, every bubble.

GT: Oh. Maybe, yeah that, that is a suggestion. That, yeah, that’s possible I think.

ID: So I go next slide?

GT: Mm, next slide.

ID: I don’t need to answer right?

GT: Not really. Just help, help us to see like you think whether these questions are, whether these are the factors that you will consider, and whether or not that you think there should be other factors also that are not, are not listed here.

ID: Oh. Yeah. These two questions are good. Because I’m not sure I’ll be so confident. But I’m comfortable seeing the, the, the polyclinic doctor.

ID: For question three right,

GT: Mm.

ID: The, the communication between the oncologist and like family physician right?

GT: Mm.

ID: It will be important. But I, I won’t know how

GT: Yeah.

ID: How it really works right?

GT: Correct.

ID: Yeah. How useful. Actually I, I think

GT: Mmhmm.

ID: Actually I think the family physician is good enough for me.

GT: Okay.

ID: So, good.

GT: So this is acutally like for, you say first.

ID: So this is like...[?]. Mm.

GT: Oh, you say first?

ID: I say these questions are good.

GT: Ah, okay.

ID: So yeah. [ID picks up a call]

ID: Sorry.

GT: It’s okay. So this is like a questions, for like a survivor would also to consider whether they should go towards shared care, or whether they should remain in shared care. So do you feel that there might be other factors over here that we didn’t list out that we should, that you, you think are important for a survivor to make a decision?

ID: Hmm. I think you cover most of them.

GT: So most of them.

ID: Yeah.

GT: So for this part the, is information, is how is the amount of information?

ID: Oh. I like it, especially number six, the convenience.

GT: Oh, that one very important.

ID: Yeah. That is very important for me.

GT: Mm. So this part the information, you think is, is, is enough?

ID: Yeah.

GT: Okay. Then how about the clarity?

ID: Oh, yeah, it’s clear.

GT: Yeah. Then the presentation?

ID: I think good.

GT: Okay.

ID: I should answer this? No right?

GT: You can, you can also say your opinion if you want. You think you are leaning more towards, towards usual care, or you’re indifferent or you are leaning more towards shared care.

ID: Okay. Because right now right, I’m seeing the, the surgical consultant once a year and Dr Rose, Dr Rose last time two, two times, now is after ten years once a year right, so it’s two times a year.

GT: Yeah.

ID: So if I say I’m going to move into shared care, does that mean that I’m not going to see my surgical consultant anymore?

GT: I think you will still see them. But I don’t know how, not very sure how the, the frequency, because you have both sides right, surgical and the oncologist part

ID: Mm, yeah.

GT: The medical oncologist, so I’m not sure how that one will work out too.

ID: Yeah. I need to know that before I can make a decision. Because I still want to follow up with the surgical side also.

GT: Oh, okay. Why do you, why do you feel like, you want to follow up with the surgical side?

ID: Just in case. In case I have a relapse.

GT: Mm. Okay. Can.

GT: So this is the end of the decision aid. So now I’ll be asking you some questions first before we move on to the “Other resources” section. Yeah. So basically, basically right, how, how do you find this exercise help you to better understand your preferences in follow up care?

ID: Mm, yeah it’s good. I mean, last time I don’t, don’t really know about shared care right? I just, you ask me to go and see the polyclinic physician so I go and see.

GT: Oh. So this helps you more clear, more un, to understand clearer, clearer?

ID: Yeah.

GT: Okay. Then how, in what ways do you think that the, this preference exercise, that one you just, the one with the, the one with a lot of questions one right, how does it, how does the exercise, how is the exercise beneficial or helpful in decision-making?

ID: You mean the seven questions is it?

GT: Correct, the seven questions.

ID: Erm. Yeah, I mean, I, I mean you show the cost structure right?

GT: Mm.

ID: For, for usual care and for the shared care, and, I mean normally I won’t know. So

GT: Yeah.

ID: I mean, so it’s good because you start from the beginning right.

GT: Yeah.

ID: Then, erm, yeah, I think that all the questions are relevant.

GT: Okay, can. Then how do you find this, this decision aid aesthetically? Like, can comment on the appearance features like color schemes, the choice of the font, the font size.

ID: Erm. Erm. I think, I think the, if you keep it simple is better. So, so I think it’s good, because you only have one font size right, for all their, the wording. Yeah. Is, is clearer.

GT: Okay. Then how about the use of the icons, the images, the graphics and the interactive buttons?

ID: Oh, I like it.

GT: Is it easy to use?

ID: Yeah.

GT: Easy to use.

ID: Mm.

GT: So, in, so, so for these navigation buttons right, how, how do you think it helps you in your viewing, viewing experience?

ID: Erm. I mean, I won’t feel so bored. You know like, I can click something and I see something different.

GT: Mm. Okay, that’s good. And then, if let’s say we, we, we have a downloadable copy of this decision aid right, how, how do you, like, or to, to view, on your, on your electronic device right, how do you find this function?

ID: Which function?

GT: Like if we can download this copy for this decision aid to view, to view on an electronic device.

ID: Yeah.

GT: How do you find this, if, if, if there, if can, if you can have a downloadable copy? Like, what do you think about it? What do you think, if we have (repeated) the downloadable copy, then how, what do you think, or what do you find about it, like having this downloadable copy? Do you find that it is useful?

ID: I, I think, I think, it will, it will help me understand what is usual care and shared care right, better.

GT: Mm.

ID: Then, maybe I can make a decision after reading this presentation.

GT: Okay. Then what do you think about the length and time taken to go through the decision aid?

ID: How long did we take, to go through?

GT: I think it’s about forty minutes. Close to forty minutes.

ID: Shorter would be better.

GT: Okay. How short though?

ID: Twenty minutes?

GT: Twenty minutes. Okay. Then will you visit, revisit this decision aid for some of the information presented?

ID: Yeah.

GT: Which portions will you revisit again?

ID: Maybe if I’m not clear about the, what is shared care versus usual care, then I might visit that section again.

GT: Okay, can. So if, if given a chance right, will you use this decision aid to discuss follow up care with your oncologist?

ID: With the oncologist?

GT: So maybe like, what we are thinking of is that maybe this oncologist can give a link for you to, the oncologist can pass you the link, then you can just go, can read up on your own, or either there, then you can, you can discuss with your oncologist whether you want to take up shared care or remain in usual care or yeah.

ID: Oh, yeah.

GT: Okay.

ID: But I’m only seeing Dr Rose in December.

GT: Yeah, I know. Yeah. So, yeah can. So can move to the end, to the “Other Resources” section. Yeah, here you don’t have to, here each tab would give you different, each different topics, actually you can click on each tab and see, but you don’t have to click on the tab, tab itself. I mean, click the survivorship first.

ID: Isn’t this a summary of what we went through?

GT: This is actually the website resources.

ID: Oh.

GT: Yeah. But we’re not going to go through every website. You just see, help us to see the range of the topics.

ID: Mm.

GT: Yeah. So the, there’s the icon there that shows the country of the, where it came from.

ID: Mm, yeah.

GT: Yeah.

ID: Is this from like, mayo clinic, or something? Oh cancer . Good. Last time when I, when I found out I had breast cancer right, I read up on them.Is it why I have me right?

GT: Hmm?

ID: “Menopausal hormone therapy”. I’m not having hormone therapy right.

GT: Yeah, no.

ID: I think this one is, applies to

GT: Mm.

ID: To people who have

GT: Who did surgery.

ID: Yeah. Mastectomy.

GT: Yeah.

ID: And, you have, you do plastic surgery right.

GT: Mm.

ID: For me, doesn’t apply.

ID: Yeah. After my first, first session of chemo right,

GT: Mm.

ID: I went, I (repeated thrice) went into menopause already.

GT: Oh.

ID: Well, these are good.

GT: Okay.

ID: I want to check later. “Support groups” [?].

ID: Oh good. I like this.

GT: So for this “Other resources” section, how do you find about the amount of information?

ID: Oh good. I like it.

GT: Okay. Easy to understand?

ID: Yeah.

GT: Okay. How about the presentation? It’s like the, poor fair good or excellent?

ID: Erm. Excellent.

GT: Okay, thank you. Okay, so just one last question. Any other thoughts that come into your mind while you were viewing the decision aid that you haven’t shared?

ID: Any other?

GT: Yeah, any other thoughts that came into your mind while you were viewing the decision aid that you haven’t shared?

GT: If don’t have, it’s okay.

ID: No.

GT: No. Okay, then that’s all for the decision aid. Thank you so much.

ID: That’s it?

GT: Yeah. I will stop share first.
