# Supplementary material for: Development and pilot testing of a decision aid for navigating breast cancer survivorship care
Source: BMC Med Inform Decis Mak. 2022 Dec 15;22:330. doi: 10.1186/s12911-022-02056-5 (PMC9753367; doi:10.1186/s12911-022-02056-5)
Supplement: Supplementary file 5 — Additional file 5. Transcripts and the final decision aid prototype. [file 12911_2022_2056_MOESM5_ESM.zip › Additional file 5/ID09_transcript.docx]

ID09

**Study ID**: ID09

**Interviewer**: GT

**Date**: 9 Dec 2021

**Transcribed by**: IC

GT: Okay, so we’re going to go through this decision aid together, each page at a time. This decision aid consists of five key sections. As you’re viewing each page and section, tell me out loud any thoughts that go through your mind. I may also prompt you with some questions along the way as you navigate across the pages. So for instance, at the end of each section, I will be asking you about the amount of information, whether it’s too much, just right or too little, and the clarity of the information, whether it is easy to understand or is understandable with some ambiguity, or do you find it is difficult to understand. And lastly, will be the presentation, whether it is poor, fair, good or excellent. So if you’re ready, you can click on the, you can read this disclaimer and then

ID: Yeah.

GT: Click the “I have read and understood the above”

ID: Okay. Yep.

GT: So basically, you just read through and then just, yeah, if anything that you want to mention, you can mention, your thoughts.

ID: Do I have to tap the interactive bubble is it?

GT: Mm.

ID: Mm. I’m trying to tap “ongoing hormonal therapy”

GT: Oh this one cannot be tapped.

ID: Okay, alright.

GT: Yeah.

ID: That’s all?

GT: Yes. Then you can click on the bottom

ID: Where?

GT: The arrow, the right

ID: Oh

GT: Yeah

ID: Oh

GT: Okay, you can start off with the first section.

GT: Can click on the first section, “What is cancer survivorship”.

ID: Okay.

ID: Sorry [ID coughing intermittently]

GT: It’s okay.

ID: Okay, so for this

GT: Yeah, so for this section right, what do you think about the amount of information?

ID: Yeah, I think it’s okay

GT: Just right?

ID: Yeah.

GT: Okay. How about the clarity of the information? Do you find it is easy...

ID: It is good.

GT: Easy to understand?

ID: Yes.

GT: Okay. And how about the presentation-wise?

ID: Mm, I think it is good.

GT: Okay, can, then you can move to the next section.

ID: Mm, do I tap the arrow for next section?

GT: Yes.

ID: It doesn’t move. Or do I click to number 3?

GT: Number 3 is this slide already, so you have to click on the next section.

ID: But it doesn’t work.

GT: You try again? Yeah.

ID: Oh okay.

GT: Yes.

ID: Okay. So this part, do you need me to respond in any way?

GT: If you have any concerns about the language, of the terms that you’re not sure, you can voice it out.

ID: Oh. It’s more for language, it’s not sharing personal experience?

GT: You can also I mean, but not so relevant I think.

ID: Okay.

GT: Yeah, maybe if you have some side effects that you have but is not mentioned here, you can mention that.

ID: No, for me it’s, I’d like to share how to improve on the condition, it’s that you have to exercise always. So what I do is I use a exercise band and I wear a pair of gloves. So I will actually pull the rubber band this way, so it sort of like helps me in my posture and also not feel tightness in the shoulders and arms.

GT: Okay.

ID: Yeah, so it has improved and in fact there was actually numbness on my left side of the armpit, but it has gone. Yeah.

GT: Mm okay.

ID: So shall I go on?

GT: Yes.

ID: Where do I click now?

GT: You can click anywhere outside of that, just click outside, it will disappear.

ID: Okay.

ID: Okay. It is quite easy to understand.

GT: Okay.

ID: Okay.

GT: Mm.

ID: So this language is not familiar to me.

GT: You mean the medicine, the medical

ID: Yeah.

GT: The name of the medicine?

ID: Yeah.

ID: Okay. So for this information, it is quite easy to understand.

GT: Mm.

ID: Mm.

GT: Okay.

ID: So I move on?

GT: You just click, yeah.

GT: Okay. Then you can move on to the next section.

ID: Oh okay, I don’t have to click on this letrozole

GT: If you want to read you can do so.

ID: Okay.

GT: Okay. For the physical and emotional effects section right, how do you find about the amount of information?

ID: The previous slide is it?

GT: Mm, those physical side effects and all those.

ID: Yeah, should be okay, quite easy to understand.

GT: The amount of information is just right?

ID: Yeah.

GT: Okay. And then how is the presentation-wise?

ID: Mm, it’s quite good, clear.

GT: Okay, can. So we can continue with this section.

ID: Okay, so finished reading. Do I click on any of these bubbles?

GT: You can click on the number 2 and the button in the below.

ID: Oh.

ID: Okay. So I click to number 3?

GT: Mm.

ID: Okay, finish. On to number 3, or 4?

GT: Number 4.

ID: Okay. So number 5.

GT: Mm.

ID: Okay. Number 5?

GT: Mm. After that you can click “back to options”.

ID: Do I go to number 5 now?

GT: You’re at number 5 now.

ID: Oh, I’m at number 5.

GT: Yes.

ID: “Back to options”.

GT: Okay. So for this usual care section, do you find the amount of information is too much, is just right or

ID: No, I think just right.

GT: Just right. Then how do you find about the clarity of information, was it easy to understand?

ID: Yes.

GT: Okay. How about the presentation?

ID: Okay, good.

GT: Good. Okay, then you can click onto the shared care bubble.

ID: Mm. Okay finish. So do I go to number 2?

GT: Mm.

ID: Mm. Number 3?

GT: Yes.

ID: Mm, okay.

ID: Okay, finish. Go to number 5?

GT: Yes.

ID: Cannot. Should I press the forward arrow?

GT: Can. You try clicking now? You go back to number 5?

ID: This number 5 right?

GT: Yes.

ID: Mm. Okay.

GT: Okay. So for this shared care portion, how do you find about the amount of information?

ID: Mm, so actually, the shared care portion, the information also will be available to the three parties. Then, at which point will this oncologist be involved actually?

GT: The oncologist is still seeing patient at the moment, it’s just that the care is shared between the different healthcare professionals. So if there’s anything that, let’s say the polyclinic doctor finds some abnormality, then they can always escalate back to the oncologist and refer back to see the oncologist.

ID: Mm. Yeah, okay. So this part “do not worry if this happens. It does not mean that a cancer has returned”. Oh okay. Maybe just that, there has been like, there is a need for further investigation, correct?

GT: Yes, correct.

ID: Yeah, okay. Can, understand.

GT: So the amount of information do you find is too much, just right, or too little?

ID: I think it’s okay.

GT: Just right.

ID: Mm.

GT: How about the clarity of the information?

ID: Mm okay, I think it should be okay.

GT: Easy to understand?

ID: Yeah.

GT: How about the presentation?

ID: Should be okay.

GT: Good? Poor, fair, good or excellent?

ID: I think it’s good.

GT: So after this you can click “back to options”.

ID: Doesn’t work.

GT: Doesn’t work. Hmm. I think your cursor has to go to the, yeah. Back here. So after this you can click on the next section.

ID: What do you want me, oh, next section.

GT: Yes.

ID: Doesn’t work.

GT: Your mouse has to appear. You need to bring back your mouse.

ID: I’m using the mouse pad.

GT: Using the mouse pad.

ID: Are you able to forward for me?

GT: Can, but ideally we want you to try to do on your own.

ID: Okay.

GT: You see later if you can press.

ID: Okay. “Comparing the options”.

GT: Mm.

ID: “Communication between healthcare professionals”

ID: Mm okay. Okay got it. So go to number 2?

GT: Mm. So it’s either the number 2 or the next arrow button. Both of them can be used.

ID: Okay, got it.

GT: So for this section “comparing the options”, how do you find about the amount of information?

ID: Good.

GT: Okay. How about the clarity?

ID: Good.

GT: Then the presentation?

ID: Good.

GT: Okay. So we’re done with this section.

ID: Yep. Next section?

GT: Mm.

ID: What do you mean? Why? Oh. “How do you feel about the cafeteria” is just an example?

GT: Correct. Basically to see which side you lean more to, whether usual care, shared care, or you’re indifferent.

ID: “Reason for shared care”, “reason for usual care”. Oh. Mm. When you say “reason for shared care”, “reason for usual care”, maybe it’s a little bit ambiguous?

GT: So what do you think should be put there instead?

ID: How about, decision for shared care?

GT: Mm. Yeah, that makes sense.

ID: Yeah.

GT: Yeah.

ID: So, yeah.

GT: Okay, can. We will take that into consideration.

ID: Because you are asking all this questions and showing all the information with the purpose of ask, checking the decision of the patient. So I think the word “decision” should be better.

GT: Mm. True, true.

ID: Yeah. Okay. So next section?

GT: Yes. So over here, you can just help us to see if, let’s say, these factors are relevant to help the cancer survivor make a decision about the follow-up care. You don’t necessarily need to answer the questions. You just see if the questions are relevant.

ID: “How confident will you be in a family physician that has been trained ?” If it’s trained, then should be confident.

ID: Okay. “How comfortable are you with seeing this trained doctor for your cancer care?” Yeah, I think it should be okay. The question should be okay.

GT: Okay.

ID: So I forward?

GT: Yes.

ID: “How useful will a service like care” Okay. I think should be very good, the questions.

GT: Okay.

ID: Like for me, I don’t take any more medications, so it doesn’t matter, so I will answer number 3. So for somebody who is still on medication, probably they will, answer number 5. Yeah.

GT: Okay.

ID: I mean, this part about the pharmacist being involved will be usually relevant to people who are still on medication, for cancer treatment, correct? Nothing to do with like normal kind of medicine, like hypertension?

GT: Yeah, the pharmacist can also recommend other lifestyle advice. Like other than medication advice, they can also talk about lifestyle changes and all that, so they can recommend in that aspect as well.

ID: Yeah, but they will not advise on other medication outside of cancer treatment right?

GT: I think it’s a general one, like overall, apart from cancer, also the other chronic conditions as well.

ID: Okay. So next.

GT: Mm.

ID: Mm. Okay, next.

ID: Mm. Okay, good.

GT: So basically, do you find these questions useful to help survivor make a decision?

ID: Yeah, sure.

GT: Do you feel that there are other factors that you think a survivor might consider, apart from what we have mentioned here?

ID: I think for this case, personally, I would feel that the gender of the doctor is important because I will relate better with a female doctor.

GT: Mm. But it could also be a personal preference thing also.

ID: Yeah.

GT: I see.

ID: So that’s it? That’s all?

GT: That’s all? So for this section, how do you find about the amount of information?

ID: Okay, just good.

GT: And then the clarity?

ID: Good.

GT: And the presentation?

ID: Good.

GT: Okay, can. So you can click on the next section.

ID: So all these questions will be asked, I mean, you will be asking these questions to the cancer survivors online like this or hardcopy?

GT: We can do in different ways, can either be a downloadable copy kind of thing or it could be an online kind of thing, maybe their oncologist can give them an online link to go and see the decision aid. Maybe they can also discuss with their oncologist to see whether they prefer to stick to usual care or they want to try shared care.

ID: Yeah, okay.

GT: Okay, this is the end of the decision aid. So, overall, how do you find this decision aid?

ID: The whole set of questions is considered the decision aid?

GT: Mm. Along with the information as well.

ID: Okay, yeah. It’s clear, it’s good.

GT: Okay. So you can click on the other resources. You can click that first. Basically there are different sections, you can just help us see if the topics are sufficient and relevant. Not necessarily need to click on them, but just click to see each tab.

GT: Yeah, so you don’t have to click because this will bring you out of the slides.

ID: Okay. “More than just surviving cancer”. Okay. Next, I just?

GT: Yeah, you can click “Back to Resources”. You can go to the second one, “Effects of treatment”.

ID: Mm.

GT: You’re too fast.

GT: Okay I click for you.

ID: Mm. Okay.

ID: Can you click for me?

GT: Okay.

ID: Mm. Okay. “Back to resources”

GT: Mm.

ID: Mm. Okay.

GT: Yeah, so for this “Other resources” section, how do you find about the amount of information?

ID: I think it is excellent, yeah.

GT: Okay. How about the clarity?

ID: Yes, good.

GT: Then the presentation?

ID: Okay, good.

GT: Okay sure. Just like to ask you a few more questions before we end the interview. So basically, how do you find this exercise help you better understand your preferences in follow-up care?

ID: Yes, definitely.

GT: How does it help you better understand?

ID: To help me understand that with more health workers involved, yeah, there will be better assurance, yeah, better assurance for the patient. And I think most important is being able to see the family physician periodically will also have better assurance. Usually for follow-up after 3 years is usually 1 year appointment with the oncologist. Yeah. So to be to able to see the family physician more frequently, I think is a good thing.

GT: Okay, can. So then, in what ways you think this exercise is helpful or is beneficial in decision making?

ID: I think this exercise is good in the sense that especially for those who are, I don’t know whether you have a Chinese version, do you?

GT: Oh Chinese, yeah currently we just have the English version for now.

ID: Yeah. So the main, yeah the English-educated will actually find it easy to navigate on their own, so you don’t need to have a person to keep explaining everything to the person.

GT: Mm.

ID: Yeah.

GT: Okay. Then how did you find this decision aid aesthetically? Like you can comment on the appearance features, like the color scheme, choice of font, font size.

ID: Okay. I think should be good. If it’s on the computer, should be good. Will it be available on the smartphone?

GT: Smartphone-wise, I think should be available because it’ll be on a website format, so it should be viewable on the phone as well.

ID: Okay.

GT: Okay. So, how about the use of icons or the interactive buttons?

ID: Yeah, okay, that’s good. It’s excellent.

GT: Okay. Then for the navigation-wise, how did you find navigating through this decision aid?

ID: I think it’s good. It’s easy to do. It is easy to navigate.

GT: Okay. Do you find it confusing sometimes?

ID: No, not at all. But of course if I’m not using a mouse, I’m using my this, you know right, that if I’m using my keyboard pad, it’s a bit difficult sometimes.

GT: The control portion wise.

ID: Yeah.

GT: Can, okay. Then, what do you think about the length and the time taken to go through this decision aid?

ID: The length of time? We took, like you said, 40 minutes right, which is quite accurate.

GT: Yeah.

ID: Yeah. Am I considered slow in reading and understanding?

GT: No, you are okay.

ID: Yeah. So, for me, because I’m quite used to doing presentations using powerpoint so I’m quite familiar. You know, so I actually do a lot of reading quite fast, so I don’t think to me that’s not an issue.

GT: Then will you revisit this decision aid for some of the information that was presented?

ID: Yes, especially for the one about other resources, I think it’s very useful.

GT: Okay, can. Then, if given a chance, will you use this decision aid to discuss follow-up care with your oncologist?

ID: I don’t understand your question.

GT: Will you use this aid to discuss about follow up care with your oncologist? That means like, to talk with your oncologist to make a decision about the followup care?

ID: Oh, I see. Hmm. It all depends, I think, it all depends on what come first. Usually, if you are letting the oncologist to speak to the patient, then after that the patient goes into this website to look at the questionnaire, I think it better prepares the patient. Yeah, so, but if you were to say, contact your patients and ask them to go into this website, so they won’t know head or tail about it. So, may not be easy for them to make a decision straightaway, you know. I think people still need to have a personal touch you know, to talk to the oncologist and before they make a decision, to have the assurance that they will still receive specialist care.

GT: Can. Then, any other thoughts that come to your mind when you are viewing the decision aid that you haven’t shared?

ID: No, I don’t think so.

GT: Okay, can. So, any questions that you have for me?

ID: When will this happen, this decision, the shared care?

GT: So it’s basically after the survivor has completed at least 3 years out of their treatment, then they can

ID: Oh yea, I know. I mean, when will this be launched?

GT: This one, not sure yet.

ID: Oh.

GT: Because we might take some time. Yeah. But actually because currently we are doing a project to, we are trialing out already the project, so some of the participants are in the shared care program. So we are actually trialing out to see if it is beneficial, and if it is helpful, we might consider implementing it on a wider scale, yeah, across the whole of Singapore, and not just NCC.

ID: Okay.

GT: Okay, can. If that’s all, anything else to share?

ID: Nothing.

GT: Can, then I will stop share first, and the recording as well.
