# Supplementary material for: Development and pilot testing of a decision aid for navigating breast cancer survivorship care
Source: BMC Med Inform Decis Mak. 2022 Dec 15;22:330. doi: 10.1186/s12911-022-02056-5 (PMC9753367; doi:10.1186/s12911-022-02056-5)
Supplement: Supplementary file 5 — Additional file 5. Transcripts and the final decision aid prototype. [file 12911_2022_2056_MOESM5_ESM.zip › Additional file 5/ID06_transcript.docx]

**Study ID: ID06 Date: 31/1/19**

**Interviewer(s): ET & KY**

**PART 1**

ID: I think this one should be “the available options currently in Singapore”

ID: how do I go on?

ET: You can click the next page here. Do you think it will be better if there is the word “Next”?

ID: yes, because I didn’t even observe this. Or you can have something (points to bottom right of screen above bar) and there’s the word next you can click. Because I didn’t realize this has an arrow.

ID: how do I go back to the other page?

ET: that’s the home page, so you can just click the house.

ET: So just now that was the first section - do you feel that it is comprehensive? Do you understand you are reading?

ID: It was quite simple.

ET: And do you feel like it was too little or too much information, or was it just right for you to understand the importance of cancer survivorship?

ID: I think it was alright.

ET: Did you like the colours and the graphics?

ID: I think it was quite ok.

ID: maybe you need to explain about what body image concerns mean, cause body image concerns is very vague.

ET: Are there any of the terms that are hard to understand?

ID: I think for layman, they won’t understand what is “range of motion”. Lymphedema, maybe you can put “swelling at arm / breast” then bracket lymphedema. (surgery)

ID: “skin discolouration”, I think you need to put in a bit more layman term, cause for those that study until only primary school, they may not know what is discolouration. (radiotherapy)

ID: this one means there is a possible increase right?

ET: Yes, and the probability is over here.

ID: Maybe for those with possible increase you need to put as “possible” because for this one it seems definite that there will be increase. (cancer risk). For these two I think there is a possibility.

ID: My suggestion is maybe you want to put some.. like this you put statistics but how about this and this? To the patient they will say, this is going to increase my risk of heart disease, so am I going to get it? So they may be concerned that they will get this heart disease or soft tissue cancer in future.

ET: So in general, most of the side effects here there is a very low chance of getting it. But the statistics are not well-studied. That’s why we couldn’t quote anything here.

ID: Then in that case, you may want to at least mention that it’s “low” or you put it at the back, like separate the “usual” side effects and the “rare” side effects.

ID: is this also considered rare? (HER2+ SE)

ET: It is the currently only known side effect of the drug

ID: Or you can say “may” have an increase cause at least it’s not a definite thing.

ID: for layman I think they won’t understand the word drug, drug to them is a different thing.

ET: So medicine?

ID: ya medicine.

ET: So just now for that section, the main focus was on the risks. So your main concern was whether or not we can make it sound less definite right?

ID: Yeah. Maybe you should use words like “possible” or “may” and for the medical terms, you should use the laymen terms before the medical terms.

ID: you use “return of cancer”, but over here you use “cancer relapse”. So do you all think that cancer relapse is more acceptable?

ET: Initially we put cancer relapse, but some doctors brought up the concern that survivors may not understand the word relapse. So maybe we’ve overlooked this part.

ID: It should be consistent, as long as it’s consistent.

ID: My suggestion is.. because “be it a family member” it sounds… maybe you can put it this way “it is important for you to have someone (a family member, friend, counsellor) whom you can turn to for emotional support”. Because I feel that this person is not an “it”.

ID: what do you mean by “different doctors” (follow up care options), different doctors of different field, different specialty?

ET: So currently they will only see an oncologist for cancer related stuff, but in this case in the shared care model they will not only see an oncologist but also family physicians and nurses.

ID: Then maybe you want to say “shared among group healthcare professionals?” because it can be doctors, it can be pharmacists, and because different doctors is like I jump from one doctor to another, then its like… since you mention it’s a group of healthcare professionals it can include like pharmacists, oncologists and family doctors, so you can specify so at least the patient will feel like the information is shared among this group of people.

ID: this one sound very funny “why are there options?”…

ET: maybe the phrasing is it?

ID: Yeah.. maybe you don’t even need to have this, just the introduction then what are your options because you’re just trying to explain why we want to set up this follow-up care.

**PART 2**

ID: this one actually it has a back to options whereas the other one where you have the five icons (side effect) you have a cross. Maybe you want to make it consistent? Because when you have a popup, either you use a cross or you have a back to options.

ET: Because for the other ones, the popup is just one page but for this one you have to go through 5 pages, so you can go back at any time.

ID: Oh actually when I see this I didn’t realise when I see the 12345 that these are the pages, unless you mention like “next, to see”

ET: so do you think at the introduction there should be like a guide to explain how to use this navigation tool?

ID: Yeah… I think because more people are not used to having this over here, we’re more used to “next” “back” at the sides.

**PART 3**

ID: the purpose of this page is to mention to the people to go to primary care doctor right?

ET: no, for usual care, we are trying to draw a picture of what current care is like, and what roles each of the doctors play currently, and then for shared care it’s what roles those same doctors play if they come into the shared care arm. So in the previous version of the decision aid we only talked about the oncologists, then many people gave feedback saying that they don’t really know what goes on and some of them don’t really visit a polyclinic at all. So here we try to draw out the bigger picture of both sides so they can make a clear comparison.

ID: it sounds like the community pharmacist only do this, like they’re not involved in other.. like they won’t only see symptoms for fever and runny nose.

ET: So sometimes people do bring their prescriptions and they can dispense those drugs as well, but the general consensus is that we do look for them for simple symptoms like fever and runny nose. Maybe we can add in “for example” so they won’t feel like it’s restricted to this.

ID: Yeah because it sounds like they are only restricted to only fever and runny nose… and I think the community pharmacists may protest.. maybe you can mention that they fill up prescriptions and give recommendations on appropriate recommendations and also give health advice (in a sentence) so that it doesn’t sound like they only…

ID: oh I lost, where am I supposed to go next (lost at options page after reading usual care)

ID: so everybody is seeing surgical in march..? (usual care timeline)

ET: Oh no, this is just to show you a rough estimation of this 12 month period, you will see them around 6 months apart. So it doesn’t have to be March. Maybe they can meet in June and they will see them again in December, or July and they can meet again in January. But there’s always a 6 month gap, because it’s twice a year.

ID: Because to me when I see this, I will see surgical every 3^rd^ month, 3 is March and 9 is September.

ET: so if you read the words without seeing the picture, do you think you can understand?

ID: The word here doesn’t mention about the alternating between the surgical or medical… you only mention you will see an oncologist, so to me I will feel that “ok, that means every .. a year, I will see an oncologist, but I won’t know whether it was alternating”

ID: I think without the guidance, I won’t know to go to shared care.

ET: Ok so maybe instead of asking them to choose to go into shared care / usual care, just make it flow?

ID: cause once they go in and when they come out, they will be lost already, especially if they are not computer literate…

ID: so over here they won’t see the surgical oncologist?

ET: Yeah, for shared care.

ID: Meaning that they will contact the survivors after the medical appointment?

ET: It’s to follow up on them on how did their appointment go, was everything clear, did they not understand anything the doctor said, and just to check up on their general well-being.

ID: this sentence sounds weird… “equipped with the medical knowledge to look out for cancer survivors” meaning? To look out for what? (care navigator) so why do they need medical knowledge to look out for this person?

ET: Maybe we will add it in in the future so that it’s clearer. It’s to look out for warning signs, symptoms of the return of cancer, and any medication that’s not suitable for the survivors. They’re doing like a surveillance role. Not sure if the word surveillance is comprehensive?

ID: surveillance is not suitable (for understanding), you all have to rephrase.

ID: maybe you want to mention, at interval or 6 months…

ET: Actually it is not strictly 6 months, most of the time it is, but sometimes people cancel on their appointment, or come in earlier or later.

ID: but generally it is?

ET: Yes, generally 6 months apart.

ID: this one means you want to refer back to the oncologist right? (back to the oncologist)

ET: Because now they are alternating between healthcare professionals, when the other healthcare professionals see that there is an emergency, they will contact the oncologist to do a more thorough check. So it does go back to the oncologist in a way.

Sp; Yeah but this one and this page doesn’t match.

ET: So you feel that they are separate information is it?

ID: yes!

ET: Because for this page we are saying more on how the information is being relayed, if there is something wrong it is brought to the attention of the oncologist. Maybe the title…

ID: Yeah the title is a bit… Maybe you can mention something about the information is actually… refer back to oncologist? Or something like that because I think the title is a bit…

**PART 4**

ET: So this part they will go through a list of questions for them to come to understand whether they are more towards shared care or usual care or whether they are indifferent. After they complete the answer sheet they can bring it to their oncologist to discuss more on which option they will take. So we can go through to see whether these are relevant questions that a survivor would ask in their decision making. The questions are all here, this is just the online version. Maybe you can take a look through to see if you find them relevant.

ET: Do you feel like any of the questions were not easy to understand or irrelevant? Or what other questions can we include to make it more helpful in decision making?

ID: maybe this one, “how confident will you be” because they haven’t even met the physician so how confident can you be? I mean you can still ask, but I haven’t met the person so how do I know whether I can be confident or not? You can arrange somewhere [inaudible] for me, but at the end I may not be comfortable.

ET: We do want to see whether they are comfortable going over, but at the same time people have their concerns going from a specialist to a general clinic, which they feel is a downgrade (but it’s not true), they may not feel comfortable as they think that the doctor is less well trained than their oncologist. So we are trying to find out if we tell them that the doctors have been trained by an oncologist and equipped, whether they are ok with it.

ID: think I don’t have any other comments on this.

ET: Ok, so at the end of this section, there is the conclusion slide.

ID: so people are going to go online to do this or do here?

ET: For future use, when we roll out the decision aid, we will put it online for people to test. And we’ll put it here when we inform people about the new options.
