# Supplementary material for: Development and pilot testing of a decision aid for navigating breast cancer survivorship care
Source: BMC Med Inform Decis Mak. 2022 Dec 15;22:330. doi: 10.1186/s12911-022-02056-5 (PMC9753367; doi:10.1186/s12911-022-02056-5)
Supplement: Supplementary file 5 — Additional file 5. Transcripts and the final decision aid prototype. [file 12911_2022_2056_MOESM5_ESM.zip › Additional file 5/ID01 - Transcript.docx]

**Study ID: ID01 Date: 25/10/19**

**Interviewer(s): ET & KY**

ET: is the screen too dark

ID: it’s ok

ET: can you understand all the terms? (different cancer)

ID: ya, I understand

ET: you can click on this also (referring to treatment slide)

ID: you must give instructions because just now that one the instructions have, like this one, i wouldn’t know

ET: do you know what is AI

ID: ya… the…I thought its 1 2 what is AI

ET: it is a class of drug, aromatase inhibitor. So I should spell out for you right?

ID: ya.. because for layman they don’t need to know the class of drug. So for them, if they are interested if like the long term effect of late effect like this

ET: but you will recognize which drug you are using is it?

ID: For that, tamoxifen, I having all these thing, then after that I on letrozole. So if the person who having such drug, they only relate, like for me like when I read I only relate to this (the effects)

ET: So I don’t need to put AI?

ID: no need because if you want to tell the class of drug, Its your target population. If your target population is layman, they don’t need to know. But they need to know all this thing, relate la

ID: Eh? Finish ah? (for treatments effects)

ET: haven’t, last one

ET: So maybe this one not relatable to you

ID: no, I on Herceptin. Ah so when you are target therapy… for me targeted therapy... so this is hormonal right?

KY: Herceptin… considered, it is for HER2+

ID: correct correct HER2. So in the sense that if they can explain what is targeted therapy. Because people can understand chemo, radio right, hormone. But target therapy.. targeted to what? Layman they don’t know what is targeted. Like all these things (the other treatments) people can understand ah but target…?

ET: you don’t know what is that

KY: If we put HER2 ah can recognize?

ID: Ah yes yes, we can recognize because the doctor usually when they explains to us “because you are HER2+, that’s why the drug targeted to this” so if not it won’t… understand why I need to have targeted therapy.

ID: Correct… (emotional slide)

--- skip acceptability qns ---

ID: so this is 1… now im doing 2?

ET: yes, now you are doing 2

ET: so for this case right, like the shared care is in the future, there’s no option yet but we want to put the information inside for the future… so we give you information about what the shared-care is…

ID: ok this primary care physician in hospital…? Or you meant GP… I mean polyclinic doctors? What is your primary care physician meant? The hospital doctors uh?

KY: no… polyclinic or GPs.

ID: ah polyclinic or GPs

KY: this part is it not very clear?

ID: ah because you see, ya correct because the primary care physician… see for me I also have hospital appt. I haven’t go polyclinic, I go GP, polyclinic very long. Because the GP itself don’t have the NEHR or sharing of information. That for me is my problem so every time I need to explain my medication, I need to explain my blood sugar all that. That’s why just now that doctor ask me its good that if I can go polyclinic. Because easier transfer but I didn’t go but I intend to go but problem is that the timing la. The timing is… ya very long queue. I need to ask you how do I make appt if I wanna go polyclinic?

KY: you can get Dr Rose to write saying that you are suitable for their polyclinic care bag(?) so that there is a referral or if you walk… first time you have to go to a polyclinic… have you ever been to a polyclinic?

ID: no not yet

KY: so choose one that is nearby, you go there then you can actually… the first time you need to walk-in and then register yourself as a patient so they recognize you and can get your data, get you inside their datebase and then subsequently you can make appt online

ID: ok that means I don’t have to inform them about all this things a, you all automatically.. in the system.. that means say like.. for your study..for example, I just register, you all will.. can have available information through…

KY: er so if its for example things like your… those that in NEHR, definitely the polyclinics can see but I suspect it is still within the cluster. So like you are going singhealth polyclinics, then I think they can access the NCCS side notes and everything but im not too sure about whether you can cross cluster cause sometimes you can go to like NHG polyclinics.

ID: ya mine is… east side. I will go marine parade polyclinic. I don’t know whether singhealth or NHG

KY: most likely east is not NHG.. so most likely is singhealth, you just check with them again. But usually within the same cluster, can see each other’s notes but extend out can only see NEHR

ID: im ok. Because my concern is that you all get the information. Not NHG you all will have big problem

KY: ya, so that’s something I think all the clusters are still trying to work out. So in this case, is the primary care term… does it make you a bit not sure

ID: ya, im not sure in the sense. If you were to write example, such as polyclinic doctor then in the sense, better understanding..

ID: ok, in the sense pharmacist navigator… how in the sense they access..?

ET: access.. the records..?

ID: ya… no no no in the sense because you say I available to ask all these related right, how do I get in contact with the pharmacist navigator since you have this information here. Because you say remind, make schedule appt. so that means with that I assume its sms ah. They will say oh you have appt

ET: for this right, its something that we haven’t run yet, like it’s a shared-case. So the pharmacist is actually involved in the study then they will call you up and check on you cause they will have the details on when you went for your appt and when is your next appt. so they will just like “Oh I noticed you went for your appt with your oncologist on this day, how did the appt go… was it smooth?

ID: ok but then, if say like, the pharmacist call me, so I wont remember certain things, suddenly I remember certain thing eh why I having say blurry eye? Is it side effect of medication? I taking hormone targeted, then how do I have that query? I call? Because call usually people don’t give you the number what? What’s the helpline? Because you say it will be available for you to ask health-related questions, ensure and maintain a healthy lifestyle, so this the.. what platform in the sense?

KY: so do you think if it’s a number for you to call during office hours, it will be…?

ID: ya, because some people.. suddenly something happen, they panicky, they will like, most of the time people will (be) blaming. I blame on the medication, but they wont blame on whether its their lifestyle or new tumor or cancer set it, they would not. So they will,.. “oh this medication… because I just take the medication” so that’s the first thing .. they will pinpoint..

KY: I think we can make it more obvious as to how we can establish a contact.

ID: Correct so if they were to call the pharmacist, they say, “oh this is…” “you having it (side effect) but im not sure because this will be the side effect. Generally patients who take this medication do not experience this. Maybe the next step you say, why don’t you… because I don’t think so from this medication.” So in the sense I cannot point on the medication already so next one see doctor la, better…

KY: so maybe they will ask more of the “whats the symptoms like? When’s the onset” that kind of thing

ID: ya ah because it can be like.. I really very stomach pain is it medication… then maybe they can say like ya this side effect, did you take your medication before food or after food. Then I realise I take my medication wrongly. It should be before food or after then they will be like “oh no wonder I keep on having this stomach ache” which is like no need to see doctor la, just change the timing of you (medication).. ya.

ET: is this table clear (role)

ID: ya

ET: is it useful to help you understand?

ID: it is good, so at least you know you are not out of radar, somebody somewhere will help you there. Because for me I always this one forgot forgot (appt reminders). So I really rely on appt reminders. Say like example they give me the form but I forgot to put on my calendar. But the appt reminder come then I will quickly tell my boss. Eh I got appt then he say wah I say I forgot la but at least they will tell 1 week in advance so not so bad, I can plan my schedule..

ID: so I will… link live? (cost of appt table)

ET: ya so actually its suppose to link to the website but to help you see better we put it in this format. Do you think this is easier to view or the website?

ID: good good, ya because they need to prepare money… cause especially for cancer survivor.. so 6monthly not so bad but if your follow up one year, cannot remember how much do I pay last time. I just shared with my.. just now at the counter the pharmacist and then I need to pay the medication. Of course got insurance cover my certain medication so I have to pay 76$. So I say wah 76 not cheap ah then she say but this is about one time 3 to 6month medication. I say this is not about that. 76, a one time pay. Some people one day the salary don’t earn until 76, it can be 50$ of lower. So ah to one time take out 76 ah, even if you say medication 3month or 6 month, its about the money in that moment. So this thing is good. At least they know im going (for appt), better prepare. Cause for us, sometimes different, it can be consultant, can be senior consultant.

ET: But if you are a Singapore citizen it’s the same rate

ID: ya correct, cause this one for me subsidized, I pay 36 but don’t forget just now the pharmacy after subsidized I pay 76. so 76 and 36, then I haven’t taxi fare and I haven’t makan money, you get it not? so if the pay ah.. not say one day pay, its like 2 3 days pay.

ET: This is the same because its for oncologist (pricing), this is the polyclinic rate

ID: ah ok. I also don’t know this is the rate… wah now go up already ah?… aiya this one average ah, but sometime more. Its about 30 over dollars excluding medication. So this one is consultation, it depends, short consultation then you longer half an hour it will be more.

ET: oh its by per session ya,

ID: per session, it depends on the thing, no more 24, this one about 30$ already. I don’t know my house also.. 30$. Last time was 24, now no more…

ET: can you understand the funding part?

ID: Can, ya, ok.

ID: so… this one.. it will be on the website right? This thing and the thing.. information right. What im trying to say because you see ah, because you wanna save space, when this thing meet this thing, when we read (the words too close), suddenly “your coordination” then we read through “for your care coordination through”. Cause you don’t even have full stop so I tend to read all the way. (table alignment and spacing)

ET: ok so have more spacing or a line at least

ID: correct like you in line no matter how you cannot come out, like say this is your line right? cause you see this is already come out, you get anot? You already out of it.. better.. may they wanna save space but it don’t help for we all when we read we tend to read there… just the layout la..

ID: Eh? End of step 2 already? How come go there ah?

ET: I think last night I forgot to press the link back

ID: OK I at end of step 2 right?

--- skip acceptability qns ---

ET: Ok this is for you to write your answers. (step 3)

ID: oh ok can

ET: it is for you to see which care model you prefer.. so its more of an interactive section. But because now its on ppt you cannot click the buttons but when its on website you can

ET: so for this line right, actually it’s a drag bar, so the more you are closer to this, the more important you feel it is, then the closer you are to here, the least important you feel it is

ID: its very difficult to gauge that. Its good to put numbers say like example 1 to 5, 1 to 3, 1 to 10. Then its easy. Because if like that if I gauge in the middle.. so what scoring do you..,. you get or not? what scoring am I looking at?

ET: so you’d prefer if there are actual numbers that ..

ID: ya actual number easy for them. Cause like.. especially some people who are fickle. Some people good / poor at scoring so its good that you have numbers.

ET: but our next few question is all like that because we thought it will be easier to drag the bar.

ID: so for me.. say I .. so that means you see this important, so like between that to that..

ET: ok maybe from now we give you from 0 to 5 since you want numbers

ID: ok 0 to 5 can. so will be like this is qns 2, your Likert scale 0 to 5… from 1 to 5 or 0 to 5?

ET: 1 to 5

ID: ok… -- answering qns— Qns 4 also very important, cause need to prepare money especially the timing, there’s no payday, people don’t put aside the money…

ID: I’m not sure, in the sense polyclinic also the same. So if you have subsidy, does it apply to polyclinic. Because I have insurance on certain medication, hospital they will, but I do not know whether will extend to polyclinic.

KY: this one will depend on your own insurance scheme. Sometimes they may put only specialist, SOC, then those that are like…

ID: usually polyclinic they don’t, like for mine, medication another 76$. That one they cover by my insurance but it has to be hospital, ya

KY: I think because like all your other like letrozole everything, only NCC have, polyclinic they don’t dispense also.

ID: for me, idk, my location probably very important because it has to be convenient for me from here I go to work easier. Later I go back. But if I go my polyclinic, marine parade, to go to my office will be a distance, then not easy to get cab also…

ID: so qns 7 right, qns7 you didn’t put the number so my answer is this… so how do i…?

ET: I help you write – less for polyclinic

ID: this is good, got people sayang you. Why I say that is because most of the time, you already post chemo, slowly slowly, the oncologist slowly let you go, another oncologist but those like long term oversee type, you already detach from that other oncologist then you feel people dumping you. You already build relationship with one another, then this oncologist you haven’t build relationship. Especially you are not paying class(?), then you see different different doctor. Relationship-wise, bonding not there, not very nice for us ah, you feel you already have that… not low esteem, you already feel you are not who you are last time, my voice not so heard for instance. So when all these event come in you feel aiya not important, keep pushing, like you know my cousin say wah so concern must have radiotherapy must chemo, wah after that lesser and lesser, people like clear away, like don’t want to see you. Initially I have that problem, after that I accept la.

ET: You know what is the care coordination about?

ID: Care coordination for me is this, the navigator coordinate, especially like just now since you ask question, whether is side effects of medication, oh you better see your doctor gp or whatever.

KY: also like, all your doctors kind of know each other also, so at least I know someone is taking care of you

ET: So you’ve answered all the questions already right, do you feel that you are leaning towards currently what you are going through now, or do you feel that if the shared care is something for you then you would prefer it?

ID: ok, the usual care is good when you’re in early stage of treatment. Shared-case is.. in the sense, if the cancer survivor is… can say after 5 years, shared-care is important. Because after 5 years people will see you are cancer free. When you are cancer free, we don’t want to burden the doctor. The usual care. Don’t… wah… so its good for you to have the shared-case because you don’t need the immediate… the usual care the immediate attention all these things, so you already surpass that then you move on. Because the shared-care will be like other thing very important. The lifestyle, doctor won’t go and follow up with you so like polyclinic or yeah… so that’s the thing. It depends on which stage are you in la..

ET: Yes, correct, so for yourself personally? Which one would you prefer?

ID: for me now is shared-care la cause mine already I surpass the 5years right? But of course, I still have the side effect until now, its like I like to share so that my… people who are the early stage (for) example will benefit. That’s why I told Dr **** about my initial, when I have problems sexuality.. these things I shouldn’t really go through if its like.. people aware. Especially when example, all these information pamphlets, like you don’t want people to talk at least something they can look on. For me that time I refer to many on chemotherapy. I only refer to do not get pregnant. Do not get pregnant when you on chemo, only that.. so we don’t really see. Because do not get pregnant doesn’t mean.. you never say no fertility or whatever, so its not clear.

--- skip acceptability qns ---

ET: so for the qns its just, you want a likert scale?

ID: ya better la because very difficult, in Singapore, we in the sense, very molded by numbers from small, so when we don’t see numbers, very hard to read. Same as older people, maybe they don’t go to school all that but they know that 10$ note very important, like 2$, 5$ like that la, so numbers also.

KY: ok so maybe, do you feel that this kind of exercise right, cause you know that maybe some survivors, they don’t really… like for example certain questions they may be very adverse towards, some of them want to maintain multiple doctors, so do you think that this exercise is good like the…

ID: this exercise is good.. in the sense then its also, you see people different. Some people they cannot make decision, they don’t know what they want. So in the sense like its ok la, but its good for them especially have many people, different. Cause sometimes you bored that doctor the same thing the same thing, doctor already explain. So sometimes they want to hear second opinion? Not say second opinion but other people say, eh you see ah, you see like that. Actually they have other things, some is like that. But if they keep on having the same, everybody, because you all having all the information right, the same? Everybody same tune then in the end the person aiya, better not, the same la, might as well don’t need. Ah some is like that la, they denial.

KY: any other… like we asked a few questions right? What other questions do you think may be relevant? Like certain things you think that maybe… like a preference for certain attributes or preference that will make you want to go to like shared-care or prefer either one?

ID: that’s why if I have something that I feel “wah, this is not good”, I would rather go back to my oncologist. Like maybe, I’m not sure whether it come back, a new cancer or what, then you feel the same area or other area, I wouldn’t waste my time, because this is weird because you are not the contact expert. I wouldn’t ask a pharmacist that “eh, I have this lump, blah blah” I wouldn’t. because I do not want… because for me if I go to my GP (I will ask) the same question, so I directly want a referral to my oncologist back. Because I know you are the contact expert I wouldn’t want to waste time, so you advice me what I’m supposed to do. So to me is like I describe the steps rather than.. anxiety don’t forget about anxiety, don’t forget wasting my time, wasting money, because all these things not free, especially my primary care. Polyclinic can be wasting time wasting money, in the end will refer me to my oncologist so might as well I directly go.

- next step -

ET: So for this right, it’s a pilot trial section because we are coming up with a study to trial the shared-care like just now you’ve read through right? So this section is specially for that study but in the future when the study is finished, we will take off this section. So we just want you to see if you understand what you are reading.

ID: can

ET: So this is a video you can watch, for the people who are watching this, they are actually in our study, they will already be assigned to shared-care, so this is to help them understand what they will be going through.

ET: ok, can understand the video?

ID: yes.

ET: So this is a summary of the video because some people prefer to read the information

--- skip acceptability qns ---

ID: very cute, the video that they do ah, no no good but they not sensitive la, because when you say shared-care, the people in that, because multi-differential, you must different different people, like you see doctor doctor, so if you say pharmacist all that, this three picture ah… I think their picture will be like.. why all look like doctor? So when they do (the video) they not sensitive. If you say this other thing then put all.. because when you say multi.. then your cartoon I expect also…

KY: got pharmacist etc?

ID: correct correct, cause you said then they all is the same… you know they take at random, just the picture?

ID: some people, the learner is visual. So visual sometimes the words all.. you like to see all these picture then they directly associated with that picture..

ET: so you mean for the video, instead of just picture…

ID: no no, the video is good, but you see this part. You see healthcare professional? They want multi-discipline mah, but you see like this one ok, but its good that when you say team, you bring out here the 3 people right? So the 3 people will be the pharmacist all these things. So will be like.. what? Pharmacist put on stethoscope meh? No right,

KY: so like throw drugs maybe, hold some medication

ID: ah, right, and say another issue say number 2, doctor, see your primary care can be GP all that…

ET: no need to wear the surgeon gown

ID: haha yea correct correct.. cause when you say healthcare professional, you like that but the first thing my eyes see.. eh? How come different? You see something but not synchronizing with the picture.

ET: So its like, different from what you’ve been reading which is this 3 picture

ID: Ah, you see if you like that, that mean like that la (use the same picture). If you have this 3 picture, say never mind, even the pharmacist still wear stethoscope, you can tell, you have this 3. Means you put this 3 in the theme la, you don’t run, use the same. So people, like I can link, ah then suddenly say like… different people.

KY: do you think we should put in words to make it more specific that “ah this is actually for people who are in the trial only” cause just now we actually explain to you that this part is only for people in the trial, so we should at least write down in the thing so that next time I will ask them to read and then (inaudible)

ID: correct correct, its just that what im saying in the future, if you want to make video for this shared-care, so some people understand through video rather than writing, the picture must be consistent. This one I can say is people see, I mean do but cannot see, so they just do, no meaning, not synchronizing. That means it’s the task that you do without understand. Like I want picture, you give me picture but the picture don’t bring understanding.

-- final section –

ID: this is good (website)

ID: do you have self-examination, the steps, do you all teach the steps all this thing? Is it inside under education?

ET: ya, so we link you to the website, then on the website when you enter, you can navigate around, this is the dictionary for like certain terms that you don’t understand, you can search here

ID: I think they have la… that the self breast examination that should be the main thing. So these are the things that people like, for me I will teach my daughter, so sometimes very difficult so you will choose picture and then the type of like.. when you see different breast.. the size and shape, what does it .. ya.. good

ET: do you think like you would want more links?

ID: the link is good, because you see the website itself, people will tap on what they want to know, different people different. So if the site… if have like sexuality then people will go by that. Because these are the things like indirectly helping but I don’t want to tell you about sexuality problem because it is a concern so at least the website that website help to address all these things. Then they will say “ok, what is the help line?” then if I having these things, you need a sexologist right? Singapore not easily available, sexologist, so you have that line, I know I can contact that without even my husband know(ing).. so that line give me privacy option.

KY: so you will want more local / Singapore-specific links and resources?

ID: yes because you see if im having problems, I wouldn’t want to go to a website that is not local, because cannot get help, secondly you ang moh. Ang moh and me not the same. How you take life and image is different. So I rather like see the picture, it relate to me. Malay, Chinese, Indian, doesn’t matter but I see ang moh ah automatic, people will be very biased, “ah different”, everything will be different. So its like not significant to population. I want my own population, people who are like… even sometimes it can be the food, your food and my food not the same so its like, “is it food related?”

ID: so good to have local website and the whatever problem, like even like sexuality come in or other thing, say like for them, very important for me to educate my family especially got daughters. My daughter did ask me “mummy that means when I at your age, mummy when I grow up, that means my breast will be cut?” so like, how do you want to advice… I won’t know the technology like cancer markers all that but if you have like that ah (on the website), now I can say “you see now they have all these. When you grow older, when you have breast, I teach you how to check self examination, but we can go to these like to have like what option, like tumour markers all that very... we can refer to.

KY: so maybe even some information for your family members, care givers ?

ID: correct correct.. cause for us when we already suffer this, we don’t want other people (family members) ya, so in the sense, we at high risk, family will also be at high risk. So we don’t want, so what are the things, even in terms of food for cancer survivors, simple things. Food. Especially from recovery, surgery, chemo. Then after that I put in all these things so at least under one website for breast cancer survivors, that website, everything is under one roof. Can go shopping local in Singapore (products for survivors) all these thing available, so in the sense I am not alone, so you will also have that chat, like you see im experiencing this then somebody else as a member will go inside and like “ I also experience this” then we exchange

ET: like forum?

ID: ya, so at least good in the sense, I have this experience I went to see doctor so maybe you want to do (also). Ah you see cause some people don’t want to see doctor, they want to on my own. so at least forum better cause these are people who experience themselves, rather than google. Can be anybody… to have that (forum) will be complete

KY: ok so maybe we will put maybe some description of what the website contains.

ID: correct cause some people they don’t like to read. I only want to see straight what I want so like what you said, if you specified, I go direct to there, I don’t want to see other thing. Some people they don’t like to read a lot a lot information some people they don’t like to search also.. searching like that time I have very very bad peripheral numbness, its really very difficult... so difficulty even managing gadget with information which is important, so you don’t want to every time I ask question must search very difficult. In the sense like, should be no problem if software, that means telephone also can. So that means if they do that, that means I can access, cause some people don’t even have laptop, so its easier for them they use their smart phone.
