# Supplementary material for: Development and pilot testing of a decision aid for navigating breast cancer survivorship care
Source: BMC Med Inform Decis Mak. 2022 Dec 15;22:330. doi: 10.1186/s12911-022-02056-5 (PMC9753367; doi:10.1186/s12911-022-02056-5)
Supplement: Supplementary file 5 — Additional file 5. Transcripts and the final decision aid prototype. [file 12911_2022_2056_MOESM5_ESM.zip › Additional file 5/ID07_transcript .docx]

**Study ID**: ID07

**Interviewer(s)**: IC

**Date**: 16 November 2021

**Transcribed by**: GT

IC: I think I will just put it here. So for today we will be going through the decision aid together, each page at a time. So this decision aid consist of 5 key sections. As you are viewing each page and section, tell me out loud any thoughts that go through your mind. I may also prompt you with some questions along the way as you navigate through the pages. So after each section, maybe I just show you this [*IC passed ID the acceptability questionnaire for reference*]. I will be asking questions about the amount of information, the clarity and the presentation as well. So this is actually for you to fill up later on also but to make it easy I will just go through after each session also. Do you have any other questions for me before we start?

ID: No we can start.

IC: Please read the disclaimer on the page and then if you are ready you can press start.

ID: So I can actually click the yellow colour right?

IC: The interactive bubbles, not the yellow colour.

ID: So I click the interactive bubble is it?

IC: Yes.

ID: Then after that go to the next page?

IC: Yes.

ID: Then after that so many colours so I can.

IC: You can start with the first one yes. So for the part on the cancer survivorship, how do you think about the amount of information? Do you think it is just nice, too much, too little?

ID: Too little. So I need to tick here is it?

IC: I will tick it later.

ID: Clarity of information is actually ambiguity. Presentation I would say fair.

IC: So could you elaborate a bit more on what kind of information do you think we should include for the cancer survivorship part then?

ID: The relapse part. Since you all already put down there, that is the part where most cancer survivors most feared of but it was nothing mentioned about the relapse. And for us, we want that one, that is the most important thing why we are reading this. And why we want the aftercare because we do not want the relapse. But there is nothing mentioned about the relapse. So read already it is as good as not reading

IC: Which part do you think was confusing or ambiguous?

ID: Because you all put down there you know three years then what some hormonal therapy so that is considered cured or not cured. And also are you sure cancer can be cured? I don’t think so you know because so many medical journals that I have read through all say that cancer is no such thing as one hundred percent cure, there is always this possibility of having a relapse [*note: ID background is social work, used to read medical journals for 3h each night*]. And coincidentally my cancer support group I have seen quite a number of relapse and some have died. Three weeks ago, I just received news another of our member is also having a relapse and she is also now I think waiting for time. So these are things that really make us fearful but there is nothing mentioned about this.

IC: What do you think we can improve on the presentation slides?

ID: I think more bullet points because not everyone is very how to say is good in English language and also maybe some drawings because this platform will be good for those who can use the PC and those who are so called educated you know but cancer hits all kinds of people so people who are not computer savvy or IT savvy or people who are not English educated then they may have no access so maybe another language will be good or maybe you can have some printout as well you know make into a small little booklet you know so that it is easy for the survivor to actually refer to especially if it is old people.

IC: So we can continue on to the next part [*referring to ‘physical effects’ section*].

ID: So that’s it.

IC: This one you can click on the bubbles itself.

ID: Which bubble?

IC: So like on the surgery part, the radiotherapy.

ID: So I can click all is it?

IC: Yes.

ID: But I did most of it.

IC: So these are things that you…

ID: I went through all.

IC: You already know as well?

ID: Ya except for the Her2 because mine is hormone therapy.

IC: So these are familiar information to yourself?

ID: Ya.

ID: Then after that?

IC: Then the next part will be the emotional effects, you can press the next section.

ID: Next section is it?

IC: Yes.

ID: So after that click where?

IC: The next session so for this part for the physical and emotional?

ID: I would say too little also.

IC: Too little because there is a lot of information that you already know?

ID: Also with some ambiguity. Presentation I would also say fair. Because I think it’s more than that the side effects for the medicine is more than what was listed down there and also everybody react differently and then also the chemotherapy and all these things right I think also affect different people. Then next is it?

IC: Ya. So this is the next part sorry the previous section.

ID: Then after that?

IC: You can press the next button.

ID: So what am I supposed to press now?

IC: You can go back into options and you can look into the shared care one. (Pause while ID is reading) Then for these two parts?

ID: I think the usual care is just right, easy to understand because I went through all this so presentation I think is quite good. Then the shared care I don’t know how to say because the shared care I think it is a bit difficult to understand and then maybe the information is a bit too little. Presentation I think is fair because I think my feel is this shared care program is a very ideal program on paper but in practical it is not useful to most of our cancer survivors. Number one why I said that because if we go to GPs frankly speaking from my own experience right I don’t have much confidence with our Singapore GP. Because when I was doing chemotherapy I need to jab booster jab so with the letter written by the oncologist from National Cancer Centre I went to so many clinics to ask the doctor to help me jab my booster jab right all rejected me so that makes me question the part of the doctor number one the professionalism of the doctor and also this doctor so kiasi then how to trust them with post cancer survivor you know care I have no confidence this is one you can tell me they are trained whatever but I think there is still a very big question mark because it is a booster jab where if I come to SGH cancer centre right it is also the nurse that is jabbing me so you are telling me the nurse is actually more qualified than a GP so this is something I think a very big question mark and you ask me we are not talking about cancer it’s not those simple flu or fever that kind of thing where you take Panadol you can settle so to trust our lives to this kind of doctors I don’t think I want to take the risk. Number two the insurance claiming part because for all these kind of dot program [*note: ID’s father is under this program at NCC which was very troublesome for insurance claiming (vs. just using Medisave) and requests for other tests; thus, ID regretted signing up for the program*] so called like I think heart centre they have this dot program also so my experience is the claiming of insurance is very troublesome and also we cannot claim as much. For example, if I come cancer centre I can use my CPF to pay or medisave to pay or I can claim my insurance and if I claim insurance I can get the full amount. But if I go to a GP for aftercare consultation, I can only claim $20 then the rest I have to foot out myself this is number two. Number three medications wise also if you for this kind of GP right a lot of times medicine you cannot use CPF to pay, you have to use hard cash to pay so this is also a turnoff so definitely no and cancer medicine are all very expensive. Then number four it is also not ideal to me because with cancer after chemotherapy, radiotherapy and still on hormonal therapy you have a lot of other side effects, you have a lot of other illness that comes along so if we are cared for in the hospital, it is easier for the doctors here to make referral from one department to another department whereas when you go to a GP right then you will have to be like referral then you have to go to the polyclinic to take letter then you have to queue that kind so I think it is so very troublesome. So you ask me if I am given a choice my answer is no I will not take part in this program.

IC: I understand.

ID: So next one is it?

IC: Sorry so the difficult to understand is also…

ID: The difficult to understand is also because you all also never present the part, payment, money and then what are the benefits of joining this program. So if I join this program got no benefits and then straightaway now I already give you four, five disadvantages the non-benefits then why would I want to even consider or think about this program in the first place no. So I think it should be clearer and also it will be good if let’s say you all can have some benefits thrown in you know if not otherwise I think who would want to go for such a program and also for me personally my own experience I speak for myself. I was misdiagnosed with cancer for the first round, six months later then I got the proper diagnosis. So because of this reason also I am very kiasi and very kiasu that’s why you can check my records I got a lot of appointments with a lot of doctors here because the trust has really gone down so I wouldn’t want to take the risk another time. Then the next one is what?

IC: You can press the next section.

ID: So I press number two is it?

IC: Mm.

ID: So after that?

IC: Then the next section.

ID: So what should I press? The number is it?

IC: Mm the number or the arrow is fine.

ID: So family physician so I would say I am not confident at all. So click number one is it or what?

IC: So this one is actually reference questions.

ID: I am not confident at all.

IC: Understand.

ID: Then how confident are you seeing this trained doctor for your cancer care which is the family physician right also no. So I go on to the next page right?

IC: Mm.

ID: This one I think I would choose maybe neutral number three. I would also say maybe I think it’s not useful at all. So can go to the next page right?

IC: Mm.

ID: This one I would say neutral for question five. Number six is no. Number seven is actually I already decided I don’t want already so I don’t know what number.

IC: So even if there’s more evidence of the benefits also no. Then maybe I will just put no for you.

ID: So next page right?

IC: Mm. So these questions we want to see whether or not these questions will be helpful for patients to see like which side they lean on.

ID: I lean to usual care.

IC: You are leaning to usual care?

ID: Yes.

IC: But for this kind of questions, do you think are there any other factors that would other than the ones you mentioned just now, would that affect your decision?

ID: I think this is something that concerns our lives and if we don’t take responsibility for our own safety and our own life right then anything goes wrong who is going to be answerable and actually if you ask me I receive quite a wholesome care system you know receive the care from the whole healthcare system here in SGH which you ask me I am happy with it. Then if you talk about family doctor as and when if I feel that I want my family doctor to know my own condition better or whatever I can always just go in and give him the info that I want to give and how much to give you know it is up to me to decide you know and then if let’s say my GP felt that there is a need for him or her to contact my onco they are all doctors they can contact on their own so actually this is a redundant system I don’t need that. And also if the GP if let’s say is a family doctor who has been with me for many years a lot of time when you just go in and talk talk like that not taking medicine or anything it’s even free of charge so why should I pay so this is a redundant system and don’t waste time. So what should I click? The next one is what?

IC: The next one so would be this one this is the preference exercise the question.

ID: So what should I put?

IC: The amount of information.

ID: Too little, also ambiguity also fair. Comparison the options too little. Then I would say it’s fair. Then after I click other resources is it?

IC: Mm. That one won’t open so that’s it for the decision aid thank you so much.

ID: Still got some other thing?

IC: So for the other resources those actually linked to different websites for additional information that the patient would be interested because this one cannot connect to the internet so cannot see.

ID: The rest I need to answer?

IC: Yes, so before you answer those questions can I ask a few additional questions?

ID: Can, I can give you the answers.

IC: So overall what do you think about the decision aid?

ID: Lousy, sorry.

IC: It is okay.

ID: I want to be as honest as I mean no point in me coming 40 minutes giving you all the political correct answers to make you all happy and then your report looks good but it is not going to benefit us no point.

IC: I understand. So do you mind elaborating on why you think it is so?

ID: Like I tell you already I think for myself based on whatever I just shared earlier I will not take part in this program.

IC: Overall for the navigation wise, do you think it is easy? Or there’s some confusion in where to press this kind of thing?

ID: I think a bit, it can be more user friendly.

(Doing acceptability questionnaire)

ID: So here the question here it says did you find this decision aid useful in making a decision about follow-up so my answer is no. I have already given the reasons. Then would you use this decision aid to discuss care options with your oncologist you know my answer is also no because since I am not (repeated) keen at all so there’s no point. Then you ask will you recommend the use of this decision aid to other cancer survivors my answer is no because myself already not considering it so why would I want to introduce things that I think is no good to other people. You only recommend things that are good so if I feel that is no good then there is no point in me recommending. Why do you think so this one would be NA.

IC: And then any other things that you think might not be?

ID: No, I think I have already given already mentioned.

IC: Understand, no worries. I think that’s one part there’s a few questions as well [*referring to the first part of the acceptability questionnaire*].*.*.

ID: I think is neutral. Then what else?

IC: Can you help me fill up this demographic information?

ID: Okay today’s date is 16th right?

IC: Yes.
