# Supplementary material for: Development and pilot testing of a decision aid for navigating breast cancer survivorship care
Source: BMC Med Inform Decis Mak. 2022 Dec 15;22:330. doi: 10.1186/s12911-022-02056-5 (PMC9753367; doi:10.1186/s12911-022-02056-5)
Supplement: Supplementary file 5 — Additional file 5. Transcripts and the final decision aid prototype. [file 12911_2022_2056_MOESM5_ESM.zip › Additional file 5/HCP01 - Transcript.docx]

**Study ID: HCP01 Date: 22/11/19**

**Interviewer(s): ET & KY**

HCP: the introduction is fine…the only thing is words like “endocrine therapy” patients may not understand what endocrine therapy means. For most patients, they understand what hormone treatment mean rather than endocrine therapy.

HCP: ok, so “why is cancer survivorship important” these are fine, the only thing is we may have to dump down the language for the average person reading this. You can check the reading level on Microsoft word and there is supposed to be a standard of reading level that you’re supposed to reach, as in low level, not high. Once its above that high level, a lot of people may struggle to understand so you’ve got to think about the average person that reads this may not understand words that are here. So, when you have words like “coordinate care” things like that, sometimes in a consent form that we give for clinical trials, we actually have to dump down the language. So the certain reading level you’re supposed to (achieve). You may have to think about “if you put this in the Microsoft word” and check the reading level, you may have a very high reading level that means “ok, I need to start changing words like intervene” and change it to a less advance word – that may be important.

Sp: so the bubbles go to separate pages? Oh I see…

Sp: so the risk for lung cancer, it says the risk is high if you receive radiation therapy. So it says after mastectomy but you don’t have to have mastectomy to receive radiation. So they can just receive radiation after breast conserving surgery. So you can just say “breast surgery” because this qualifies it. “This risk is only there if you receive radiation after mastectomy” which is not true. The radiation doesn’t matter what kind of surgery you have, so instead of saying mastectomy just say breast surgery.

Sp: I think it’s fine (cancer slides), the only thing is that there’s a possibility that this may then worry patients. Because, depends on how much discussion the person has had with the doctor, or they may have forgotten, then this may trigger the concerns again. “Now I don’t want to take this medicine because it tells me there is a risk of bone cancer”. So you may want to say “if you are concerned, you may wish to discuss this further with your primary doctor”. But I think its good that you say that the risk is rare, because otherwise, people panic. So again, words like manifest, you may want to dump down your language, instead of saying manifest, you can say “appear” “come up”, you want to avoid words that require high reading level.

HCP: if you want to use “endocrine therapy” I think its fine but in the beginning you should at least say “hormone therapy” then brackets endocrine therapy.

KY: So in this case, the bubbles can actually be clicked.

HCP: then you need to tell them, because I don’t know, cause you see you can click on the bubbles.

HCP: did you say what AI was before? So you may want to qualify what AI means because otherwise people will think artificial intelligence, cause that’s what most people think AI is.

HCP: There’s another targeted therapy now called pertuzumab, but you already said example, maybe just leave it as that because there are other types of targeted therapy.

KY: so in this case, do you think it’s useful to have the long-term and late effects segregation?

HCP: I think its fine.

HCP: if I click on this (end of step 1) what happens? It’s the same as the arrows (next) is it?

HCP: so pelvic examination… maybe you want to speak to Kiley about this because not everyone will necessarily have a pelvic examination because some doctors don’t do this, so you may want to discuss… but I’m ok with it because he may think that it’s standard of care but some doctors don’t.

HCP: so… I can click on the bubble? Ok then again you may have to say that I can click on the bubble.

HCP: just that… the term primary care physician is not commonly used in Singapore. People may wonder what does that mean although you did say general practitioners…

HCP: ok again, things like comorbidities, that’s a very medical jargon, so its not just English level reading, it’s a medical jargon. No one’s going to.. a lay person will not understand what comorbidity mean. So management of other medical conditions as opposed to comorbidity. And maybe you’ll want to put an arrow down here rather than.. (next arrow at bottom rather than side)

HCP: what’s HCP? Was it defined before?

KY: yes, but I think we’re going to remove this part. It’s causing too much confusion (asterisk)

HCP: yeah, because even if I see it in one slide, I won’t remember it anymore despite the fact that I’m looking out for words.

HCP: sorry, the arrow’s a bit confusing. Sometimes its up there sometimes its down here, sometimes it disappears, now its down here again… yeah so keep it consistent.

HCP: you may want to clarify that you’re not being charged more (for pcp), which is your cost in participating in the trial is no more higher than the usual standard. Potentially, it may be less because if you end up in the shared care model, you’ll get less. If you end up in the standard care model, then its just the usual cost. So the cost participation is no higher than if you choose not to join this trial. (cost part of table)

HCP: you may want to leave a greater gap between this 2 cause I start reading and realise later than it’s a different column. (table format)

HCP: You may want to… because I had to read this 3 times to understand that statement because… you may want to simplify the word itself (favorable are the cost savings etc.).. maybe its just me but I had to read it 2-3 times before I could understand what it’s trying to ask.

HCP: now the appointments are not made by the participant right? Appointments are made by… if you’re on a trial, they make the appointments for you. Because if I answer this question, I would think of making a new appointment, then that would qualify what I think about appointment is – when I try to make a new appointment, as opposed to someone making an appointment for me.

HCP: Again, they may get confused. “what is patient navigation again?” you may have to qualify – that means someone helping you. Maybe not the word navigate but helping you arrange your appointments and things. Isn’t it the same as before? Care coordination versus patient navigation?

KY: -explains diff- I think we need to qualify that…

HCP: This doesn’t appear unless I hover other it is it? (video play button) you want to make it appear so that straight away they know so they click. Because I almost click there (next button)

HCP: I like it, very nice (video). ok so this is…. Oh the internet (more information). Ok.

HCP: ok so how many slides are there [ET: 55 including popups]. So its very detailed. MY only comment is that its.. people may lose interest half way through because there’s just so much. And you have a lot of things that.. because the purpose of this is to purely talk about the trial right?

KY: -explain that it’s for future purposes beyond BASIC-

HCP: you may want to differentiate into 2. Because you’re trying to do both right now when your only purpose is really to get people on trial. But there are.. you could almost summarise everything into the video itself whereas before the video I had to watch almost 30-40 slides and they were almost very repetitive. I don’t know whether you may want to condense it and make it easier because most of the time in clinical trials, they (participants) just read one page of information and this is.. I know you’re trying to give a lot of information but it almost gives too much information that it drags on and on. Cause when I was going through the slide I was like “when is it going to get to the point of this study” and it just went on and on. So, 50 slides is a lot and you may want to think about how you can compact it. Like a lite version. You know how when they do clinical trial consent form. Sometimes they have a very thick version then they have a lite version that people sometimes just read the lite version that condenses everything down, so the video almost does that but I don’t have a choice of doing a lite version. I had to read through the whole thing then do the lite version again. Its very good information but I think it’s good information as a resource like you said but is it too much for the purpose of what you want to achieve? To get people on the trial, it may be a bit too much. So you may want to separate to one for resources and one for the purpose of the trial, so that the trial thing is just “yes/no, I want to participate in the trial”. This is actually using up a lot of time just for them to say no I don’t want to participate in the trial.

KY: is there anything that is inaccurate?

HCP: no no, everything is accurate, it’s just the wording etc, you want to make it very simple. Getting people on trial usually means you have to make things very simple to understand why am I here in this trial, what are my pros and cons. Whereas if you talk in a very long-winded way, sometimes, the thing about patients sitting in the waiting room, they don’t want to be here, they just want to go. And then you make them watch a video, watch a powerpoint slide that is 50 slides, it can get a bit too much for them.

KY: eventually, besides just the trial, we wanted to make it such that [HCP: it’s a very nice resource] I think the bigger end goal is that eventually, disregarding the trial, there’s this option. This (PDA) will at least help them go through the [HCP: ya I think that as a purpose, this is actually very good].

KY: just the last part, for the preference / values section, we wanted to actually guide the patients to think about… because sometimes they can be very adversed to shared-care just by looking at it, but by going through all the factors, they may find that…

HCP: oh yeah, so the purpose of this is actually… why do you have this? To help you decide to enroll them of not? (into study)

KY: no, so it’s not for the purpose of trial. Eventually, certain patients may have preferences that they just prefer the usual care where they keep seeing oncologist [HCP: so if they choose “I prefer usual care”, then you don’t enroll them into the trial?] so this one is for the eventual goal. Our trial, like you said, we want to take out certain elements of it, like the key summarized version of the first 40 slides and then we go into the video to just let them understand [HCP: I see, so we’re just skipping this.. cause I was wondering that the purpose of this is you just select the one that don’t want… ok that doesn’t make sense -talk about BASIC trial-]

KY: so this is more of like a value add to help them to see that “oh, these are some factors that I do subconsciously consider when we talk about…” are there any other factors that you think that patients would consider in this part of.. when making this kind of follow-up care decisions? So here we have things like cost, or like they’re personal preference for the doctors as well as having the idea of care navigator as well as coordination. Are there any factors that you think we should also flash it out for them to consider? Because I think a lot of these things are subconscious and a lot of them they package them altogether.

HCP: I think the most subconscious thing, and its actually not that subconscious, they may actually voice it, of people not wanting shared-care, they say they are worried they are getting an inferior surveillance because they’ve gone from a specialist to a primary care physician. Right, wrongly they think this is not as good even if they’re trained, they’re still not a specialist. And they’ve gone from a cancer centre to a polyclinic and sometimes the stigma associated with that is “cancer centre is better than polyclinic”. So these are not subconscious but actually the real thing. And we’ve encountered that when we want to refer patients; after 10 year follow up, send them to polyclinic etc. they don’t want to go, “no I want to stick with you because you’re (at a) cancer centre. Also, I want to stay with you because you’re my doctor, I’m familiar with you. I go polyclinic, each time I see a different doctor, I don’t like that.” Sometimes, they are happy to go, “oh good, I’ll get away from this place. I’m not cancer patient anymore. I can go to my polyclinic which is nearby”. So these are the pulling things and then there are the ones that pull them back to NCC. It’s the fact that they don’t like PCP.

KY: polyclinic actually fam med, so stick to same doctor, between 2-3 doctors [HCP: ok you may want to say that the one seeing you are….you say they are trained but people may assume they’re just trained doctors, you want to say they are specifically doctors that have come to NCC and work, and specifically trained to know how to follow-up on cancer patients]
