# Supplementary material for: Development and pilot testing of a decision aid for navigating breast cancer survivorship care
Source: BMC Med Inform Decis Mak. 2022 Dec 15;22:330. doi: 10.1186/s12911-022-02056-5 (PMC9753367; doi:10.1186/s12911-022-02056-5)
Supplement: Supplementary file 5 — Additional file 5. Transcripts and the final decision aid prototype. [file 12911_2022_2056_MOESM5_ESM.zip › Additional file 5/ID02 - Transcript.docx]

**Study ID: ID02 Date: 31/10/19**

**Interviewer(s): ET & KY**

**PART 1**

ET: ok? Can understand?

ID: yes

ET: ok then we start

ID: now only start? Ok

ID: So when I read also, I will make sure I understand?

ET: Yes, anything you don’t understand or you think is unclear you can voice out cause its part of feedback.

ID: ok, I understand… ok

ID: broad range of physical, psychological, financial, emotional, spiritual and social challenges, ya.. I think a lot more than that..

**PART 2**

ET: just now you said that there were more than just these right? What else do you think we should add?

ID: ya… ok la I treat it as all these ah.

ET: hmm ok

--- acceptability questionnaire ---

ET: then how about the clarity?

ID: a bit complicated lor, you need to really look through then you have to understand a bit more. Let’s say depends on the level of your understanding. Let’s say if you not really understand too much about what happen to you then you don’t understand much about this…

ET: do you think putting it in an image like that with words is good or do you think it can be better.. like maybe less words or… more colour

ID: try to be less words, for me I think its like 1 2 3 4 very (list down) better (importance of survivorship)

ID: cancer preventions… ok.. I didn’t know that. Recurrence and second cancer I didn’t know that

ID: is this rare? (risk of getting cancer) is it rare?

ID: ok let me understand this term… that means what you’re trying to say is prevention all this is very important and make sure that there is no other kind of cancer coming up is very important. Nevertheless, the risk of getting that is very rare. Is it rare?

ET: Do you want to find out more? Because here it says you can find out more by clicking on all these (bubbles)

ID: orh, I haven’t read all that yet…

ID: you all are talking about all breast cancer only is it?

ET: breast cancer survivors

ID: only breast cancer, not any other type ah, oh ok ok… ok this one I understand… (agreeing with slide content and saying she understand)

ID: mhmm which I have one, you never include, which happened to me. Chemotherapy will also make a condition called avascular necrosis. This has happened to me.

ET: so you want us to include that?

ID: I don’t know I just suggest to you that chemotherapy the side effect that you have not included here which is a lot a lot, do you have any?

ET: Ok lets go further on to see, so now we talk about..

ID: long term and late side effect, ok ok.. (agreeing with slide content)

ID: But I talk to her (Dr) about all these she don’t care about me le… (laughs)

ET: we can look into the specific treatments now…

ID: (agreeing with slide content).. and also the other one is like, after the surgery.. finish is it, is there more?

ET: no, for surgery its just like that

ID: it’s a permanent effect, numbness over the surgery…(read from list on slide), there’s another one which is like after you have the surgery, that our veins.. sometimes when you come across a very intensive operations or medical conditions, then actually when you need to have a second chemo or you need to have a… so the effect is like your hand and all the veins here cannot be used anymore, for blood test, you can’t use for blood pressure, you can’t use for injections of the drugs. This is also one of the permanent that I feel everyday you are coming across. You are actually reminding you every time you come and see doctor, take (blood) pressure, “ok this hand I cannot use, must use this hand”.

ID: ok luckily I don’t have this effect… this is long term effect this is late effect.. late effect means…?

ET: means after you finish the treatment then suddenly a few years down the road then you experience it.

ID: long term effect then.. immediate you can see…?

ET: its like during your treatment you get it but it persists even after you have finished your treatment.

ID: orh ok actually I don’t know the definition of this 2…

ET: its over here

ID: ok.. (reads slides) ok chemotherapy… huh? Got this ah? Cognitive got problem ah? No wonder im so stupid now… (continue reading slides and agreeing)… huh? Heart disease ah? That’s why I tell her I may have heart disease she (Dr) don’t want to bother about me.

KY: what was your chemo regiment? Do you remember the specific drug name? because some drugs are more prone…

ID: my one is very intensive, super intensive, I choose the most intensive one

KY: to try to eradicate? I think surveillance la and screening

ID: I should let you(?) read this page before I see her, she always don’t want to chap about me… endocrine therapy, tamoxifen long terms.. ok this one I didn’t take, I don’t know

ET: but if you read through do you think its easy to understand?

ID: (reads and agree).. what is all these meaning?

ET: these are the names of the drugs

ID: ah I see ok…. Targeted therapy… ok (agree with slide)…. Ya I think she thought im in this line (distress), so she don’t want to care about me… now I know…

**PART 3**

--- acceptability questionnaire ---

KY: maybe just one question, just now you know the late and the long term effects right, do you think there’s a need to separate them? Or we can just talk about it in general, like together

ID: I personally don’t feel much difference about the late and long term, it seems to me its like the same thing. But how to differentiate between late and long term? To me its like those are the side effects. Some will come late some will come immediate. Some long term some short. Some don’t even have. When you have late and long term like.. very hard to.. like you make people confused. So maybe you can write like “this may be coming late” or what, cause they these effect are the same, when you separate into 2 parts, people will be confused. So you just write then you may bracket it, those are some.. maybe below can write some are coming late or maybe.. I feel they are the same la, I don’t like, for me I don’t like some is coming here some is coming there, I don’t have any difference of these 2 ok..

ET: so your options… (next section)

ID: ok available options, what options I have? Survivorship care options… huh? Got options one ah? I don’t know leh?

ET: Ok so right, this is something new, we haven’t launched the shared-care option, so what you’re currently going through is usual care…

ID: oh is it? Usual care is the one I’m seeing. Do you know got quota one not? Do you have a lot of quota or not? Its like your condition is A you can only have this, condition B, C, D then you have that… (reads slide)…

**PART 4**

ID: all these I also need to request (usual care events table)… (read slides)… ok no leh, this one all don’t have leh, how come I don’t have this bone mineral density every 2 years, where got, this one also don’t have.. maybe its because I didn’t take tamoxifen, I only have this… so this one?

KY: this one also only if you taking drugs that require

ID: then uh… ok la just talk nonsense ah… people don’t have these 2 doesn’t mean they don’t have bone problems, we all have gone chemotherapy just now you said radio you have these side effects blah blah.. all the long term short term every term ah, but now you are only taking care of this group which is so… I feel like not very fair right?... no la im not complaining…..

ID: what is shared-care, what is shared-care (reads slide)… when will it be launched?

**PART 5**

ID: (reads slide)… oh ya I need this. When did it (shared-care) start?

ET: we still need to run trial for it first, then after the study approve then..

ID: choose me for your trial, so I can save my money on my healthcare. Then its like depending on your health status, your doctor will dare.. (read slides) means those like annual checking all those things right??

ET: so lets say they do already then they realise you need to come back more often then they will make more appointments with you. But if don’t need then ya…

ID: pharmacist?.. (reads slide)… pharmacists also involve ah? Ok.. Roles.. healthcare professionals… management of…

ET: comorbidities, do you know what is this?

ID: I don’t know, what is this?

KY: chronic conditions, like high blood pressure, diabetes, heart diseases, maybe we will change the wording

ID: comorbidities… I didn’t come across this word..

KY: ok we can change it so that it will become more user-friendly

ID: ok.. probably need some explanation la.. (continues reading)… so this is by who?

ET: over here, so if this is ticked then its by this, do you want to go back?

ID: ah ok ok, ya I didn’t really (see), so it will be oncology, GP and pharmacist... so there are different role… ok so oncology will take care of cancer-related things and GP will be taking care of all others.. pharmacists will be taking care of drugs… ok that’s more understand.. so this part is… health promotion? What do you mean by health promotion?

ET: so its like, “do you smoke? you need to cut down on smoking” or “if you drink?” then promote…

ID: oh ok daily health maintenance la

ID: HCP including… (asterisk)

ET: is this a bit weird? Do you understand this? No? don’t understand?

ID: done at the oncologist side but may be assisted by other HCPs…

ET: healthcare professionals

ID:… including supportive care nurses (SCNs)… ok

ET: so its basically saying the oncologists themselves will not do it for this one

ID: ok.. where is your star? (asterisk)

ET: here

ID: ok go back to this one.. this star where? Oh ok ok

ET: is this confusing? Should we just take it out?

ID: no its not confusing eh provided you see the star.. I overlooked the star… that’s why I think you should have a style, this one is for which one…

ET: so its easier if we put it by the type of doctor rather than the table that you have to ownself see is it?

ID: I think this is clear, this is clear, this is very clear (this = table)

ID: (reads & agrees)… how is care coordinated? That means just now that one you repeat one more time so that all this care that is given by the doctor… the result.. this is the result given to you? Sorry you understand what I mean? Im also a bit confused about what I said.

ET: erm no, so just now we said shared-care right, like over here, got these 3 people, correct? These 3 people are all from different institution like the oncologist is from national cancer centre then these 2 are not. So how do they know about you? Like how do they care for you? Like how is their care being coordinated?

ID: How do their care being coordinate?

ET: so lets say you come here you got all your information about all your cancer treatment and what you did right? But then now when you go to a polyclinic doctor, they won’t receive the same information?

ID: they will receive right? All the confidential information

ET: they can check if you ask them to check but they won’t actively check unless you tell them to right? But then now in the shared-care model..

ID: but they need to check before they can see you right?

KY: so sometimes they will not… for example you say you are a cancer survivor parked under NCC then ok oncologist take care of you, they won’t actively go and see got what problem and understand some of the side effects…

ET: ya but in this case its like they will be coordinating la, so they will find out more and like actively help you.

ID: ok so are those people under one roof, or like what you mean is like oncology and the GP. So your GP is it under this roof or the GP that is seeing us is under private clinic?

KY: now we are trying out, because we are starting with NCC so we are trying with the SingHealth polyclinics first.

ID: oh Singhealth polyclinics…

KY: ya, but definitely, in the future, once we think that this is ok right, and then as you know private and public, we need some government subsidies to back up in the long run so only slowly then we may expand towards the primary care and also because now all our health records everything it is easier for public institutions to retrieve as compared to those private, I think a lot of them are still fighting for access

ID: access ya, especially those who cannot tell like me, what are the treatment you have been taken 20 years ago, how can I remember all those? I also don’t want to remember. So the meaning of this page is... all these things will be recorded in your file. After taking care, you will have all your summaries in your personal files. That means between healthcare professionals…

ET: they can see this information

ID: ok, now I understand, ok next… comparing the two options (of) care model...

ET: so the usual care and the shared-care

ID: The usual care is only oncologist right? The shared-care is like what you said, it will be linked out to polyclinic and all those thing so… (reads slide)….

ID: ok this one very clear… how well does the model work…

ET: not so clear?

ID: ok who is suitable for the model, this one very clear. You and her. How does this model work… ok… (Reads slide)..ok so this one is study of this model in other country?

ET: ya, because Singapore don’t have yet

ID: ah ok can, I can understand… (agree with slide for cost).. ok same? Hahah

ID: (reads slide funding)... Eh? This one all same what!

ET: ya

ID: (continues reading slide on location) ok this one easy… what will your relationship with your oncologist… this one will continue… but within once a year she also forget me already…

ET: ya so it’s the same just that now you will see the other doctor more.

ID: but oncology will be the same person right? Shared-care will be all different doctor already.

ET: your oncologist is the same then…

ID: still, seeing once a year is better than seeing once in your life, correct? So this will be still.. (reads slide)… actively communicate.. also through paper only right?

ET: ya..

ID: (reading..) in the trial of shared-care, there is some limitation to the doctor you can choose because only… that’s what you said la..

ET: ya, we start small

ID: ok.. but this one? Mmhmm..

ET: can understand?

ID: I understand la but…(cause we explained)

--- acceptability questionnaire ---

ET: was the information too much?

ID: because now its still a bit confusing, then I also see a lot of information are overlapping which is the professional and GP are almost the same thing, maybe because its not completely up yet so its like a lot of uncertainty, its like US and Australia they are doing a lot and all those thing, so it still have a certain level of confusion, it is…

ET: quite hard to understand?

ID: ya, you have to really like what I have to ask you, what is the whole picture, the shared-care all this, then you explain to me then I understand but if lets say I do not get the explanation from any of you, then I may consider it… eh? Why suddenly go to US and Australia, you know? That kind of feeling. Like just now when I read I said “eh? Suddenly you mention about Australia and US, is it any related of this with the (option)… there’s no (link), we just wanted to start-up in Singapore, maybe you can just write “this will be start-up in Singapore soon but we get the good result in all these countries”…

KY: context?

ID: ahh then you give example for the other country because suddenly you put other country I may feel confused. Like mmmmm eh? How come got other country coming into this? I mean this is my personal comment….

ID: I think less information, this is a bit more, I feel, I see repeating some, some of the things are actually repeating..

--- Completing value section, subject re-read the header each time it appeared on the slide ---

ID: how comfortable are you with trained primary care doctor.. that means this is referring to what you are mentioning just now, how comfortable… half-half only, I haven’t see yet. Ok? Fair right? (continues values section)… In your option (opinion)……

ET: is the phrasing a bit weird?

ID: how favorable are you with the cost.. center, middle… (continues value section)…

ET: the ease of making so like is it very important to you that its easy to make appointment or like you don’t really mind.. or like its not so important

ID: middle la middle… err this is very detailed already… in your opinion is it easier to make an appointment with the doctor in the polyclinic or with the oncologist.. you see ah, this is totally different things leh. In the polyclinic you make the appointment with any doctor, you have 20, 30, 40, 50, 100 but this is (ncc) you only make to the same doctor. So in your opinion, easier.. of course its easier in the polyclinic la but I don’t want it… you get what I mean? Because its totally different thing of comparison… (continues doing value section)..

ID: oh this one is… (finished section)

ET: ya so based on your answer, it will help you evaluate which one do you think like is more… like you prefer la…

ID: for the odd number questions.. where is odd number question?

ET: so she got write (your answers to the qns) for you

KY: you know you answer whether how important the factor is to you then after that you will put your preference right? For example one that you prefer..

ID: oh ok, I didn’t know about this

KY: ya so something like that to actually help the person using this to actually see “oh ya, I didn’t know that actually im more towards .. somethings are more important to me than .. ya.. so based on this you can kind of weigh la.. so for example in this case, if patient navigation is like not very important and also.., then maybe you won’t benefit as much from shared-care. Maybe you just want one aspect of the thing, so I think this will help you, help the person who use to be… (inaudible)

ID: so its if like my 1 and 2 is like..

KY: most important and 50% right? So lets say you can kind of know that for this 50% is more like you are neutral towards the shared care or… so in this case, this is a very important factor and neutral you’ll be like ok. Then this is also quite neutral [ID: towards the high side] but things like… I understand your concern with this one cos it’s a bias question cos easier and whether you want it is different, we will try and change it, but lets say ideally this question will actually help you, for example lets say in this case you are more leaning towards the polyclinic side right

ID: I think this one will get you to accept the polyclinic one

KY: shared care

ID: ya! To guide the patient, you better go this

KY: So in this case its like least important, it just mean that you will be more towards it

Sp. Ya understand

--- acceptability questionnaire ---

Section 4

ID: huh? This is the introduction actually ah?

ET: to the study that we want to do

ID: (reading..) maybe.. suboptional?

ET: optimal*

ID: that means you will kick you out la… kick you out from here la

KY: no as in like not the..

ET: not at the best state..

KY: so they may be not consider things like, for example next time you may visit, they may not ask you so much about your other aspects, like for example what your GP is doing with you

ID: ya, she (Dr) don’t really care about…

KY: ya, so I think its good to know that what GP is also doing with you so that she can know that “oh, at least that part is settled..”

ID: ok.. (reading slides).. only 12months ah?

ET: ya cause it’s a trial, k so this is a video that explains what happen when you join as the shared-care arm”

ID: so you will like… pulling patients from this oncology care to the shared-care

KY: trial only

ID: that means those like already past.. then they will pull you out, so all the care like mammograms everything…

KY: will still be done

ID: all this one will be done.. like just now the three tick table la

KY: this one (video) will have some visual

ET: ya, maybe you will understand better with the video

ET: can understand right?

ID: yes, understand well

ET: ok, so this page Is a summary of the video, you can just read through briefly.

ID: ok, at least one scheduled appointment with a polyclinic doctor within 3months, ok.. (read slide)

--- Last section ---

ET: so this is just additional information for people who wanna see it.. so the website

ID: basically the whole idea of today, we all talking is the switching to.. or not switching la you just…

ET: its for you to decide, help you decide…

ID: help you decide if you want to participate in the shared care? Ok..
