# Supplementary material for: Development and pilot testing of a decision aid for navigating breast cancer survivorship care
Source: BMC Med Inform Decis Mak. 2022 Dec 15;22:330. doi: 10.1186/s12911-022-02056-5 (PMC9753367; doi:10.1186/s12911-022-02056-5)
Supplement: Supplementary file 5 — Additional file 5. Transcripts and the final decision aid prototype. [file 12911_2022_2056_MOESM5_ESM.zip › Additional file 5/HCP04_transcript.docx]

**Study ID: HCP04 Date: 30/1/19**

**Interviewer(s): ET & KY**

HCP: so this is meant for the clinicians right?

ET: Survivors.

Sh looks confused, KY explains page tracker at bottom of page

HCP: so this bit talks about effects, the emotional effects, physical effects and how survivorship care helps to manage it? Whereas this section talks about how it is also important for the picking up of the recurrence is it?

KY : So it actually [inaudible] This one is more about the importance of due diligence after treatment and everything ends.

HCP: This is like pretty rare, I suppose it can happen as well (increase risk of cancer as an effect)…

KY: We wanted to quantify it

HCP: So this one comes with a figure (lung) so does this one come with a figure as well (other cancers)… don’t have right? Maybe we should [KY: find out what are the..] ya, see whether you have any information or not, because its quite rare a occurrence also this one. Or maybe you could lump the two together “increased risk of cancers for example lung, soft tissues” it’s just both together. And then you can quote the data for the lung, that would save you. That’s just one option.

KY: So for this popup, the back arrow is not for the popup. There’s a [inaudible]

HCP: oh I see there’s a cross (navigation of popup). Cause when you read this way, I suppose I don’t go back there again (look to the right side).

KY: so I put an option to go back…?

HCP: Maybe either bigger, make it bigger then it’s a bit more visible. Because you’ll be clicking the bottom bit all along.

HCP: That’s fine, the rest are ok.. (radio popup)

HCP: So just now was physical, now emotional, ok that’s fine.

HCP: not in contact… maybe not “usually” in contact, cause not in contact seems like we’re practicing very different… (usual care) just my thought. (…in their own ways) Which is true... Sometimes there may be occasional.. we write memo to one another but not usually, not as coordinated.

HCP: Actually rad oncs also follow up

KY: so typically for the very long term ones, what is the schedule like?

HCP: the rad oncs will also follow up for the long term follow ups because not all patients receive chemotherapy and not all receive radiotherapy so for those that did not receive chemo, may only be followed up by surgeons and rad oncs

KY: what if patients receive all three?

HCP: they will usually be followed up with by all three.

HCP: What’s the purpose of putting this number here? (did you know part)

KY: Because for BASIC we’re getting only [inaudible] from the family physician clinic so there will be firstly a bit higher cost, and also the concern with the changing of doctor … the polyclinic also has permission to make it such that the patient usually sees the same doctor

HCP: so this is the family physician clinic right? To allow them to see the doctor.

KY: Cuz previous runs a lot of them say that [inaudible]]…

HCP: I see…

ET: do you think that this information can be better placed at the back with the shared care?

HCP: cause im just trying to understand the context. I will see the back…

KY: So we should label doctors as onco for care schedule diagram?

HCP: ya make it a bit vague. So that’s the usual care one right?

KY: Yes.

HCP: so the pharmacists will be the coordinator? Do you want to consider putting this in a triangular format just to show a more… holistic view. The layout is good but the triangular form gives you the.. idk, you consider. (shared care imagery)

HCP: so this assumes that the person read the first bit first right? Because you did not go into the details of what the surgeon…

KY: Maybe we should manipulate flow such that usual care is always read first

HCP: So if you’re gonna let them read first one followed by this one then the description for this one is probably gonna be the same as earlier right?

KY: Yes, then do you think maybe it’s better for us to introduce what is a family physician because that is the “Did you know” part?

HCP: So this is in the usual care, this is what the primary physicians do la?

KY: Yes, so the key difference will be the cancer survivorship issues.

HCP: so I think the trick is to show them that this is what happens in the usual care, this is what the oncologist do in the usual care and then this is the shared care, so this talks about what the shared care is about… ok I understand.

HCP: Maybe that thing, the same doctor one can be put over here (shared care) then this one applies to them more, better.

HCP: The who holds onto the SCP? This is a physical copy or electronic copy?

KY: But for the pharmacy navigator side it will be, because they are not part of the … so in our care plan itself we will have gotten the [inaudible] and part of RD(?) wise we are going to email them if they are interested, and [inaudible] and the surgeons will not have the patient details, they will just get the RDID…

HCP: The navigators are not being paid right?

KY: The study we are paying them, but by right they can walk in and everything, technically it’s free

HCP: This one is like what is the difference for the patient participating in this (in terms of additional cost?)

KY: We can update this in future after the study.

HCP: Sorry, I don’t.. so what’s this supposed to show me? (what matters to you introduction) Maybe you should put, prefer usual care and prefer shared care. Cause now its food in the cafeteria so it’s a bit confusing… So if you choose usual care you’ll be asked…

KY: [inaudible] So subsequently there will be questions, and the questions will have a scale.

HCP: Right. So let’s say I click over here, it will be more towards preferring usual care?

KY: Ok so if you go to the next one.. The questions are in 12345.

ET: It may be confusing… So we’re trying to say, if you choose more 5s you prefer shared care, and if you choose more 1s you prefer usual care.

HCP: Ok I understand that. But what is this “reason for usual care” “reason for shared care” means?

KY: so we were trying to – some of the questions…

HCP: Oh… So it is “why do we want to choose this” I see I see.

KY: but maybe we introduce this at the end? like we just explain what is 12345?

HCP: No you can explain this because you want them to decide on this one first before your next question comes out. So this one and this one sort of doesn’t… I know what you’re trying to do, you’re trying to give them an example of how to pick, so maybe you have to separate it into two slides. then for example, how do you feel about the food, I like it, I don’t like it, then they pick a scale. Then the next one you can use the actual question… This one is the family physician right? But then for this one, they don’t need this anymore right?

KY: Yeah, the options will depend on the qualifiers…

HCP: Cause this one talks about how comfortable you are with the family physician regardless of the usual or shared care right? Trained doctor.. how comfortable are you with seeing this trained doctor meaning this…? How confident will you be..? ok…

KY: (explains previous system used for preference section and why we changed it) So initially we don’t really… We tried having all those scoring system to indicate, but a lot of them, even if they prefer [inaudible], they may still prefer to go for usual care… for example the cost, they might think that it’s cheaper for me but [inaudible] So what we tried to change is that they themselves go through the questions, and they can rate themselves at the end where they are. So like a quiz generator [inaudible]

HCP: so that means this one (scale) you put at the end so that you don’t get a bias. I think that’s not a bad idea. So, you’re asking them to base their decision on all those qns that they answer?

KY: yeah after they go through everything some people will be indifferent at the end

HCP: Maybe “what would your preference be” (last slide of section)

HCP: so this is a decision aid for patients to then decide whether they want to go “A” or “B” la…

KY: so these are some other resources that we included the links

HCP: erm.. what websites are these… the US ones are… I suppose these are a bit more breast cancer specific… ok I think that should be ok! Then you go back home is it? Ok looks alright!
