# Supplementary material for: Development and pilot testing of a decision aid for navigating breast cancer survivorship care
Source: BMC Med Inform Decis Mak. 2022 Dec 15;22:330. doi: 10.1186/s12911-022-02056-5 (PMC9753367; doi:10.1186/s12911-022-02056-5)
Supplement: Supplementary file 5 — Additional file 5. Transcripts and the final decision aid prototype. [file 12911_2022_2056_MOESM5_ESM.zip › Additional file 5/ID08_transcript .docx]

**Study ID**: ID08

**Interviewer(s)**: IC

**Date**: 4 December 2021

**Transcribed by**: GT

IC: Can you see my screen?

ID: Yup.

IC: I will be passing you the remote control. So, today we'll be going through the decision aid together, each page at a time, this decision aid comes with five key sections. As you are viewing each page and section, tell me out loud any thoughts that go through your mind.

I will also prompt you with some questions along the way as you navigate through the pages. So for example, I'll be asking you questions about the amount of information do you think that the amount of information is sufficient or the clarity of the information you think that the phrasing of information is understandable such as the jargons, is the language clear and how do you find the presentation of information such as the use of graphics and interactive buttons this kind of thing. So, do you have any questions before we start?

ID: Okay, can I just know that today's presentation right, it will be recorded. So, are you going to present it to anybody?

IC: No, it won’t be presented. We will only be looking through, or at least listening to the audio recording for transcription purposes, because we need to analyse the information from there. But the video itself we won't be using it. We will only be using the audio recording.

ID: I see, okay, so so is it okay if I just okay sure, you can go ahead.

IC: You can see if you can navigate through my screen because I give you the remote control. Is it working?

ID: Yup

IC: So you can read through this disclaimer and then once you're ready you can press the that I have read and understood the above.

ID: Okay.

IC: You have to press on this.

ID: Yes, I did. Not sure, keyboard is it? How does this thing work?

IC: Are you using your phone?

ID: Yes.

IC: I’ll press thisfor you first then let’s see the rest you are able to press.

ID: Okay. Ivy, can you hear me?

IC: Yes.

ID: What are we waiting for right now?

IC: Sorry, we started the decision aid already so you can read through this and then you can press the next

ID: I see. I have read through.

IC: Okay, then you can press the.

ID: The remote control?

IC: The arrow on the bottom. Can you press it?

ID: The remote control?

IC: No, the, can you see the

ID: The interactive bubble?

IC: Sorry?

ID: The interactive bubble? Is this the one?

IC: The arrow (repeated)

ID: The arrow?

IC: The arrow below on the bottom of the screen.

ID: Where is the arrow?

IC: The bottom, do you see my cursor? This one here.

ID: This one? No.

IC: Can you try pressing it? It’s not working is it?

ID: It is not working.

IC: It is not working. Never mind, sorry because we didn’t expect the phone thing then when you are ready to go on to the next page, you just let me know I’ll help you press.

ID: Okay sure.

IC: Then I’ll press to the next one?

ID: Okay. The cancer survivor. Okay I am done.

IC: So if you have any thoughts or questions this kind of thing you can just let me know.

ID: Okay.

IC: So for this part, for the cancer survivorship, what do you think about the amount of information provided? Is it too much, just right, or too little?

ID: I think it’s fine.

IC: Then was the information clear, is iteasy to understand?

ID: Mm hmm.

IC: Then the presentation wise? Like how it looks this kind. Poor, fair, good or excellent?

ID: On a scale of one to five I give you?

IC: One to four.

ID: One to four, I think four will be good. Do I have to click on them?

IC: So these have interactive portions so the bubbles are clickable.

ID: Okay. So I need to click on them right? Surgery okay. Mm hmm.

IC: So for this part, the amount of information?

ID: Good.

IC: Just right?

ID: Yup.

IC: And then the clarity of information?

ID: Good. Yeah it’s clear.

IC: Then presentation wise?

ID: I think it’s just nice.

IC: Three or four?

ID: Four.

IC: Then next section.

ID: Okay. Mm hmm. We are going back to the previous screen is it?

IC: We are on the other form of care.

ID: Ohh the shared care, I see. Okay can you go back to the… are we into shared care now?

IC: I’ll press onthe shared care.

ID: Yes please.

IC: This one is the shared care.

ID: Mm hmm. Okay. So the shared navigator will follow up every quarterly is it?

IC: Mm, yes. Every here and there they will follow up.

ID: What does the months indicate? It’s every three months is the follow-up?

IC: Every three months, yes.

ID: Okay, alright done.

IC: So for these two parts for the usual care, how was the amount of information?

ID: Okay.

IC: Just nice or too little or too much?

ID: I think just nice.

IC: Then clarity wise, easy to understand, understandable with some ambiguity or difficult to understand?

ID: Mm hmm, easy.

IC: Then the presentation, one to four?

ID: Good.

.

IC: Three? I’ll put three for this one?

ID: Okay.

IC: Then the shared care wise, the amount of information?

ID: Mm, good.

IC: Then the clarity of information?

ID: Yes.

IC: Easy to understand?

ID: Yes.

IC: Then the presentation wise, one to four?

ID: Four.

IC: Okay. So generally so far, the amount of information that is provided is quite okay?

ID: Mm hmm.

IC: Anything else you think should be brought up?

ID: Follow-up? We are on follow-up now right?

IC: As in any other information that you think we should include in this decision aid?

ID: No, I think just nice. What’s the difference, okay this clear.

IC: We also have this, just to remind people about what is the care navigator.

ID: [reading information out lout] Okay I am done. [reading information out loud] This is before subsidises is it?

IC: Which one? The cost?

ID: Before subsidised it says here.

IC: The cost will be after subsidy for the citizen.

ID: Mm mm okay.

IC: So this part the information is okay?

ID: Mm.

IC: Clarity-wise?

ID: Sorry, come again?

IC: Clarity.

ID: Clarity is it?

IC: Is it easy to understand this kind of thing?

ID: Yup.

IC: Presentation, one to four?

ID: Four.

IC: Okay. Then the next part.

ID: Sorry, come again? [looking through the slide] I can press right? Can I?

IC: This one, no.

ID: What is this? How do you feel about the food at the cafeteria?

IC: This is an example of the format of the questions that will be presented

ID: I see. But you are actually applying on the shared care right?

IC: Yes. We hope these questions will help the participants or patients understand or at least know which side they lean towards. Maybe I go to the next one you can see.

ID: Okay. I would say four

IC: Four for the first question?

ID: For both questions that would be four. Very important, number five. I think four for question four. Five would be number three. Number three for six. Number three for seven.

IC: So based on the responses that you have provided, it sounds like you are leaning towards shared care for this portion?

ID: Mm hmm.

IC: And then so wanted to ask you also are these the kind of factors that you would consider when you want to decide whether to take up shared care?

ID: Yup.

ID: Or at least the kind of factors that would influence which kind of care you will be taking up?

ID: Mm hmm.

IC: Any other factors that you think will be important as well?

ID: Okay, in terms of as you know side effects of post radiotherapy, there will be we are talking about arm swelling, lymphedatis, so for myself right I do experience that but it’s kind of manageable now because I have been through physio and a few exercises have been taught to me so I have been trying to do it on a regular basis but it does flare up whenever if I take a plane. So, after each flight right the arm swelling part is really unbearable which requires the basically we need to go to the physiotherapist for some relief. So currently the thing that I am facing right now is now that I do have encountered whenever I have a swelling I will still require a referral letter from the oncologist to fix an appointment for physio so I think this will be good if you can incorporate that into your shared care. As and when we need that, I believe I am not the only person that is suffering from this. We don’t have to go through the oncologist or probably this can be done either through the GP or it could be done from the main navigator that is the pharmacist.

IC: So that it will be more convenient for yourself as well?

ID: Correct yes. I am not too sure but this is the protocol of SGH currently the physio department requires a fresh referral letter every 2 months so I think it is something not really practical.

IC: Especially for more long term kind?

ID: Yeah correct.

IC: We will take that into consideration, thank you.

ID: You’re welcome.

IC: Any other factors that you think would help or influence your decision making for this kind of follow-up care?

ID: I think I am quite okay with it, I think that’s about it.

IC: So the information provided is just nice for this part?

ID: Mm hmm.

IC: The clarity wise, is itclear?

ID: Yes.

IC: Or maybe just a bit of confusion at the first part, the first page, this question?

ID: Yup. Maybe you should just take away the example.

IC: I understand, then presentation wise? One to four?

ID: Four. One to four is it presentation?

IC: One to four.

ID: Okay three. Ohh that’s the end is it?

IC: Yes, that’s the end of the decision aid. Thank you so much.

ID: I thought there was more then in that case can I add some more?

IC: Sure, hold on.

ID: So you are talking about the shared care right? If let’s say we are talking about survivor after three years right, this is going to be implemented after three years? Would it be three years?

IC: Yes, at least three years.

ID: What about the mental health aspect? I believe there should be some kind of support .

IC: So it might good to for example to incorporate or at least have an avenue for patients to be referred to those place when they need to.

ID: Correct, you are right.

IC: Sorry am I too quiet, can you hear me?

ID: I can but there is a lot of echo behind. I am trying hard to listen.

IC: I think it’s my laptop. I will speak a bit louder, I am sorry.

ID: No problem.

IC: Do you want to add anything else?

ID: I think that should be fine.

IC: We actually have this other resources portion so for patients who are interested to find out more about these topic and then they can click on them and it will lead them to the website with the information there, so something like this.

ID: Okay.

IC: Maybe I can just quickly let you go through, let you see what we have here.

ID: Okay.

IC: If you have any other topics that you think will be included, you can let me know as well.

ID: I think as for now it is okay.

IC: So generally the information provided is quite comprehensive?

ID: Mm hmm.

IC: And then is it easy to understand , the clarity-wise?

ID: Mm hmm.

IC: Presentation, one to four?

ID: Four.

IC: Some other follow-up questions, for this preference exercise, how did the exercise help you to better understand your preferences in follow-up care?

ID: Sorry come again, I can’t hear you.

IC: Sorry, how did the exercise help you better understand your preferences in follow-up care? In what ways do you think this exercise will be beneficial or helpful in your decision-making?

ID: I guess convenience because if it is to be followed up with a GP, that is close to my vicinity I think that it is very convenient instead of going to you know for example NCC which is not near to my place. Especially

IC: Sorry?

ID: Sorry, its’s okay. Go ahead please.

IC: No, I was just going to ask another question. So what were you saying just now?

ID: No, I was just trying to say especially when days that we don’t feel good I think that is really the benefitting part.

IC: Because the NCC is a bit far away for you as well right? Going there is a bit difficult?

ID: Kind of. Sorry your this research right is pertaining to NCC only or it’s going to be the whole of Singapore?

IC: We will be starting with NCC first but if the rest of Singapore would like to take it up then it will be good also. We will have to see how it will help the patients in the first place. Whether or not it will be helpful. Because if it is not helpful then there is no point in doing it. But if it is helpful of course we will like to implement it in a wider scale. If it is implemented, it will involve the NCC doctors and the community pharmacists so we have the for example Watsons this kind of places, we have the GPs as well, the polyclinic doctors, it won’t just be NCC per se but it will involve other healthcare professionals in this model.

ID: Okay.

IC: So how do you find the decision aid aesthetically? So like the appearance features, the colour scheme, choice of fonts, the font size, this kind of things. Do you think it is okay or do you have other preferences for this kind of things?

ID: I think it’s fine.

IC: How about the length and time taken to go through the decision aid? Do you think it’s okay, too long or too short this kind of things?

ID: I think should be alright.

IC: If we have a downloadable copy of this, would you think it is helpful?

ID: Yes.

IC: So will you revisit this decision aid for some of the information presented?

ID: Yup.

IC: If you could, would you use this decision aid to discuss about follow-up care with your oncologist?

ID: Yes.

IC: Any other thoughts that came into your mind while you are viewing the decision aid that you haven’t share previously?

ID: No.

IC: Generally okay so far?

ID: Mm.

IC: Okay, thank you so much.

IC: That’s it for the decision aid. So I will just need to ask a few more questions about the decision aid.

ID: Sorry Ivy, speak louder please.

IC: Sorry I will need to ask a few questions more. I will show you the questionnaire. Can you see the questionnaire?

ID: Yup.

IC: So maybe you can just let me know what you answers are to this and I’ll mark it here.

ID: Okay. Would you prefer a digital decision aid over a booklet? Yes. Were you able to navigate across pages and links? Yup. Was the decision aid interactive? Yes.

IC: So this part is the one I was asking you questions about just now so I have already marked it down.

ID: I see, okay.

IC: So this one.

ID: Sorry, which one?

IC: B2 onwards.

ID: B2 ah. Sorry because I am maximizing my screen. So we are talking about B2?

IC: Yes, B2.

ID: Find the preference verification exercise yes. I found the presentation of information to be slanted towards shared. Length of the decision aid was just right. Next is it? Under section C?

IC: Yes.

ID: I think I have commented right earlier. Would you use this decision aid to follow? Yes. Would you use this decision aid to recommend to other cancer survivors? Yes. When do you think will be the most suitable time to introduce to cancer patients? I think at least immediately after the treatment. What do I like about the decision aid? Convenient, I guess if it is cheaper that will be a better bonus.

IC: This is referring to the shared care model?

ID: Yup. Okay I think.

IC: Thank you so much.

ID: You’re welcome. Something wrong with your speaker is it?

IC: I think my computer is just a bit loud, sorry about that.

ID: I think no it’s your speaker because each time when you speak right there’s this like you know haunting sound at the background.

IC: Oh no okay, I will find a way to fix this. Sorry about that.

ID: No, just letting you know I think it’s your speaker issue. Okay alright so this will just be a one-time right?

IC: Yes, one-time. We might need to ask some follow-up questions later. But that one we will contact you again for that. Is that okay?

ID: Okay sure.
