# Supplementary material for: Development and pilot testing of a decision aid for navigating breast cancer survivorship care
Source: BMC Med Inform Decis Mak. 2022 Dec 15;22:330. doi: 10.1186/s12911-022-02056-5 (PMC9753367; doi:10.1186/s12911-022-02056-5)
Supplement: Supplementary file 5 — Additional file 5. Transcripts and the final decision aid prototype. [file 12911_2022_2056_MOESM5_ESM.zip › Additional file 5/ID03 - Transcript.docx]

**Study ID: ID03 Date: 07/11/19**

**Interviewer(s): ET & KY**

**PART 1**

ID: -read- there’s no decision that I have to make right?

ET: yes, for now, we are just going through the material

ID: ya its this, not that I have to make a decision right?... oh, this is something that you want patients next time to go through then come to a decision?

ET: yes, when we have the other option ready

ID: ok

**PART 2**

ID: so I’m supposed to just read and try to comprehend, is it?

ET: ya, so actually the first two pages that you read is our first half a section, which is the introduction on cancer survivorship, like the concept of that. So do you feel that you understand what it was saying? Like can you summarise what it was trying to say?

ID: yes, so, maybe we should do it (questionnaire) as we go along, easier.

ET: ok -questionnaire-

ID: Can go back one is it?

ET: yes can

ID: no, I think this is quite good, in terms of the timeline (first slide) because until you actually put it pictorially like that, we don’t actually see it like that, like its just one seamless… ya so its quite good to see the timeline, to realise that actually, ya we’ve finished this so here and that’s hence la, can delete for this research purpose..

ET: for this slide it’s trying to say that for survivorship, from the moment you are diagnosed, actually your survivorship journey begins already.

ID: ah ya, until I read this, I didn’t think that way.

ET: normally you would think like ”oh after treatment?”

ID: ya, so at least its quite a good way to frame the issue. And of course this one have to read through but it’s good that you have this pictorial thing, I mean surveillance and all these things so.. of course, I would want to read a second time kind of thing but ya ☺

ID: ok I think the colour coding is good

ET: You can actually click in to read each of the cancer

ID: actually, maybe the colour coding is better if it corresponds

ET: to the organ is it?

ID: as in if I call soft tissue cancer, then all the soft tissue here is best the same colour then if its womb cancer, if its green here, the womb can be in green la, anyway its not these colours in real life so to correspond it, its actually.. and of course the blood, I mean its blood veins so its better. -after clicking in- ya so you see, the soft tissue cancer here, the colour suddenly becomes the lung cancer colour, so for me its confusing [ET: the colour should be consistent]. Whereas I don’t know let’s look at lung cancer, then its actually the reversed. I mean if you want it to be very… [ET: more intuitive?] yes,

ID: sarcoma is cancer right? Is a type of cancer?

ET: so the term wasn’t …

ID: ya, I think when I… cause you’re talking about cancer right, I think is that like the first time it appears, the term sarcoma?

ET: ya, so its soft tissue cancer

ID: ya, but I mean to the lay person, “it increase the risk of sarcoma”, sarcoma is it another kind of cancer? Or do you want to say risk of cancer.. cancer of the blood vessels? Then you can put bracket sarcoma. [KY: it’s a subtype of cancer].. it’s a subtype of cancer right? So you can say increase the risk of cancer of the blood vessels, bracket sarcoma angio osteo whatever.

ID: it’s the same one… (ovarian and womb cancer popup)

ET: ya the ovarian and the womb, we put together because it is related

ID: but it’s the same one is it?

ET: ya, it links to the same page

ID: then you should just put one link because it’s the same page. Otherwise I will be toggling and thinking whether the two pages are the same. So if it’s the same, you just one. You can have two bubbles but one link.

ID: so which one occurs first? Late effects, right?

ET: the long-term effects occur first, cause it occurs during your treatment but they prolong [ID: oh they persist, I see] but the late effects come in after your treatment

ID: but the long-term effects start during treatment, is it?

ET: like you will start feeling, for example, fatigue and then it last even after you finish treatment

ID: ok, then maybe you want to tweak it because when I read it like that, maybe it’s the word persist, that means its long-term, that means its after treatment is completed, but this is the immediate… this is the one that… I am trying to understand which one comes first. Actually what you mean is side effects that surface during completion… during treatment and persists beyond then its long term right? But effects that manifest after treatment completion. but it is like immediate? Like after the whole completion, after the whole treatment or after each session?

ET: After your whole treatment [ID: you mean the whole treatment] so like some time in the future, like if you start experiencing for example dysfunctions..

ID: so maybe this one, you can also do it like the graph, like the timeline, so the timeline is like, long-term effects then you have your time line like during treatment and persists beyond, you can have your time milestones [ET: so its easier to (visualize)] ya. So here because when I read like that im trying to understand, manifest after but like how long after, immediately after or you know, late is like subjective right, is it like 2 months later or 1 year later? anything is after the treatment what. Ok.

ID: so, this one I can also click? (treatment bubbles)

ET: would it better to have an instruction here saying you can click in like the previous slide?

ID: yes! Like maybe a.. I don’t know, ya, but I guess if you mouse over, its ok. But its not like “click for more info”? I think earlier slide there was something like that [ET: ya, there was a click for more info]

ID: so this is part of the educational info? [ET: section, yes] which I think is useful. Like for me when I read it [ET: is it new information?] some are not say new but it kind of like categorize it. Like I’m always asking him (Dr) about tingling but when I read this then I know “oh, its about late-effect, its part of late effect” so I think in that sense it helps, kind of normalizes the experience of the patient. Because without knowing that.. ok I don’t know what is neuropathy but without knowing that actually tingling is erm.. [ET: part of the effect], ya maybe even years on, you still… so this I know it’s like late-effect then ok la, I mean it normalizes… eh? This one cannot click, oh must close first (unable to directly click on next bubble before closing slide)

ID: what’s this? (AI)

ET: AI is a class of the drug, so its aromatase inhibitor

ID: AI is the class of drugs?

ET: ya, so these are examples of the drug [ID: but what does AI..] and it stands for aromatase inhibitor.

ID: oh, you should put that (full spelling of AI), cause I thought it’s a spelling error, I thought you meant “ALL” late effects, but I mean I don’t know what is AI so if you want the lay… what did you say it was? [ET: aromatase inhibitor].. aromatase inhibitor ya…

ET: would it be easier if we just put the drug names? So as a survivor, you can [ID: which is the drug name?] anastrozole, letrozole, or exemestane.

ID: like I know this (tamoxifen), because I take it. but I don’t know this and this and I definitely don’t know what is AI. Oh so she was asking me about this this this this this this is it? this is the group of drugs is it? I see.. ya I guess… I just don’t know what is AI so if you can explain what it is, so.. it will be (helpful) ya.

ID: but I think all this is useful. It kind of explains what is happening so its useful.

ET: yup, so you are done with step 1 right, so -questionnaire-

ID: erm, I guess you should always use the.. I don’t think its too little I think its just right and if people want to know more, maybe you want to have an option for further reading. The font and size of font are readable.

ID: typo… (section 2 slide 2 table – you* will be seeing)

ID: this is a new model la (shared care)

ET: actually, you can also click on all the HCPs

ID: oh so we don’t know, you have to put that at every prompt.

ID: is this the GP? (table slide)

ET: yes, the GP and polyclinic doctor that were mentioned earlier

ID: pharmacist, currently the pharmacist don’t play much of a role except to prescribe medicine right?

ET: to dispense the drugs

ID: this is a … a breakdown of what we read is it?

ET: yes, it’s a more detailed breakdown that shows you who will be doing what

ID: what do you mean by.. this one.. actually what is comorbidities? I saw it somewhere just now, at the primary care physician… what is it actually?

ET: it is other conditions that you may have, for example diabetes, high blood pressure, high cholesterol

ID: ok, so you may want to explain that more lay

ID: how does the two tick here mean rapid access to oncologist? You’re saying that they give me more rapid access? I don’t understand this part

ET: so for example, because we said that these 3 people will be looking after your health in the shared care model, so when you go and see your, let’s say polyclinic doctor, then if there’s a need to they can immediately refer you back to the oncologist. So they are incharge of doing rapid access.

ID: oh they can escalate your consult with the oncologist?

ET: yeah because they are in contact with the oncologist. So for example now, if you see your polyclinic doctor outside, but they are not fully aware of your cancer history, like what you went through. Then they cannot really access..

ID: then what you mean is that they will facilitate rapid, giving you a rapid access to oncologist, not so much on… because im wondering if I see my physician, the whole point of seeing the polyclinic physician is to share the care so that I don’t have to keep coming to cancer centre. So when I see this I thought ok, but im seeing him (pcp), why is it rapid access… so actually what you meant is you will help to facilitate a faster access to oncologist if necessary, right?... So the navigator is the pharmacist? (table) should use the same term pharmacist navigator… how come the pharmacist… oh so they don’t prescribe, then what do they do? They only assess toxicities ah?

ET: in this part, there’s more

ID: ok so maybe you should use the same term

ID: what do you mean by done at the oncologist side? You mean done by the oncologist?

ET: no, so its done at the cancer centre but it doesn’t have to be specifically by the oncologist, so other people can help them for example the supportive care nurses. Because its only for this item, which is the development of your survivorship care plan

ID: So you mean done by the oncologist team? Ya, it think its clearer because what do you mean by oncologist side? That’s not quite correct English... you don’t mean site right? [ET: no, side], on his part, so it should be done either.. but if its meant done by him? But you say not necessarily by him, so by his team la, the oncologist team, oncology team or whatever. Rather than side.

ET: would it be easier if we just removed it and left it as the oncologist

ID: but its already here [ET: without the asterisk], but what you wanted to say is its by a team la … you can say done by the oncologist team but…. I think its fine im just wondering what is oncologist side. Phrasing ya.

ET: so actually for this part instead of the link, we did a pop-up so it’s easier, you can immediately see [ID: ya I think its easier] like the cost

ID: and all this coloured one, the colour for the tables, I think its quite easy to read

ET: so you don’t read through (to) the wrong line

ID: ya its quite good to do it blue white blue white (cost slide)

ID: same? (ncc price)

ET: ya cause its both the oncologist so for this it’s the polyclinic

ID: then actually…

ET: cause for the shared care you’ll be going to both [ID: you also see.. I see]

ET: would it be easier if we didn’t put click here to find our more like its the same [ID: then how?] we put a description saying that it’s the same as usual care so you won’t have to click twice

ID: maybe what you can do is that since these 2 are the same, you should have a merge line for the two, then you put this up (polyclinic price). So then, its like a table right? So you should do the same as just now that one, the colour coding for the price. So that I can see the. first line is based on the number of clinics. Second line is this. So the second line will be empty for this part(usual) then the third line is merged. Either that or you put this above, you know what I mean? Anyway there 2 points and 3 points, so you need to merge this then you click one link, by virtue you of the row being merged, you know that it is for both.

ID: so it’s the same right? Like this is the same right?

ET: ya, cause it depends on the personal..

ID: ya so it’s the same likewise, what I mean, you draw a table then this one is actually a merge so it applies to both. Then those are specific to each, you can put it.. [ET: for example lines or colour difference] ya like this one is obviously going to be cancer centre, then this one shared care can be either polyclinic or GPs then you will obviously have 2 boxes

ID: I think this is too close (spacing of words in table)... like distinguish between the 3 columns

ID: so are you saying that this is a pro? this is a con? Are you saying? It cannot be a pro right, this one(usual)? It can only be read as a con (last comparing row)

KY: some patients they may not want to liaise up their GP with their oncologist [ID: ya but actually is it a con or a pro, that’s what im trying to understand].. some patients may treat it as a good thing in the sense that they are compartmentalizing their [ID: oh I see] so we don’t want.. when we develop it we don’t really want to have bias

ET: but the phrasing makes it sound like it’s a con or it’s the way you interpret it?

ID: for me I would want my healthcare to be integrated, for me. Unless there’s a good reason to compartmentalize it which… [ET: it’s seldom the care?] ya for me when I read, because of that mindset, I read it as a con… obviously you’d want all your healthcare providers to talk to each other so…

ID: so.. I don’t really understand this part, a bit of a mouthful, cause, I don’t know what is comorbidities so I have to go back to find out…

ET: so what it’s trying to say is that now you’re seeing only your oncologist, you don’t have any other doctors, if let’s say in the future you have high blood pressure, these things will surface when you see your oncologist so they will only be managed by your oncologist or like currently if you have a personal primary care doctor like personal GP, they will be managing your HBP but without any background context or much information about your cancer history

ID: I think for this kind, that’s why I keep reading it as a pro/con thing, I think it is ok to say the feature objectively, then like the limitation to the doctor is obviously you cannot choose your doctor or like the choice is limited (shared), that must be a con right? So because this is a decision aid, in reading all these, I keep thinking there is double negative, there is negative, I see.. its like how people want you to switch the electricity plans, so they have a tick tick tick tick feature, one glance I see more ticks for (an option).. so you need to craft your characteristic of that whatever (options) in such a way you have either a tick or no tick, so maybe you put “choice of doctor” then under usual care you tick “can choose”, no tick or better still, cross = cannot choose. So one look, I can see “oh if I go by this way I can do this I cannot do that”, but here, im trying to figure that out. double negative, may not be optimal so if its optimal… so its a bit hard… so if you want to do that, I think this is part of the consideration. so you need to basically draw out that element.. like “more frequent meetings with oncologist” tick, then the other one will be less frequent… oh it has to be.. phrased it such that its tick or cross, then one visual glance you can see.

ET: so less reading and more visual?

ID: Because I can read but [ET: its easier to understand?] ya so limitation, no then im trying to see but I want to choose my doctor, then it will make me not want to [KY: do shared care], ya but of course you can have other things like.. I mean if you think that there’s some.. depending on your preference, some people may be ok not to choose their doctor, that kind of statement, because not all choices are… like you say some people like to compartmentalize for some reason, so you may want to put certain statements to reflect that depending on individual preferences, just like how some people don’t mind going to private, but it comes as a cost, that kind of thing.

ET: -questionnaire-

ID: bearing in mind that this is a decision aid, so after I read, im not sure how it… it doesn’t aid me to make a quick mental note of the decision. But I think it is good to .. the content is there and its good for the patient to learn about these things.

**PART 3**

ID: this is my situation now? so I have to click.. oh write here ah?

ET: because its not a website, but in the future we want to make it more interactive…

ID: -read qns and clarify what to write-

ET: ok for this it’s a scale (smiley face) because for some people, they don’t want to strictly go by numbers, they want to be able to write (in-between)

ID: -how important is the type… type ah?

ET: like does it matter if it’s a specialist or polyclinic…

ID: Do I need to like… no need right, just on the scale?

ET: ya

ID: so how should I do this?

ET: maybe you can just write a percentage for each qns

ID: ok I’ll just write a percentage

ID: how favourable are the cost-savings associated with appointment with polyclinics… ok this question is a bit convoluted. how favourable are the cost-savings… that means you save more at polyclinics and how important is that for you is it?

ET: yes

ID: ok… more convenient to go to polyclinic… but this is like independent of the.. this is just convenience right? It’s independent of like “I want to see an oncologist” but actually the polyclinic is more convenient.

ET: ya its independent

ID: ok ya, so obviously a polyclinic is more (convenient)

ID: I’m not sure, can I answer B?...

ET: do you feel that a question like that doesn’t really help you define like which option you’re leaning towards, because like you said, sometimes you’re not sure if..

ID: because I’ve actually haven’t been to a polyclinic much, for this journey... so, there’s no experience to… right? Because it’s not like we have used this shared care and then we have gone to polyclinic then we can compare. So, actually I cannot compare but in terms of the appointments here (NCC), the ease of changing is quite easy, just make a phone call. So its also not meaningful for me to [ET: answer this question?] ya.. [KY: it’s ok to leave it blank]…

ID: patient navigation, ok, this patient navigation, you have to define it a little bit more.. you mean the… first, I don’t know.. what does the.. I’ve forgotten from the slide what does the pharmacist navigate for the patient… what does it do [KY: help to toggle between…] -reads navigator’s role- so is this going to be like the new role of the pharmacist? Because its different from what we are [KY: the usual role right? We’re trying to get them just to expand a bit to actually serve as navigators cause these are actually community pharmacists so actually...] so I think for purposes of this brochure or whatever, you need to paint the current.. currently, pharmacists do this, moving forward for this model….

**PART 4**

ID: ya, I was saying… so I think it is useful to.. for this… I don’t know, this is my view because it’s a new model that we are trying to get people to consider this new model or embrace this new model. Then you need to paint the current. Like, currently, oncologists solely do this and then what is the effect.. maybe longer waiting time etc. then to me, currently, the pharmacists only dispense my medicine. So for me to understand how are they going to navigate my care in this shared care plan, I got to think a little bit longer

ET: it’s not easy to imagine it?

ID: yes, because even for me, its difficult, let alone old aunty / uncles. So, I think it helps if you paint a before and after kind of thing… so coming back to this…

ET: do you feel that because you don’t understand it, you can’t really rate it?

ID: ya, because I can’t. because for me… ya, I can’t really rate it because I don’t know what it is.

ET: so having gone through just now what you’ve read, so they (pharmacist) will follow up with you, remind you of your upcoming appointments, do you think a service like that is favourable, do you think it will be important to you? Like at least you have this person you can contact.

ID: I think it is important to target groups of people, for this part I think maybe for the more educated, they don’t really need to be reminded of appointments, but maybe for senior citizens or for people who… so maybe there could be an opt-in / opt-out. But I think it is, of course, always useful to be reminded.

ET: so basically, it’s your personal value, if you feel that for yourself, it’s not so important then you can just give it a low rating…

ID: but then the question then would be “how skilled is this pharmacist? what kind of value-add can he give you?” because he’s a pharmacist, I would not trust, I mean “trust” in a very loose way, a pharmacist for oncology-related consultation questions. Like, I would not bother to.. actually sometimes its different. Like when I ask an oncologist, “what would tamoxifen do to you?” his answer may be different from what my pharmacist here will tell me. He will tell me a very medicinal thing but the oncologist question to… even my gynecologist will give me a slightly different answer because they are skewed that “oh tamoxifen will increase your womb cancer thing” but my oncologist treating my breast cancer will not tell me that because he will scare the shit out of me. I mean this is from a personal experience. I ask different people the same question, I actually get different answers. But the answer I would trust most is with my oncologist. Let’s say it’s the same thing, I also ask my gynecologist in KK the same thing about tamoxifen, but because she’s a gyne, she’s looking from the womb perspective, so she gives me a different thing (answer) which troubled me a bit. I had to come back to ask my oncologist who then… so let alone ask the pharmacist because the pharmacist will give you the pharmaceutical (perspective).. I mean this is our general concept of a pharmacist. Of course if I ask him about how many times I should take, when I should take, I would not bother my oncologist with that kind of question, but I would ask (pharmacist). So therefore, im not sure how to answer this and Qn 11.. so actually I’m more inclined to say… but actually I would appreciate if I can get my tamoxifen in my neighborhood clinic. I don’t have to come here to get my tamoxifen, you know what I mean? [ET: cause coming down all the way then] ya I always come here then I have to take it. of course now they have all these medicine delivery and all that but you’re talking about navigation in that sense. I think in a community store, its ok. [ET: but it’ll be better if you can even collect your drugs from there?] ya, in fact I need that more than his (pharmacist) advice to me about the condition which I will trust the oncologist more, I mean this is my view.

ID: -reads- what’s this sorry? Answers to even numbered questions…

KY: -explains-

ID: can you like.. you need to revise it such that… nobody is going to understand this, so you got to.. you know how they do those grammar or IQ test, or whatever? So basically you need to [ET: a score will just appear?] ya, you compute already for them, when they put 75%, the system will compute for them and give them a score/ outcome which has a certain characteristic. So maybe there are 4 outcomes, A B C D, so if my aggregate score, whether its even or odd number, I don’t know, my aggregate score reaches a certain target, it puts me in this category of people who think this way.

KY: then in this case, do think this kind of scale, we only define the two ends, and then we leave the in-between as blank, do you think this is better or do you want us to have like sub-categories? [ET: like just numbers]

ID: I think if you don’t have sub-categories, you leave it very much open to me to suka suka, but I think its useful to still put the markers like “very important”, “somewhat important” whatever but they should be able to.. [ET: choose in-between?] choose in-between. Ya the problem with the markers is that I either strongly agree or I agree. But maybe I have a between the “agree” and “strongly agree”. But I think its ok to have the ruler to allow people to put on exactly whatever percentage but you still have the in-between markers to guide them as a frame of mind. So you still need to indicate here, neutral or something or somewhat important, you know? Something like that. [ET: so more guidance..]

ID: -ask about developers of PDA- I really think like… because I currently do not know what my GP can do for me, so you need to paint the current. Like, currently.. [KY: usual care part?] ya, like what is going on now. then, what are changing. I suppose things are changing so what is changing, what the difference means. Of course we must change for the better, so, the change must mean good things for certain people and do away with the disadvantage or whatever, so you need to point out very specifically, like the concept of shared care is good because I can pop-in to a polyclinic anytime, that’s the whole idea, right? But for the common thing, or maybe even I don’t have to come here to see my psychosocial counsellor, I can clock-in to a polyclinic psychosocial counsellor to… and then that person would know what I’ve been through. Whether I go here go there, the communication is shared. It’s integrated, so they will know that “oh, last month I came for one session here, I met with whom, but for whatever reason, maybe next month I meet with someone else” so in that sense, I think it’s not… how should I say, so the spreading out, decentralization of the care is not a bad thing. But for certain pertinent examinations, I will come back here, like the mammogram. But even mammogram if I can do it in polyclinic, it’s fine, so long as the information is shared holistically and effectively such that the same quality of care is still being dispensed then its ok. Cause definitely, I’d prefer to collect my medicine from somewhere near my house then coming here, cause not everyone lives in outram. So that part.. I think.. so maybe there has to be a suite of different... you can’t do away completely with the oncologist, I mean for me personally, I value my visits to the oncologist a lot because I don’t see him (dr) often, so when I see (him), it has to be quite targeted, but the other things that I don’t need him to do, like maybe this kind of lifestyle, diet kind of thing, you can flow down to the dietician part, the polyclinic dietician part.

ET: -questionnaire-

ID: what’s the values part, sorry?

ET: -explain and continue with questionnaire-

ID: im not sure if “values” is the right word. [KY: Preferences, maybe?] preferences… ya, because this one im trying to think values.. my values is about.. but what you mean is preferences right? Is it convenience, is it cost? Not so much values…

ET: ok so maybe we’ll change the phrasing to preferences

ID: or like… what is important to you is not exactly the same as your values. I mean in this context. I’m a thrifty person, that’s my value but it doesn’t mean I would not pay for quality care, so its not exactly synonymous.

ET: so it can be like “your priorities” or “your preferences”

ID: preferences and consideration, so it can be more... like is it budgetary, is it distance, is it quality of care. Its not exactly.. values is a bit vague, values is about honesty nothing to do with.. but what I think you meant is my preference, what I would prioritize over this.

ET: -explain context for section 4- do you feel that the video was comprehensive?

ID: I mean its ok but its not like the.. are you supposed to… like its not for you to decide whether you want to do this or that right? [ET: -explains-]

ID: I think it does tell them a bit… but are there any specifics that can be included?

ET: what do you mean by specifics… actually after watching the video, the next slide is a summary of what was covered, the key information [ID: like this?] yeah, so it’s the same information

ID: actually I feel it’s the same. You have a table right? I don’t need to watch a video to… I mean the video is giving me one scenario at a time but your video is … but they already selected, so if im one or the other I watch the same video?

ET: only those who are selected for the shared-care will watch the video, those who are not will [ID: not watch anything?] yeah, just continue seeing their doctors

ID: so what’s the purpose of the video?

ET: its to help them understand better what to prepare for the next 12 months [ID: to understand this?] ya, what will happen, how will their care be different from what they are currently used to.

ID: then I think it’s ok.

ET: -explain last section-

ID: but these are.. this is international sources la, its not our own local… [ET: -shows local pages-]

ET: do you feel that we should only put the local ones?

ID: I think its ok (to have international one)… but they are quite trusted resources right?

ET: do you think we can improve on the labels? For example, some people want to look for specific things then we should put a sub-category and the link there

ID: is there a sub-category?

ET: for example like you might want to find out on more information on, let’s say, if you are taking tamoxifen, what else can happen other than the conditions here? So we’ll have a link to more information on tamoxifen

ID: ya I think definitely, the problem about us searching is like, you talk about the online resources. I’m not sure how accredited it should be because it’s coming from a hospital, obviously, the hospital would want to provide certain accredited resources. So I’m not sure whether it should just go to a generic website, or it should be…. -refers to slide- oh so it actually goes to the NCCS website [ET: the organization website, so the five links are actually -shows organization-] ya definitely the sub-categories would help, at one glance, I know where I can find out about Tamoxifen. I mean for now, I don’t know where to find my first reference source to find out more about tamoxifen. actually I don’t know, let’s say today I miss (a dose?)... but I will always be inclined to go to the NCC one (website) or anything I find on the NCC website that is hyperlinked right? I won’t go and anyhow read some US.org don’t know what.. ya so in that sense that sort of accreditation, like if you list certain trusted websites that, for some reason, the hospital knows that even the things there are used by practitioners, it helps.

KY: do you think we should also add on to the list about, you mentioned, the information about tamoxifen. So do you want more information about the drugs?

ID: ya, even the types of cancer, is it the hormone receptive or HER 2 [ET: the different diagnosis of breast cancer?] or ERPS, ya, and what exactly is a lumpectomy, mastectomy, that kind of considerations. This one I think the video… is it a video? It’s usually not a video, right? It cannot be a video.

KY: the 10 steps (self examination)

ID: I guess a video is not suitable. Ya I think all these will help (websites)

-end-
