# Supplementary material for: Development and pilot testing of a decision aid for navigating breast cancer survivorship care
Source: BMC Med Inform Decis Mak. 2022 Dec 15;22:330. doi: 10.1186/s12911-022-02056-5 (PMC9753367; doi:10.1186/s12911-022-02056-5)
Supplement: Supplementary file 5 — Additional file 5. Transcripts and the final decision aid prototype. [file 12911_2022_2056_MOESM5_ESM.zip › Additional file 5/HCP05_transcript .docx]

**Study ID**: HCP05

**Interviewer(s)**: IC

**Date**: 19 November 2021

**Transcribed by**: GT

IC: I will be going through the decision aid together, so one page at a time. This decision aid consists of five key sections. As you are viewing each page and section, you can tell me out loud any thoughts that go through your mind and I may also prompt with you some questions along the way as you navigate through the pages. So, some things that can mention about the content, how accurate the information provided is, how about the language do you think is too much jargon or if it is okay, the appearance how does it look and then how feasible do you think it will be for routine use in clinical settings. So, if you are ready, you can start.

HCP: I click on each of them?

IC: Mm, so you can start with the first one.

HCP: Then I go back to home?

IC: The next section so like for this kind do you think the information is okay?

HCP: Yes.

IC: Or do you think there’s more information that should be provided?

HCP: I think it looks quite (repeated) simple and easy to understand. The words are also quite okay. So I go back again previous section to have a look. Very nice that there is a lot of smiley faces. The physical I don’t know why the face is so red, with red blotches is there a reason? Do they have things on their face, that’s why you make them? I don’t know whether my thing, normally the only thing you may get this is from hot flushes from the treatment right so I don’t know whether a nicer physical picture could be used. I think it is okay, emotional. I think this page, you are seeing the same page as me, is it?

IC: Yes, I can see.

HCP: This is fine. So next section…minor thing you are doing American spelling is it or British spelling for lymphedema?

IC: That one I would need to ask the…

HCP: It is just me, it’s up to you if you want to standardize. A lot of people do American. I guess surgery site is more layman as opposed to surgical site or site of surgery, not sure. Maybe at surgery site the English doesn’t sound as refined maybe at site of surgery. So, this minor details because I am a surgeon so I think most of is I think image, numbness I think that is okay. (unsure how to close the box)

IC: You can press the close the close the X button on top.

HCP: Is there a reason why chemotherapy comes before surgery and chemo? Because in the sequence of events I mean depend right patients either have surgery alone or they have surgery, chemo then radiotherapy? Or unless they have surgery, radio but no chemo so I don’t know. It’s fine fine. Lymphedema again. Discolouration is British spelling right, lymphedema is American spelling, minor thing it’s already so good that I am just picking up little things right. Do we have numbness and weakness from radiotherapy?

IC: Mm.

HCP: I thought that is more for surgery and chemo. Maybe I am wrong. I think this is very vague, the sentence increased risk of heart diseases especially if lymph nodes are radiated. I thought the risk of heart disease depends so it is only I think it is mostly if it is a left sided cancer if you are aiming here. Because lymph nodes you can have lymph nodes behind the chest wall, you can have lymph nodes on the armpit, lymph nodes up here so I think radiotherapy could have said lymph nodes on armpit shouldn’t increase the risk of heart disease. If you radiate the right side, it shouldn’t have but if you radiate I think more so if the left sided cancer I mean that’s what stands out to me. Maybe just double check with Dr Rose. Maybe you want to have another star for the risk of soft tissue cancer. The risk of lung cancer you are trying to assure them that it is low risk. Actually, it is not that low after all thirteen in one thousand. Actually, the soft tissue cancer is even lower risk, maybe you put the lower risk then if I am a breast cancer survivor I will freak out a bit from this.

IC: So at least to reassure them that it is not…

HCP: Because if you bother to put the risk for lung cancer, then the soft tissue why not right put the risk as well I think. You put the risk of leukemia also so that last one wasn’t. Although I am not sure whether we have the risk part. Ohh that’s it. Tamoxifen is not supposed to affect your bones. Not as much as the opps not as much as the Letrozole. The risk this one actually affect the bones more. Quite interesting the oncologist is stressed out like the surgeon, it is actually all of us all combined right? The surgeon and the medical oncologist all combined one.

IC: Mm.

HCP: Actually, the nurse practitioner comes in also after the usual care we have the Sister Mabel

IC: Mm mm.

HCP: the breast what do you call that advanced practices nurses so I don’t know whether they need to be included here under the usual care model we have here too. They run our survivor clinic. So, is this supposed to mean like say if the surgeon will see at month three, see the medical at month nine or this is just an example throughout that one year?

IC: It’s an example of how the follow-up schedule may be for a patient but it will also depend on the patient itself.

HCP: Because when you say month zero my first thoughts is this for the first year of treatment starting from month zero right or is this supposed to represent I guess under the context this probably means under the survivorship years right how often we see I think one can be interpreted both ways I think when trying it’s quite clear and poor radiation oncologist is out of the picture already.

IC: You can look at the shared care one.

HCP: Ohh I haven’t click on that one yet. I don’t think it is necessarily Western countries, I don’t have the data offhand. Some I think Korea also after that the patients are discharged from the hospitals so if you want to make the patient feel that is not just a Western thing. Internationally cancer patients are also discharged into the community actually. It’s not just a Western thing. This comes under shared care, right?

IC: Yes.

HCP: So, the pharmacist will be the navigator?

IC: Mm.

HCP: I was not aware that…

IC: So, this is the model that we are following from some of the studies that were held in I think Australia or America.

HCP: Why the pharmacist? Is it because they are giving the hormonal drugs is it? How about patients who are not on any medications? Why would the pharmacist come in?

IC: That one I am not very sure but I think it has something to do with it is more accessible to the patients because like for example if they live in America or Australia then in the community it is easier to access those kind of like their version of Guardian.

HCP: I agree, but how about here? Our pharmacist not that…

IC: So that’s why we want to see.

HCP: Not really the navigator here right do they?

IC: So we actually are currently running one in the Singapore context and then we are working with Watsons for that actually. So we engage the Watsons pharmacists as the care navigator.

HCP: I mean maybe majority of the population do go to pharmacy. Just that I work here so Ia am spoilt right I just go straight to once I need. Would the care navigator be in the middle navigating and coordinating everything? Looking at this looks like the family physician is, the care navigator is quite far away from the oncologist ot I don’t know this is just minor details right?

IC: Mm.

HCP: But they are all three so that means the oncologist and care navigator are also in communication, right?

IC: Yes.

HCP: They are not it is not the oncologist will tell the family physician, will tell the care navigator maybe a triangle? I don’t know because it is three way right or like a circle, triangle I don’t know.

IC: Because now it looks like…

HCP: It is very linear the care navigator is at one end, the oncologist is at one end. To me when I first see this, I think that the family physician is the one coordinating in the middle liaising both. I think it should be the survivors who see mainly the medical oncologist (repeated) and not the physician and not the pharmacist but I think what you are trying to say that survivors mainly see the medical ones not the surgical and radiation oncologist so it’s just the other way. So actually hormonal treatment is outside the (repeated) initial treatment? Cause when you say three years out of treatment the patients are still on hormonal therapy right?

IC: Some would be, some would…

HCP: So, the hormonal therapy doesn’t come under the treatment?

IC: Ya.

HCP: Three years out of treatment is out of surgery, the chemo and radiotherapy.

IC: Yes.

HCP: So they never meet the navigator? The navigator is over the phone?

IC: So, they can meet the navigator in person as well but usually the idea is that it will be over the phone kind of thing.

HCP: Just a minor thing, so if let’s say they meet at month three and month nine so when does the mammogram come in because a mammogram is once a year only right?

IC: Mm.

HCP: Then maybe any idea when it comes in. Go back to options?

IC: Mm.

HCP: Then the next section?

IC: Yes.

HCP: If it is just usual care, we also communicate with each other. Not that distinct. We do right but I think the communication may not be as much as shared care. We don’t communicate, but if there is a problem then we communicate. Care navigation question mark, what is this question mark? I click on it. Actually, the usual care is oncologists right with the medical and surgical not alone, right? So, the navigator is free of charge is it when they call them? You mean have to pay for the navigators?

IC: For the program that we have now, no.

HCP: So, for the navigator it’s out of freewill or they are getting funding from somewhere?

IC: Funding from somewhere. So, for the patients’ side they generally only need to make payment for the consultation.

HCP: I don’t understand, so I am supposed to show my preferences usual care and shared care but the question is one, two, three, four, five related to the questions.

IC: So, for these questions it’s to see which side they might lean towards. If the patients are not confident in the family physician, then it sounds like that they might be leaning more towards usual care.

HCP: Ohh one or two then five is towards shared care?

IC: Yes correct.

HCP: So, I answer this as a doctor or as a patient?

IC: So, this one is for the patients to…

HCP: So, what do I do? Do I answer them too? Or don’t need to answer, I am just reading the questions.

IC: You can answer also.

HCP: No, I am just looking through the questions.

IC: So maybe for this part you can let me know like based on your interactions with the patients would these be questions or factors that might pertain to their decision making also?

HCP: I think they will be concerned about whether the family physician is trained although there are a few they would rather see because they don’t want to come to the hospital so much. I think if they feel that they are trained then they should be quite comfortable. The other one is the rapport that they have with them all this while. I think communication is very important. The e for shared care is gone.

IC: Yes. So, like do you think there are other factors that we should include for the patients to make their decision?

HCP: I guess the other questions some may feel I don’t know if the patient may think about it but as doctors if they do have something let’s say if they pick up can they come back quickly to the hospital? Would they still receive timely care? Will I still be able to see my usual doctor but we don’t want to promise them the usual doctor also it is subsidized and doctors leave. Then I guess at some point the shared care the mammogram is still done in the hospital is it or community? Then of course whether it is easy to get the mammogram is it quality read. If there is anything, do I trust the mammogram there or trust here? Next section is it?

IC: So this is the end of the decision aid.

HCP: So this will be given to the survivors for them to take a look?

IC: Yes.

HCP: So, they have to answer on the spot or something they scan at home the QR code they answer on their own leisure or it’s in a booklet form or what?

IC: So we can have a booklet form for them so a physical copy or we give them the link or at least the doctors give them the links to this kind of decision aid. If not, another thing is we hope that if it is used in the routine clinical settings then maybe the doctors can go through with the patients themselves using this kind of decision aid.

HCP: I think it takes a while to read through right? So whether they can maintain their… Will it be in English only?

IC: Currently we only have the English version maybe in future we might translate to other because we also understand that not everyone can read English. So these resources will link to online links.

HCP: So this is the which country they are from is it?

IC: Yes.

HCP: America flag the line come down the red line.

IC: They turned it around.

HCP: The flag looks a bit off right?

IC: Mm.

HCP: Have to think for a while what country flag. Just open them is it, the same thing all the flags a bit. (Clicks on the link)

IC: This is the intranet one right so cannot.

HCP: No problem.

HCP: This one is just the formatting thingy?

IC: Mm.

HCP: Looks slanted. I think it is very nice, it’s very comprehensive.

IC: So overall, are there any other thoughts that came into to your mind when you were looking through this?

HCP: I think I voiced out my thoughts along the way. I think it is very nice and comprehensive.

IC: How about the appearance? The interactive parts so do you think that it is interactive enough, is it easy to navigate through?

HCP: I think it was, the only thing is sometimes I think halfway went through do I go back to home or next section but I think it is okay. Very nice colour scheme also.

IC: And then how difficult or easy do you think it will be for you to introduce this kind of decision aid to your patients? So, for example we mention providing them with the links or physical copy of this?

HCP: Should be quite easy.

IC: And then will you be comfortable discussing the information that we presented on the decision aid with your patients?

HCP: Yes.

IC: And then…

HCP: Was there one part do you have any questions you want to contact somebody was there something in the end? There was no like if let’s say after doing this they have questions do they go back to the doctors. After reading you will have a lot more questions right?

IC: Maybe we can put it into the conclusion.

HCP: Under the conclusion if you have any further questions actually at the bottom right after reading if you wish to speak to your oncologist about your suitability, it’s there. If you have any concerns.

IC: And then what feasibility aspects of the decision aid should be considered or improved before it is rolled out to the patients?

HCP: Back on this page right maybe what matters to you can you put a question mark? I didn’t realise this part is going to be a survey already. What matters to you also after this. You want to make it more personal, we want to know what matters to you. I think it is okay. I guess you really have the spectrum on top you really want to push it then you put the one, two, three, four, five in different colours from yellow to purple but I think it’s too much. Don’t need (repeated), but if you really want to put the survivor thing on top it took me a while I ask you what does this means I realized after you explained to me I think it is quite okay. It’s quite clear.

IC: Okay then I think that’s it. Thank you so much.

HCP: Thank you.

Part 2 of recording:

HCP: But I guess not everyone is suitable for this shared care model right and I think I guess we will only show patients the doctor if you feel that you are suitable then you will show them what will you tell them about it is it?

IC: Mm.

HCP: So, I guess maybe the patients have to feel that they are also suitable for this, medically I feel assured that my doctor has told me I am suitable for this you know what I mean not like because some of them have very complex cancers and they are definitely not suitable for this model at all.
