# Supplementary material for: Development and pilot testing of a decision aid for navigating breast cancer survivorship care
Source: BMC Med Inform Decis Mak. 2022 Dec 15;22:330. doi: 10.1186/s12911-022-02056-5 (PMC9753367; doi:10.1186/s12911-022-02056-5)
Supplement: Supplementary file 5 — Additional file 5. Transcripts and the final decision aid prototype. [file 12911_2022_2056_MOESM5_ESM.zip › Additional file 5/ID13_transcript.docx]

**fStudy ID**: ID13

**Interviewer**: IC

**Date**: 8 March 2022

**Transcribed by**: KY

IC: So we are going through the decision… we are going to go through the decision aid together each page at a time, so this decision aid consists of 5 key sections. And as you are viewing each page and section, tell me out loud any thoughts that go through your mind. So, I may also prompt you with some questions along the way as well when you navigate through the pages. So for example, you can see the prompts here. So, each section, I’ll also be asking you about the amount of information – do you think it’s just nice or too little, too much, those kind of things. And then clarity of information – is it easily understandable, is it clear, those kind of things. As well as the presentation – how does it look like, the graphics, the navigation buttons, all those kind of things

ID: Ok. Didn’t bring my glasses today.

IC: So I will just ask you along the way, after each section. So once you are ready, you can read the disclaimer there and then when you are ok, you can press the button below.

ID: Ok.

(Clicking sounds)

ID: (slide 14) Do you actually start cancer survivorship from diagnosis? Because you survive actually after your partly cleared or something like that. When you [are] diagnosed, it’s like in your head it’s like death (laugh). So…

IC: So the definition of cancer survivorship, does it include from diagnosis?

ID: Diagnosis, ya.

(Clicking sounds)

IC: So for the first part about the navigation page where we showed you how to navigate the pages, what do you think about it? Do you think it was helpful?

ID: Yes, I think it’s quite clear because you’ve got the click buttons and you got the arrows and everything, it’s so… I think that’s quite clear for me.

IC: And then for the cancer survivorship parts, so that just now that part, what about the amount of information, clarity, and presentation?

ID: The presentation… I suppose it’s clear enough. It’s just that it’s a bit bland, but I suppose people won’t really look out for some of the… and attractive page because it’s supposed to be informational and not entertaining (laugh). Although [if it’s] entertaining, it would be quite fun.

IC: So, it would be considered as fair [or] poor?

ID: I’d say fair and…

IC: The looks wise?

ID: Yes, the presentation.

IC: Then do you think that we could, what kind of improvements can we make? Like for example, you mentioned that it was bland, so more color, more animations, or more graphics for example?

ID: Yes, I think animation would be nice although I can’t think of what’s sort of animation you put there. Because if you are not a text person, if you see pictures and moving things on the page, it’s easier for you to put your attention to the page rather than just [inaudible] pages, and bland pages like that.

IC: Understand. Then how about the amount of information? Do you think it was just nice or should we add more, or should we have less?

ID: I think it’s enough information because too much information would be a bit overwhelming, [be]cause some people I guess.

IC: And then the information that was presented, were there anythings that you didn’t know before or all of it you have known previously?

ID: I think most of it are I know already.

IC: And then the clarity of the information, is it clear, is there like some confusion, ambiguity?

ID: No, the language is simple and clear.

IC: Ok. Thank you.

ID: Carry on?

IC: Yes.

(Clicking sounds)

ID: (slide 18) Can I click on things that I have not undergone?

IC: I think you haven’t press the HER2 cancer therapy and the hormonal therapy.

ID: Ya, because I didn’t have this.

IC: If you didn’t have it, then you can press [overlapped voices].

ID: Ok.

IC: Can click the arrow below.

ID: Alright.

IC: Sorry, so for just now that part about the physical and emotional effects, then, what are your thoughts about it? So maybe the 3 things as well.

ID: Physical and emotional effects… Ya, all… everything that’s listed there, I think is relevant and ya. I was just thinking if there is… because like older people and people like me, sometimes it’s hard to read. Sometimes, I think it’s good if there’s narration, so that if you not, your eyesight is not too good, at least you can listen to it. So in the presentation, if there’s narration for you to click and it will read for you, I think that will be helpful, especially for older people. And, but then, you’ll have to do it in maybe different languages, which is quite a bit of work. Ya, but the information is all relevant as well as I have said. And…

IC: The amount of information?

ID: Yes, I think when you’re going through slides like this, the amount of information is good to be brief. So, it is brief. So I think that’s for me, that’s…

IC: Just nice?

ID: Satisfactory. Yes, it’s good. Ya, because if it is too much text also, in the end, it gets a bit wordy.

IC: Although it’s too much text, but then do you think it’s necessary or should we take something out?

ID: Yes, for this, I think it’s enough, the briefness is good enough, I think.

IC: Then, were there any confusion or everything was clear?

ID: It was clear.

IC: Then presentation wise, the looks, the graphics those kind of things?

ID: Yes, it was, it’s clear enough and it’s this size is big enough.

IC: Poor, fair, good excellent?

ID: Good.

IC: Ok. Then so far, you know from the start, do you think that there should be other information that we should include as well?

ID: So like maybe where to get the help, or is that coming up later in the presentation?

IC: There’ll be a part on other resources, but I guess it also depends on what kind of help you are referring to.

ID: Ya, for example when you say, you should get help from a counsellor or a family friend. Of course you can find your own family friends, but if you want to get help from a counsellor, maybe there should be another button to take you to another site that shows you where you can get counselling help.

IC: (slide 33) On the slide itself? On that page?

ID: Ya. Or I don’t know how…

IC: Later on?

ID: Later on, ya.

IC: And then, the physical effects part, were there any … information that you didn’t know before or most of, it all of it, you have known already?

ID: Yes, I think most of it I’ve known already.

IC: The previously, you were told or you have went to look for the information?

ID: I guess I went through it, so (laugh)…

IC: Because going through it, you will know that those things?

ID: Yes, but if I suppose I were a new cancer patient, it would be good for me to see those things.

IC: Ok can. Then, can continue.

ID: Ok.

(Reviewing)

ID: (Slide 34) This shared care, when you say cancer-related care are shared, it’s without you present? Does it… the information on you is shared with other colleges to help others? Is that what it means? I’m not quite sure what this shared care statement means.

IC: So the information is actually your own personal health information that usually is separated between the different health care professionals. But in shared care, this information might be shared across the institutions, for example, so you can see a bit more information later.

ID: It’s like… it’s on a database and other shared care providers have access to it?

IC: Yes.

ID: Oh, ok. Understand.

(Reviewing)

ID: (slide 41) Is this advanced practice nurse a new care option?

IC: I think it has been there for quite some time, but it would… so some of the survivor clinics is done by the advanced practice nurse.

ID: Ok. [Be]cause I’ve never encountered the… meeting with an advanced practice nurse.

IC: Do you see the surgical oncologist as well?

ID: My surgeon actually is a breast surgeon, but I don’t think she’s an oncologist because after the surgery, she sent me to the cancer center to see an oncologist and a radio [radiation] oncologist as well.

IC: So maybe that’s why you might have not encountered that advanced practice nurse.

ID: Ok.

(Reviewing)

ID: This family physician clinic also is a new thing, is it?

IC: It has… I think it’s been there for quite some time as well, but I think it’s not as well-known as for example, the general practitioners, the GPs, as well as the general polyclinic.

ID: Ok, I haven’t been to the clinic for a while.

IC: That’s quite a good thing too.

ID: Yes (laugh).

(Reviewing)

ID: (slide 43) Ok these… the pharmacists, this is not in relation to your… to cancer right? Is this just a general information?

IC: Yes, but in the shared care they might have different role, different kind of training. But this one is generally [the] current practice.

ID: Alright.

(Reviewing)

ID: (slide 44) This… the text boxes in different colors, are those relating to shared care?

IC: This one is usual care.

ID: Ok.

IC: (slide 44) So shared care, you have to click the other button there.

ID: Ok.

(Reviewing)

ID: So does that mean you don’t see the oncologist anymore if you have shared care?

IC: Yes. Or at least if for example, some people will see the oncologist twice a year like they go for 2 appointments. So I think yourself, it’s… currently its only one appointment per year is it?

ID: I think it’s 2, at the moment is 2.

IC: 2 per year?

ID: Yes, 6-monthly.

IC: Ya, so for situations like yourself, probably one of it will be seen at the polyclinic, so one session will be done at the polyclinic, one session come back here; or you might not even go back to the center and then just go to the polyclinic.

ID: Ok.

IC: So it depends on the situation.

(Reviewing)

ID: This is all within 1 year. (Phone ringing) Is that my phone? Sorry, Can I take that? I’ll put it on silent. Sorry about that. Alright.

(Reviewing)

ID: This is what I just went through right?

IC: Mm. You can press the… yup.

ID: That’s after I finish that already.

IC: Sorry, can you go to the previous slide? (click) Ya. I think you didn’t press… Just now I think you didn’t press the shared care one.

ID: I did. (click)

IC: (slide 44) Ya, maybe it wasn’t very obvious. We will take note of that.

ID: Alright.

(Reviewing)

IC: So this is the one that just now you were asking about right?

ID: Right, yes.

(Reviewing)

ID: So it’s basically the same right?

IC: It’s just another information might be consolidated and then shared across the health care professionals and health care providers. Then this is the same.

ID: This is the one?

IC: I think you have to press the usual care, one of the buttons to continue. Maybe in the meantime, I just wanted to check with you again on those same things.

ID: Ok.

IC: So, what do you think about the amount of information?

ID: It’s… I real-I feel like as I go along, I tend to lose concentration (laugh). But maybe it’s just me.

IC: Is it too much information or?

ID: Not really.

IC: Or might need a little bit more attractiveness?

ID: Yeah… if you put it that way. It’s sort of boring (laugh) but I…

IC: Bland like you mentioned.

ID: Ya. Although, maybe it’s because the information, apart from this shared care, is already so familiar for me. So…

IC: Can be a bit dry for some parts?

ID: Ya, yes. And I… the presentation… yea, I guess it’s alright. I think this has enough information if I were a new cancer patient, if I’m an old or I don’t know how to put it, if I’ve been a cancer patient for awhile, it sort of something that you may seen before, so… But because it’s simple enough and short enough, it should be ok.

IC: Then, so for separately, so the usual care information and the shared care information, both of them are just nice? The amount of information?

ID: Ya, but maybe the presentation can be a little bit more, separated, like maybe 2 columns or something like that, so that’s it’s clearer – this is for usual care, this is for shared care. Because when I look at that, even though it’s clickable, I might not… I might miss the clicking, which I did (laugh). Yes, so if it’s like 2 columns, you know this is for usual and this column is for shared care, so that I think that will be clearer.

IC: Ok.

ID: And because the diagram is the same, it looks like, it may not be, but it looks like it’s different information, but it was actually the same information right? You had the oncologist, the nurse, the primary care doctor, and the pharmacist. And then you had the other slide which showed the pharmacists going to… with their arrow pointing to both the usual and the shared care.

IC: Actually that one was… there’s separate diagrams actually for shared care and usual care. So for usual care, it’s actually that the pharmacists is not fixed but is only if the… you go and see the pharmacist and they need clarification, then they will talk to the other health care providers. But under shared care, there’s actually an assigned or at least a group of assigned pharmacists that will be involved in your care as well. And then, they will also have access to the shared common plan so they can communicate. For example, they can call or they can communicate through that plan itself. It’s actually a different thing. But maybe it’s not as clear from the diagram.

ID: Yes, [be]cause I miss that. [Be]cause to me like, the slides look the same, it’s just that because you have the same health care providers on both slides except that the pharmacist has different access points.

IC: Ok. So for usual care, would you say that it’s easy to understand?

ID: Ya, [be]cause I clicked here at first, so it was, the information was all there.

IC: But the shared care might be a bit confusing?

ID: The shared care, I didn’t quite see the difference because except you explained to me that [under] shared care, you would be seeing doctor could be a family doctor at polyclinic or one of those doctors instead of seeing just your oncologist right? So…

IC: Then, do you think it’s like hard to understand or understandable but with some ambiguity for that one?

ID: Ya, it’s... I mean the information is understandable, but the difference between the usual care and the shared care, the only difference I see is that the… you don’t see the same oncologist all the time. You might not see the oncologist at all and just see the other doctors or it’s split between your usual oncologist and the family doctor.

IC: Ok can. Then, presentation wise, you mentioned just now, then could you give a rating, the 1-2-3-4?

ID: Presentation wise…

IC: The looks, the navigation, the animation those things?

ID: Ok, I’d say fair.

IC: Fair for both the usual care and shared care?

ID: I guess the usual care, I’d say good; the shared care, fair.

IC: Ok. And then, would you like other information to be included as well?

ID: I was thinking of alternative because a lot of cancer patients always want to seek like TCM (Traditional Chinese Medicine) care as well, so how does that… or is that not advisable or you know whatever it is (laugh)? Whether it’s allowable but not advisable? Not recommended? Or whether you can have both? So, I would like to have that information because I know a lot of cancer patients do go to the TCM (Traditional Chinese Medicine) and how does that figure in all the doc[tor]s’ medicine. Because I meant in some hospitals, acupuncture is already accepted as mainstream, or maybe not mainstream but it’s additional to a normal care, normal medical care. And the, ya, that… I would like to know that as a follow-up care for cancer patients.

IC: Alright sure. Ok, and then, were there any information that from just now that usual care and the shared care, do you think anything was redundant or not helpful, not useful? Or generally everything seems to have their purpose in this?

ID: I think it’s good to know. But I suppose when you going through it, you don’t really think, you know whatever comes, you just do. So, it’s helpful I suppose.

IC: Ok sure. Alright, then I think… I’ll help you with [inaudible] Ok, then actually we do have the column part here.

ID: (slide 48) Yes, ok. Ya, I think this is a lot clearer because you can see straightaway.

IC: Much clearer?

ID: Ya. Although… it looks like there is a lot of information on the page, but I think if you… it’s also good because you can see in one, at one go what the differences are.

IC: Do you think that we should have put this at the start, or this should be at the start is it?

ID: Yes, I think if this were at the start, it would straightaway show the difference between the usual care and the shared care.

(Reviewing)

ID: So in shared care, you can actually have a group chat with your oncologist?

IC: Those are between the health care providers.

ID: Oh I see, ok, between health care professionals right. Ok, so that does not involve the patient.

IC: Ok.

ID: Then continue?

IC: Press the next one.

(Reviewing)

ID: This is the same information right?

IC: Mm. But in the column.

ID: Yes.

IC: So maybe this might be more useful at the front?

ID: Yes, I think this is… yes, this will be clearer.

(Reviewing)

ID: (slide 51) What is the walk-in store?

IC: For the pharmacist, for example to Guardian or Watson’s, those are where the community pharmacists are.

ID: Oh, I see. You mean at like when you walk-in you already have to pay is it?

IC: No, if you want to ask them about things, you normally don’t have to pay for it.

ID: Ya, what I mean is if you want to go to the hospital pharmacy, but you can’t do that anyway right? You can’t walk-in?

IC: I think currently, we can’t. Normally I think you will still need a prescription as well.

ID: Ya.

IC: But this one we’re actually referring to the community pharmacists, those are the oneo involved in our program.

ID: I see, ok, I understand.

IC: And then, sorry. So for this one, those 3 again.

ID: Ok.

IC: The amount of information?

ID: It’s… you mean the price and all that? The cost of the…

IC: Generally, the... all including the whole, all of the different tables.

ID: Oh. Ok, I think presentation is clear enough.

IC: It’ll be good, excellent, fair?

ID: Good.

IC: Then amount of information - is it similarly, it’s just nice or too much, too little?

ID: Just right I guess. Ya, because I don’t know what other information I might want to know. Ya, just nice (laugh).

IC: And then, is it easy to understand or a little bit confusing for example?

ID: Yes, it’s easy to understand but the… but on how will you be presenting this information. Because right now, it’s like I’m just sitting here looking at it. If I were looking for those information, then it will be good because I can get them straightaway. But if I weren’t looking for those information, I don’t think I would keep it in my brain, it’s just “oh, ok” and then I’ll forget about it. So, that’s just a comment.

IC: Can. So, what you might look out for, what you might want to know, it’ll also depends on your own preferences?

ID: Ya, because on like how we should be presenting this information, is it something you put on a poster? Or is it something you would put when somebody is looking for the information on the internet? Or is it general information that you give to patients when they come in as a cancer patient? Or is it then a booklet or a pamphlet to let them know?

IC: Ok. So actually, that is..., was another question we wanted to ask. Like what would you think would be good? So for example, do you prefer… I mean the idea of this is actually to be on the website… website kind of format? But like you mentioned, so, it will be good also if that information, maybe in another format, but this kind of information would be good to put for example on posters, on pamphlets as well?

ID: Ya, yes. Because like for example, if I’m sitting in waiting room, [be]cause sometimes you have to wait a long time If you have those posters, if you have nothing to do, you can look at them and you might learn a few things from that. Or, if I’m actually looking for the information, if it’s… there are pamphlets available, then, I can just get them. Because sometimes you really don’t know what to expect, so sometimes you don’t know what to ask either. And if the information is available right there, then you can just pick it up and learn something from it. Of course, if you’re looking for the information and you look on the internet, that will be good also because then you know exactly where to look and what you want to find out (laugh).

IC: Ok, can. Then, would you prefer this information to be presented on hardcopy version or on a softcopy version? For example, maybe like we give you a tablet then you can look through or give you a website and then you just click on your phone those kind.

ID: Oh, for me personally, I like… I’m old-fashioned (laugh), I like something I can hold on with, besides the phone is hard to read because my eyesight is not so good. And I think a lot of older people, I’m just assuming because (laugh)… I think it’s easier to read something on paper. And if it’s on a poster, even better [be]cause it’s big and it’s eye-catching also. On the internet, of course it’s good if you are looking for it. [Be]cause if you are not looking for it, you’ll just miss it.

IC: Ok can. Alright then, you want to continue to the next part. We have just 2 more parts left.

(Reviewing)

ID: Is this a clickable page?

IC: No.

ID: Ok.

IC: But it looks like it’s clickable is it?

ID: Because it’s the pages have been clickable so… sorry.

IC: It’s ok.

ID: So I was expecting something to be clickable.

IC: Ok.

ID: Because there’s the choice. So then it’s just continue?

IC: Mm.

ID: This one also… oh this is an actual questionnaire?

IC: Per se, yes.

ID: Ok.

IC: So this one is clickable (laugh).

ID: Ok.

(Reviewing)

ID: I don’t mind shared care.

IC: So for this one, those 3 again. The amount of information – do you think it was just right or too much, too little?

ID: I think it’s just right.

IC: Just right. Then, was it easy to understand? A little bit confusing?

ID: Ya, it’s easy to understand.

IC: Presentation wise?

ID: It’s clear.

IC: Then, rating will be?

ID: Good.

IC: And then, in your opinion, what is this section about?

ID: It’s just to find out if the conditions are right, whether I would consider shared care as opposed to the usual care.

IC: Ok can. And then, would you think that this exercise is helpful to allow you to better understand your preferences in follow-up care?

ID: Ya, because this is not… it’s something you’re considering right? Ya, so, I think it’s useful for you.

IC: And then, in what ways do you think that this exercise will be beneficial or helpful in your decision-making?

ID: For one thing I would know about shared care [be]cause I don’t know about it. For another thing, if it is rolled out, it is convenient and if the doctors that I’m seeing under shared care are well-trained, and they know what they’re doing, then, I don’t see any difference because if you need to be referred to someone who knows more and they will refer you, so I think it’s beneficial for aftercare for cancer patients. And it’s convenient and its cheaper.

IC: So those are some of the factors that will be important to you when deciding on what kind of care you’ll take?

ID: Ya.

IC: Ok. Are there any other factors that you think will be useful to include or things that are important to you that maybe we should include for like important to yourself or your peers, the other fellow cancer survivors as well.

ID: It’s the… maybe the… when you have to do tests, will these places where you see the shared care doctors, would they because at the hospital everything is there. So, it’s convenient in that sense. But if you go to polyclinic, they may not have facilities for testing, so, ya, I would like to know if that would be available once they start the shared care, the shared care program, whether all the things facilities will be there as well. It’s not essential but it’s good to know if they’re there.

IC: If not you might have to come back to the hospital just for the…

ID: Ya, for the test.

IC: Sure. And then, were there any other… were there any information that you think is not relevant or… Or what are some of the factors that are listed that might not be as helpful to you to think of not as important for yourself? I think the first 3, the first few are the ones you mentioned are quite important to you.

ID: Ya. I think that the point on whether the community care plan is appealing, I think that’s not a necessary point because if the other things are already ticked, that mean (laugh) it’s a feeling… so it’s like redundant to me.

IC: For that part? Ok can. Alright, then, you can go to the next part. Or do you have any other comments on this section?

ID: I can’t think of anything else.

IC: Then the next part. Sorry… it’s ending. So there’s still the additional resources you can click later.

ID: Ok.

(Reviewing)

ID: So I don’t need to click this… the additional resources right.

IC: You can just take a look. So, we just want to know whether or not this is comprehensive enough? Or what are the other information that you would like to know about? So maybe we can include the alternative, the traditional medicine, for example, that you mentioned just now, especially here.

(Reviewing)

ID: There are… groups right like cancer support groups? Would you have the information on here as well under resources. Is it under… this one? Ok.

IC: So this would be important to know?

ID: Ya.

IC: Almost done.

ID: I’m not hungry, my stomach always does that.

(Reviewing)

IC: Ok.

ID: Ya, it’s quite interesting to read about all these.

(Reviewing)

IC: So that’s mostly it. Then do you think this information is just nice, too little, too much?

ID: I think it’s just nice and because if you need more, you can just click on those extra information.

IC: Ok. Then do you think it was easy or confusing those kind of things?

ID: It’s just that that page where I got confused between the shared care and the (laugh).

IC: Oh, as in I was referring to the other resources part.

ID: Ya, the other resources. Yes, good, I like that, I would have liked to click on it, my stomach keeps making growling noises.

IC: And then presentation wise?

ID: Yes, it’s clear.

IC: Then, the rating?

ID: The rating… excellent.

IC: Then generally so far across the whole decision aid, the amount of information is just nice?

ID: Yes, I think it’s just nice. Ya, because I can’t think of anything else I’d like to.

IC: And then generally easy to understand except for the shared care part that you mentioned?

ID: Yes, it’s easy to understand.

IC: Then how do you find about the navigation overall? Is it easy use or a bit confusing sometimes?

ID: It’s quite clear and also in the beginning, also you explained the navigation, so I think it’s good enough.

IC: Ok, and then… were there any information overall that you think will not be useful or they’re redundant, not helpful, those kind of thing? Or everything is?

ID: Well, I think everything is relevant to you if you are a cancer patient and I think you would want to know all these information and you will go onto read about them.

IC: Ok. And then, some follow-up questions. Maybe I can open it up here, let you see. So do you mind answer-helping me answer these questions? Then maybe I’ll read out to you and then you just let me know your answers.

ID: Ok.

IC: So the first one is would you prefer a digital decision aid over a paper booklet? Think you mentioned just now you prefer the paper one.

ID: Yes, although I don’t mind if it’s digital one as well.

IC: So will you be neutral or you will prefer paper booklet?

ID: I think I’ll still prefer paper.

IC: Because you… it’s more… you’re generally a bit more traditional, you prefer holding something right? Just now you mentioned.

ID: I’m old fashioned (laugh).

IC: And then was it easy to navigate across the pages and links?

ID: Yes, it was.

IC: Was the decision aid interactive?

ID: Yes.

IC: And then, so the content, we went through just now, it’s the… these 3 things here.

ID: Do you want to scroll down? Ok.

IC: And then, so the next question is do you find the preference clarification exercise helpful? So that was the one you checked, click to check on which side you lean towards.

ID: Yes, it was helpful. But it was helpful for you right rather than me because it will not make a difference to me since the shared care is not yet rolled out.

IC: So that one is … if the shared care is eventually rolled out, then because the eventual choice is still up to the patient, whether or not they want to stay in NCC in usual care, or take up the shared care where they might only come back once in a while, then the rest will be done in the community. So, we are just wondering if it will be helpful because sometimes, we are just wondering if patients like… what are the considerations they might want to make when…

ID: To decide between the 2.

IC: Ya.

ID: Yes, it’s useful.

IC: Then do you find the presentation of information to be slanted towards the usual care option, to be slanted towards the shared care option, or to be quite balanced?

ID: I think it’s balanced. The information, you can decide what’s… one way or the other.

IC: Then…

ID: Slightly slanted towards… because you’ve got options that people would prefer for the shared care like convenience and all that.

IC: So, the presentation wise, it’s balanced. But because of the considerations that people might have, it will seem a bit slanted? Was it?

ID: Ya, maybe I’ll take that back because it’s quite balanced if you consider one, for example, the first one it says it’s… you might not have confidence in the shared care doctors, so I think it’s balanced on second thought (laugh).

IC: But it also depends on the preferences of the participant.

ID: Yes.

IC: Ok sure. Then, do you think the length of the decision aid, was it too long, just right or short?

ID: I think it’s just right because for the amount of information.

IC: And then so the next part is about the utility in decision-making. So do you find this decision aid to be useful in making a decision about follow-up care after cancer treatment? Yes or no.

ID: Yes, it’s helpful because you know what to expect from the shared care, yes.

IC: And then, would you use this decision aid to discuss follow-up care options with your oncologist or other health care professionals caring for you?

ID: But, I don’t quite see what I will be discussing with the oncologist or the health care professionals. Can you elaborate on that question?

IC: So maybe like, just now I mentioned, because the kind of care that you will take up is based on your personal choice, so someone might introduce you to the shared care model for example. But whether or not you do go to shared care or do you stay in NCC - usual care - it’s your choice. So, just wondering like, do you think that this decision aid, so this these slides or even for example the pamphlet if we do have a pamphlet later on. Will you use this thing to talk to your oncologist, to your doctors, about the follow-up care for example?

ID: You mean…

IC: To… whether or not to stay at NCC or to go to shared care

ID: Whether I would use this information in this aid to decide?

IC: Whether or not you use this decision [aid[, ya, the information in this aid to decide.

ID: Yes, of course, I would use the information and…

IC: The information provided here then you will talk to the oncologist with?

ID: To ask if I could opt for this.

IC: To… if you have any clarifications or questions for example.

ID: [Be]cause at the moment, I don’t see what I could discuss with them on this since it’s not an actual option.

IC: But if it does…

ID: But if it were… Ya, I might. If it is and I were given the option, I might discuss it.

IC: Ok sure. And then would you recommend the use of this decision aid to other cancer survivors if like this thing actually, eventually rolls out.

ID: Yes, I think it’s good for people to have choices.

IC: Or the information provided as well?

ID: Ya.

IC: Ok. And then when do you think would be the most suitable time to introduce this decision aid to cancer patients? So upon their diagnosis, during active treatment, immediately after active treatment, or years after active treatment, or other time periods?

ID: I think a good time would be just after active treatment because I think the… at the start, it’s hard to think about other things other than just getting treatment.

IC: And then, last 2 questions is what do you like about the decision aid?

ID: I like the fact that you have a choice (laugh) So…

IC: That is very important to you as well?

ID: Ya, and it is an extra option and that is always good to have more options.

IC: Then, do you have other suggestions pertaining to the decision aid? That you might not have mentioned previously like just now?

ID: I don’t think so, I cant think of anything right.

IC: Ok, can alright. Then I think that that’s mostly it for the decision aid. Thank you so much. Then, just need your help with some demographic information.
