# Supplementary material for: Development and pilot testing of a decision aid for navigating breast cancer survivorship care
Source: BMC Med Inform Decis Mak. 2022 Dec 15;22:330. doi: 10.1186/s12911-022-02056-5 (PMC9753367; doi:10.1186/s12911-022-02056-5)
Supplement: Supplementary file 5 — Additional file 5. Transcripts and the final decision aid prototype. [file 12911_2022_2056_MOESM5_ESM.zip › Additional file 5/HCP06_Transcript.docx]

HCP06

**Study ID:** HCP06

**Interviewer:** GT

**Date:** 15 December 2021

**Transcribed by:** IC

GT: Okay, so we’re going to go through this decision aid together, each page at a time. This decision aid consists of five key sections. As you’re viewing each page and section, tell me out loud any thoughts that go through your mind. I may also prompt you with some questions along the way as you navigate across the pages. Some of these questions that I might be asking may be about the content, like how accurate the information provided is, and how about the language and appearance, as well as how feasible you think will be for routine use in the clinical setting. So if you are ready, you can start, can start by reading the disclaimer and click the “I have read and understood the above”.

ID: Click. Can see? Can see the shared screen?

GT: Yes, I can see.

ID: Okay.

ID: So what am I supposed to do now?

GT: You can just, you can just read it and then any thoughts you have you can just share with me.

GT: Okay. So for this section, how do you find about the information provided?

ID: It’s okay. What do you mean by how did I find about the information?

GT: How accurate, or is it sufficient?

ID: I just started reading. This is barely the

GT: This is actually the first section. After that we move on to the next section.

ID: Oh.

GT: Yeah.

ID: Okay.

GT: So you can just comment

ID: I think this is quite preliminary, I guess the overview, I suppose.

GT: Yeah, correct.

ID: Mm.

ID: Just to highlight that “frozen shoulder” is not really a consequence of surgery. It’s more a consequence of radiation therapy.

GT: I see.

ID: So another suggestion is if I go back, it would be useful to go back directly to this page instead of always going back to this page.

GT: It will be that page. Because that page you are reading on this different bubbles of the treatment.

ID: What do you mean by different bubbles?

GT: So you click on the next arrow right, there are different bubbles for different treatment types, so there will be different treatment modes and the different physical effects associated.

ID: So [GT], after I read “chemo”, and I want to go back, it should go back

GT: Oh. It goes back to the

ID: to this page instead of this page, then I need to press this to go to this, you know what I mean?

GT: Okay, one way

ID: This is an extra

GT: One way you can do is

ID: page

GT: you can click, you can click out of the, you can just click the, the slide itself. So for example you click, you try clicking on “chemotherapy”, click on the “chemotherapy, so you just click here, you just click there. Yeah, just click. Yeah, then it will just go back like this.

ID: Yeah, but this is more intuitive, right?

GT: Yeah.

ID: Because you actually have a arrow that brings you back.

GT: Because I think the arrow is referring to the

ID: Okay, that was just a suggestion.

GT: Yeah, okay, sure, thank you.

ID: I, up to you to click right out of it

GT: Sure, sure. Yes thank you. We will take it into consideration.

ID: I’m not here to argue with you.

GT: Yes, yes, okay.

ID: I see there is a back arrow

GT: Mm

ID: That is coming back to the

GT: So it is confusing, right?

ID: It’s not confusing. It’s just unnecessary.

GT: I see.

ID: Right? So, it’s just feedback.

GT: Okay.

ID: So nobody will know to click this, right?

GT: Because actually there is also a cross, there is also a cross button there, so.

ID: What cross button?

GT: At the, at the top, top right end at the corner there is a cross button there.

ID: Sure, okay. Then why don’t you remove the arrow at the bottom then?

GT: I see. Like remove it away, the arrow.

ID: Okay, just curious about that. Okay

GT: Okay.

ID: So what is the next section? This is the next section?

GT: Yes, this is the next section. Mm.

ID: “What are the options”.

ID: Okay. The feedback is that it is very wordy.

GT: Mm. Okay.

ID: Okay. If I’m the physician, I mean, if I’m the patient, I’m really lost right now.

GT: Okay. So any suggestions on how we can make it less wordy? Like putting it in

ID: Reduce, reduce the number of words. And maybe put it all into one page? What does shared care look like, and what does usual care look like, so that at one glance, there is a comparison.

GT: Okay, can.

ID: It’s spreaded over so many page

GT: Yeah.

ID: It’s very hard to read, for example what it used to be and what, what difference.

GT: I see.

ID: And what’s the difference?

GT: After these slides we’ll have some comparison, we’ll have a comparison table after this, after this shared care.

ID: So this part, what could, what are the possibilities, is it referring the survivors back to their oncologist, if there’s something out of the ordinary. Then it says “don’t worry”. But what are the examples of

GT: Okay.

ID: Yeah. Because it is immediately alarming to the patient if they are sent back to the oncologist.

GT: Mm.

ID: So if the intention is to reassure them,

GT: Mm.

ID: You know, then providing some examples of a benign reason could be helpful.

GT: Okay.

ID: Oh, so we’re back to this page, is it?

GT: Yeah, so here you can click on the next section.

ID: I think this is a misrepresentation because there is definitely communication between healthcare professionals.

GT: Mm.

ID: Right? To put it as “X” is not accurate and also doesn’t give credit to the current type of care that we are providing.

GT: Okay.

ID: Unless, you are trying to say that the oncologist don’t talk to the family physician and pharmacist currently.

GT: Yeah, I think that is what we’re referring to.

ID: Yeah, mm. I’m not sure how many patients actually have a family physician.

GT: Mm.

ID: Do y’all know? In Singapore? Don’t know?

GT: Yeah.

ID: Okay.

GT: So back to the previous section right, there is actually a question mark there that shows the cost breakdown. The question mark can, the question mark button can be pressed. On the second, second slide. Mm. This one.

ID: Why do we need this?

GT: Because I think it’s

ID: Why do we need the care navigator?

GT: It’s the pharmacist, the pharmacist on board, to kind of like, to give advice to the patients and their medications as well as any lifestyle advise.

ID: Okay. How often do they call the patient?

GT: For now, our project, they call them every three months. For the BASIC trial.

ID: Okay. What is the common question for the uptake of the utility of the, you know, say I’m doing my work, going in to my normal day life and suddenly a pharmacist call me and try tell me about my cancer care.

GT: I think, yeah, it’s

ID: How many people have been receptive to it?

GT: Some people find that they would rather see the person face-to-face instead of like, through a phone call, because they don’t feel comfortable talking, just talking to a stranger through the phone and divulging their, their health information

ID: Yeah.

GT: Yeah. So for that participant, we actually did like a, we did like a Zoom, a Whatsapp video call for her.

ID: No, but how many people also find it, do you make a private appointment to call them or? Or when y’all are free, y’all take turns to go down the list to call? I keep on imagining if I, if I’m the patient and I’m going on my life normally and suddenly I get a call out of the blue and they say, you know, how’s your cancer treatment, you need to change your diet or anything? I’ll be like, now not so convenient.

GT: Will a video call be more helpful instead of a phone call?

ID: I wouldn’t want my day interrupted by anybody.

GT: I see.

ID: Video or otherwise. And especially if I’m not thinking of my cancer,

GT: Mm.

ID: And, and suddenly, you know, if I’m having a meeting, or you know, I’m working on a project on, on a business question, and suddenly interrupt my day by telling me about my cancer

GT: Mm.

ID: I would find it extremely discomforting.

GT: Mm. So you find this care navigation portion to be a bit unnecessary?

ID: I think it’s an interesting and new and novel thing, but it may need to be better curated. Number one you need to see if it value adds, and number two, you know, you shouldn’t be doing things if it does not value-add, if it causes distress or disturbance, right?

GT: Mm. Yeah.

ID: And it also costs money right?

GT: Yeah.

ID: And then, and then you also need to find out what is the best interval

GT: Yes.

ID: A lot of considerations.

GT: True, true.

GT: Yeah, so for the consultation cost, there is a question mark there.

ID: So, what am I supposed to do with this information.

GT: Just see only.

ID: So you’re not including the cost of the navigator at all?

GT: Yeah, from here we, we actually absorb the cost, cause actually NUS is actually paying for it.

ID: I know, but this is again a misrepresentation right? You choose this but it’s in the trial, then if this is what I want to roll out long term

GT: It is not as feasible. Yeah, okay.

ID: Because if you are actually trying to see whether this is private or something larger, then the accurate costs should be reflected right.

GT: Okay.

ID: And if I’m the patient, I would want to find out every three months somebody call me, how much is that going to my bills.

GT: Yeah.

ID: Right? And is my tax dollar going to increase? Especially if it’s not going to be, well if I’m the patient, I would, when I have questions

GT: Mm.

ID: I want somebody to be available to answer it, but if I have no questions, I don’t want to be disturbed.

GT: Okay.

ID: But that’s my personality. I admit like, not everybody will be like that.

GT: Yeah.

ID: Yeah. Some people like, if they are retirees and all that, they may not mind somebody calling out of the blue to chit chat.

GT: Yeah, I mean, if it is a working, if they are working, then they may not want the disturbance.

ID: Mm. So different people have different profiles will be different.

GT: Yes.

ID: I think that if you want to highlight the shared care model, you need to highlight the benefits of it.

GT: Okay.

ID: I mean, the family care physician is going to take care of all your other medical problems, and that I think is the greatest plus.

GT: Mm.

ID: That means

GT: Yeah.

ID: the family care physician knows about your hypertension, your cholesterol, your thyroid problems

GT: Mm.

ID: and knows how to manage the side effects of endocrine therapy or chemotherapy, along with everything else.

GT: Mm.

ID: So, that to me is the biggest benefit and not the cost-savings or a person calling you intermittently.

GT: Okay.

ID: And that may be conveyed, or should be conveyed to the patient.

GT: Mm.

ID: Right?

GT: Yeah.

ID: Usual care means that your, to me, usual care means that your oncologist or your cancer care is distinct from the rest of your medical conditions. Whereas shared care means it’s considered in entirety and everything is coordinated by one family care physician.

GT: Okay.

ID: I, yeah. And that has way more benefits.

GT: Okay.

GT: [ID], there’s this

ID: Oh, then how, how do I get back to here?

GT: Yeah, go back to, cause you haven’t click the rest of, yeah.

ID: You want me to click the button is is? Or?

GT: No, there’s still one, there’s still more, there’s still, yeah.

ID: Yeah, so my biggest comment is that the shared care model is under, the benefits of it, is undersold.

GT: Okay.

ID: Right? That means, really, you are putting the family physician at the centre, at the heart of it. And that relationship between the patient and the family physician who understands and know and can absorb the cancer aspect of the patient’s life into her whole healthcare.

GT: Mm.

ID: It’s not being conveyed in this decision aid.

GT: Mm. So over here, do you feel like these are the factors that breast cancer survivors would consider when making a decision about follow-up care?

ID: Mm, I think cost is important, convenience is important, but, I would, I would be willing to pay twelve dollars more, right

GT: Mm hmm.

ID: and travel down to see someone else.

GT: Mm.

ID: If, you also have to consider that people like routine.

GT: Mm.

ID: They’ve been doing something, seeing their oncologist for three years, right, they feel that they are under good care of their oncologist, if there’s not a very big plus to be moved to shared care. The cost is of little concern.

ID: That’s not a meaningful enough reason to change, to why I should

GT: I see.

ID: discontinue communication with oncologist.

GT: Okay. Then do you feel that there may be other factors that are not listed here that they’ll consider when they want to make a decision?

ID: Mm hmm. That means, the shared care model, what I have been saying twice already.

GT: Oh, sure. Okay.

ID: That would be the reason that I would choose shared care if I’m the patient, that means if I have one doctor who can take care of all my problems

GT: Mm.

ID: Or I can see one doctor

GT: Mm.

ID: having all access, if there’s any need, then you go back to my oncologist. If there is no need, then I save time. That, that is a great plus, but it is not adequately conveyed in this decision aid.

GT: Okay. Yeah, so this is the end of the decision aid.

ID: Oh great! I think this is a very nice thing, and is very pleasantly put together. I think all your graphics, and then the clicking and all that, is actually very easy.

GT: Mm. So here there are actually other resources and there’s, you can click onto each tab actually. So there will be like different topics, but I think now you can’t click because it is actually linked to a website, so don’t need to on the thing.

ID: Oh, okay.

GT: Yeah, can just see the, the range of the topics that are available here.

ID: Very nice.

GT: Thank you.

ID: Very nice. I, I think that it is actually, like the patients will feel engaged, like a bit empowered when they, you know, take this, and if I were the patient, I will be like, oh, I am trying, I am being considered in shaping what I would want on the kind of care services, so I would feel good.

ID: Okay. So the two big things. One is the layout of the comparisons instead of so many slides for the follow-up care options. Maybe one slide show the two things that you compare upfront.

GT: Mm.

ID: Right? And two is highlighting the benefits of having the family care physician at the center of all the patient’s needs.

GT: Okay. Then I would like to ask also, because how difficult or easy do you think it will be for you to introduce this decision aid to your patients? For example, maybe like, you can provide this online link for them to access.

ID: You’re talking about my survivor patients is it? Or?

GT: Yeah

ID: Who are you targeting?

GT: The breast cancer survivors , so those, so for our this target will be for those three years out of their primary treatment, at least three years.

ID: I think it shouldn’t be difficult because you have a specialised subsidised, not subsidised, but a specialised survivor clinic, and all those patients are five years out, right?

GT: Mm.

ID: And that is a, typically a slightly lighter clinic.

GT: Mm.

ID: whereby they see about ten patients a session

GT: Mm.

ID: So, the resident physician who is running that clinic can introduce this, it shouldn’t be too difficult.

GT: Okay.

ID: But they may not have the time to let the patient do it during the consultation.

GT: I see.

ID: But the patient can, then, maybe in the waiting room do it, right? So you can provide the QR code, they can scan it and they can go to the waiting room and do it.

GT: Okay.

ID: Or, it can be sent to the patient as an SMS, maybe?

GT: Yeah.

ID: But, I don’t foresee a patient doing this with the doctor alongside in the clinic consultation.

GT: Too, too time-consuming right?

ID: Absolutely.

GT: Yeah. So it’s more like patients read on their own first

ID: Yeah.

GT: If they got questions

ID: If they can do it, they can do it in the waiting room. So that means when the patient register and say that “Hey, I’m a survivor patient, I’m coming for the survivor clinic”, immediately you know that they are already five years out, and they belong to this population. So while they are registering, they then ask us to scan the QR code, then they sit down and help them pass time, you know, before they come in.

GT: Yeah.

ID: Then yeah, yeah. So I think those are the possibilities. I think inside the consultation a bit difficult.

GT: Okay.

ID: Were y’all thinking of that when you ask questions or what?

GT: Yeah, so my next question was to ask whether you feel comfortable discussing the information presented in the decision aid with your patients.

ID: What do you mean by “feel comfortable”? Of course I feel comfortable, but I feel like I don’t have the time. Right? Our clinics are really a bit tight, and we will not be going through, physical effects, why don’t you click radiation

GT: More like

ID: You know these?

GT: Yeah.

ID: No, we will not be doing that.

GT: Okay, can. Then, any other thoughts that come into your mind when you were viewing this decision aid that you haven’t shared?

ID: No, I think I have shared along the way my thoughts as they have come up.

GT: Yep, okay can. Then that’s all for the decision aid.

ID: Perfect, thanks very much.

GT: Thank you.
