# Supplementary material for: Development and pilot testing of a decision aid for navigating breast cancer survivorship care
Source: BMC Med Inform Decis Mak. 2022 Dec 15;22:330. doi: 10.1186/s12911-022-02056-5 (PMC9753367; doi:10.1186/s12911-022-02056-5)
Supplement: Supplementary file 5 — Additional file 5. Transcripts and the final decision aid prototype. [file 12911_2022_2056_MOESM5_ESM.zip › Additional file 5/ID14_transcript.docx]

**Study ID**: ID14

**Interviewer**: GT

**Date**: 9 March 2022

**Transcribed by**: KY

ID: So can I start now?

GT: Yes.

ID: Ok.

(Reviewing)

GT: Ok, you can click on the first bubble first.

ID: This one?

GT: Yes.

ID: I click, nothing. First bubble?

GT: Can click, you try again. You can’t click, your side can’t click is it?

ID: Can’t.

GT: Try hovering it over the first… I can see… hover it again the first one.

ID: I can only click is the home you know. All others is… ok this one.

GT: Should be able to click this one.

ID: Ok. It’s fine.

GT: Yes, correct.

ID: Where should I click now? Ok, next.

GT: Yeah.

(Reviewing)

GT: Ok. For this section right, how did you find about the amount of information?

ID: Useful.

GT: You feel that it’ just enough, or you feel that it can be more?

ID: Basically, it’s enough, because it explain what is survivorship, follow-up sessions, cancer programs… [voice fades].

GT: How about, do you feel that it is easy to understand or do you feel it’s [overlapping voces]?

ID: Ya, easy to understand.

GT: And how’s the presentation?

ID: It’s clear [for] this part.

GT: How would you rate it?

ID: May I continue?

GT: Wait first. How would you rate it from poor, fair, good, or excellent?

ID: Good.

GT: Ok, then you can continue to the next section.

ID: Next section.

(Reviewing)

ID: Can I continue?

GT: Yes.

(Reviewing)

GT: Okay, so for this section - the physical and emotional effects - right, how do you find about the amount of information?

ID: I think it’s clear. It's clear.

GT: It’s clear also?

ID: (slide 33) Ya. If let’s say, maybe, ok, like the second paragraph, the last line the last word ‘counselor’. Counselor, is it good maybe to show a website, a web link, which organization can be the counselor or this information is at the back? I'm not so sure, because you still haven't go through all the slides. Because friend, family member for sure we know who are they. But counselor, from were?

GT: Ok, so like to direct you to the resource itself?

ID: Yeah, at least I know “ok, which association, or what is the contact number or the website”.

GT: Ok. And how about the presentation?

ID: Okay, it's clear, simple, and clear to understand.

GT: Would you rate it a… is it a… how would you rate it? Poor, fair, or excellent?

ID: Ya, good, it’s good. So I can go to the next section?

GT: Yes.

(Reviewing)

GT: Can go back to the usual care- shared care?

ID: The previous one? So slide… right now, I can’t till I finish this one is it?

GT: Maybe… yup.

ID: No, I didn't see the go back.

GT: It's okay, they got ask you to go to shared care after that.

ID: I think after I finished clicking…

GT: Ya, after you finish with this slide.

ID: Ya.

(Reviewing)

ID: Ok, then where can [I] click, I didn't see?

GT: You can go to shared care.

ID: Shared care… a bit slow in response. I think maybe because it's a request control.

(Reviewing)

ID: Okay, I go back right.

GT: Don’t need.

ID: Just carry on [to] next session?

GT: So I would like to ask you for this section - the follow up care options - how do you find about the amount of information?

ID: Useful, ya, easy to understand.

GT: Also, enough information? Or do think it’s too much?

ID: Because it’s very generic so I think should be enough already, information [is] sufficient.

GT: Sufficient. How about the presentation?

ID: Also good.

GT: Okay, can. Then can move on to the next section.

ID: Section. Ok. Comparing the options.

(Reviewing)

GT: Okay?

ID: Ya.

GT: For this section right - comparing the options right - how do you find out about the amount of information?

ID: Also, it’s easy to understand.

GT: And enough?

ID: [Be]cause everything is generic, so it’s based on the nature of this deck of slides is more generic, telling the patient, what are those information, then… Yeah, I think it's good enough already.

GT: Okay, how about the presentation?

ID: Also good, back to go to the next section?

GT: Ya.

(Reviewing)

ID: How to click?

GT: Just press the next arrow, the arrow at the bottom [overlapped voices].

ID: Because if put right here, there are two possible decisions.

GT: Yeah, I was just explaining to you.

ID: So, I don't need to choose right now?

GT: After that, there is a[n] exercise with these issues. Yes. Yeah. So over here, you can actually put a ticks in the ones that you feel are… apply to you.

ID: Ok.

(Reviewing)

GT: So I think you choose a lot more on the usual care side.

ID: Ya.

GT: You're leaning, you feel that you lean more towards usual care?

ID: Yes.

GT: Any other factors here that you feel that are not covered in this exercise that you feel is relevant?

ID: I think the reason is my situation is stabilized already. Although there is a shared care plan, I feel like, since I had the breast cancer [and] went for surgery till now is already, let me count how many years, 5 years plus going to be 6 years. So, I don't have any side effects, no matter is after surgery, after the radiotherapy, after the hormonal therapy, I mean the medication. So, for sure, I don't need to see the oncologist so often, to see the doctors so often so another thing about is like… Then I[’m] also being covered by the company policies, the medical policy, so the cost is not a problem to me. So for this kind of factors, I think [it] also depends on individual, their own background.

GT: So any other factor you feel that, from you personally, that you feel is not covered here?

ID: I think it’s quite sufficient enough to find it out why I would like to choose the usual care or the shared care. I don’t think about cheaper, convenienting, maybe… ok. If… now cannot [because] submitted already.

GT: Yeah.

ID: I think I'm more on the usual care side. So, I don't need to read say like, explore the shared care. This is what I think about. So I can go the next section?

GT: May I know maybe what is the… your hindering, hinderance towards shared care?

ID: No, nothing.

GT: Because you’re just more comfortable with usual care?

ID: Yeah, so that’s why I didn't look through the other side, the shared care.

GT: Then maybe I also would like to find out for this section, how do you feel is the amount of information?

ID: Yeah, the information [is] good enough already.

GT: Easy to understand also?

ID: Yeah.

GT: And how about the presentation?

ID: Also good as well.

GT: Okay.

ID: Then I can go to next section?

GT: Yes.

ID: It’s not working. It is not working.

GT: It’s not working?

ID: No, I can’t see my little palm (hover cursor).

GT: After this, this is not working. Ok.

ID: I can’t see my little palm.

GT: I also, my end also, I can’t control. I think I have to escape and come out again.

ID: I don't need to redo right?

GT: No, we'll just start from here again, just tick again.

ID: Yeah, so I submit my result. Maybe this one?

GT: Ok. You try whether… still cannot?

ID: Ya.

GT: Something is wrong.

ID: Don’t know why (laugh).

GT: So basically, this will be the conclusion slide already.

(Reviewing)

ID: Ok, I'm fine already.

GT: Ok, you can click on the additional resources.

ID: Also cannot. Now ok.

GT: So over here, just let me know about the range of topics over here you feel… How’s the range of topics over here? Is there anything that perhaps you would like to see that is not covered? [overlapped voices] You can click on each tab actually, each tab can be clicked on.

ID: This is to say, this page of information is to say like what resources I need to have right?

GT: Ya, so you can actually click on them to see what… [overlapped voices] But don’t click on the links because it will direct you to a website link. Ya, you can just see the topics, whether or not that you feel is something that you would like to see.

ID: Yeah, ok. I'm fine. Effects of treatment, ok. So, each one I've got to view is it?

GT: Ya.

ID: Okay. Aiya, click by mistake.

GT: Ya, that’s the problem.

ID: Eating, ok.

GT: Go back to… and then…

ID: Cannot click again (repeated).

GT: Ok.

ID: No, cannot click again. Ok, now can. Ok, support groups. Ok fine.

GT: The self-help also? Self-help.

ID: Self-help just now I saw.

GT: You saw already? Ok.

ID: Or I’ve already clicked.

GT: Can. So for these online resources right, how do you feel the amount of information is?

ID: As per what I said, generic, they are helpful and provide…

GT: And easy to understand?

ID: Yeah.

GT: How about the presentation?

ID: Also good as well.

GT: Okay, so I'll just like to ask you some questions to… to ask you some more questions. Do you feel that there might be other concerns right that other patients might have that the decision aid did not cover?

ID: Sorry, I couldn't get your meaning.

GT: So do you feel that there are any other concerns that other patients that might have that the decision aid did not cover?

ID: No, I think all covered already.

GT: All covered already?

ID: Ya.

GT: Any other information, apart from just now you mentioned about the counsellor; any other information that you would like for you to make a[n] informed decision?

ID: No, I couldn't catch any name.

GT: Any other information aside, just now you mentioned about the counselor right?

ID: Ya.

GT: So that… any other information that you will want to help you to be able to make a[n] informed decision?

ID: No, I think it’s fine enough. If let’s say, it’s a counselor, counselor…

GT: It could be other information that you want to see.

ID: No, I think enough already.

GT: Enough already?

ID: Ya.

GT: Then, is there anything that you read here that other patients or survivors might find confusing?

ID: No, I think it's good enough already.

GT: Then, how do you feel like this… that exercise just now you went through, the ticking right.

ID: Yeah.

GT: Do you find that it helps you to understand your preferences in follow-up care?

ID: Ya, its good enough.

GT: Ok, then how do you find the decision aesthetically? So [you] can comment on the color scheme, the choice of font.

ID: I think this is initial stage, and then, for sure more subsidies is good, especially like our age group. After three years’ time, I'm not working. If let's say I go to see,.. no matter is go back to the oncologist, for sure no more is covered at my company insurance. And if paid from my own, is the problem. So it’s like, ok, maybe one information good to, I mean to include right here, the plan, can I switch my plan?

GT: Switch your plan? What do you mean?

ID: So to say at the very beginning I think about, ok, I still wanted to go for the usual care plan. Then, after some years like me, if I'm going to retire, then I don’t have any further income, I will go to look into the share care, which is at a lower cost, more convenient. So maybe information to advise the plan that we can shift or not. If not shift, we can switch to another plan.

GT: Ya, so maybe these also can be discussed with your cancer doctor so that you can really make a… make your decision whether or not to move to… stick to usual care or to switch to shared care. So the oncologist also can also advise you and also advise whether you are suitable or not to even be going to shared care in the first place.

ID: Mm.

GT: Ya. Ok, then how do you find the use of all these icons and graphics and interactive buttons?

ID: Yeah, fine enough.

GT: Easy to be… easy to use and do you find their use was a bit difficult?

ID: No, I think it’s appropriate.

GT: Alright ok. Then, overall, how do you find this navigation experience? How… do you like it?

ID: Yeah, and it won’t be too lorsor (lengthy) (laugh).

GT: Any other further comments or suggestions you want to make about the navigation?

ID: No, I think is is fine. Or maybe… I think it’s fine. Maybe you can add on top of step… No, I shouldn’t say step, step 1… Like this one is ‘other online resources’, maybe is like point 5 or what, as in like maybe the first section is section 1, section2, so it will be easier.

GT: Number at the top is it?

ID: Ya, number it, might be…

GT: Ok, we’ll take it, take your feedback into consideration. Ok, then Just a few more questions. What do you think about the length and time taken to go to through the decision aid?

ID: Suitable, what do you mean again?

GT: Like, do you find it very long or do you find it ok or…?

ID: You mean the information very long or decision…

GT: The time taken to go through this decision aid.

ID: I think this is enough time, just fine, the time just fine.

GT: Yeah, okay. If let's say there is a downloadable copy of this decision aid right, do think it's useful? That means you can see on your phone.

ID: Ya, will be useful.

GT: Then will you revisit this decision aid for some of this information presented here?

ID: Ya.

GT: Which parts were you going to see again?

ID: Return of cancer, other online resources, because you never know. Although I'm taking the medicine, then there might be lapse of the cancer will come ack again or what. So I think this part, I would like to know more about it.

GT: Ok. Then, if given a chance right, will you use this decision aid to discuss follow-up care with your cancer doctor?

ID: Yes, I will. Yeah.

GT: Any other thoughts that come to your mind that [when] you’re reviewing the decision aid that you haven’t share?

ID: Yes, I will.

GT: Any other thoughts that you have?

ID: I think at this time, let’s I really encounter some symptoms, it’s going to return of my cancer.

GT: Can, then, ok, then any other questions you have for me?

ID: I’m fine already. It’s still helpful, I would like to say. Because I know government's trying to do something else [that] is not heavily depend[ent] on the breast cancer. And then actually, maybe like me, I have so called fully recovered if I'm not wrong, then it’s no point to go to the oncologists and then waste the chance for another patient to look for the doctor, to look for the oncologist. I understand.
