# Supplementary material for: Development and pilot testing of a decision aid for navigating breast cancer survivorship care
Source: BMC Med Inform Decis Mak. 2022 Dec 15;22:330. doi: 10.1186/s12911-022-02056-5 (PMC9753367; doi:10.1186/s12911-022-02056-5)
Supplement: Supplementary file 5 — Additional file 5. Transcripts and the final decision aid prototype. [file 12911_2022_2056_MOESM5_ESM.zip › Additional file 5/ID11_transcript .docx]

**Study ID**: ID11

**Interviewer(s)**: IC

**Date**: 4 March 2022

**Transcribed by**: GT

ID: I don’t know, I find that this is too wordy.

IC: Too wordy is it?

ID: It is too wordy (repeated) and I felt that maybe with some graphic that would be good with graphic because what you want us to be involved is the cancer survivor follow-up care is that what you want? (IC: Mm) So I think that is good enough. And then to me I think this is too wordy. Then you see click here I think that is good enough because you expect people to click here to find out, I think that is too wordy, click here to find out more or click here alright. That will help you in your decision aid because basically you want to tell people what this really can help you so.

IC: This is actually showing you how to navigate because we also have some previous issues like the navigation was confusing apparently. So this is just introduction.

ID: I do not know, can I go back?

IC: For the other ones yes, for this one no.

ID: Okay why I say that is that I noticed you all are not consistent in your clicking. Like click here to continue and the other one is just forward this and that so my suggestion is that please give the person knowing that this colour whatever it is because you all are talking about same Powerpoint slide. Consistency is very important that’s what I feel, click here is to continue alright if you are going to use this colour then use it the same. Another thing is that I do not know why you all choose orange when already for the highlighted word is already cancer follow-up. Don’t use the same colour, that is my opinion. So can I move on to the next one?

IC: So press the arrow.

ID: I noticed that you all use very long sentence like who is it for, why is it for, make it short that means your phrase is short so that at least you capture, people do not need to who is this for. So I feel like the phrasing may be make it short so that people can straight away understand. And I noticed also you all like to say it in a full sentence but for Powerpoint slides I think I don’t know I personally feel that it is not necessary. And you see your colour of this you notice that like for example this colour with the word diagnosed is like it wasn’t clear the wording it doesn’t stand out. So maybe, am I too critical?

IC: No it’s fine.

ID: Maybe the colour that you choose alright to make sure it complements with the words I think that is very important.

IC: But actually this will be in a website format but I understand what you mean by like it might be a bit too wordy.

ID: It’s too wordy that’s what I feel. Another thing I think the colour sorry seriously it wasn’t very attractive to me.

IC: I understand.

ID: I mean that’s my personal opinion. This is basically your content page right?

IC: Mm.

ID: I don’t know, I find that…I like this portion here. It is good alright but I felt that… because this is more like a manual right. Why do we need to have this statement you know because it is main menu of the decision click end of this. I think basically for this statement just put a note below or whatever some suggestion that you know read through the order but I don’t know personally I find that this is a bit too wordy. Must I move on to one by one?

IC: Yup you can start with the first one.

ID: I like this portion here with the exception of colour. I like this portion here, it is good because it is basically telling you the journey alright. My apologies but I find that whoever started up this tends to give more information to the reader. That’s my impression that the person who did that wanted to pour out a lot of information. I think basically, generally I find that it is a bit too wordy that’s all. Too wordy and the person is trying his or her best to write in a very complete sentence so as a result I mean if it is literature I agree but if Powerpoint I just want to get the facts and say why is it important. Worried about you know possibility of cancer coming back, I think that the impact that will go in but I realised I have to read so many things and then after that I don’t know. That’s my personal view. Whoever the person please don’t scold me.

IC: It’s okay (repeated). We really appreciate your comments as well.

ID: And the thing is that you see you all have no consistency you know on the title because when you all doing designing all these things I felt that be consistent, this is like a heading. Okay let’s go back to the Home again. I know that you are trying to match with the wording here, the colour but then you realised (repeated) that it is very close to all the main words that you are using, the same colour. So I think whoever designed this maybe have to take into consideration the colour scheme that you are going to use, please don’t scold me.

IC: Whatever you say will be really helpful.

ID: You see this one, click onto each icon, I mean definitely we need to click on to find the possibility but I need to click on this one to find the possibilities I just too wordy if you ask me.

IC: Sorry can pause for a while. So I just wanted to check with you, just wanted to ask about so just now I mentioned about amount of information, clarity and presentation of information. For the first part which is the cancer survivorship, the first one with (ID: diagrams) you said just now the amount of information seems to be wordy right?

ID: It’s too wordy.

IC: Okay.

ID: Too wordy is too much is it?

IC: Okay. And then but is it easy to understand, difficult, a bit of difficulty?

ID: It is easy to understand but I think basically as a reader I don’t think I want to read every sentence just to find out what is it unless I really (repeated) need it.

IC: I understand. Then the presentation, the look wise will probably be, poor, fair?

ID: I will give middle, fair.

IC: Then the physical effects?

ID: You mean the moving all these I think it is okay.

IC: No the physical effects the second part. The one with the chemotherapy.

ID: Physical effects erm I think that the information is good. But as I say again on the whole, I think the person who designed this tends to want to write this in a complete sentence and I find that the whole thing is a bit the wordy is too much. So, I would say generally I feel that the whole thing is that the feel is they should reduce the number of words and also may be good is the colour, the choice of the colour is very important.

IC: So the presentation would also be fair, poor?

ID: No la, don’t give people poor, very bad. I would say fair.

IC: Then is it easy to understand?

ID: Yes I think information is good.

IC: Generally easy to understand, it is just wordy?

ID: Yes, wordy only that’s all.

IC: Then the emotional effects, the third one?

ID: Which one you want, the first one is what?

IC: The amount of information.

ID: I would say give them a good.

IC: Just right?

ID: Just right.

IC: Then clarity of information?

ID: Okay also.

IC: Easy to understand?

ID: Easy to understand.

IC: Then presentation?

ID: Again same thing wordy, too wordy.

IC: Still fair?

ID: Fair.

IC: You can go to the next part. So you can actually click into just now the page, can click into the different people there.

ID: Mm hmm. I do not know, my comment basically this is your information right (IC: Mm), why is there a closed bubble, the words is like more how shall I say my first impression because why it is bigger, the font is bigger than this. So, basically what I want to see is the information but instead straightaway that one the first impression is to close bubble. I mean that’s my thought. I hope this is not done by you.

IC: It’s a team effort (laugh).

ID: (laugh) Don’t come after me. I would find that at least this area, this is good. Can we do the follow-up, I think the follow-up care option so far I see this I think this is the best.

IC: Mm. So I think actually there is another page on the follow-up one. Maybe press this one.

ID: I would say at least the follow-up care one at least I think it is well done if you ask me. It is good. You see I click here usual care, so okay let’s start again. You see my shared care, they ask me to click the button, how you follow-up a change under right? So you basically give me this, and then what am I supposed to do? You get what I mean or not?

IC: Yeah (repeated).

ID: So I would be thinking what am I supposed to do so I click on to this shared care so I find that there must be some

IC: Interactive portion?

ID: Ya. So this would be comparing the options.

IC: So for the just now that one, the follow-up options?

ID: I would say that the follow-up care option was well done.

IC: So, just right information?

ID: The information was okay.

IC: And then clarity?

ID: Okay.

IC: The presentation?

ID: I would say presentation for follow-up care I would say it’s good.

IC: Oh okay can. Then the comparing the options?

ID: Comparing the options (pause)

IC: The next page also. So that one you can click on which one is applicable to yourself.

ID: Why is this underlined? Because when you underline, you give people the impression that because last time because there were some words that you all say click here. Because it is not in a button form but because yours is going to the next one you already give people a feel that this is something to be clicked. Then moving here, I can’t find to be clicked. So suggestion may be to improve on this because when many of us when we talk underline this, it may be actually telling me something that I can actually click on.

IC: Understand. Okay so generally this part comparing the options, the amount of information? Just nice ah?

ID: Mm.

IC: Then clarity of information?

ID: Okay.

IC: And then presentation?

ID: I would say okay, good except overall I think tends to put in a lot of information.

IC: Then generally so far was the information even provided at the start, was it things that you already knew or did we present new information to you?

ID: New information because I am an outsider, saw these things, certainly like the fees these things I also need to know and the parts like which steps should I go which I think it is good. The information are very useful, especially if okay I am seeing the oncologist what’s next this and that. Another thing when you are making comparison, I think yes, I think the information is good.

IC: Okay. And then do you think we should have included other kinds of information? Or this is quite comprehensive?

ID: I think this is good enough because for a start. Unless they really need more then they have to read up more. Because this is just a, to me it is just you want to introduce to them that you are going through this journey and what are the things you need to go through which I think is more than sufficient. So I move on to the next one is it, finding out what matters to you?

IC: Yup that’ll be number six.

ID: Okay, paying more, travelling longer distance to see the, actually both also can. I am fine. (reading to herself) No, it is important to me. I submit ah?

IC: Mm.

ID: So what is that you want me to do?

IC: So it’s actually each line you can choose between the left side or the right side one.

ID: So I choose already, more on the right side.

IC: More on the right side, so it sounds like you are quite okay to try out the shared care. So this one this part we actually want patients to see what matters to them and then from then on can see whether you want to try out shared care. Because some patients might not know whether or not shared care may be suitable for them.

ID: I am more concerned it’s the doctor. Sorry, that’s why I can say again maybe instead of like underline or whatever maybe you want all to highlight or maybe change the colour of the font or whatever I think that is good enough, consistency. So you want me to go to the next page now?

IC: Sorry so for this one the amount of information?

ID: Okay good.

IC: Then clarity of information?

ID: Clear, very clear.

IC: And then the presentation?

ID: Okay.

IC: Good?

ID: Good.

IC: So for this one, these are the things from previous discussions and what we thought would be important to patients as well. So are there other factors that you think will be good to include?

ID: I think this is already very informative. Especially if you give them too much information, they also cannot absorb. Especially I think this is like for you are going to present this more for people who just got cancer is it, or along the maybe the starting all these things or maybe they need to check for example let’s say over here. Why they don’t let me go?

IC: Aye? When we will be introducing this will depend.

ID: It’s meant for like those cancer survivors?

IC: It’s for cancer survivors, breast cancer survivors.

ID: Because (repeated) I find that the information was good in the sense that you got the whole list you see. Can press escape? I go to, this is number 55. Should be here. (reading to herself) When you say click here for more, I like this one (repeated) because it allows the person to decide what they want you see. My comment (repeated) is that like try to be more consistent in your colour of the, the colour of the words. And for us, we are also quite particular of the font size you know all these things. And also the button effect all these and that, because I realized that the colour of your button you all also choose according to your preference of the colour. I think consistency is very important. And the headings all these the colour choice be consistent so that people will know, you give people an idea that these are all titles. So the same thing at least we know that this is a title, what we want all these things. I know you all trying to match according to maybe the starting of the thing but to me when I want to read I want to know what is on top. Is this the title or what is it, rather than to match the colour. Then like when you underline basically when you underline, it’s for people to say I know that it actually allows me to click on to go into another elsewhere but not for you to underline the point of words. So that’s my overall comments.

IC: So these are the topics that we have. If you want, you can go into the different buttons and then just let me know if you think that if it is enough or do you think we should add other things as well? So these actually are website links.

ID : I think that the website link is good because the website link allows us even say I want to know more where can I find the information you know. And like for example (repeated) follow-up all these things so that we can follow which I think is good. Cannot click?

IC: Because I think it opens up on my colleague’s computer.

ID: Understanding this and that, other online resources then my comment is like for example let’s say online resources all these things perhaps also maybe if there is article written by, then you all can just add on every time so that at least we can know or refer back so that I don’t see once already then I don’t want to see anymore. So it allows me to keep me on say not bad, this website they also got updated information and then it will prompt us to always go and access it, information especially the online resources you know. And also maybe also like if information is up-to-date, that means it is like current, they have some new things you know. Maybe we can go in and check all this and that so at least this website will be live in the sense that everytime there is update of knowledge this thing then we go and look into it. And also perhaps also like… my comments are you talk more on your point of view like doctor who to see all these things. Perhaps you all might want to include like things what are the causes that you all would like. In cancer centre, they normally organize now because of COVID you all don’t organize last time, you all organize some things like management. How to say, taking care, you all organize things like

IC: Self-care?

ID: self-care thing. Or for example breast cancer, for breast cancer ladies quite vain, so sometimes can teach us, ask us how to use scarves all these things. Especially when my hair drop, all these things how to do it then how you dress all these things so maybe like publicize this also on the website. So, let’s say I cancer survivor my hair keep dropping, what other tips can be done. So, it may not be in the main website like resources and also upcoming like erm what you call that workshop, like upcoming courses, or whatever, or talks on the diet all these so you can incorporate it inside. And then people like am interested then they register. So that’s my comments so at least we know especially ladies are quite vain especially when your hair keep dropping that time you got worried you see. Then where to get the wigs all these things and also moving forward let’s say I do not know the profile like some of them they may also be willing to donate all these things, you all can match them. Like some of them after their wigs they don’t need already and there are people who really need it where it is in good condition, maybe can pass on to the next person. So this is something for, like you can link up with them at least cut cost for people. I mean this is my suggestion.

IC: Can. So that part is quite comprehensive the information?

ID: I think the information is quite comprehensive, like other resources all these things or maybe workshops all these things upcoming workshops.

IC: Can have a bit more on that.

ID: A bit on that so people I need some information but I actually very stressed up, I need to you know. Although we have this called the cancer support, breast cancer support and we have this, but it is also maybe you want to include that inside also say that if you want can join the support group or whatever it is so that at least people can find help. You know I need a support group all these things so can get help.

IC: So this one I will just put too little, the amount of information?

ID: No, cannot. The information it is good, but additional (IC: Additional okay) add on. It is just like an additional, this information is good so like to add on if you want to like let’s say help those cancer survivors like for example let’s say maybe you want to where you can seek help you know because sometimes it is not treatment only, the medical you know. Sometimes it’s the head that is involved so during this time let’s say of course we know that sometimes when you really got cancer that time you really sometimes become stupid in the sense that you cannot think, where you can seek help. Now we all know every time every October it’s breast cancer awareness but when you really get it that time at times you really do not know. I mean the reason why I said that it’s because last year I think one of my friend got it. Suddenly, she just become like she got breast cancer, so I say maybe you go breast cancer support group all these things and sometimes it need someone actually to prompt her you can go this (repeated) and that you know. So, she is a very intelligent lady, more intelligent than me. Suddenly I just realized how come she just suddenly just cannot think well (IC: Mind blank a bit) I do not know. She is a very intelligent lady but it’s just that maybe some people if they don’t want to open up, just say can go all these things. Like because this is already dedicated more to the breast cancer survivors or whoever it is so. Maybe like other resources so just like one page whatever if you need help, can seek breast cancer or the cancer society all these and that and give them the contact number all this so that at least they can call them easily, not to go and search. I mean this is under other resources, this is my comment.

IC: Okay, then easy to understand?

ID: Okay.

IC: Then the presentation?

ID: Overall I say presentation it’s colourful but as what I said it is wordy.

IC: Sorry, the other resources.

ID: I think it is good.

IC: So I put three?

ID: Ya, good.

IC: Okay. But generally looks okay but it is just wordy.

ID: Ya, I think the first time I think your team you all want to put up too much information and at the same time I think you all want to use grammatically correct English in a sentence. So I think if they allow try to be in point form I think that will be easier. Because let’s say when I read, I want to just what it is it like and just stick onto it you know.

IC: I have some follow-up questions. So you can see the questions and let me know the answers.

ID: Would you prefer a digital decision aid over a paper? I prefer paper booklet. First one neutral.

IC: Do you mind sharing your reasons?

ID: Because paper booklet I can keep and anytime I can still refer you know but digital sometimes I have to depend on my laptop I have to on it. For easy I will be there but for here I need to on and sometimes you know laptop doesn’t work all these things and it takes time. So by the time I really need to take time I will be too tired and don’t even bother. Then the other one if you are talking about this ya it is easy. Was the decision aid interactive? Yes. Please rate the decision aid.

IC: So this is what we went through just now, the amount of information, the clarity all these kind of things…the B2.

ID: Do you find the preference clarification exercise which one sorry?

IC: The preference one is the figuring out, finding out what matters to you.

ID: Okay.

IC: The one with the left side, right side one.

ID: Okay.

IC: So yes?

ID: Ya.

IC: Then B3 is in general.

ID: Mine is because slanted towards shared care.

IC: As in this one the information that we provided in general. So I understand that your preference is leaning towards shared care but then how about the information that we gave to you like when you read do you think that it seems…

ID: No, doesn’t. It gives people the option, balanced (repeated). The length of the decision aid was just right because this one need to think one you know so must be. (reading to herself) Do you find this decision aid helpful in making a decision and follow-up about cancer treatment? How so? Sorry this one I don’t understand because this is my opinion that you know I don’t mind this so what do you mean by that decision aid useful in making a decision about follow-up? I may prefer this but if there is no such facility so it…

IC: So we just want to, we are actually asking about if we do have this shared care model eventually then if we present this to you or your fellows, peers those kind then do you think it will be like helpful for example the information that we provide here or this kind of preference exercise. Do you think it will be helpful for making a decision whether or not to stay in usual care or to go to?

ID: I think if you give the patients the option, maybe this will be put up to them. So let’s say for example I don’t mind then I can try out. But if the people say that I don’t want or and you give them this they will be very unhappy so definitely they will look for elsewhere. So I think maybe this is a first start for example so do you want it and I think you give people a choice rather than you push it down to them. Because when you push it down to them, people will tend to complain, they will not like it.

IC: Then maybe I will put no, it depends?

ID: Ya it depends. I mean if talking about general, for me I am okay. Would you use this decision aid to discuss care? Yes. Would you recommend the use of the decision aid to other cancer survivors? Yes, because they are given a choice for this one. What would you think, when do you think would be the most appropriate time to introduce this decision aid? Er I think years after, C.

IC: Roughly how many years do you think will be good?

ID: I think about after 5 years when you are more stabilized already. What did you like about the decision aid? What do I like?

IC: Or what do you think is good these kind of things?

ID: I think what about the decision aid maybe you give the patients an option, the options (repeated), and the ownership. The ownership and the options because what do you want you know. But I think having said that, I think the doctor that is assigned to the patient is very important. Patients must feel very comfortable with him or her. I think most of us, we are not particular like where to, like how long how far because we don’t need to see them everyday. So is that what we want is when we see them what can they provide for us I think that is more important in the sense that if let’s say really there is anything serious all these things then at least we got someone to talk to. So I think the doctors, it doesn’t matter about the distance.

IC: It’s about the relationship between…

ID: The relationship and what can the doctors offer in the sense.

IC: Other than what you mentioned.

ID: Get the consent from the patients and then another thing also let the doctor choose the patients. That’s what I feel that because sometimes doctors and the patients don’t have the chemistry, no point forcing them. Of course, you all say as doctors you all very professional, everyone the same this and that but there are sometimes I mean let the doctor also have some say that’s what I feel. Because yes doctors are supposed to be professional this and that yes I agree but at times you really just too overwhelmed or whatever it is but of course I mean to be fair the doctors in Singapore they are all very good. So pertaining to this decision aid is that also at least let two parties decide.

IC: So who they want to…

ID: So let’s say for example give the patient the choice, at the same time also let the doctors have a say.

IC: In?

ID: In whether how should I put it (repeated) because some doctors might feel that this (pause) how should I put it. I will say let the doctors have a say because some doctors may feel that they want to er they may also want to branch out into other areas so they might want to see people of other kind so let them have a say because they also want to get other experience also as well.

IC: For the doctors who may want to recruit to be involved in this?

ID: Yes, let the doctors say rather than asking to assign them but if the doctor is more willing I think they will do a better job that’s what I feel. I may be wrong but that’s what I feel in the sense because some doctors may have certain preferences of certain areas so let them have a say. So are you all interested all this and that.

IC: And then let me say what other questions I have. So overall, including the in front I have already asked this in the front part, do you think we should include in other kinds of information other than the other resources one that you just now mentioned?

ID: I think it is good enough.

IC: Quite okay?

ID: Ya.

IC: Just quite wordy?

ID: Ya wordy, too wordy for some. This one is okay because you will need to choose you see. But for the front part, I find that as I say it is too wordy when presenting all these things, I think we need to capture the attention because you don’t forget some of them they may not like to read too many things you know. And some of them may be just, I just want to see the important words that’s all. So I felt that it is just the important words just maybe bold it and then after captures the attention.

IC: Okay. Then do you think there are parts that you think wasn’t needed, that it is redundant or not helpful?

ID: I think all of them are helpful. (IC: All of them are helpful?) Yes.

IC: Okay. So far do you think that are there medical jargons that you think is confusing or require additional explanation? Or is there words or phrases that you think might be confusing?

ID: I don’t think so but okay erm let’s say I guess they will learn let’s say for example like oncologist all these things for a start maybe people may not be aware what does the oncologist do that’s all. But once they get the hang of it, because some of them they may think doctor means doctor why got oncologist all this so the word they may not understand so I do not know how to present. Got some people they may not understand the term what is oncologist you see.

IC: Alright. I think that is mostly the questions. Thank you so much. Do you have other questions for me?

ID: No, not at all.

IC: Then just need your help to answer these questions. So I will just write it down.

(filling in data collection form)
