# Supplementary material for: Development and pilot testing of a decision aid for navigating breast cancer survivorship care
Source: BMC Med Inform Decis Mak. 2022 Dec 15;22:330. doi: 10.1186/s12911-022-02056-5 (PMC9753367; doi:10.1186/s12911-022-02056-5)
Supplement: Supplementary file 5 — Additional file 5. Transcripts and the final decision aid prototype. [file 12911_2022_2056_MOESM5_ESM.zip › Additional file 5/ID15_transcript.docx]

**Study ID**: ID15

**Interviewer**: IC

**Date**: 18 March 2022

**Transcribed by**: KY

IC: So, we’ll be going through this decision aid together one page at a time. This decision aid consists of 5 key sections as you can see over there, these are the 5… the key sections. And then as you are viewing each page and section, tell me out loud any thoughts that go through your mind. I may also prompt you with some questions along the way as you navigate through the pages. So, for example, for each of the section, can help me tick the amount of information – is it too much, just right or too little? Then the clarity of information – was it confusing? Or was it easy to understand? Those kind of things. Then the presentation wise, how does it look? The graphics, the aesthetics, those things.

ID: This presentation wise, meaning what?

IC: How do the whole section look like, like visually.

ID: For the cancer patient?

IC: Later on, when you see… so like does it look nice or is it interactive enough, those kind of things. Or do you think that we should use for example, different colors, fonts, this is just purely visual. This one is for pure visual. Okay, then once you’re okay, [ID: Yah] then you can read this one and then click the … use the mouse to click.

(Start reviewing)

ID: This one?

IC: Mm.

ID: Can I click here?

IC: Yes.

ID: Should I move the arrow?

IC: This is to show you how it’ll be like. So, you can press the ‘click to continue’, the underline portion.

ID: Click to continue.

ID: Should I click the diagnose?

IC: This one don’t have any clickable portion, so you can click the arrow over here, done with this page.

ID: The arrow? [IC: Mm] Okay.

(Reviewing)

IC: Actually, do you want to go back to the home? Can you press the home button? (Click) Yah, and then maybe start with the first one?

ID: Okay. Doesn’t… (click) okay.

IC: Just a bit laggy.

ID: So, I click the next one?

IC: Mm. Click on the arrow.

(Reviewing)

IC: So for that first section which is the ‘What is Cancer Survivorship?’, so you can help me tick the amount of information – do you think we should add more or if it’s just nice or there’s too much information? Then… (ticking) So the… it’s difficult to un-it’s a little bit ambiguous, is it?

ID: Yeah.

IC: Which part do you think was the ambiguous one? Can go back to the previous section? Yup.

ID: I think is the possibility of the cancer returning.

IC: That one is a bit not clear, is it?

ID: Yah.

IC: Okay. Then…

ID: So I go to the next one?

IC: So sorry, so do you think we should include other kinds of information for this part, the main introduction?

ID: Maybe can explain more about how possible that it will be returning.

IC: About the relapse?

ID: Yah, it may be on… maybe to get more different type of medicine or maybe to advice certain exercise or whatever.

IC: To reduce the recurrence?

ID: Yeah.

IC: Okay. Alright, can. Then you can click on the next.

ID: I go to the next one? (laugh)

(Reviewing)

ID: So for this one, should I click?

IC: There are actually still some other portions so… maybe you want to click on the long-term effects and late effects?

ID: Long-term effects, okay.

IC: That one is not clear that it’s clickable right?

ID: Mm.

(Reviewing)

ID: What do you mean by effect of here after treatment is completed?

IC: Mm, okay. So, when we refer to treatment, it can be... it’s usually about the primary treatment so the chemotherapy, radiotherapy, those kind. But understand that because you still take the medicine, is it?

ID: Not on cancer medicine. [IC: not on cancer medicine] Cancer medicine only taking for 5 years.

IC: The 5 years, right? But that one you will consider as treatment as well?

ID: Yes.

IC: Okay.

ID: Should I press anything?

IC: The… so this is it for this page so you can click on the arrow for the next page.

(Reviewing)

ID: In fact, I have taken all these, chemo[therapy], I have radio[therapy], I can’t understand about HER2+ cancer therapy.

IC: It’s a targeted therapy only if you have that marker in your cells then they would suggest this kind of therapies for you. Like we mentioned just now, like different people, different kinds of treatment is more suitable for them.

ID: So far, I only gone surgery, radiotherapy, and chemo[therapy], I have not gone for all these…

IC: The 5 years one?

ID: 5 years? The medicine, the hormonal therapy, is it?

IC: Yah. Or which one do you take?

ID: I can’t remember.

IC: Tamoxifen or…?

ID: Yes, I think I have all the medicine I’ve taken. I kept here even though it’s done. Let me see. [Be]cause I need to put it in this because my children has to know what are the medicines that I’m taking. So, anything happen, at least they can refer [to] this. (Reviewing medicine list) This one. See, I even have the heart medicine also.

IC: Okay, I see the one for the bones, understand.

ID: Then that’s the last one because it’s an old one. Current one is… so I do take Lipitor, what else… biomedic (likely painkillers), those are biomedic (likely painkillers) and Lipitor, that’s I think the main one.

IC: Can, so this one is the ones that treatments that you went through so like you mentioned the surgery also right, radiotherapy, chemotherapy, you can click on the next buttons… can actually click on the buttons.

ID: Surgery? Okay.

(Reviewing)

ID: So this is the explanation when you do the surgery, is it?

IC: Mm.

ID: But I think it’s really successful that [IC: for yourself] [be]cause I don’t feel the pain. [IC: that’s good] I even tell them said, “can I turn to my right-hand side?”, you see no problem. I worry because there’s a… the operation… So I say it’s okay if you feel good, so far so good. And you only pain when they just take out the plaster, that’s all. And you know they have something like they put my rolling (unclear word) or something like that, they collect something…

IC: The back that has, is it?

ID: Just for only about a few days, after that, okay. So far so good.

IC: Recovered well also?

ID: Very good.

IC: Was it done in NCC or?

ID: No.

IC: Somewhere else?

ID: My clinic, the specialist because its company sponsored (laugh).

IC: That’s why…

(Reviewing)

ID: These skin patches, it’s only a very short while, not say remain forever, right? It’s only for short while, they don’t… beginning I also worry. They say will turn black you know, I very worried but so far okay. Heart disease means for the left side cancer, but mine is the right-hand side.

IC: So, it’s less likely for yourself?

ID: Yah.

IC: For that one.

ID: When I do chemo[therapy], I only lost my hair (laugh). Totally all... all over my body the hair, include my this, everywhere.

IC: Even the…

ID: My eye, this one also. Totally, bald.

IC: But after that it came back?

ID: Yah, it came back but getting lesser, my hair. Beginning, I did not know about it. When I take a shower, I use… suddenly I found all my hair…

IC: It came out like…

ID: Like one lump like that, so I quickly get my husband to shave out my hair.

(Reviewing)

ID: Tamoxifen. Hot flushes, yes.

IC: That time you had this also?

ID: Yah. So far, I don’t really have those blood clots or urine bleeding, so far no. So far so good. Can I go next?

IC: Yup.

ID: Do I have to tick the?

IC: Go to the next one first, then after that both the effects parts.

(Reviewing)

IC: So, for this section, can tick the 3 of them. (Ticking) And then, just one question, so overall, those were things that were quite relevant to you as well because you went through it right?

ID: Yah (laugh).

IC: Do you… was there any other information that you think we should have included for the physical and emotional effects?

ID: Not really, the only thing… my family more worried when I’m breathless.

IC: Breathless? Restless?

ID: Maybe because I don’t know, is it because ages or what? Because I’m so used to be a sportswoman you see….

IC: Restless?

ID: Yah, I used to be sportswoman, so I don’t think that is the cause of what, so I think maybe ages?

IC: Still quite active now?

ID: No more.

IC: Not as much.

ID: Especially now when I look after my grandchildren (laugh).

IC: But they also run around?

ID: No, not really, because they move out already so I don’t look after them.

IC: Okay, so there’s less.

ID: Less, that’s why not before but maybe in future there’s one more coming up (laugh). Because they stay with me, if they move out, then I don’t have to look after. But they stay together, or sometime usual ones to play with. Okay, just, I’m… I really like children maybe because I got my children… 10 years after marriage. I brought them 10 years after marriage so.. (laugh).

IC: But now there’re quite a number of grandchildren as well?

ID: I had one already which is coming to 5 years and 2 more coming up.

IC: Wow, okay.

ID: So different a few months, one is coming next month and the other in September.

IC: Your family will be quite busy.

ID: Not really because I only have 2, my eldest now is born in 1988, 35? 33? Then my son is 28. My daughter will get another one, my son will get the first one (laugh). So I think it’s okay. It’s good to have them because you know you have something, and you don’t feel bored when you’re alone, always think of your sickness all these, so it’s good to have like something. But they always tell me say “mummy you go outside and do something, you don’t have to stay at home”. That’s one thing good about my children.

IC: Okay.

ID: Then I go to the next one?

IC: Yup. You can press the…

ID: What they say join the… (laugh).

(Reviewing)

IC: Okay. You can press the next one.

(Reviewing)

ID: Like I say, maybe like what they say once a year, I may go for one more so… so far, I have referred here I think 3 years already? Around 3 years? I think they have not done the bone mineral test density.

IC: Have you talked to Dr Rose or the doctors about it?

ID: I do told her said that in here, they did not have like taking… like I said earlier, blood test, scanning, MRI, and then they even go for my scanning on the… whether to see my… the heart and everything and the bones so… But here, they have not done so far. That’s why I was wondering…. that’s why my brother say it’s cheaper because their… they don’t look further, they only go for one unless you complain something and then they go for what MRI all that.

IC: But do you prefer having more tests or… is this system fine for you?

ID: Unless… like bone mineral… like I said, maybe ages. When you are ages, your bone is getting weaker. But according to my oncologist, he say because due to your cancer, it maybe cause, and your medicine may cause your bone. So I can hear that, so far okay (laugh). And blood test say, so far even my main, only once in a while, I feel very tired, not like some people they can’t even bend their knees you know. So I say, so far so good, and my sister, they are very happy to see me. They say “you are stronger than me” (laugh). Then she said, “but you go for bone test and everything”. Then I say, “yah, so far it’s okay” and I still can like, they say now Malays, some of them they had to pray they have to sit down and like what I said. And some of them, they can’t do that. They have to sit on the chair to do the prayers. So my sister says, “so far so good, I can see you still can do that” and she’s so happy to see me sit down on the floor. She says she can’t! She has to sit on the chair, and she need the higher chair, she cannot take the lower chair.

IC: Because it will be painful for her?

ID: When she stand up, she got problem. That’s why. Mine… so far okay.

IC: That’s good.

ID: This... I think these are the things that they are follow[ing] up.

IC: But yourself currently, it’s not doing this?

ID: Well... (laugh)

(Reviewing)

ID: This is Dr Rose’s advise me to go... types of family doctor. So in another to say, I can ask my polyclinic to see the special doctor to look every visit?

IC: Yah, so there’s family clinic per se…

ID: I used to go for polyclinic

IC: At the polyclinic.

ID: Yah.

IC: So, those doctors is more likely that you go back to the same doctor compared to…

ID: Yup.

IC: Normal polyclinic. So some people prefer that to see the same doctor, so that’s an option as well. But this one are usually… they will only offer you… the polyclinic will usually offer it to you if you are for example, if you have a chronic disease like diabetes, high blood pressure, those kind of things. Or else, you could ask them directly like “I want to see a family doctor” and then they probably move you to that one, but it is a bit more expensive compared to the usual…

ID: So far, I only went to see them for my diabetic and my cholesterol, so I don’t think I need to have more specialized….

IC: If it’s fine so far, it should be okay, but if you want more then we have… there’s this other option.

ID: Yah.

(Reviewing)

ID: I think the below one is correct they say. They may or may not ask about your cancer history.

IC: That’s what happened for you is it?

ID: But sometimes I do mention I have cancer, so I just tell them, is it clear to my cancer? They say no (laugh). It’s okay.

(Reviewing)

ID: These are the place[s] where I used to buy my medicine, that’s my… more for my what they called it…

IC: Supplements?

ID: Supplements, yah. These are the place (laugh). And I used Watson’s because I have my member’s discount. So that’s why we used to go Watson’s. Guardian, Watson’s sometimes when Watson’s don’t have, then we use Guardian. Unity is… very few. Okay, so should I go next?

IC: Mm.

(Reviewing)

ID: Okay.

IC: Can you go to the next page?

(Reviewing)

ID: So this is what we’re talking about.

IC: Do you want that…

(Reviewing)

ID: Maybe I should go for my shared care (laugh). But because if I ask for the other doctor, like I say those who knows about my cancer. But will it be together with the others doctors? Like he or she may know about my others sickness, the diabetic and cholesterol all these?

IC: As in at the polyclinic is it?

ID: Yah, the shared care. I need someone the doctor which know all my sickness.

IC: So the idea is that with this kind of fixed family physician primary care doctor, they will have information about your cancer as well as your chronic conditions like your diabetes, those things as well. So, during the same consultation they can go through both side with you.

ID: They can go through of…

IC: Mm. Because now it’s quite separated.

ID: Yah.

IC: But you prefer to go through everything in the same consultation per se or…?

ID: Because like for here, I go more on the… just the mammogram and the result, that’s all only. Like if I go poly[clinic], I… at the same time, I can also ask about my cancer progress as well. So, I think I should go for the one… shared care (laugh).

(Reviewing)

ID: See every 3 months, so I think it should be better. Should I go next?

IC: So, the usual care and shared care, so each portion. Then the shared care…Okay so far? {ID: Yah} Any other information you think we should include inside? Or what other things you might want to know for example?

ID: So far, I really appreciate to have the shared care (laugh). I think it’s easier and… but because like here, I only go once a year. So if I go shared care, at least after, within 3 months I may know how is the progress. So it should be better to have the shared care.

IC: You would prefer to have a bit more like… bit more frequent kind of visits?

ID: Yah. Some people may say, “maybe I free cancer, that’s why I don’t have to go so often”. But at least you get to know every 3 months like I say, you can… in case of anything, you know earlier rather than you wait for another 3 months. So, I think like that is better.

(Reviewing)

ID: So, I click that arrow?

IC: Yup.

ID: What do you mean by community pharmacists [are] usually not involved?

IC: So in the shared care, they will be the ones calling you every 3 months just to check up on you. And if you have any questions about your medications those kind of things, you can ask them. But in the usual care, they don’t have this kind of role of role for your care. The most that you will interact with community pharmacists during the usual care will like you mentioned, when you go to Watson’s, Guardian, this kind of places to get medicines or if you want to ask them some things. So, they are a bit more involved during shared care.

ID: Okay, So I go next one.

(Reviewing)

ID: This consultation cost is in… for the oncologist is… like for the in… those the under the cost there, they mentioned that amount right and each of them like… general clinic is this and for pharmacist walk-n store is free. Special arrangement… oh 20 dollars for that, okay. So this one they are explaining for both usual care and shared care? The price, the amount?

IC: Yah, because the total amount, it will depend on your number of follow-ups which would depend on the individual person, so it’s hard for us to like give you an exact number on the spot but this is just a rough number, for example. You can roughly see like if you calculate, for example, if you have 2 visits with the oncologist and then you replace one of them with the family physician, then it will be a a bit different the cost, depending…

ID: This one I click Singapore citizen, permanent resident, different different prices.

IC: Yah, because of the subsides and things like that as well. So citizenship would be a bit different.

ID: So my case is Singapore citizen or permanent resident, it’s Singapore citizen, right?

IC: Yours is the red IC right?

ID: Yah, Singapore citizen. Permanent resident got higher subdize? Higher subsidies.

IC: So this one is the amount that you pay, it’s not the amount that is subsidized. [IC: okay] So that part may be a bit confusing? [ID: yes] Okay, I understand. So like you can see this is the non-residents [and they] are not even subsidized. [ID: yah, alright] Okay, can, we will take a note of that. (laugh).

ID: That’s why how come the subsidize is lesser? So, this is the charges.

IC: That’s the charge.

ID: Okay, so I go for the next one.

IC: So this one is the comparing the options, this one here. So, the 3 again.

ID: Same… just right.

IC: Generally the information presented so far, it’s okay?

ID: Yah.

IC: Anything that we should include for example?

ID: So far, I can’t think of anything… because so far, I have gone nothing much on this, so maybe the next visit?

IC: Okay, then the next part.

(Reviewing)

ID: So if let’s say I take the shared care, so how to mammogram? I still come back here, right?

IC: For the current… so we are actually trialing this shared care model currently in the current format. You still have to come back.

ID: Because I think if I’m not wrong, I read just now they say mammograms still here and the results, the rest is still just like 3 months just like you know.

IC: Of course, we are looking into whether or not we can do the mammogram in the polyclinic but it’s still in the works.

ID: Yah, but sometimes like I’ve said it’s not... I didn’t say everything; I didn’t complain. It’s just comparison which I find that mammogram when you do in government all these, it’s not the same as the way they do in the private.

IC: Because you went through both.

ID: Yah. I ... the last visit that’s the best mammo[gram] I did before.

IC: At here?

ID: Yah, here. That’s the last… that’s the best because I don’t feel the pain and I find that she do it very nicely and she really encourage you to do, how to be you know. That’s good so far, I find that is good.

IC: So, in NCC, doing the mammogram at NCC is fine.

IC: Yah, it’s okay.

IC: Good so far?

ID: So far, she’s the best.

IC: Currently you have done 3 mammograms here? Year 2, year 3?

ID: Of course, I did the, the last time when I do my health screening, I did that also and that’s how they found out my… They really do it good but it’s just like its pain for me. But here, they do good, and I don’t feel very pain, not so good… I mean not so pain as before, maybe because the signals is there, that’s why I feel the pain…

IC: Maybe it’s the way that they do…

ID: Yah, they don’t really… that’s why she really will…. She really do it nicely, say “you know this part, you don’t have flesh, it’s no more flesh, sorry will feel pain” She explain. That’s good. I go next?

IC: You can click the arrow there.

ID: Do I have to put a tick on it? No?

IC: You can if you want.

(Reviewing)

ID: So far almost all for shared (laugh). I mean cheaper don’t say cheaper, I mean of course everybody wants to have the cheaper one but I mean if you think that you want everything but you want cheap also… so it’s very hard.

IC: So for this part, just wanted to ask you like these are the things that will be important to you when you deciding on the kind of care that you take up?

ID: Yah.

IC: Are there any other factors that you think will be important?

ID: For the care share?

IC: So like that is not here but then like things that you think would influence like if shared care doesn’t have it then you will want to go back to usual care kind of thing.

ID: I think should be okay.

IC: Mostly there? Okay can.

ID: Unless I go through, then I will give the feedback again. So, it depends. [Be]cause right now, I’m not in the shared care, so I was thinking that if I can try, then I can give more feedback on it.

IC: Then if it’s okay, then you can click the submit the results part. So for this section, what did you think was the purpose of this section like in your opinion?

ID: My opinion that is to save the time (laugh) which is… and to get a result faster or better you know your progress better, rather than you wait for the next visit.

IC: Okay. It’s for the shared care?

ID: Yah, for the shared care. I mean for primary… I mean those like for normal care, you have to wait for the appointment. But in this case, within 3 months roughly you know what’s happening. It’s just like last time when I didn’t know that I have the cancer and it takes how many years to know the results. That’s why I’m so surprised 2001 and I gone the biopsy for 2019, that’s how many years. Been there, but luckily it didn’t grow big. According to them, it’s a mir- not much, not big enough.

IC: Not too much change?

ID: Not too much change. Not big, the growth is not big but still… [IC: still there] Like what they say, I’m so surprised they say when I’m in the stage 3. [ID: that means, okay] Is it due to your what they call it lymph nodes? [Be]cause of my lymph nodes, that’s why they call it as stage 3.

IC: It went there, is it?

ID: Yah. They count the number of lymph nodes, that’s how they count the stages. I’m so shocked also, I’m in the stage 3. But usually people will say, stage 1 you know, I… In fact, before I got it, I have my ex-colleague from the previous company, she just comes and “you know something, I have my breast cancer”, and she say hers is stage 1. But she has to remove, she has to remove all. I don’t understand but when mine is stage 3, why I don’t have to remove? That’s why I said, “I don’t know”. She said hers is remove one side. That’s why I say, okay, mine so far so good (laugh). That’s why, but okay. She also survived now.

IC: But still okay so far?

ID: She got it I think a year or 2 years before me, then she migrated to New Zealand.

IC: Still there now?

ID: Yah, she stays there with the family. Okay. I don’t know, I always gather with all the people with having the same… the same sickness. Shall I go further to the next one?

IC: So, for this part, just tick the 3 again.

ID: I don’t know whether to consider too much or too little.

IC: You can put in the middle then.

ID: Yah, for the time being.

IC: Or I mean like too... in the middle here but…

ID: I don’t know.

IC: Why did you think that it was… it might have been too much information for just now that part?

ID: Which part? The matter?

IC: The ‘What matters to you?’ part.

ID: Actually, it’s just an explanation only, they explain for you too much, but I find the… okay. I mean like…

IC: Is it necessary, it might be a bit too much right?

ID: Yah.

IC: Okay. Then do you think that the checklist itself, is it helpful for you or will it be helpful for people to make a decision on whether or not to go to shared care or stay in usual care?

ID: I feel it’s good because everybody will think that with every visit unless they look at it. Then they can compare the symptoms, so it’s good to have a checklist. If you do your work also sometimes, it’s good to have your checklist, because sometimes you may forget. So, if you have the checklist, at least they can see oh maybe I miss this one, it’s a good addition.

IC: That’s good. Then, you can click on the... click here for additional resources. So this part, just need your help to just maybe just look through these resources and then like… let us know if there are other things that you think we should include inside. So, these will actually go to websites.

ID: Can I go my… webs to look at all these? No?

IC: As in this current one?

ID: I mean if I were to refer home, can I refer from here? No? Can I get online?

IC: So you mean this whole decision aid right? [ID: Mm] So, because we are still trialing it, I’m not sure… it’s not out yet. But eventually, if we do have that shared care option that shared care program out officially, then we hope that this will be put… its actually supposed to be a website kind of format. But it will be good for you, is it? You think that this…

ID: Yah, sometimes when you have nothing, you can just refer. Just like you stay at home you got nothing to do, watching TVs, but at least you can read through all these, more information for yourself, rather than you come here then you can see all these. Sometimes when I go for Facebook or what, they advertise on the certain sickness, I go to read through all very long. So at least they can explain from here, that’s good.

IC: Maybe I’ll check with my colleagues if it’s possible we send you a copy or something. But officially, it’s not out yet. That’s why we are trying to see if… how possible, how helpful this kind of things will be. So, you can click on the buttons to go to…

ID: You may redirect the external website.

IC: As in it’s inside the buttons, so you can click on the buttons itself.

ID: Which button? The website? Or anyone? Okay.

IC: Then the websites are inside the next page. (click] Yah.

ID: Okay.

IC: So like you can let me know like if there are other information that we should include or you like us to include.

ID: I think it’s good to have this, but I don’t think that overseas-based website is useful.

IC: Less relevant?

ID: Less relevant. Sometimes… I don’t know, but I find that local is more useful. And I noticed those in Facebook all these are more for overseas, that’s why it’s very lengthy and then you read until you really… wow. At least here you know you want to understand, okay, you go to breast cancer and understand; you want cancer survivorship, you go there. Rather than you read through whole thing, my goodness. That’s good, this one. Shall I click close?

IC: Go through the other ones also.

ID: For breast self-examination, I mean sometimes I tried to do it, but I cannot give a result whether is it or is not. I don’t know how is the examination.

IC: I think that one cannot open from there. But this kind of things will be important like things that you will want to know?

ID: Yah.

IC: So I think cannot go to the website.

ID: Yah. Okay. Healthy eating, I see a lot. I do read through sometime about cancer and then some people say cancer cannot take meat, this, and that, but I don’t know like I say, different person, different treatment and how is your body at, that’s why.

IC: Sorry, [be]cause this one is the company laptop so it cannot go into the websites.

ID: Yah, doesn’t matter.

IC: But I will check with my colleagues if we can send you at least this resources list.

ID: I can’t close it.

IC: It looks different. Okay.

ID: I think all these cannot open.

IC: Cannot open sorry.

ID: I think it’s okay.

IC: But these are things that you want to read up also?

ID: Yup.

IC: Can click to…

ID: Close. Type of treatment so far, I think I go through just now, so it’s okay.

IC: You want to go in to see what’s there or?

ID: No, I… it’s okay (laugh). I think in fact, I’ve gone through that’s why I know what is happening.

IC: Okay, can.

ID: So, I go home?

IC: Yup, so, that’s it actually for the decision aid. Thank you so much. Then can you help me do this one, the some… for the other resources? Just now that list.

ID: Yah. Nothing much, just see, so.

IC: But do you think the ones that you saw, the websites are comprehensive like there’s enough websites that?

ID: Yah. Should I put it under understandable with some…

IC: But what would the, what is the ambiguity or like…?

ID: How to explain…

IC: Is it confusing or not as clear?

ID: Too much.

IC: A bit overwhelming?

ID: Yah.

IC: Then this one would be this one?

ID: Yup, difficult to understand or understandable with some…

IC: This one is how it looks. Okay, can, thank you. Then I just have a few follow-up questions. So, overall, how did you find the decision aid aesthetically? So visually about the appearance features like the color scheme, choice of font, font sizes, those kind of thing.

ID: That’s not so particular for me. (laugh) Neutral.

IC: So quite neutral?

ID: So the B2…

IC: Then all of them, we do this one first.

ID: Okay.

IC: So this one, help me answer the questions. Both are of okay, paper or digital. The navigation generally okay. Were there some parts that’s a bit confusing about where to go? It was not… it’s not interactive, is it? Do you think it should be more interactive or like this is actually okay even without the interactive elements?

ID: Should be… not really… I mean like what I say so far, I’ve not done everything and I’m not sure how is it you see.

IC: I understand. Okay. This one is the checklist. Think you talked a bit just now. Like if we do have the program out, the shared care program out, then do you think that the checklist will be helpful in your decision-making?

ID: Yah. The length of the decision… (laugh) okay, just right.

IC: I think because we were talking a bit just now so it take[s] a bit longer.

ID: Yah, it’s true. Because I think if let’s say they are no …

IC: if there’s nobody talking to you, then it’ll be okay. So like what other things you think would be helpful or useful inside this whole decision aid that can help you make a decision about the follow-up care?

ID: I don’t know how to say.

IC: Like generally the information provided is informative and useful?

ID: Mm.

IC: Okay, can.

ID: Good advice, ce or se? I always confuse. I think is se.

IC: I think is se also. (Completing questionnaire) The active treatment, it also includes your 5 years, the tamoxifen. Then do you think that we should give the… this decision aid, we should introduce this decision aid like after chemo[therapy], after radiotherapy, during the 5 years or?

ID: I think…

IC: When is a good time? Or when would you like it?

ID: You can even give them after treatment also, but I think it’s good to have given during treatment, the same time you can have… at least they can think about it. Rather than you wait until… so, I think it’s good during the treatment instead of year after active treatment.

IC: During like for example during chemo[therapy]?

ID: Yah when they do chemo[therapy], then at the same time, you can also explain. Because I’m sure you go chemo[therapy], you can explain maybe this way then this one, let’s say if this one; it’s good at least at the same time, we can just think about it. Because if you wait until they already done for years, then after that now then you tell me all these, so it’s good to have during treatment.

IC: Introduce early on?

ID: Yah.

IC: Okay can. Are there anything else to add for this part.

ID: No.

IC: Were there any information that you think we should have excluded or it’s like redundant kind or generally everything is comprehensive and enough?

ID: I’m not sure because you know why, when you are working, you think a lot; but you are not working, you can’t think of it unless you face it. Because when I [am] working, I do a lot of things that I need to improve things so you will always think of something else. But because when you are not working and you are not doing anything, you can’t think of it you see, unless like maybe… like I said you are working, you can think of how to improvise this, how to improvise that. But for me because this is the first thing… the first time I do, maybe in future, I can… oh next time I can tell them do this, I can tell them to do that. So, not at the moment.

IC: Okay, can. Thank you then I think that’s about it. Just need your help to fill up this, it’s the demographic survey.
