# Supplementary material for: Development and pilot testing of a decision aid for navigating breast cancer survivorship care
Source: BMC Med Inform Decis Mak. 2022 Dec 15;22:330. doi: 10.1186/s12911-022-02056-5 (PMC9753367; doi:10.1186/s12911-022-02056-5)
Supplement: Supplementary file 5 — Additional file 5. Transcripts and the final decision aid prototype. [file 12911_2022_2056_MOESM5_ESM.zip › Additional file 5/HCP08_transcript.docx]

**Study ID**: HCP08

**Interviewer**: IC

**Date**: 6 April 2022

**Transcribed by**: KY

IC: Okay. So thank you so much for participating in our study. So we will be going through this decision aid together one page at a time. This decision aid consists of 5 key sections and as you are viewing each page and section, tell me out loud any thoughts that come into your mind. I may also prompt you with some questions along the way as you navigate across the pages. So for example, I might ask about the accuracy of the content, about the comprehensibility of the language – is it easy to understand, those kind of things. And then appearance wise, what did you think about the decision aid aesthetically. So whatever that comes to your mind, you can just let me know.

HCP: Okay.

IC: So once you are ready, you can click to start.

HCP: I can just comment right?

IC: Yes.

HCP: (Slide 15) Can this be a … So breast cancer survivors may experience, like I think make it a bit more tentative, so that not all of them feel that “I definitely will get this”.

IC: Okay. So it’s the phrase ‘breast cancer survivors **can** experience’ to ‘breast cancer survivors **may** experience’?

HCP: Or some breast cancer survivors may experience.

IC: Okay.

HCP: Survivorship care, anxious, is it just medical support or is it also psychosocial support that we are talking about?

IC: Guess the most one would be medical and physical support, but there is some parts that are emotional support as well, but I think it’s not as clear here, that means.

HCP: Yah, so medical sounds like more of looking into the physical bit rather than a more holistic view, so maybe we can rephrase or add on something also here.

IC: Understand.

HCP: (Slide 16) ‘The return of their cancer’ sounds a bit weird. Are we trying to avoid the word ‘recurrence’ of their cancer?

IC: That one I’m not sure but do you think that, so for patients, for breast cancer survivors…

HCP: Okay, KY said yes. [Be]cause it’s like a big word, is it? Recurrence.

IC: Might be a bit taboo word for them.

HCP: Okay. ‘Successfully complete’, not past tense right (Slide 16)? I don’t know, I think the returning sounds a bit weird (laugh). Returning cancers, okay, maybe never mind, let’s move on. We can think about this.

IC: Sorry, so for this part about the cancer survivorship, overall, what do you think about the accuracy of the information that was presented?

HCP: I think it’s okay, I think it’s just the phrasing. Yah, so many cancer… many people with breast cancer successfully complete instead of completed, there’s a possibility of cancer returning in the breast or other parts of the body.

IC: Okay. Then, do you think we should add other kind of information for this part, other than maybe adding on a line or a word about the emotional, psychosocial support for the previous slide?

HCP: I think it’s okay.

IC: Okay. Then do you think that there’s anything confusing in this portion? Or like things that the patients might have difficulty understanding?

HCP: No, I think it’s okay.

IC: Then how about the aesthetic wise such as the color scheme, choice of fonts, font size, these things?

HCP: A bit plain, but…

IC: Okay. So how about the use of icons, images, graphics, and interactive buttons?

HCP: Interactive buttons, I think it’s fine. Graphics… so I think the overall look is that … because I don’t know what are the other options, but it does look a bit plain. But the choice of the icon is okay, I feel. Maybe I don’t know whether it’s better if the background, instead of white, it’s something else, some other colors, have to try out and see.

IC: Understand. So, but do you think that it would be better if we like for example, add more graphics, add more icons those things?

HCP: Maybe not for this slide. [Be]cause if too many, then it will be quite messy also. But maybe the background color or the background design.

IC: Okay, I understand, thank you. So I can go to the next one.

HCP: (Slide 17) So this one, I think, do we need to also mention the acute side effects part? Those… so because it says there that cancer treatment can cause various side effects, but we are only talking about effects that affect the survivors right? That’s why we only include the long-term effects and the late effects. So, I think it has to be a bit clearer because it sounds like we are saying that cancer treatment only cause this 2 kind of effects. So whether we can add on the acute side effects which does not continue until your survivorship or we can add on a point that says that these are the effects that will affect survivors. And then the animation, as in like the… this thing, the chart, can you see my mouse? [IC: Yes] So, this thing, I needed some time to really look and comprehend the diagram. I am not sure whether it can be made a bit [IC: clearer?] simpler.

IC: Sorry which part was the… like a bit confusing part for the diagram? So when it appears, is it?

HCP: As in like when I click on it, I have to take a few seconds to really comprehend what this diagram is trying to tell me.

IC: The link is not as obvious at first glance?

HCP: Yes. Maybe like the ‘typically 6 months to 1 year’ can be on top or something. This bit is also, I think a bit too near to the end of the slide.

IC: Similar for the late effects as well?

HCP: Mm.

IC: Okay, understand.

HCP: Okay, I click. (Slide 24) Can maybe not put stroke? Can we say like swelling at arm **or** breast? If it’s swelling at breast, also it’s not lymphedema, right?

IC: Oh, okay.

HCP: Then I would think also body image concerns sounds more like a psychosocial thing rather than classify it under physical effect. [Be]cause it’s a more, I feel it’s more like a psychological concern rather than really physical, although it’s caused by the physical… like loss of the breast and scarring. And then I mean aesthetic wise, whether we can just have one line for each [side effect]. So like for this one the example right, I think we should cut down so that it’s one line instead of 2 lines (slide 24: reduced arm mobility). [Be]cause like that it’s a bit not so neat. So again, this one numbness **or** loss of sensitivity.

IC: Okay. So for the ones with the stroke, then expand it to [HCP: Or] the word ‘or’, okay.

HCP: And also, I’m really not sure swelling at the breast can be considered lymphedema. I think have to find out.

IC: We will check that one out.

HCP: Yah.

IC: Thank you.

HCP: (Slide 25) Fatigue, tiredness, I think just choose one because it’s the same thing, right? Fatigue [and] tiredness. Skin discoloration… I feel it’s quite straightforward so no need this ‘skin patches of different color’. ‘May add on to post-surgical pain and reduced mobility’ – this one is not too clear. Do we mean that there might be pain from the RT (radiotherapy)? There might be reduced mobility? (Reviewing) Sorry, is this in literature? Swelling, physical effect caused by RT (radiotherapy)?

IC: Yah, all of these is, either taken… both taken from literature and then checked with the doctors as well.

HCP: Okay. [Be]cause I don’t see a lot but okay.

IC: Might be a less common side effect.

HCP: Mm. (Slide 26) This one also, I think just either fatigue or feeling, maybe feeling tired rather than fatigue or tiredness. Then, I don’t know whether you need… if we just want lay people, then just put tingling sensation, don’t need the neuropathy. Infertility among young survivors but older also right? I think don’t need ‘among young survivors’ infertility. This part if increased risk for the following rare effect, then don’t need ‘increased risk of’, I think just put blood cancers. [IC: Okay] For ‘e.g.,’, the… do you need this comma here? Because usually I don’t put.

IC: Oh, understand.

HCP: (Slide 27) How come got all these? (Displayed confused fonts)

IC: It’s my computer font.

HCP: Sorry, so what did I click. Can we put, so this one is the drugs, right? Can we have a title to say that these are the drugs that’s classified under HER2 therapy? Because there’s no title so…

IC: So indication that…

HCP: That these are the names of the drugs. So, do I click on these (treatment icons)?

IC: It’s actually the same things as you saw, it’s just the… Some of the things my computer doesn’t read.

HCP: Same thing as I saw, meaning?

IC: The surgery, radiotherapy, chemotherapy, if that’s what you are referring to?

HCP: So you mean all these are actually like the chemotherapy, surgery, all these is it?

IC: Yes.

HCP: Okay. So it’s not supposed to be here, is that what you’re saying?

IC: It’s supposed to read surgery, radiotherapy, and then chemotherapy, HER2 therapy, hormonal therapy.

HCP: So meaning that this page, if I click on this one, it should link me to something else, is that what it is?

IC: No, it’s the same one as… its actually still the same page.

HCP: Okay, wait, I don’t understand. So if I… are these icons supposed to be here? The blue, yellow, and …

IC: Yes, because the, if you compare the sizes of the previous, the radiotherapy, the surgery one, it’s actually bigger, so that’s why it covered the whole thing. But then because that one is a bit shorter, so it didn’t cover until the labels of the icons there.

HCP: But all these other don’t have the additional icons, only the HER2 got.

IC: Yah, so actually it’s kind of like a pop-up, so the icons are here at the back.

HCP: So you’re saying that like the… even under these, there should be all those is it?

IC: So if you can see the… (IC showing) So like if you see the…

HCP: I don’t see your mouse.

IC: If you see the left side and the right side, then there is the blue color and the orange color.

HCP: Yes.

IC: So those are the buttons that, so those are the label buttons. So when you click close to return, it’s actually the same thing. Does that make sense?

HCP: Okay, so now it’s the chemotherapy, right? If I click on the orange button…

IC: So when you open up the pop-ups, you won’t be able to press the rest untilyou click back, click here to return. Yah, click to close.

HCP: Okay, wait.

IC: So won’t be able to go directly, not supposed to go directly. Then go to the next one.

HCP: I shouldn’t also have all these things, right? Because the rest of the slides also don’t have. I mean I don’t see them on the other slide, I don’t know whether it’s a computer thing. I should only have the blue and orange button at the side right, to standardize.

IC: Okay, understand.

HCP: Because like all this yellow thing is not on the other slides.

IC: Okay, so at least make it consistent whether or not to include for all?

HCP: Yah, I think so.

IC: Okay.

HCP: (Slide 32) This one, can we have like capital for all?

IC: Okay, for the letrozole and the

HCP: For the drug names, yah. Okay, so this one close to return, it returns me to this page. [IC: yes] I think it will be easier if, as in I think it will be better if it returns me to…

IC: The hormonal therapy?

HCP: This page, yah.

IC: I think why we design it as that was because most of them would have taken one or the other, so, they would press into the one that is more relevant to them.

HCP: Okay. B[e]cause I probably would want to see the other one also.

IC: Okay.

HCP: (Slide 27) I wonder if this one should be the same layout as the hormonal therapy (Slide 28). This is easier for me to understand, to click and see. So instead of listing out like that right, in one line, then maybe you can have an icon, maybe your Herceptin will be one then pertuzumab is one, then can just click. Although I don’t know whether the patients will know what they have, but they should.

IC: Okay. So for this part, sorry, so for the physical effects, do you have other comments about it?

HCP: I cannot really… wait, let me think (laugh). (Slide 27) I wonder if it will be better if this box right spans across the slide, [be]cause it looks like it’s very… I don’t know how to describe. But in terms of like design, I don’t know whether it will look better, but I think have to try out and see. [Be]cause it just look like very isolated, this thing, then one chunk here, one chunk here. Have to try and see.

IC: For all of them also? The headings of the treatment.

HCP: Yah. So this one is, if I click here… how come I go to tamoxifen? So if like that right, if I click this one, the blue color one, shouldn’t it link me to HER2?

IC: Okay, I think I know what you mean.

HCP: Because if you click all these it should link me to the next…

IC: It’s actually just the background, it’s not linking to anywhere.

HCP: Just the background? [IC: Yah] But I think if we want, as in we should standardize. So, if let’s say, like for example, if I click on this, it should link me to the next topic right which is the hormonal, or something – HER2. So if I click on the orange color, it should link me to hormonal, so then there shouldn’t be any more orange here, unless you want to link back to surgery. It shouldn’t link me to this tamoxifen. I don’t know whether, you get what I mean? (Navigational issues with the treatment buttons).

IC: Yes.

HCP: Can I continue?

IC: Yah, sorry, wanted to ask some questions about the content, language, and appearance. So, overall, do you think that we should add other things in this portion, the physical effects one?

HCP: Physical effects…

IC: Like are there other information that you think would be relevant to add inside here that will be helpful for patient to know?

HCP: No but actually I’m thinking whether it will be better if this thing is designed in a way that maybe like under physical effects, you have a[n] image of a person, of a human, like a human figure. Then, the person can actually like for example, if I have like swelling, so, they can actually click on the arm that leads them to lymphedema. So rather than associated with the treatment that we… that they have received, it will be them clicking on a body part, on the things that they experience, as in on the physical effect that they experience.

IC: The visual aid for them.

HCP: Yah. Because I might not know what I’m experiencing is due to what right? Like… I might not know that the fatigue I’m experiencing is due to the chemo[therapy], it might be due to other things.

IC: Okay, can. Then, how about the language, do you think there are things that may be confusing in the physical effects part?

HCP: I think those words that I have already highlighted like all these, ‘feeling tired’ and… Yah, I think I already pointed out those along the way.

IC: Then how about the appearance wise? So, the color scheme, fonts, font size, those things.

HCP: I think the font size is okay. Actually, come to think of it, it’s a bit mismatch like this… the top ‘Physical effect’ (title of slide), the font is like… actually okay, I think okay.

IC: It looks a bit different from the…

HCP: Yah, but I’m not sure whether if we change to like a more formal font, it will look all very like rigid.

IC: Okay, then how about the use of the icons, the images, the graphics here? Other than the one you mentioned just now, maybe we should add the person instead.

HCP: I think okay, as in the icons are used appropriately.

IC: Okay. Then the interactiveness, like the interactive buttons?

HCP: Can, just that I think need to fix all these. The ones that are not consistent. But other than that, I think okay. Actually, I feel if let’s say we want to keep this format right, then how come it… are you clicking on something? How come it comes out on its own? (Referring to some mouse control issues) [IC: I’m not but okay] You see it comes back on its own. Anyway, I feel if let’s say if we are going to keep to this bit, as in this format, whether can there be like for each of these, there can be some image to show. [IC: Okay] cons, rather than a list of words which… it’s a lot of words to read.

IC: Some visuals there will be…

HCP: Yah. Okay.

KY: Sorry, I interrupt a bit. What about you click into the treatment then the human body?

HCP: Can also, but I think some bits will be repeated, because you have… [KY: Yah, lymphedema] some of the effects are repeated, so if you have the human body like in front, then after that you click in, then you can have like the cause of it, like what is... it might be caused by your this this this.

KY: Yah, so the initial thought is because some patient they never go through certain things right, then they don’t have that… they also like… don’t really want to know or they will find it irrelevant.

HCP: To them?

KY: Yah, so that’s why we have the treatment then if you take that treatment then the side effects makes sense.

HCP: Mm. But I think some patients, depending on their literacy level, they might not know whether the one they are receiving is chemo or HER2?

KY: Mm, yah. Okay. Can, we’ll try.

HCP: Okay. Then I move on?

IC: Mm.

HCP: (Slide 33) Financial concerns can, rather than burden? I think we can stop here, as in the emotional support, then full stop, because we don’t need to really dictate who the patient can turn to.

IC: Okay. Alright. So…

HCP: That’s all right for this.

IC: So for the emotional effects, what are your thoughts about the content, the accuracy of the information presented?

HCP: I think okay.

IC: Then how about any other information that you think we should include for this part?

HCP: No, actually, I’m just thinking whether we need to list out the… like ‘feelings of depression, anxiety, or fear’. Or whether this should be like an example, so like ‘can bring emotional distress for example, depression, anxiety, or fear, even after treatment, survivors may also feel distressed’. So rather than saying that can bring these feelings, so we are kind of like saying that these 3 are the things that you will feel, but rather they actually can also have other feelings of distress. So these are just like examples but again I think, because like here you list examples, then below also you list examples, so may need to have not so many words.

IC: Okay, understand. Then how about the appearance wise? So the appearance features, the color scheme, choice of font, font size, for this part?

HCP: I think okay, but again the background, maybe not white. Sorry, the first paragraph, ‘even after treatment, survivors may still feel distressed for various reasons’, I think we don’t use the bracket so much, maybe ‘various reasons like concerns over…’.

IC: So remove the brackets and then…

HCP: Yah, I feel so.

IC: Then how about…

HCP: I wonder if it will look nicer if like this is standardized throughout, like instead of a bracket. Brackets feel to me a bit, not so…

IC: So have everything out there.

HCP: Not so nice. Like a lot of bracket everywhere looks a bit messy.

IC: Understand, okay. How about the icons, the images, the graphics here?

HCP: It’s okay, but the pink against white not very clear.

IC: Okay. Sorry, let me try something, [be]cause you mention that it was going to the next one even without you clicking right.

HCP: Yah, the radiotherapy.

(IC resolving issue)

IC: But generally, how is it so far like is it okay so far?

HCP: I think okay, quite easy to navigate, I think. It’s just maybe the aesthetics and the wordings.

IC: Is it like easy to know where each button to click for example?

HCP: Yah, quite clear.

IC: [Be]cause that’s something that I think some people had trouble with, like they think it might be confusing on where to click, so we just wanted to confirm also.

HCP: I think so far, quite okay.

IC: So far quite okay, that’s good. Hopefully, this will be better now.

HCP: Actually, if I’m a patient right, so are we intending for this shared care to be like with the polyclinic doctors or also the GPs?

IC: The idea is this is a[n] introduction to the different follow-up care models, so to the shared care model as well. So most likely it would start with the oncologists at NCC, but we could put it with the GPs.

HCP: (Slide 34) I’m just... [be]cause like other care providers in the community will not be very clear to a lay person. So who are you talking about? Is it my GP or is it the polyclinic doctor or is it somebody else? So I think we need to spell this out. They might not know even what is meant by ‘community’, and also… So if I’m a patient or a survivor, I probably wouldn’t… I wouldn’t be very curious as to why there are options (laugh). I mean… this one and whether it’s effective or not. Can we kind of… instead of saying that it’s equally effective, can we list out the advantages rather than… I mean if it’s equally effective, then I… then why do I need to choose shared care? I can also choose usual care. So maybe like rather than why there are options, maybe can be like click this, click here to know about the benefits of shared care or something like that. Then, I would be more inclined to click.

IC: But then for those kind right, would you think that if we add specifically like these are the benefits of shared care, will it look like it’s slanted or like biased towards shared care, for example?

HCP: I mean it would or… if so, if we are not trying to do that, then you can have 2 bubbles, so click here to know about advantages of usual care then this one. [IC: Have both sides] I mean it depends if we are… I don’t feel that there is a... it is wrong to kind of like advertise shared care. And then also, ‘cancer-related care are mostly provided’, ‘mostly’ meaning like 90% then how about like the 10%?

IC: Okay.

HCP: I think it’s just the wording, or can we just say that it’s provided.

KY: This one because sometimes they say like even their GP also take care of their cancer under usual care, that’s why try to save, like not be so absolute that cancer care is only provided by oncologists. That’s why it’s ‘mostly’.

HCP: Okay. Then can we be more specific? Can it be like, ‘what are your options’, then you have 2 diagrams. Then like one diagram can show like, I don’t know whether it can be like a pie chart or some diagram that shows that a large portion of the care is done by the oncologists. Then, a small portion is probably done by like the other primary care providers. Then you have a note below to say that… this is… have to reword, so have to say that this is only applicable for some patients. For some, it’s like 100% it’s oncologist, something like that. Then the other diagram will show shared care. So, like diagram or a chart or something, I think it will illustrate a bit better.

KY: Okay.

HCP: So, do I move on or IC you got something else to ask?

IC: No, not for now.

HCP: (Slide 40) I think when you click on the oncologist right, then you don’t need the statement. As in when you click on any of the icons, you shouldn’t need this ‘who will you encounter’ already, [be]cause it’s already here. And then when I click on oncologists, the oncologist bit should be up here (replace the subheading position). Then, I think the icons below should be spread out a bit more because it looks very crammed. (Slide 41) This one follows the NCC practice right?

IC: Mm, so like sister Mabel all those.

HCP: Okay. I don’t know whether we can call those… I don’t know whether we can call them special type of nurse. Can we just say that ‘an advanced practice nurse will see you, may see you during your consultations with the surgical oncologist’?

IC: So just remove the ‘special type of nurse’ that, those words?

HCP: Yah because it’s a bit weird. I think we can just say that an advanced practice nurse will see you. The ‘you may encounter’ is also a bit weird, what do you mean by you may encounter.

IC: So, you may be seeing an advanced practice nurse?

HCP: Yah. This one can we list out? The similar roles as an oncologist.

IC: Okay. So format can be more similar to the oncologist one?

HCP: Yah, so perform breast examination and like review your scans and whatever, then under the supervision of an oncologist.

IC: Okay.

HCP: I think Hospital should be capital. Then again, all these, this one can remove.

IC: The heading?

HCP: (Slide 42) Yes, ‘who will you encounter’. The icon you can move up to here. Family doctors and GP clinics, can we just say general practitioners as in like… to replace the whole line, general practitioners. Then this one, I just want to ask whether is it necessary for them to know the difference?

IC: For this one?

HCP: Mm [IC: I think], because I just don’t know the significance like whether it matters.

IC: Yah, so I think one of the reasons why we put it like… we kind of emphasize the difference of same doctors is that there are some patients who actually feedback that they don’t like that they are seeing a different doctor each time they go to the polyclinic and would prefer to see the same doctor. And then, if you do see the family physician, for example, at the polyclinic, so, you’ll be able to see the same doctor or at least have like only a few doctors like one group, small group of doctors seeing you when you follow-up there. But it will be a bit more costly. And it’s also kind of a lead on to let them know that there’s this family physician doctor at the polyclinic [be]cause quite a number don’t know about that, and then because our shared care model actually involves these family physicians as well.

HCP: Mm. Sorry, I’m just thinking about the phrasing. So this family physician clinic is something that you have to book specially right? Like if not then usually the patient will see a different doctor, is that right?

IC: Yah, so usually they would be assigned or at least they will go to see the regular polyclinic doctor, the general polyclinic doctor. But then for those who either ask for it or they like they request it or for those who are on like long-term kind of follow-up, especially for those with chronic conditions, then they will be…

HCP: Okay, understand.

IC: Show that there’s this option for them.

HCP: So, can we say that, you need to rephrase my words. But can we say that ‘polyclinic doctor, you will be seeing a different doctor each time unless you are booked into these family physician clinic’. So then your bubble will be in the family physician clinic, you will see back the… the same doctor will be seeing you. ‘Patient to see back’ is weird. I think ‘the same doctor will be seeing you’, and then consultation session cost more, so we need, if I’m the patient, I will want to know how much more. What’s the usual rate and versus this family physician rate. This one ‘you may have encountered’, I think don’t need, just type of family doctors then directly go to your family doctors, as in your GPs and polyclinic doctors. No need this phrase.

IC: Okay.

HCP: (Slide 35) This one, can we not have, or can we think of a better way to illustrate the, like the polyclinic, the GP, all these? Instead of having so much words here, then it will either be cancer centres - hospitals or cancer centres, then community. Okay. Can I move on?

IC: Mm.

HCP: (Slide 44) Will they [pharmacists] call? They might not ask right, community pharmacists.

IC: Might not, yah.

HCP: Like if I walk-in, I don’t tell them I got cancer. Even if like, yah, they might not call right. Can this link [to survivorship care plan] be like here? Then, you don’t need this. So if this one can be like the diagram that I was talking about – usual care, shared are – then when you click on it, then there’s a pop-up to see the difference.

HCP: (slide 43 to 44) I think the question doesn’t really link, because this one is your, so you are talking about their… he different are professionals right, then after that you click on the next one, it says ‘how are the options different’? It sounds like you are asking me how these people work differently? Because I think the options are actually here right, which is a previous slide. So actually, I feel, maybe you don’t even need to have this question. So you just go on straight to click on the following to find out how…

IC: How they are different.

HCP: Yah, how care is different. I am also thinking whether this are necessary information (referring to care communication tools across HCPs) to the survivor, so… can I just ask the, why we include these? It’s just to show the difference is it, between the 2 models?

IC: it’s also to say that there’s this communication that occurs currently between the oncologists and the advance practitioners and then with the primary care doctors. But then with the shared care, there is an additional form of communication through the shared care plan, the one that was in the middle. So, in a way, it’s also to provide a comparison, for like a better word. So, it’s like this is the current ways that they are communicating. But then in the shared care model, all 3 of them can communicate through the shared care plan, to make a kind of distinction.

HCP: So my thinking is that if it... if there is some kind of communication, actually it doesn’t matter to the patients what kind of communication, it’s like a backend kind of thing. I don’t know whether it’s something that we need to let the patient unless there is like for example, okay, so for example, you say… like may communicate via this methods then it takes a longer time but whereas like shared care, if you have a common platform, then it takes a shorter time. So then it makes a difference to me, right?

IC: Okay, yah, so…

HCP: So I think it needs to be something that has an impact and that has that significant to the patient.

IC: That patients is more…

HCP: It’s relevant to me, relevant to the patient.

IC: I understand.

HCP: Sorry, so, this thing will be given to... what’s our, what’s the target population? As in like is the patient going to undergo, are we putting these patients in shared care or what’s the… what’s our target? Who’s our target?

IC: So the context of this also is that this is for, this is to be presented to breast cancer survivors to show them that there’s this alternative care. And then, would you want to take the shared care model up, this alternative care up.

HCP: Oh, okay.

IC: It’s just to give them context and information about the alternative shared care model, and then they can decide by themselves, as in we hope that it will help them to see like if.. to see which form of care they will prefer to undergo for their, for example, their long-term follow-up, those kind of things.

HCP: Okay.

HCP: (Slide 46) How do I go back?

IC: You want to go back to the usual care, is it?

HCP: Yah, okay.

HCP: This one can we, I think the words in this slide should be, click on the buttons to show how our follow-up care schedule will look like under usual care or shared care. I think don’t need so many lines, then you will have a lot more space for your diagram. Then the usual care, can we make it the same like the shared care? Like this one got a lot of animation but that one like don’t have, like that only.

IC: Actually, there was the animation.

HCP: Maybe not so much, just…

IC: Sorry, I’m pressing. But not as much as the shared care one.

HCP: And also, I feel like ‘1-2 visits to oncologists in the cancer centre’, I think this surgical, radiation, all these, no need. Then this one no restrictions to the number, meaning it’s for the… it’s for the chronic condition, right? It’s not for like the cancer follow-up right?

IC: This one for the usual care, it depends on why the patient goes there, so…

HCP: So it’s your survivorship care should is just the visits to the oncologists, correct?

IC: The one mostly, at the oncologist ones. But there still might be…

HCP: So actually, I feel this one no need, the ‘no restriction to the number of visits’, because if we are talking just about… okay so then maybe on top, we can say your follow-up schedule for survivorship care. Then, this bit, I think ‘no restriction’ can maybe just be like a small print kind of thing. Because if we put it here, it looks like it’s part of your…

IC: Survivorship care, is it?

HCP: Yah, I mean it might be, but it is not the main thing. The main thing is that you still have to go back to the oncologists. Then, this bit also, the ‘as many times as appropriate’ is for your own condition, is not for… correct, it’s not for the…

HCP: (Slide 48) This one, I think don’t need this... just say ‘summary of the available options’ can already, or differences between the options. A summary of the differences. Then don’t need two lines, just one line.

IC: So remove ‘the following…’.

HCP: ‘the following table’ yah, I think it’s quite clear that it summarizes. So just a subtitle will do. Then, I think like between oncologists and advanced practice nurse, there should be a space [line spacing], then like this bit also, between each bullet point, there should be space [line spacing]. [Be]cause it’s all like very crammed together, and whether maybe like it can be a smaller font so that…

IC: There’s enough space in between also?

HCP: Yah.

IC: Anyway, removing the, that line above may give more space as well.

HCP: Yah.

HCP: (Slide 49) Then this one also like between each point, there should be a space [line spacing].

IC: (Slide 50) I think here is the general clinic the family physician clinic, the cost different that you are asking about just now as well.

HCP: Yah. So actually, there’s no difference, are we saying that there is no difference?

IC: As in between the…

HCP: [Be]cause this is also free, right? And you are put-… we are putting everything…

IC: So for this one, the idea is also, especially the primary care doctor, it would depend on the number of times that they go, because depending on their condition and their own preference, they might go once a year or they might go like 3-4 times to the primary care doctor, for example, [in] a year. And then oncologists, 2 times a year. So, this one we can’t give a kind of overall cost because each persons’ visit schedule will be different.

HCP: But can we say that a bit clearer in this? [Be]cause if I look at this slide right, I click click click, there’s actually no difference, so we cannot say that there’s key differences between the two options for considerations, because I don’t need to consider anything, [be]cause everything is the same. But, I think if you talk about like, so for example, if my survivorship care is in NCC and I don’t opt for the shared care, then I still need to go to the GP for my chronic conditions. But, if I opt for a shared care, then I can see together, my survivorship and the chronic conditions. So I think that has to be a bit clearer. I think don’t really have to like say how much, like we don’t have to estimate how many times the patient goes, but rather, just a statement to say ‘additional charges depends on your whatever conditions’, kind of thing. Then the shared care will be, like we just have to say like, you can see together. So then, the difference is a bit clearer [be]cause like that, no difference.

IC: Can, understand. So sorry, so for the previous 2 sections, so the comparing the options as well as the previous one where the information about the follow-up options were show. So just wanted to go through those questions again. What do you think about the… do you think that we should add other kinds of information inside or are there things you think are redundant that you haven’t mention already?

HCP: No, I think I mentioned already.

IC: Then how about the comprehensibility, do you think there are things that maybe confusing to the patients?

HCP: I think no, I think quite okay.

IC: Then the use of the… so the appearance features, the font color, those things.

HCP: I think quite okay, but, so for example, this one. (Slide 49) Maybe you can… the between the usual care and shared care, it should be further apart. So shared care should be here, so that there’s a bit [IC: there’s a gap]. I mean this slide, this small gap looks like it can almost continue, so I think needs to be a bit more gap.

IC: More distinction between the two.

HCP: Yah. Then this follow-up schedule can move somewhere I think, or it can be like usual care follow-up schedule, shared care follow-up schedule, rather than here, then it takes up like quite a bit of space.

IC: Then how about the icons, graphics?

HCP: I think okay. Just that these people look a bit similar (doctor graphics). I mean I wonder there are like icons that are more, look a bit more different from one another to differentiate the different roles.

IC: Then the navigation wise, it’s, is it easy to navigate all those things?

HCP: I think okay.

IC: That’s good. Clicking all those…

HCP: Yah, I think it’s fine.

IC: The Next section.

(IC resolving clicking issue)

HCP: (Slide 54) Nothing to click, is it?

IC: Yah, there’s nothing to click here. You can click the… yes.

HCP: Can I go to the previous slide? I think no need, is there why we need… is there a reason why we put this scale, or can we just have a very direct introduction to say that the following slides help you to choose.

IC: I think one of the reasons why we put this is because the patients didn’t really understand like what was this about. So, we tried to put a visual there to help. But do you think that if we just state in words, then is it easy to understand like...?

HCP: Yah, I think it’s okay. I mean I think… Yah, I don’t feel like the visual really is like help me with comprehending, like understanding anything else. I think it’s important to just be direct as to what we want the patient to do. Like so now that you have seen all those things, please decide or the following helps you to decide. Something like that, very straightforward.

IC: Okay.

HCP: This one I click?

IC: Mm.

HCP: (Slide 55) Yah so you see here, I’m willing to pay more to see the oncologist, it’s not very like very clearly displayed in the previous slide when you showed the cost.

IC: Because it seems like it’s the same thing for both sides right, just now

HCP: Yah, I mean I have to think a bit, like a few steps more before I [think] “yah, actually I see the oncologist is more expensive”. So, this one, okay. I think there needs to be space between each option, so that it’s clear that it’s a different option. Then also, in terms of the alignment, so like the doctor visit… the doctor visit should start at the ‘it’. You get what I mean? Rather than starting from the box here. So, it should be aligned. All the words should start from here.

IC: Okay, I see what you mean now.

HCP: Yah.

IC: Understand, we’ll fixed that, the alignment thing.

HCP: And whether it can be like not a table form. But that one have to think about it. Or maybe we can, you can have like 3 slides, the first slide would be practical considerations. Then you have the options, then the second slide ‘involvement of care providers’, rather than all these together. But I think it’s fine, it’s just that if you cram all these, iit’s not so nice aesthetically.

IC: It looks a bit crammed right now, is it?

HCP: Yes, a lot of words and quite crammed.

IC: We’ll see what we can do about that.

HCP: Okay.

IC: So sorry, for this part right, so these are some of the factors these are the factors that patients and some other people have feedback that these will be important to them.

HCP: Sorry, how do I go back.

IC: That these factors are important to them when deciding what kind of care that they would want to tale up. So, I just wanted to ask you like do you have any other, do you think that we should add other kind of factors, or what are other factors that we should add inside here?

HCP: Actually, the usual care right, the patient may also be staying near to the cancer centre. It might not be a longer distance.

IC: Yah, I think that’s why the shared care one, we actually put it more convenient location. This is more relevant to those who are staying a bit further away. I think Outram is quite central as well.

HCP: Yah.

IC: it’s just those at the ends maybe a bit, they might prefer, as in that’s why we have the option, if they can go to somewhere closer to them.

HCP: The other services means what, here?

IC: Other services like the physiotherapy, those things, dieticians.

HCP: Okay, so I think have to be clearer, oncologists, there shouldn’t be a stroke [‘oncologist/’ displayed on slide] here right?

IC: Okay, yes, we’ll remove that.

HCP: Usual care, can we talk about like something on the pharmacist as well? Like if I have any questions, I can always approach the cancer centre pharmacists also.

IC: Don’t need to go to the community.

HCP: Yah, the community. Then the shared care one, I can obtain health advice readily from, we are talking about the community pharmacists right?

IC: Mm.

HCP: Appealing means what? It’s appealing to me.

IC: Like it’s a good idea that they will like this common care plan for themselves, or for the providers to have this common care plan.

HCP: Okay. Can it be something like the use of a common care plan will be... or it will be useful to me or to my care, something like that, rather than appealing. Maybe a bit more like explicit.

IC: Okay.

HCP: Like to enhance communication or what.

IC: Like to what’s the difference between the common care plan and the usual care kind of communication.

HCP: Yah. Okay. Nothing else to add.

IC: Okay then, sorry, so the aesthetics wise about this part?

HCP: Yah, so I think a lot of words. So, if maybe 3 slides then… can I see the conclusion first? Okay. Sorry, can I go back. Okay. I feel… I don’t know whether it’s possible to like calculate for them.

IC: So like put a number there for them?

HCP: So it says… so for example, I tick more on the shared care, then this like preference scale should maybe highlight this part, I am willing to try out, then this one will be pink(?) because you actually click more options on the shared care. Is that what we are trying to guide them towards, that if they click more on a… on this part, then they are more inclined to try?

IC: Yah.

HCP: Right? So I think this thing that pops up should show that, because this one right, like even after I click then I… this thing pops up, it’s the same, no matter what I click here, whether it’s more of usual care or more on shared care, this thing will be the same. So but after you ask me to choose all these, I expect this thing to be a bit different, depending on what I choose.

IC: I think one of the reasons is because PowerPoint is not able to do those things [HCP: Okay], because the eventually we want to put it on a website format, so it might be easier to put it there.

HCP: You see whether it’s possible, but I think it’ll be nice if like... Or you know like one slide on your results, something like that. Then it makes me feel that I tick this for, like it’s worth ticking you know, I got a result.

IC: The navigation wise, the ticking is easy to click?

HCP: Yah, it’s okay

IC: Then the conclusion, so that’s mostly it. Thank you. We do have an additional resources part. So that one, just need your help to look into like each one to see if its comprehensible enough or if we should add other kinds of websites or topics inside here. So these would… when you click on the link, then it will go into the website.

HCP: Okay. (Slide 63) I think need to be a bit more… it’s a bit scattered, as in like the layout. Can we have a, like maybe a box here with color then your HealthHub, NCC; then, overseas-based websites, then a box. So that it differentiates a bit better. Do I need to… I don’t need to see the contents right?

IC: Don’t need.

HCP: Sorry, what do I need to?

IC: So for this part, do you think that we should add other topics or other kinds of links those things inside the specific topics? Or do you think that this is actually comprehensive enough?

HCP: What about like the ‘living well after cancer’? The part on healthy living, healthy lifestyle.

IC: Right.

HCP: Yah.

IC: Okay. so like…

HCP: Is it here… no. Okay.

IC: So it’ll be things like…

HCP: Like living healthy, like exercise.

IC: Diet those things.

HCP: Diet, yah.

IC: We do have some healthy eating, but I think it’s a slightly different thing…

HCP: Self-help…

IC: …from what you are referring to.

HCP: Maybe we can change this title to…

IC: To health living, I mean…

HCP: ‘Life after cancer’ or something like that. The support group, there are also other societies, I don’t know whether y’all want to consider putting in like the 365 cancer prevention society, the AIN society. Yah so there are also a few more. But it’s available on the NCC website.

IC: It might be good to just put it here explicitly as well?

HCP: Yah. Support groups and other support services, other services.

IC: So like for example, for the other services, we do have the for example, the ‘Look good feel better’ those kind, so maybe if we do add the living after cancer one, then we should move things like these under there?

HCP: What is this actually? Oh, I cannot click.

IC: I think can try, don’t know if it will open.

HCP: I think the ‘Look good feel better’ is, I thought it’s like during cancer treatment one that they teach the patients how to do make up and putting on wigs and all those. Can we see what’s the content?

IC: There’s also a part about the… let me see if I can open. So it’s also the cosmetic those kind, okay.

HCP: Yah.

IC: This one is during [treatment]. But how about those that for post treatment, for example? Like if it’s about post-treatment workshops those things?

HCP: Post-treatment, you have to… I think can go to… this one I’m not sure, but I think can go to the breast, the BCF page and see whether there are some programs that are post treatment. I think for NCC side, it’s not very specific and we have removed a lot of programs because of COVID.

IC: Then we will look into this. Thank you.

HCP: Yah, so the ‘Look good feel better’ one is during treatment.

IC: Yah sorry, that one I got a bit confused.

HCP: And actually, I am wondering whether it’s, it will be a bit clearer to the patients if like everything is listed on one page.

IC: For the resources?

HCP: For the resources, so that I don’t have to click in [and] click out.

IC: So everything in one? Okay, understand what you mean. Okay, can. Then that’s mostly it for the decision aid, thank you so much. I do have a few questions.

HCP: Sorry, I cannot hear you.

(Sorting out sound issues)

IC: So I was saying that this is the mostly it for the decision aid. Thank you so much. So I just have a few follow-up questions for you. So how difficult or easy do you think that it would be for you to introduce this decision aid to your patients?

HCP: I think should be okay (laugh). I just need to tell them to go through, should be quite easy.

IC: Then would you feel comfortable discussing the information presented in this decision aid with your patients?

HCP: Yah. Do I need to rate like 0-10 kind of thing?

IC: Later there is a scale rating as well. So, we’ll go through that later. Then, what feasibility aspects of the decision aid you think that we should be considering or improve before it can be rolled out to the other patients?

HCP: Sorry, can you repeat?

IC: What feasibility aspects of the decision aid should be considered or improved before it can be rolled out to the other patients?

HCP: Feasibility meaning? Can you give some examples?

IC: So like, for example, the ease of use, all those kind of things, relevancy.

HCP: I think it’s all… it’s what we have covered earlier, the feedback that I have given so far. I think nothing else. So, this one will be provided as in, will give them a… it will be shown to them on like iPad or they have to use their computer?

IC: So actually, that’s also some of the things that we would like to ask your opinion about, do you think it will be better if we show them on an iPad for example when they are waiting at the clinic, or we just send them the link then they can click themselves, or maybe both, if they want?

HCP: I think both will be good, I mean if we want a higher uptake I think, because I mean I feel some patients would probably like that to have something to do during the wait, but some patients will be too worried to do anything. So, if we can have a few like both of the avenues, then maybe it’ll be better. Of course, I think it’ll be good if the… if a nurse actually sits there and go through, but then it’s also resource, so not sure whether, or not a nurse but maybe someone else. Not sure whether it’s something that we are able to do. Usually, I think they will do better if someone sits with them and go through.

IC: Understand. Then, actually just following up on that as well, so other than nurses, who do you think would be good to introduce or go through this decision aid?

HCP: Actually, I think some of the things are quite straightforward, I mean the information is already inside, so whoever who can navigate through. Don’t really have to be nurses, I feel. Yah, I think even RCs [research coordinators] are okay (laugh). Yah, I mean unless they have got different as in like additional questions which the person cannot answer then of course, it’s okay to say that we will find out more and then let you know.

IC: Okay. And then, when do you think we should introduce this decision aid to the patients? Like so introducing also the alternative care model for the patients.

HCP: I think it’s okay to introduce when they come for their appointments, at the… after they have reached this point like the 3-years post treatment point.

IC By post treatment, you mean by post chemo[therapy], surgery, radio[therapy] right?

HCP: Yah, as in like when they have, when they are eligible.

IC: When they are eligible…

HCP: For the shared care model.

IC: Okay. Actually, that was the other thing that we wanted to ask, when you think, for example…

HCP: But isn’t that what you wrote down in the previous slide, it’s 3 years post right?

IC: Yah, we did list 3-years post. So, but then because sometimes, so we just wanted to see like will it be better if we show it before like when they are doing their active treatment or after they finish, right after they finish active treatment or a few years after like you mentioned, when they are eligible.

HCP: I think when they are eligible, it’s better. Because when they are having treatment, I think usually they have a lot on their mind, and this is something which is… So, then, I mean if we introduce too early, then what happens if they actually do recur? Then a bit sian right. So, I think, it’s better to introduce when they are actually eligible.

IC: For the patients who are a bit more stable also?

HCP: Yah, and not so much on their plate.

IC: Okay.
