# Supplementary material for: Development and pilot testing of a decision aid for navigating breast cancer survivorship care
Source: BMC Med Inform Decis Mak. 2022 Dec 15;22:330. doi: 10.1186/s12911-022-02056-5 (PMC9753367; doi:10.1186/s12911-022-02056-5)
Supplement: Supplementary file 5 — Additional file 5. Transcripts and the final decision aid prototype. [file 12911_2022_2056_MOESM5_ESM.zip › Additional file 5/HCP07_transcript.docx]

**Study ID**: HCP07

**Interviewer**: IC

**Date**: 31 March 2022

**Transcribed by**: KY

(Remote control not working, IC explained about assisted navigation)

IC: So you can look through this. And then once you are ready, you can let me know.

HCP: Yup, okay.

IC: Then I will press the bottom part.

HCP: Yup, okay, can.

(Reviewing)

IC: So for this part, what did you think about for example the accuracy, comprehensibility, and the format of this part? So like maybe we can start off with do you think that it’s accurate or do you think there’s other things that we should include for this portion?

HCP: I don’t think so, I think this is fine.

IC: Okay. And then, do you think that it’s easy to understand or are there things that might be confusing to patients or survivors?

HCP: You give me a second. Actually, I think it’s fine because the whole idea of it I think should be simple and easy to understand, so I think this is actually fine. Are the orange words actually clickable?

IC: No, they are not.

HCP: Okay, yup, alright.

IC: And then how about the appearance wise? What do you think about this portion aesthetically? So like for example, the color scheme, choice of font, font size, those things.

HCP: I think that’s fine.

IC: Fine? And the icons, images, graphics, all okay?

HCP: I think it’s okay. I mean the pink ribbon logo tell me this is actually for breast cancer, and I suppose with that inference, that circle there refers to the breast. But on first appearance, it may not be very apparent that that is supposed to be the breast.

IC: Okay, [HCP: yah] understand. Then I will go to the next section.

HCP: Give me a second. Sorry, yup, okay.

IC: So maybe you can tell me which part you want to press?

HCP: The long-term effects.

IC: Okay.

HCP: Okay, and then how about the late effects? Okay, yah, I wonder if we could… I think this is okay.

IC: You think we should had something else inside?

HCP: It’s just that because the cross and the late effects and the long-term effects, I know what you’re trying to say, but then I wonder if maybe that’s better represented by say like a… an arrow or something like that that shows of that period instead.

IC: Okay, understand.

HCP: Yah.

IC: Alright, so maybe the presentation of it can be [inaudible].

HCP: Yah, that’s right.

IC: Okay, so the next part.

HCP: Yup, can go. The surgery?

(Reviewing)

IC: So for this part, similarly, what do you think about the accuracy…

HCP: You know the initial part when you actually come to this page right, there is actually all the flashing arrows, is it? Can you go to the… oh, okay can. So I’m not sure whether the flashing arrow is really needed. Yah, but I mean that’s just a side point. That’s really up to you all as in the aesthetic team to kind of manage that. But I’m thinking that it may not really add… but I like the fact that when you hover around each of the icon, it will then light up, so I think that’s great.

IC: Okay. How about the information that was provided about the accuracy?

HCP: Can I just double check that this information is actually targeted at primary care physicians? Am I right to say that?

IC: Sorry?

HCP: This information is targeted at primary care physicians? As in GPs right?

IC: This information is…

HCP: As in this decision aid will be used by GPs correct? As in by the primary…

IC: So this one is… this decision aid is actually, it’s not confirmed who will be using it but the target population will be the breast cancer survivors to let them understand what is the care model.

HCP: Can, so that means it’s not targeted at a medical doctor per se, that’s what I’m asking. If it’s for the survivors, then I think that’s fine, that’s more than enough information.

IC: Okay, can. But the doctors might be the ones going through with the patient, but the target population is …

HCP: Yup, I think that’s okay. Yup, I think that’s fine.

IC: Then, any confusing things that… anything that you think might be confusing for the patients?

HCP: Sorry, give me a second. Okay, sorry, yup, go on.

IC: So I was asking just now if you think that there’s anything that might be confusing for the patients, for this portion?

HCP: No, I think it’s fine.

IC: Okay, then how about the aesthetics part?

HCP: Sorry, I think you may need to kind of mention that you may be given once or more of these treatment(s), as in the treatments are not given… are not in isolation.

IC: Okay, good to clarify that.

HCP: And the likelihood is that you’ll be given one or more of these treatment(s).

IC: Yup, okay can. And then how about the appearance wise?

HCP: I think it’s fine.

IC: Okay. So that’s this part.

(Reviewing)

HCP: Yup, I think this is fine.

IC: Anything that you think we should add for the emotional effects?

HCP: Is this the only page for emotional effects?

IC: Yes, there’s only one page.

HCP: Okay, then I would suggest that it’s perhaps important to take a mention that for them to seek help at any point that they feel like they need help. Sometimes… as in what I’m trying to say that they should probably voice out the moment they feel that a negative feeling or something is persisting for too long a time. So something that perhaps is a little bit too stressed out, they should begin to consider speaking to a counselor or to seek medical attention.

IC: Okay, can. And then, is quite easy to understand, this part?

HCP: Yes, I think it’s fine.

IC: Okay, and then appearance wise?

HCP: Hang on, sorry. Yup, I think that’s fine.

IC: Okay.

(Reviewing)

HCP: Just the wording itself, you may be seeing a surgical and/or radiation and/or medical oncologist.

IC: Okay.

HCP: The blood/ imaging is not very clear. It took me awhile to kind of like relook it to understand what you mean is blood test/ imaging test. And also, for the general population, they may not understand what it means by imaging. So you may need to kind of be very clear that in this case it’s probably a mammogram or CT scan.

IC: Okay. So they understand it as mammogram, like MRI and CT scans they only know it… they mostly know it at scans is it?

HCP: Yes, correct.

IC: Okay.

HCP: Yah, and then your imaging. It may be… maybe… but I think somehow or another the blood/ imaging doesn’t actually come out that clearly and it… the part about how important tests and scans that may be ordered… it’s not that... It took a while to register that the pictures below actually goes in line with the last line.

IC: Alright.

HCP: Okay, can go ahead.

(Reviewing)

HCP: So I think the two way arrow is great but the top arrow with the square and the arrow coming out, I’m not sure what that’s supposed to mean.

IC: This one, is it?

HCP: Yah, correct.

IC: So this one would be referring to the communications, so for example, the memos and referral letters but I think it’s not as obvious.

HCP: Yah, actually, I think you should just keep it simple as in just the arrow alone would be sufficient.

IC: Okay can.

HCP: Basically, your idea is just to bring across that there’s a continuous information, right, exchange among the main stakeholders. So, I think you just need the arrows. But I mean that’s what I personal thought.

IC: Understand, okay.

HCP: Yup, otherwise okay.

IC: Okay. So, press the other one, the shared care.

(Reviewing)

HCP: Okay, this one looks like a calculator.

IC: Okay, can.

HCP: I think otherwise it’s okay. Yup, that’s fine.

(Reviewing)

IC: So for this portion, so just now that whole part, what do you think about the accuracy of the information? Or are there other things that you think we should include as well?

HCP: Actually, I understand that you are trying to … I mean it’s great that you all explain the tool, but I wonder whether it will be a… if you also have something that kind of like contrast the two forms of care, either like one on top, one below, then that one like at one glance, you kind of like can see the difference.

IC: Okay.

HCP: Yah, so, maybe what you can do is after this slide, you know you have another slide, cos I understand you don’t want to crowd the whole thing with too many words as well, so if you have another slide that kind of shows one on top of the other, the two models and then like what the difference is, maybe that maybe helpful.

IC: Understand, actually it’s going to come up in the next section.

HCP: Okay, alright, great.

IC: But this just the… I know what you mean. This is just the introducing the information. We also understand that it’s easier to see at one go also since there’s quite a lot of things going on. Then, anything that you think would be confusing for the patients or the survivors?

HCP: No, I think it’s fine.

IC: Okay, that is good. Then appearance wise? The aesthetics.

HCP: Yup, I think that’s fine as well.

IC: The graphics, the images, all are okay?

HCP: Yah, I mean I noticed that you tried to use the same picture for the same… to kind of show the same person, right? Yah, so I think yup.

IC: Then the next part is the, so we actually compare side by side, rather than… [HCP: oh, okay] up and down.

HCP: Okay, I think that’s fine.

(Reviewing)

IC: So that’s mostly the information that is previous… in the previous section but in a table comparison format. What do you think about the appearance wise?

HCP: Appearance of this current slide?

IC: Of this section, the tables.

HCP: I think it’s okay. I think it’s alright. I mean this is just what… these are just the facts that has to be there.

IC: Okay. Do you think anything would be confusing?

HCP: No, I think it’s okay. I think it’s alright. I mean I noticed that you all tried to keep the logos as much as possible standardized, so I think that’s great.

IC: Okay, that’s good. So this next section.

(Reviewing)

HCP: So what is let’s say for example, the practical considerations, I have one on the left side and then the involvement of care providers, I have one option on the right side, is that okay?

IC: Yah, it is.

HCP: So the idea is that the… you end up choosing the one where… which has the most ticks.

IC: Yes.

HCP: Okay, can. Okay, alright, can. [Be]cause it’s a… Yah, that I’m not sure if it’s entirely clear though. Okay. [Be]cause … that may not come across immediately.

IC: Okay. Maybe I can press this… so after they finish choosing, then this actually comes out. Like you mention, it’s… whichever side has more… it’s kind of like guide whether or not they think that based on their preferences, would they, do they sound like they are more willing to share… to try out the shared care or would they prefer to continue with usual care. [HCP: mm] So for this part right, so these are some of the factors that based on previous sharings as well that patients would feel that it’s very important for them when making a decision about the follow-up care. So I wanted to ask do you think that other… are there other factors you think we should add inside, for example.

HCP: Are there other factors… I think it’s okay.

IC: It’s quite comprehensive for…

HCP: Yah.

IC: Do you think there’s anything based… the factors here that we present, anything that might be confusing?

HCP: No, not really.

IC: And then how about the appearance, aesthetics wise of this part?

HCP: It’s okay also.

IC: Okay.

HCP: Yah, just that I… yah, I think that’s fine, I think that’s okay.

IC: I realize maybe I should tick some to let you see how it looks like also. So once they... if they want to check the boxes, then they can press to check the boxes, something like that.

HCP: Okay, but many… yah… okay.

IC: Something like that, then after…

HCP: My suggestion is that I just wonder right if let’s say it will be a good idea for you to take away the ‘I prefer to continue usual care because’ and ‘I’m willing to shared care’, at the start when they are ticking the boxes.

IC: The… these top headings, is it?

HCP: Yah, then you just ask them to tick whatever boxes it is. Because whoever that is reading it, they may think that “I’m looking at it, so I prefer to tick the usual care box”, so I only look at the left-hand box. Obviously, you have somebody who is administering this then that’s not so much a problem. But if they are doing it themselves, then it may be a little bit of an issue.

IC: Understand, okay. So I’ll go to the next part?

HCP: Can.

IC: So that’s mostly it for the decision aid. We also do have a portion for additional resources, maybe I’ll just briefly go through, press into each one then you let me know any other things that you think we should add in as well.

HCP: Okay.

IC: Or like if there’s anything that’s confusing, or aesthetics wise, these things.

HCP: Okay.

(Reviewing)

IC: SO what do you think about the online resources portion?

HCP: Yup, I think that’s fine. When you have too many things inside, also it’s very difficult. SO I think that’s alight. You have a select few I think that’s okay.

IC: Understand, can. Then, alright, so I just have a few follow-up questions with the decision aid. So, how easy or difficult do you think it would be for you to introduce this decision aid to your prospective patients? So for example, providing a link for online access.

HCP: I think it’s pretty easy.

IC: And then, would you feel comfortable discussing the information presented in the decision aid with your patients?

HCP: Yup.

IC: Okay, that’s good. Then, what feasibility aspects of the decision aid should be considered or improved before it can be rolled out to the other patients?

HCP: What again... what feasibility aspects?

IC: Feasibility aspect.

HCP: Language.

IC: Language, is it?

HCP: Correct, [be]cause you may not have everyone who speaks English. Then the other thing is you are assuming as well that the person is able to toggle the tablet and the… [be]cause they may or may not be able to do that.

IC: So we might have to find ways to accommodate for different languages and for different access.

HCP: Levels of technical competency, I suppose.

IC: Okay. So overall, are there other information that you think the… this decision aid should contain?

HCP: No, I think this is fine. I mean the whole idea is just to explain to them whether that there is an additional model of care that can be considered, right? So I think that is fine, serves its purpose.

IC: Okay, that’s good. Then, generally, not confusing, easy to understand for patients?

HCP: Yes, and I like it that it’s not too long as well.

IC: Okay, that’s good. And then overall, how about the appearance, the aesthetics wise?

HCP: Very pleasant.

IC: Okay, then the interactiveness and the navigation part, I know you didn’t manage to do it by yourself,

HCP: It’s okay. You click it for me, I think that’s fine. I think that’s okay.

IC: But do you think that for patients, it’ll be easy for them to navigate if they are able to navigate?

HCP: Yes, I think so.

IC: That’s good. Thank you! And then, I just have a short questionnaire that maybe I can share it with the screen.
